# A Multicenter, Open-Label, Controlled Phase II Study to Evaluate Safety and Immunogenicity of MVA Smallpox Vaccine (IMVAMUNE) in 18–40 Year Old Subjects with Diagnosed Atopic Dermatitis

**DOI:** 10.1371/journal.pone.0138348

**Published:** 2015-10-06

**Authors:** Richard N Greenberg, Yadira Hurley, Dinh V. Dinh, Serena Mraz, Javier Gomez Vera, Dorothea von Bredow, Alfred von Krempelhuber, Siegfried Roesch, Garth Virgin, Nathaly Arndtz-Wiedemann, Thomas Peter Meyer, Darja Schmidt, Richard Nichols, Philip Young, Paul Chaplin

**Affiliations:** 1 University of Kentucky School of Medicine, Lexington, KY, United States of America; 2 Saint Louis University, Department of Dermatology, Saint Louis, MO, United States of America; 3 Rx Clinical Research, Inc., Garden Grove, CA, United States of America; 4 Vallejo Dermatology Office, Vallejo, CA, United States of America; 5 Hospital Regional Lic. Adolfo Lopez Mateos, ISSSTE, Mexico City, Mexico; 6 Bavarian Nordic GmbH, Martinsried, Germany; The George Washington University School of Medicine and Health Sciences, UNITED STATES

## Abstract

**Background:**

Replicating smallpox vaccines can cause severe complications in individuals with atopic dermatitis (AD). Prior studies evaluating Modified Vaccinia Ankara virus (MVA), a non-replicating vaccine in humans, showed a favorable safety and immunogenicity profile in healthy volunteers.

**Objective:**

This Phase II study compared the safety and immunogenicity of MVA enrolling groups of 350 subjects with AD (SCORAD ≤ 30) and 282 healthy subjects.

**Methods:**

Subjects were vaccinated twice with MVA, each dose given subcutaneously 4 weeks apart. Adverse events, cardiac parameters, and the development of vaccinia virus humoral immune responses were monitored.

**Results:**

The overall safety of the vaccine was similar in both groups. Adverse events affecting skin were experienced significantly more often in subjects with AD, but the majority of these events were mild to moderate in intensity. Seroconversion rates and geometric mean titers for total and neutralizing vaccinia-specific antibodies in the AD group were non-inferior compared to the healthy subjects.

**Limitations:**

The size of the study population limited the detection of serious adverse events occurring at a frequency less than 1%.

**Conclusion:**

MVA has a favorable safety profile and the ability to elicit vaccinia-specific immune responses in subjects with AD.

**Trial Registration:**

ClinicalTrials.gov NCT00316602

## Introduction

Smallpox is caused by Variola virus (VARV), an orthopoxvirus of the *Poxviridae* family. The disease was declared eradicated in 1980 by the World Health Organization (WHO). Serious concerns about the re-emergence of VARV persist and have led the US government to assess the potential reoccurrence of smallpox as a major bioterrorism threat [1.2].

Traditional smallpox vaccines contain replication-competent vaccinia virus (VACV), an orthopoxvirus related to VARV [1,2]. These vaccines are administered via scarification to the skin, causing a localized VACV infection that elicits a protective immune response to VARV. Although these vaccines are highly effective, their replication-competent nature can cause morbidity or even mortality. Skins complications include progressive vaccinia, eczema vaccinatum (EV), generalized vaccinia and autoinoculation [3–5].

Individuals with eczema, Atopic Dermatitis (AD) or a history of these conditions are at an increased risk of developing EV [6–8]. Historically, EV occurred at a rate of 39 cases per million vaccinations in the general population and was not limited to the vaccine recipients as contact transmission of VACV is possible [4,5]. Virus shedding following vaccination places individuals in contact with vaccine recipients at risk; there is evidence that EV may be more severe in contacts compared to vaccine recipients [4,9–11]. These observations led the Centers for Disease Control and Prevention Advisory Committee on Immunization Practices to advise against the use of traditional smallpox vaccines in individuals with skin disorders including eczema, active AD, a history of eczema or AD, and individuals who have household contacts with skin disorders.

The prevalence of skin disorders like AD or eczema could pose a serious challenge to any large-scale vaccination program that relies upon traditional smallpox vaccines. A recent survey of eczema prevalence in the USA found that as many as 31.6 million individuals met the empirical definition of eczema (i.e., itching and inflamed rash, excessive dryness, skin fold location, symptoms lasting ≥ 14 days, or associated with a physician diagnosis of asthma or of allergic rhinitis or hay fever) and 17.8 million met the more rigorous criteria for AD [12]. Further complicating any smallpox vaccination program is the observation that individual recollections regarding a history of eczema or AD are unreliable. Studies suggest that 20–40% of individuals do not report that they or a close contact suffered from eczema or AD, even when medical records confirm the contrary [13,14]. Given the various contraindications (e.g. AD, HIV infection), up to 25% of the population would have to be excluded from vaccination [15]. Therefore there remains a need for a vaccine that can be used in individuals at increased risk of developing severe vaccine related complications.

MVA was used during the 1970s in more than 120,000 people for priming prior to administration with a traditional smallpox vaccine [16,17]. MVA was derived from the parental VACV, chorioallantois vaccinia Ankara, and essentially became replication restricted to avian and certain non-human mammalian cells [18]. MVA is being developed as a safer stand-alone smallpox vaccine. Clinical studies so far have shown that MVA is well tolerated in healthy adults and those with HIV and induces immune responses comparable to traditional smallpox vaccines [19–23]. These clinical data are supported by similar findings in animal models [24–27]. Sera from MVA vaccinated subjects neutralize VARV [28]. In a phase I clinical trial, subjects with mild AD tolerated MVA well and had humoral immune responses comparable to responses in healthy individuals [23]. We report the results of a Phase II clinical trial more thoroughly investigating the safety and immunogenicity of MVA in individuals with mild to moderate AD or a history of AD. The primary objective of the trial was to assess the humoral immune response induced by MVA in subjects with diagnosed atopic dermatitis compared to healthy subjects measured by an enzyme-linked immunosorbent assay **(**ELISA). The primary hypothesis is that the humoral immune response of the group with diagnosed atopic dermatitis was not statistically inferior compared to the group with healthy subjects, using the seroconversion rate measured with ELISA 2 weeks after the second vaccination with MVA as the primary endpoint. The main secondary safety objective was to detect all adverse events in the atopic dermatitis group with an incidence of at least 1:100 with at least 95% probability. Other secondary objectives were the comparison of safety and reactogenicity in both groups.

## Methods

### Study design and subjects

MVA smallpox vaccine (IMVAMUNE) was produced by IDT Biologika GmbH (Dessau-Roßlau, Germany) according to GMP and provided by Bavarian Nordic A/S (Kvistgaard, Denmark) in aliquots of 0.5 ml liquid-frozen vaccine containing at least 1 x 108 TCID50/ml. Subjects were immunized at Weeks 0 and 4 by subcutaneous injection of a 0.5 ml dose.

This controlled, open-label Phase II study was conducted under an FDA IND. Enrollment of 18–40 year old vaccinia-naïve volunteers took place at 17 sites in the USA and 7 sites in Mexico, all according to Good Clinical Practice and the principles of the Declaration of Helsinki.

Subjects enrolled in the control group were healthy without any history of atopy; subjects enrolled for the group under investigation were diagnosed with AD. The latter included subjects with a documented history of AD with no symptoms for at least one year prior to enrollment, as well as subjects with active AD having a SCORAD ≤ 30 [29]. The first subject was screened in July 2006. The last screening took place in January 2009, the last regular study visit in April 2009 and the last follow up visit in October 2009.

The protocol was approved by independent ethics committees for each site and all volunteers provided written informed consent prior to participation in the clinical trial. Approval was granted by the following committees: Comité de Investigación. para Estudios en Humanos, Puente de Piedra No.150, Col. Toriello Guerra, Tlalpan Mexico City, Mexico 14050; Comité de Ética del Instituto Dermatológico de Jalisco, Avenida Federalismo Norte 3120 Col. Atemajac del Valle Zapopan, Jalisco, Mexico 45190; Comité de Ética de la Unidad de Investigación en Salud de Chihuahua, Ortiz Mena 2200, Fracc. Las Palmas, Chihuahua, Chih. Chihuahua 31205; Comité de Investigación y Bioética del Hospital Adolfo López Mateos, Av. Universidad No. 1321, Col. Florida Mexico City, Mexico City 01030; Comité de Investigación y Ética de la Facultad de Medicina y Hospital Universitario "Dr. José Eleuterio González" Universidad Autónoma de Nuevo León, Av. Francisco I. Madero Pte s/n y Av.Gonzalitos s/n. Col. Mitras Centro Monterrey, Nuevo León 64460; Comité de Ética e Investigación del Hospital Juárez de México, Av. Instituto Politécnico Nacional #5160, Col. Magdalena de las Salinas, Mexico DF, CP 07760; Comité de Ética del Hospital "Doctor Ángel Leaño", Av. Dr. Ángel Leaño #500 Esq. Av. Juan Gil, Col. Los Robles, Zapopan, Jalisco, CP 45200; University of Kentucky Office of Research Integrity and Institutional Review Board, 315 Kinkead Hall, University of Kentucky; Lexington, KY 40506; IntegReview IRB, 3001 S. Lamar Blvd., Suite 210 Austin, TX 78704; Saint Louis University IRB, 3556 Caroline Street; Caroline Bldg C110, St. Louis, MO 63104; and the Northwestern University IRB, Rubloff Hall, 7th Floor; 750 N. Lake Shore Dr., Chicago, IL 60611.

### Safety assessments

Safety assessments were conducted upon screening and at each of five visits during an 8 week active study period: study visits at weeks 0 (vaccination 1), 1, 4 (vaccination 2), 6 and 8. A follow-up (FU) visit or phone call was performed 28 weeks after the second vaccination. Solicited adverse events (AEs) constituted a set of pre-defined, expected local reactions and systemic symptoms. Solicited AEs were recorded on a diary card for the 8-day period following each vaccination. Unsolicited AEs were all events reported by the subject within a 29-day period after each vaccination but not already solicited in the diary card. Any cardiac symptom or electrocardiogram (ECG) change which was determined to be clinically significant and any cardiac enzyme elevation above normal were defined as AEs of special interest (AESI); cardiac enzymes were measured routinely two weeks after each vaccination. ECG assessments were performed at screening, week 1 and week 6. Any AEs that prevented daily activities or body temperatures ≥ 39°C were defined as Grade ≥ 3.

### Immunogenicity assays

Blood was drawn at baseline prior to the first vaccination, 1 week and 4 weeks after the first, as well as 2 weeks and 4 weeks after the second vaccination. A sub-group of subjects had a follow-up sample drawn at least 26 weeks after their first vaccination.

Assays for ELISA and plaque reduction neutralization test (PRNT) are described in detail by Overton et al [30]. For the PRNT a different the detection limit (15) than described by Overton et al [30] was used.

### Statistical methods

The safety analysis was based on the full analysis set (FAS) consisting of all subjects who received at least one vaccine dose. The immunogenicity analysis was performed on the per-protocol analysis set (PPS) comprising all subjects without major protocol violations. All protocol violations were carefully reviewed on a case-by-case basis; major violations included missed visits, missed vaccinations, missing ELISA titer data, immunosuppressive conditions or severity of illness that were exclusion criteria in the protocol, other vaccinations within 14 days of study vaccination, errors in vaccine preparation, or participation in other studies.

All statistical analyses were performed using SAS. AEs were summarized using frequency tables and differences between groups were tested using Fisher’s exact test. For the primary endpoint analysis an exact 95% confidence interval for the difference in ELISA seroconversion rates between healthy and AD subjects was calculated. If the lower limit of the interval was above -5%, then non-inferiority was concluded. A similar secondary analysis was conducted on the PRNT seroconversion rate differences. Differences in GMT were also tested using a homoscedastic t-test based on the log_10_ titers. If the lower limit of the confidence interval was above -0.176 (corresponding to a factor 1.5) for ELISA, or -0.301 (corresponding to a factor 2) for PRNT, then non-inferiority of the AD subjects to the healthy subjects was concluded.

The sample size calculation was based on the primary immunogenicity endpoint MVA-specific seroconversion rate. Assuming a significance level of 5%, a power of >80%, and an expected seroconversion rates of 99% in both groups, a sample size of 124 per group (248 in total) was estimated. In order to account for about 5% drop out rate, a total of 130 subjects per group (260 in total) were planned to be treated. However, in order to meet the safety objective of the trial, i.e. to detect adverse events with an incidence of at least 1:100 with 95% probability, a total of 350 subjects were recruited in the AD group. In order to then best compare the safety profile of the AD group to the healthy group, it was intended to recruit a roughly equal amount of healthy subjects; however, it was also planned that the recruitment would stop once 350 subjects had been recruited into the AD group.

## Results

### Study population

**Fig 1:**
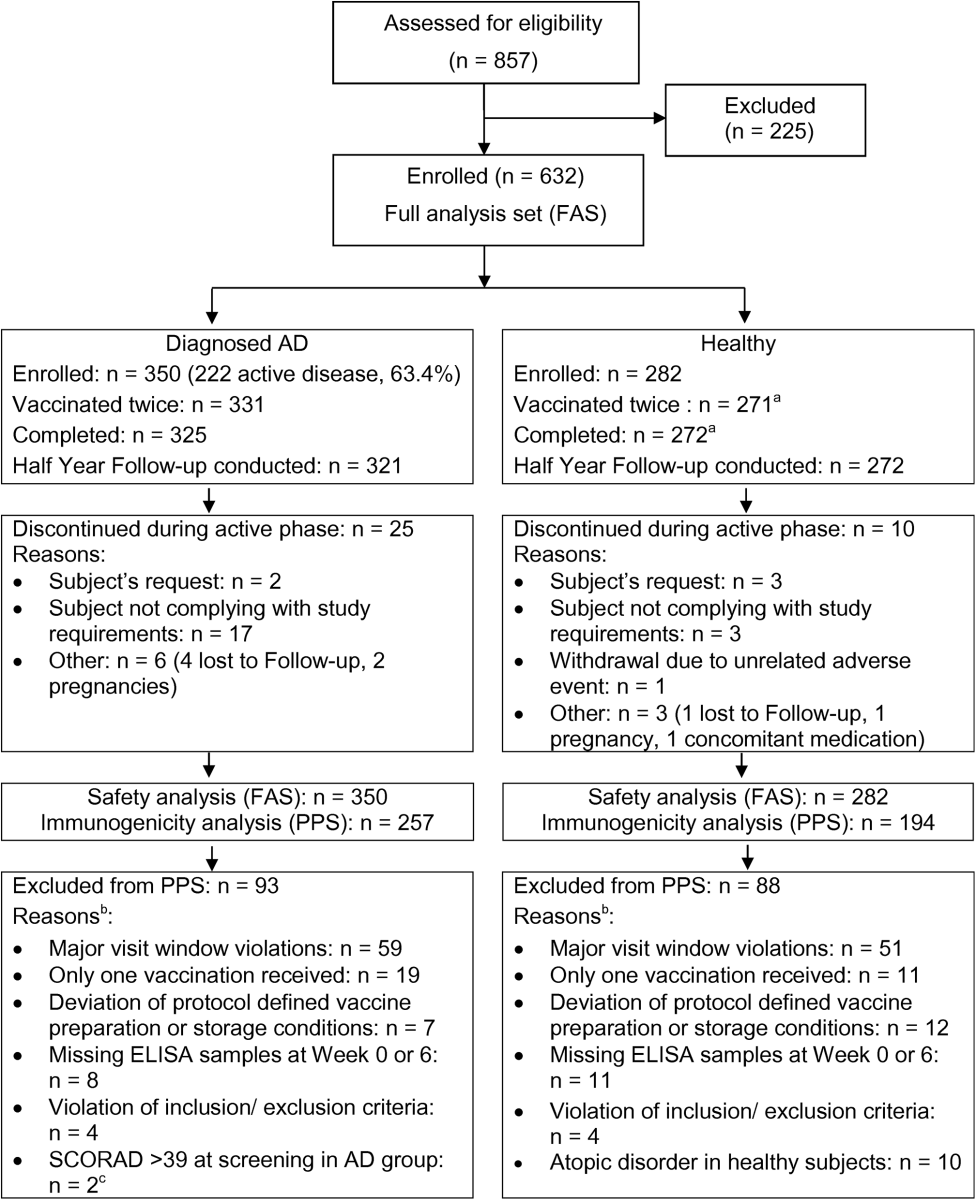
Disposition of subjects. AD = atopic dermatitis; n = number of subjects in the specified category; FAS = full analysis set; PPS = per-protocol analysis set. ^a^: There was one subject that did not receive the second vaccination but did not terminate the study prematurely, i.e. returned for the final visit. ^b^: more than one reason per subject possible. ^c^: 10 subjects with currently active AD had a SCORAD >30 at screening; 2 of these subjects had a SCORAD >39 and were excluded from PPS as major protocol violations. For the volunteers that were excluded from the PPS but had samples available, immunogenicity results were similar to those of the PPS subjects.

Three hundred and fifty AD subjects and 282 healthy subjects who had not been previously vaccinated against smallpox were enrolled (Table 1). 222 subjects in the AD group (63.4%) had active disease at the time of screening (Table 2). All 632 subjects received at least one dose of the MVA smallpox vaccine. Fig 1 displays the disposition of subjects. Follow-up data were collected by a telephone contact 28 weeks after the second vaccination for 321 AD and 272 healthy subjects. For a subset of 76 AD and 94 healthy subjects there was an on-site visit to collect serum for immunogenicity studies.

**Table 1:** Demographic data.

(FAS, N = 632)		Diagnosed AD (N = 350)	Healthy (N = 282)	p-Value
Age [years]	Mean ± SD	27.9 ± 6.33	27.4 ± 5.81	0.3095
	Median	27.0	26.6	
	Range	18–42	18–41	
Gender [n (%)]	Female	223 (63.7)	150 (53.2)	0.0092
	Male	127 (36.3)	132 (46.8)	
Height [cm]	Mean ± SD	166.9 ± 10.06	169.6 ± 9.72	0.0005
	Median	166.0	169.0	
	Range	137–201	145–195	
Weight [kg]^a^	Mean ± SD	73.19 ± 17.950	77.49 ± 19.779	0.0044
	Median	70.30	74.80	
	Range	40.9–138.8	40.9–175.0	
Body mass index [kg/m^2^]^a^	Mean ± SD	26.20 ± 5.850	26.82 ± 6.132	0.1988
	Median	25.00	25.35	
	Range	15.9–55.3	15.7–64.3	
Ethnic origin [n (%)]	Black	33 (9.4)	24 (8.5)	0.0036
	Caucasian	125 (35.7)	124 (44.0)	
	Hispanic	134 (38.3)	109 (38.7)	
	Oriental/ Asian	49 (14.0)	20 (7.1)	
	Other	9 (2.6)	5 (1.8)	
**(PPS, N = 451)**		Diagnosed AD (N = 257)	Healthy (N = 194)	p-Value
Age [years]	Mean ± SD	27.6 ± 6.20	27.2 ± 5.96	0.4618
	Median	26.5	26.2	
	Range	18–42	18–41	
Gender [n (%)]	Female	165 (64.2)	101 (52.1)	0.0119
	Male	92 (35.8)	93 (47.4)	
Height [cm]	Mean ± SD	167.3 ± 10.18	170.3 ± 10.12	0.0023
	Median	166.0	170.0	
	Range	138–201	145–195	
Weight [kg]^a^	Mean ± SD	74.25 ± 18.24	76.92 ± 18.792	0.1370
	Median	72.00	74.80	
	Range	40.9–138.8	43.7–175.0	
Body mass index [kg/m^2^]^a^	Mean ± SD	26.43 ± 5.913	26.44 ± 5.854	0.9960
	Median	25.30	25.15	
	Range	15.9–55.3	15.7–64.3	
Ethnic origin [n (%)]	Black	26(10.1)	15 (7.7)	0.0010
	Caucasian	97 (37.7)	79 (40.7)	
	Hispanic	86 (33.5)	85 (43.8)	
	Oriental/ Asian	40 (15.6)	12 (6.2)	
	Other	8 (3.1)	3 (1.5)	

N = number of subjects in the specified group; n = number of subjects in the specified category; % = percentages of n based on N; AD = atopic dermatitis; FAS = full analysis set; SD = standard deviation

^a^: weight determined at screening visit. PPS = Per Protocol analysis set.

**Table 2:** Atopic dermatitis status.

		Screening N = 350	Week 8 N = 325	P-value
AD currently active	n (%)	222 (63.4)	139 (42.8)	< 0.001
SCORAD (subjects with SCORAD >0 only)	n	220 (62.9)	136 (41.2)	< 0.001
	Mean ± SD	18.6 ± 8.29	15.7 ± 8.61	< 0.001
	Median	19.0	15.0	
	Range	0-70^a^	0-45^a^	

N = number of subjects in the specified group; n = number of subjects in the specified category; % = percentages of n based on N; AD = atopic dermatitis; SD = standard deviation; SCORAD = Scoring Atopic Dermatitis; ^a^: 10 subjects with currently active AD had a SCORAD >30 at screening. The SCORAD is a rather subjective assessment tool. A baseline SCORAD up to 39 was considered as a minor and a baseline SCORAD > 39 as a major protocol violation. This allows for a variability of 30% in SCORAD assessment. 2 of these subjects had a SCORAD >39 and were excluded from PPS as major protocol violations. P-value is Fisher’s exact test of the differences in proportions between groups or a t-test of difference in the mean SCORAD values.

### Subject demographics and SCORAD status

Mean age, height and weight were similar in both groups. Some differences between the groups were observed in ethnic origin and gender distribution (Table 1). The mean SCORAD value of subjects with a SCORAD >0 was 18.6 ± 8.29 at screening, i.e. mild to moderate disease (Table 2). At Week 8 the number of subjects with a SCORAD > 0 was lower and their mean SCORAD was 15.7 ± 8.61, suggesting that MVA did not exacerbate the underlying disease.

### Safety and reactogenicity

#### Overview of adverse events (AEs)

An overview of the incidence of all AEs is presented in Table 3. No case of death, EV or myopericarditis occurred. There was no statistically significant difference in the frequency of AESI in both groups. The trial had been designed to detect, with a 95% probability, any safety signal occurring with a frequency of at least 1%. During the active study period, three subjects experienced a total of four serious AEs. Only one event, extraocular muscle paresis in a healthy subject, was considered as probably related to the study vaccine. One healthy subject was withdrawn from the study due to several AEs (arthralgia, nausea, abdominal pain) considered unlikely related to the study vaccine. AD subjects experienced causally related AEs more frequently than healthy subjects (67.4% vs 59.6%; p = 0.046, Table 3). However, this difference was mostly due to events of mild and moderate intensity. There was no statistically significant difference between the two groups in the proportion of subjects with Grade ≥ 3 causally related AEs (7.7% vs 5.7%; p = 0.343; AD vs healthy).

**Table 3:** Subjects Experiencing at Least One Unsolicited or Solicited Adverse Events (FAS, N = 632).

Subject based	Diagnosed AD (N = 350) n (%)	Healthy (N = 282) n (%)	P-value
At least one AE documented	331 (94.6)	268 (95.0)	0.858
SAE in active study period	1 (0.3)	2 (0.7)	0.589
AESI	58 (16.6)	38 (13.5)	0.316
AE leading to withdrawal from study	0 (0.0)	1 (0.4)	0.446
Causally related AE	236 (67.4)	168 (59.6)	0.046
Causally related AE graded ≥ 3	27 (7.7)	16 (5.7)	0.343

N = number of subjects in the specified group; n = number of subjects in the specified category (with at least one adverse event); % = percentages of n based on N; AD = atopic dermatitis; AE = adverse event; AESI = adverse event of special interest; SAE = serious adverse event; related AE = AE considered by the investigator to have a possible, probable, definite or missing relationship to study medication; Grade ≥ 3 = AE which prevented normal everyday activities or body temperature ≥ 39°C; FAS = full analysis set. P-value is Fisher’s exact test of the differences in proportions between groups.

#### Solicited adverse events

As expected, most subjects in both groups experienced at least one solicited local AE (injection site pain, injection site erythema or injection site swelling, Table 4). Local AEs were most often of Grade 1 intensity except for injection site pain, which was experienced by most individuals with Grade 2 or 3 intensity. Statistically significant differences between the groups were seen for injection site erythema (p = 0.004) and injection site swelling (p = 0.005). Solicited systemic AEs (Table 5) were more frequent in subjects diagnosed with AD (70.1%) compared to healthy subjects (56.4%), a statistically significant difference (p < 0.001). The majority of solicited systemic AEs were considered to be causally related to the study vaccine. The duration of both solicited systemic and local AEs was similar in both groups. Taken together, the data indicate that AD subjects have slightly more solicited AEs with more intense reactions towards MVA than healthy subjects.

**Table 4:** Subjects Experiencing at Least One Solicited Local Adverse Event (8-Day Follow-Up Period after Vaccination, FAS, N = 632).

Solicited Local Event	Intensity/Size	Diagnosed AD N = 345 n (%)	Healthy N = 282 n (%)	P-value
Injection Site Pain	Total	283 (82.0)	233 (82.6)	0.916
	Grade ≥ 2^a^	152 (44.1)	117 (41.5)	0.570
	Grade ≥ 3^b^	53 (15.4)	31 (11.0)	0.126
Injection Site Erythema	Total	211 (61.2)	139 (49.3)	0.004
	≥ 30 mm (Grade 2)	66 (19.1)	35 (12.4)	0.029
	≥ 100 mm (Grade 3)	3 (0.9)	3 (1.1)	1.000
Injection Site Swelling	Total	180 (52.2)	115 (40.8)	0.005
	≥ 30 mm (Grade 2)	45 (13.0)	21 (7.4)	0.026
	≥ 100 mm (Grade 3)	1 (0.3)	2 (0.7)	0.591

N = number of subjects in the specified group; n = number of subjects in the specified category (with at least one report of a local adverse event); % = percentages of n based on N; AD = atopic dermatitis; AE = adverse event. FAS = full analysis set

^a^: Injection Site Pain grade 2: pain when moving the limb

^b^: Injection Site Pain grade 3: spontaneously painful. P-value is Fisher’s exact test of the differences in proportions between groups.

**Table 5:** Subjects Experiencing at Least One Solicited Systemic Adverse Events (8-Day Follow-Up period After Vaccination, FAS, N = 632).

Solicited Systemic Adverse Event	Intensity	Diagnosed AD N = 345 n (%)	Healthy N = 282 n (%)	P-value
Any Solicited AE		242 (70.1)	159 (56.4)	0.001
Body temperature increase	Total number	28 (8.1)	23 (8.2)	1.000
	≥ 39.0°C	1 (0.3)	1 (0.4)	1.000
	Grade ≥ 3 (related)	1 (0.3)	1 (0.4)	1.000
Headache	Total number	163 (47.2)	98 (34.8)	0.002
	Grade ≥ 3	26 (7.5)	9 (3.2)	0.022
	Grade ≥ 3 (related)	18 (5.2)	5 (1.8)	0.031
Myalgia	Total number	153 (44.3)	98 (34.8)	0.018
	Grade ≥ 3	14 (4.1)	9 (3.2)	0.671
	Grade ≥ 3 (related)	9 (2.6)	8 (2.8)	1.000
Chills	Total number	55 (15.9)	22 (7.8)	0.002
	Grade ≥ 3	7 (2.0)	4 (1.4)	0.762
	Grade ≥ 3 (related)	3 (0.9)	4 (1.4)	0.707
Nausea	Total number	80 (23.2)	41 (14.5)	0.008
	Grade ≥ 3	8 (2.3)	6 (2.1)	1.000
	Grade ≥ 3 (related)	5 (1.4)	4 (1.4)	1.000
Fatigue	Total number	124 (35.9)	75 (26.6)	0.013
	Grade ≥ 3	16 (4.6)	9 (3.2)	0.416
	Grade ≥ 3 (related)	9 (2.6)	5 (1.8)	0.592

N = number of subjects in the specified group; n = number of subjects in the specified category (with at least one report of a solicited systemic AE); % = percentages of n based on N; AD = atopic dermatitis; AE = adverse event; Grade ≥ 3 = AE which prevented normal everyday activities or body temperature ≥ 39°C; related AE = AE considered by the investigator to have a possible, probable or definite relationship to study medication.

#### Unsolicited adverse events

Unsolicited AEs experienced by ≥ 2% of subjects in any study group are presented in Table 6. A similar percentage of subjects reported at least one unsolicited AE (68.6% in subjects with diagnosed AD and 64.5% in healthy subjects), with most events being classified as 'systemic disorders and administration site conditions'. This was mostly due to a high rate of 'injection site pruritus', which was reported by 28.6% of the subjects in the AD group and by 17.0% of the healthy subjects, a statistically significant difference (p < 0.001). Rashes were observed only in isolated cases, with no trend towards a specific treatment group. In addition, AEs classified as 'skin and subcutaneous tissue disorders' were reported more frequently by AD than by healthy subjects (p = 0.002). Overall, more than two thirds of all unsolicited AEs in both groups were of Grade 1 intensity and Grade 3 AEs were reported in both groups with a similar frequency. Unsolicited AEs assessed as being probably or definitely related to the study vaccine occurred in 31.8% of the AD and 22.5% of the healthy subjects (p = 0.007) and they were usually associated with direct injection site reactions. Overall AD subjects had more skin reactions possibly, probable or definite related to the vaccine than healthy subjects, but these reactions were mostly mild or moderate and did not prevent daily activities.

**Table 6:** Subjects Experiencing at Least One Unsolicited Adverse Events (Occurring in ≥ 2% of Subjects in any Study Group, FAS, N = 632).

System organ class Preferred term (MedDRA 9.0)	Diagnosed AD (N = 350) n (%)	Healthy (N = 282) n (%)	p-Value
At least one unsolicited AE	240 (68.6)	182 (64.5)	0.3083
Unsolicited AE Grade 1	298 (68.5)	432 (80.1)	0.1108
Unsolicited AE Grade 3	29 (6.7)	21 (3.9)	0.7627
Systemic disorders and administration site conditions	119 (34.0)	65 (23.0)	0.0027
Injection site pruritus	100 (28.6)	48 (17.0)	0.0007
Injection site bruising	7 (2.0)	2 (0.7)	0.3114
Injection site induration	3 (0.9)	6 (2.1)	0.1977
Investigations	63 (18.0)	46 (16.3)	0.5979
Troponin I increased	54 (15.4)	37 (13.1)	0.4273
Infections and infestations	56 (16.0)	55 (19.5)	0.2930
Nasopharyngitis	23 (6.6)	11 (3.9)	0.1582
Influenza	11 (3.1)	8 (2.8)	1.0000
Upper respiratory tract infection	6 (1.7)	9 (3.2)	0.2946
Pharyngitis	4 (1.1)	9 (3.2)	0.0916
Pharyngotonsillitis	3 (0.9)	7 (2.5)	0.1196
Gastrointestinal disorders	30 (8.6)	29 (10.3)	0.4933
Diarrhoea	9 (2.6)	7 (2.5)	1.0000
Nervous system disorders	29 (8.3)	27 (9.6)	0.5766
Headache	16 (4.6)	19 (6.7)	0.2940
Dizziness	11 (3.1)	7 (2.5)	0.8107
Skin and subcutaneous tissue disorders	35 (10.0)	10 (3.5)	0.0017
Dermatitis atopica	15 (4.3)	0 (0.0)	0.0002
Respiratory, thoracic and mediastinal disorders	16 (4.6)	25 (8.9)	0.0345
Cough	3 (0.9)	8 (2.8)	0.0705
Pharyngolaryngeal pain	2 (0.6)	8 (2.8)	0.0275
Rhinorrhoea	3 (0.9)	6 (2.1)	0.1977
Musculoskeletal and connective tissue disorders	13 (3.7)	19 (6.7)	0.1008
Injury, poisoning and procedural procedures	9 (2.6)	15 (5.3)	0.0932
Reproductive system and breast disorders	9 (2.6)	11 (3.9)	0.3683
Dysmenorrhoea	8 (2.3)	7 (2.5)	1.0000
Psychiatric disorders	7 (2.0)	9 (3.2)	0.4464
Eye disorders	7 (2.0)	3 (1.1)	0.5243

N = number of subjects in the specified group; n = number of subjects in the specified category (with at least one report of a unsolicited AE); % = percentages of n based on N; AD = atopic dermatitis; AE = adverse event; MedDRA = Medical Dictionary for Regulatory Activities; FAS = full analysis set.

#### Cardiac events

To determine the cardiac risk, any cardiac symptom, ECG change determined to be clinically significant, cardiac enzyme or troponin I level elevated above normal, was noted as an AESI. When AESI persisted, or if clinically indicated, the subjects were referred to a cardiologist. A transient increase in troponin I was measured for about the same percentage of subjects diagnosed with AD (15.4%) as for healthy subjects (13.1%, Table 6).

In the AD group, four subjects presented with one AESI each other than an increased Troponin I: Tachycardia and QTc prolongation of >440 msec, both assessed as unlikely related, and T wave inversion and palpitations, both considered possibly related to the study vaccine. The case of palpitations was assessed as benign by a cardiologist and symptoms resolved successfully after treatment with atenolol. A precise cause for the palpitations was not identified; however tests indicated that the subject’s thyroid values were at the upper limit of normal and the ongoing concomitant medication with albuterol may have contributed. A second case of tachycardia, assessed as unrelated to the study vaccine, was recorded for one healthy subject at a follow-up visit. The heart rate had returned to normal at an additional visit. In total, four subjects were referred to a cardiologist, but with the exception of the case mentioned above, no cardiac treatment was prescribed. All events resolved without sequelae.

#### Serious adverse events (SAEs)

During the active study period, one subject with active AD and two healthy subjects experienced a total of four SAEs. Three of them (head injury, loss of consciousness, panic attack) were assessed as not related to the study vaccine. One SAE (extraocular muscle paresis) occurring 8 days after the second vaccination was noted as probably related to the vaccine. The subject was a 28 year old healthy woman who complained of constant mixed horizontal and vertical diplopia. The ophthalmologist treated her with carbamazepine and vitamin B complex and the event resolved without sequelae. After Week 8, one healthy subject and two subjects with active AD experienced a total of four SAEs (pneumonia, asthma attack in a subject with a history of asthma, and two events of psychoneurotic disorder in one subject); all were assessed as not related to the study vaccine.

### Immunogenicity

**Fig 2:**
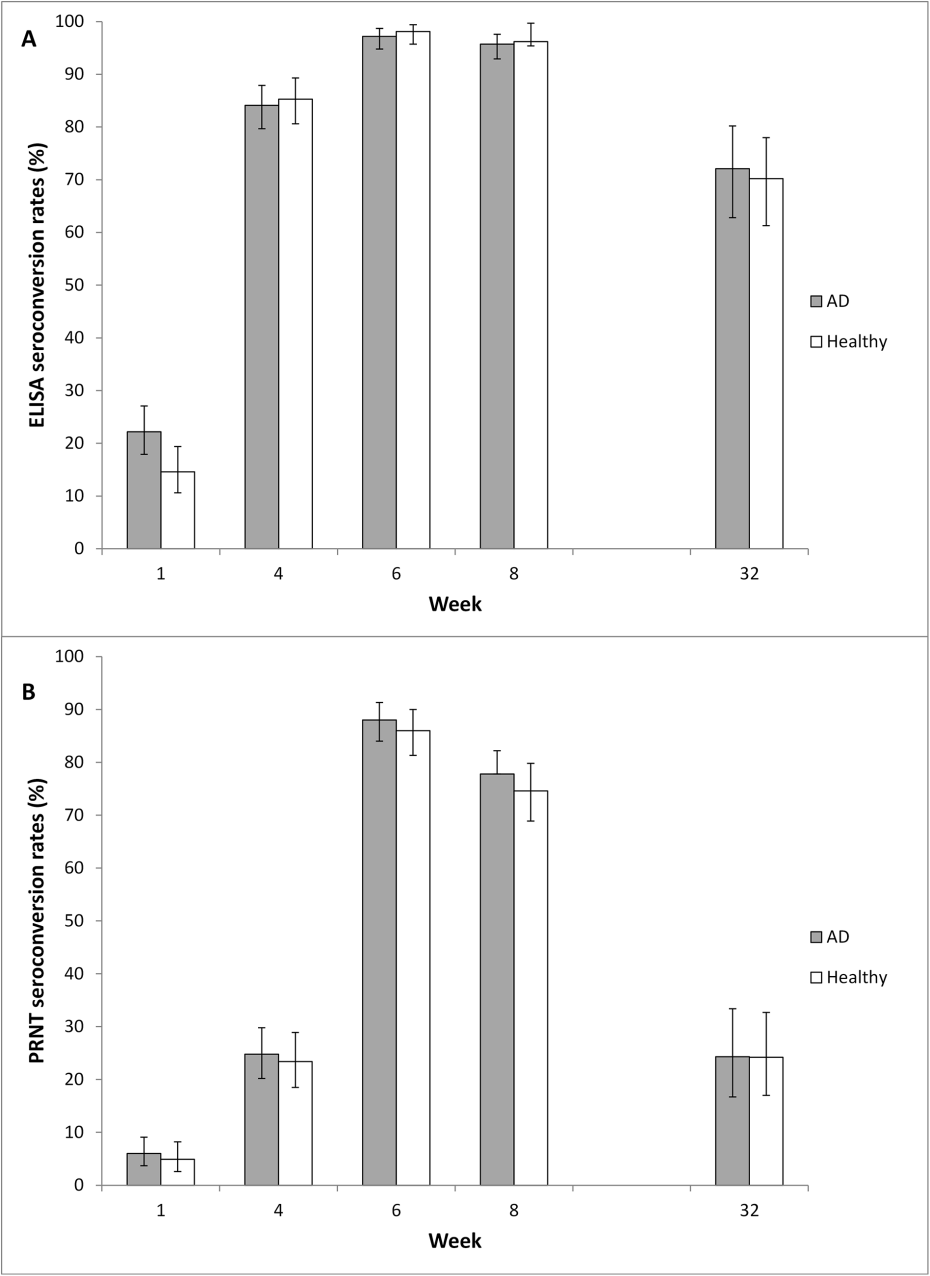
Seroconversion. Seroconversion rates were determined by vaccinia-specific ELISA (A) and PRNT (B). Data set is FAS (full analysis set), N = 632 (for Week 32 N = 235). Error bars represent upper and lower confidence intervals. Vaccinations were given at Week 0 and Week 4. AD = atopic dermatitis.

ELISA and PRNT seroconversion rates were similar for both groups at all time points (Fig 2A and 2B, Table 7). ELISA seroconversion rates peaked two weeks post second vaccination when 97.2% of AD subjects and 98.1% of healthy subjects had seroconverted. The primary endpoint of the trial was met because the ELISA seroconversion rate for AD subjects two weeks post second vaccination was non-inferior compared to that for healthy subjects. PRNT seroconversion rates peaked two weeks post second vaccination with 88.0% of the AD group and 86.0% of the healthy group seroconverting. The PRNT seroconversion rate for AD subjects two weeks post second vaccination was also non-inferior compared to that for healthy subjects.

**Table 7:** Seroconversion rates for ELISA and PRNT at all post-baseline visits (FAS, N = 632).

Week	Diagnosed AD (N = 350) n SC (%)	Healthy (N = 282) n SC (%)	Difference in SC rates (95% CI) (AD–Healthy)
**ELISA**			
Week 1	333 74 (22.2)	267 39 (14.6)	7.6 (1.4, 13.8)
Week 4	328 276 (84.1)	273 233 (85.3)	-1.2 (-7.0,4.7)
Week 6	325 316 (97.2)	265 260 (98.1)	-0.9 (-3.7,1.9)
Week 8	325 311 (95.7)	264 254 (96.2)	-0.5 (-3.9, 2.9)
Week 32	111 80 (72.1)	124 87 (70.2)	1.9 (-9.9, 13.7)
**PRNT**			
Week 1	333 20 (6.0)	267 13 (4.9)	1.1 (-2.7, 4.9)
Week 4	327 81 (24.8)	273 64 (23.4)	1.3 (-5.6, 8.2)
Week 6	325 286 (88.0)	265 228 (86.0)	2.0 (-3.5, 7.6)
Week 8	325 253 (77.8)	264 197 (74.6)	3.2 (-3.7,10.2)
Week 32	111 27 (24.3)	124 30 (24.2)	0.1 (-11.0, 11.3)

N = number of subjects in the specified group; n = number of subjects who had a titer available at the specified visit; SC is the number of subjects who had seroconverted.

**Fig 3:**
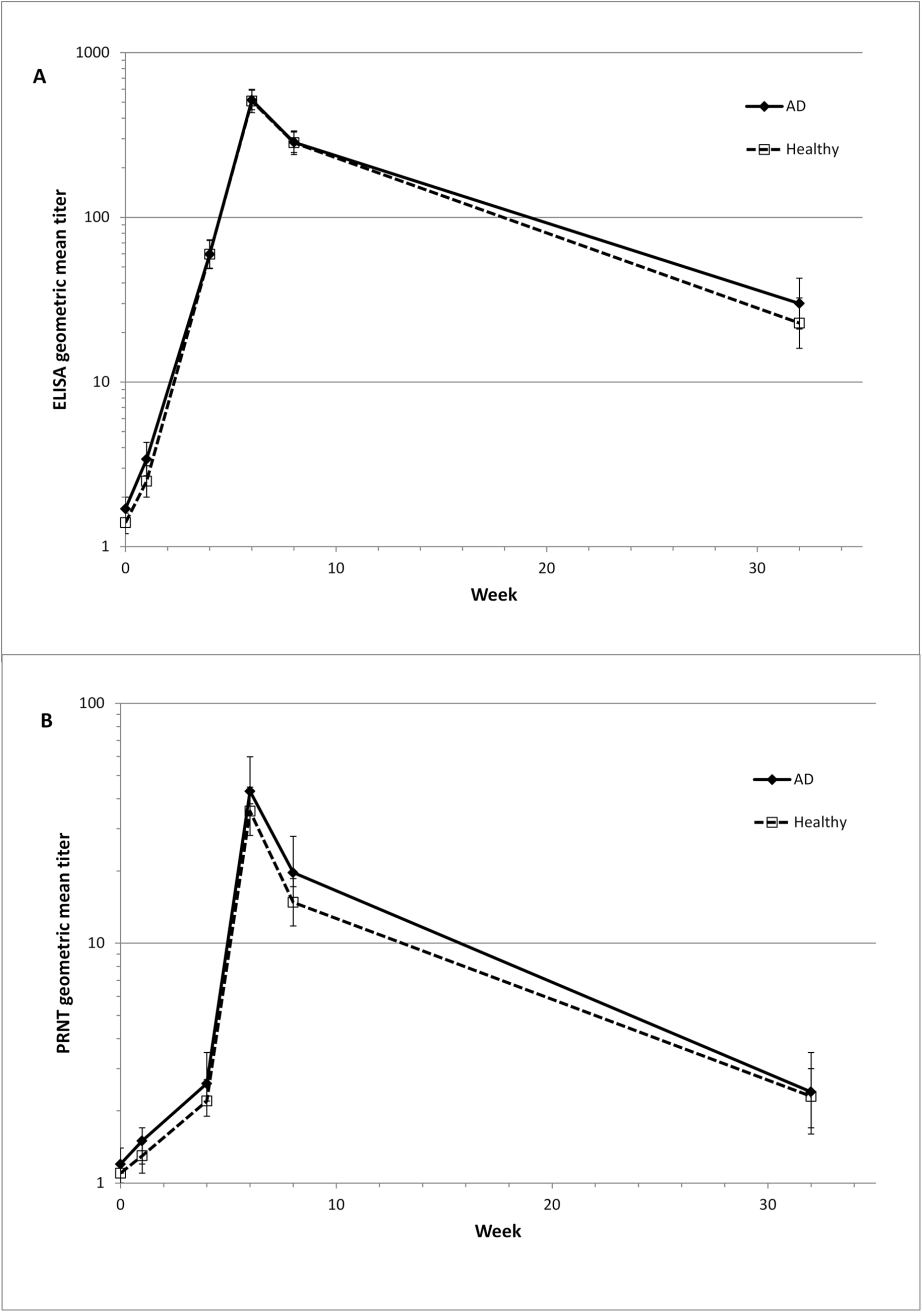
Antibody Titers. Geometric mean titers determined by vaccinia-specific ELISA (A) or PRNT (B). Data set is FAS (full analysis set), N = 632 (for Week 32 N = 235). Error bars represent upper and lower confidence intervals. Vaccinations were given at Week 0 and Week 4. AD = atopic dermatitis.

GMTs determined by ELISA were similar for both groups at all visits, reaching a peak of 516.0 at Week 6 for the AD group, which was non-inferior to the peak of 508.8 for the healthy group (Fig 3A, Table 8) (p<0.0001). The peak GMT determined by PRNT for the AD group was non-inferior compared to that of the healthy group at Week 6 (GMT of 43.0 and 38.5 in the AD and healthy groups respectively, (p<0.0001)). Again, the GMTs on all other visits were similar for both groups. Combining both groups, there was a statistically significant correlation between ELISA and PRNT results (Fig 3B) at all time points, with Spearman correlation coefficients ranging from between 0.40 to 0.65 at the different time points. Maximum correlation was at the peak visit two weeks after the second vaccination.

**Table 8:** GMT for ELISA and PRNT at all post-baseline visits (FAS, N = 632).

Week	Diagnosed AD (N = 350) GMT (95% CI)	Healthy (N = 282) GMT (95% CI)	Ratio of GMTs (95% CI) (AD / Healthy)
**ELISA**			
Week 0	1.7 (1.5, 2.0)	1.4 (1.2, 1.6)	1.211 (0.984, 1.489)
Week 1	3.4 (2.7, 4.3)	2.5 (2.0, 3.1)	1.387 (1.004, 1.916)
Week 4	59.7 (49.2, 72.4)	59.7 (48.8, 73.1)	0.999 (0.755, 1.324)
Week 6	516.0 (449.7, 592.0)	508.8 (432.2, 598.9)	1.014 (0.821, 1.253)
Week 8	285.8 (247.6, 329.8)	283.8 (240.8, 334.6)	1.007 (0.811, 1.251)
Week 32	30.0 (21.0, 42.7)	22.8 (16.0, 32.4)	1.314 (0.798, 2.163)
**PRNT**			
Week 0	1.2 (1.1, 1.3)	1.1 (1.0, 1.2)	1.103 (0.982, 1.238)
Week 1	1.4 (1.2, 1.7)	1.3 (1.1, 1.5)	1.121 (0.924, 1.359)
Week 4	2.6 (2.1, 3.1)	2.2 (1.9, 2.7)	1.444 (0.868, 1.508)
Week 6	43.0 (35.0, 52.8)	35.5 (28.1, 44.7)	1.213 (0.890, 1.653)
Week 8	19.7 (15.9, 24.3)	14.8 (11.8, 18.6)	1.329 (0.971, 1.819)
Week 32	2.4 (1.8, 3.3)	2.3 (1.7, 3.0)	1.074 (0.713, 1.618)

N = number of subjects in the specified group.

## Discussion

This Phase II trial confirmed the observations from a Phase I trial that the safety and immunogenicity of MVA in individuals with active AD or a history of AD is similar to healthy individuals [23]. Unsolicited and solicited AEs reported during the study were consistent with results obtained from previously completed clinical trials with MVA [19–23].

The number of serious AEs was low, just one serious AE involving transitory ocular paralysis was assessed as probably related to the study vaccine. The impairment was limited to the extraocular inferior rectal muscle which recovered without sequelae. To date, no other similar event has been observed following administration of MVA. No other serious AE with a possible, probable or definite causal relationship occurred in the AD group, indicating with a 95% certainty that the rate of such events in subjects with AD is less than 1%.

Not surprisingly, AD subjects were more likely to develop 'injection site erythema' and 'injection site swelling'. However, there was no indication that vaccination with MVA worsened the intensity of AD. Some subjects in the AD group experienced a flare-up or worsening of their disease during the study, while others with active AD at screening did not show any active AD at the end of the study. This may reflect the nature of the underlying disease, which shows episodes of exacerbations as well as periods of remission [31].

While the burden of AD in the adult population is not as well characterized as in pediatric populations, based on SCORAD the population enrolled in this study was similar to the distribution of AD reported in pediatric populations [32,33]. Although individuals with severe AD (SCORAD >30) were per protocol excluded from this trial, there was no signal in this trial to suggest that MVA would not be safe for them. Historically, the severity of AD has not been associated with increased occurrence or greater severity of complications resulting from smallpox vaccination. Both persons with a history of active disease or with active disease are at risk of developing EV, suggesting an underlying “genetic” susceptibility might be the most important factor for vaccination side-effects [6]. As MVA does not replicate in human cells and is administered subcutaneously rather than by scarification, MVA vaccination should not result in any of the other adverse events associated with virus replication. While the trial was not large enough to draw definitive conclusions, it is reassuring that no case of EV or any other replication-associated adverse event occurred.

Smallpox vaccination using replicating vaccines conducted in recent years resulted in cases of myopericarditis that clustered during the second week after vaccination. The occurrence of vaccine associated myopericarditis was confirmed at a rate of 5.73 per 1,000 vaccinia-naïve vaccinees who received ACAM2000 [34,35]. Cardiac monitoring during the present trial revealed no cardiac risks related to the use of MVA, consistent with published data on cardiac monitoring following administration of MVA [36]. The vast majority of cardiac related events were Grade 1 events ('troponin I increased') that were transient and not connected to abnormal ECG findings or any cardiac symptom [37].

The trial confirmed that AD subjects were able to generate humoral vaccinia-specific immune responses at levels similar to healthy subjects, confirming earlier results in subjects with mild AD [31]. Although individuals with severe AD (SCORAD >30) were not under investigation in this the trial, it is likely that they would mount a comparable immunological response, as there is little evidence suggesting that AD has any measurable impact on immunological responses to vaccines delivered subcutaneously [38].

In summary, vaccination with MVA was equally well tolerated in both populations and elicited a robust vaccinia-specific immune response. The observation that vaccination does not have any discernible impact on the status or severity of AD suggests that MVA may represent a safer alternative to replication-competent vaccinia vaccines for individuals with AD.

## Supporting Information

S1 Checklist (DOCX)

S1 Fig This is the Seroconversion ELISA PPS. (TIF)

S2 Fig This is the Seroconversion PRNT PPS. (TIF)

S3 Fig This is the GMT ELISA PPS. (TIF)

S4 Fig This is the GMT PRNT PPS. (TIF)

S5 Fig These are the figure legends for the supplemental figures. (DOCX)

S1 IRB These are the IRB approvals for the sites in Mexico. (DOC)

S2 IRB These are the IRB listings. (PDF)

S3 IRB This is the IRB approval for Dr. Greenberg’s site. (PDF)

S1 Patents (DOCX)

S1 Protocol (PDF)

S1 Table These are adverse events tables. (DOCX)

S2 Table This shows the PRNT and ELISA Titers of Subjects not in PPS. (DOCX)
