# Correction: A Multicenter, Open-Label, Controlled Phase II Study to Evaluate Safety and Immunogenicity of MVA Smallpox Vaccine (IMVAMUNE) in 18-40 Year Old Subjects with Diagnosed Atopic Dermatitis

**DOI:** 10.1371/journal.pone.0142802

**Published:** 2015-11-10

**Authors:** Richard N Greenberg, Maria Yadira Hurley, Dinh V. Dinh, Serena Mraz, Javier Gomez Vera, Dorothea von Bredow, Alfred von Krempelhuber, Siegfried Roesch, Garth Virgin, Nathaly Arndtz-Wiedemann, Thomas Peter Meyer, Darja Schmidt, Richard Nichols, Philip Young, Paul Chaplin

The second author’s name is spelled incorrectly. The correct name is: Maria Yadira Hurley. The correct citation is: Greenberg RN, Hurley MY, Dinh DV, Mraz S, Vera JG, von Bredow D, et al. (2015) A Multicenter, Open-Label, Controlled Phase II Study to Evaluate Safety and Immunogenicity of MVA Smallpox Vaccine (IMVAMUNE) in 18–40 Year Old Subjects with Diagnosed Atopic Dermatitis. PLoS ONE 10(10): e0138348. doi:10.1371/journal.pone.0138348
