# Supplementary figures and images for: A Multicenter, Open-Label, Controlled Phase II Study to Evaluate Safety and Immunogenicity of MVA Smallpox Vaccine (IMVAMUNE) in 18–40 Year Old Subjects with Diagnosed Atopic Dermatitis
Source: PLoS One. 2015 Oct 6;10(10):e0138348. doi: 10.1371/journal.pone.0138348 (PMC4595076; doi:10.1371/journal.pone.0138348)

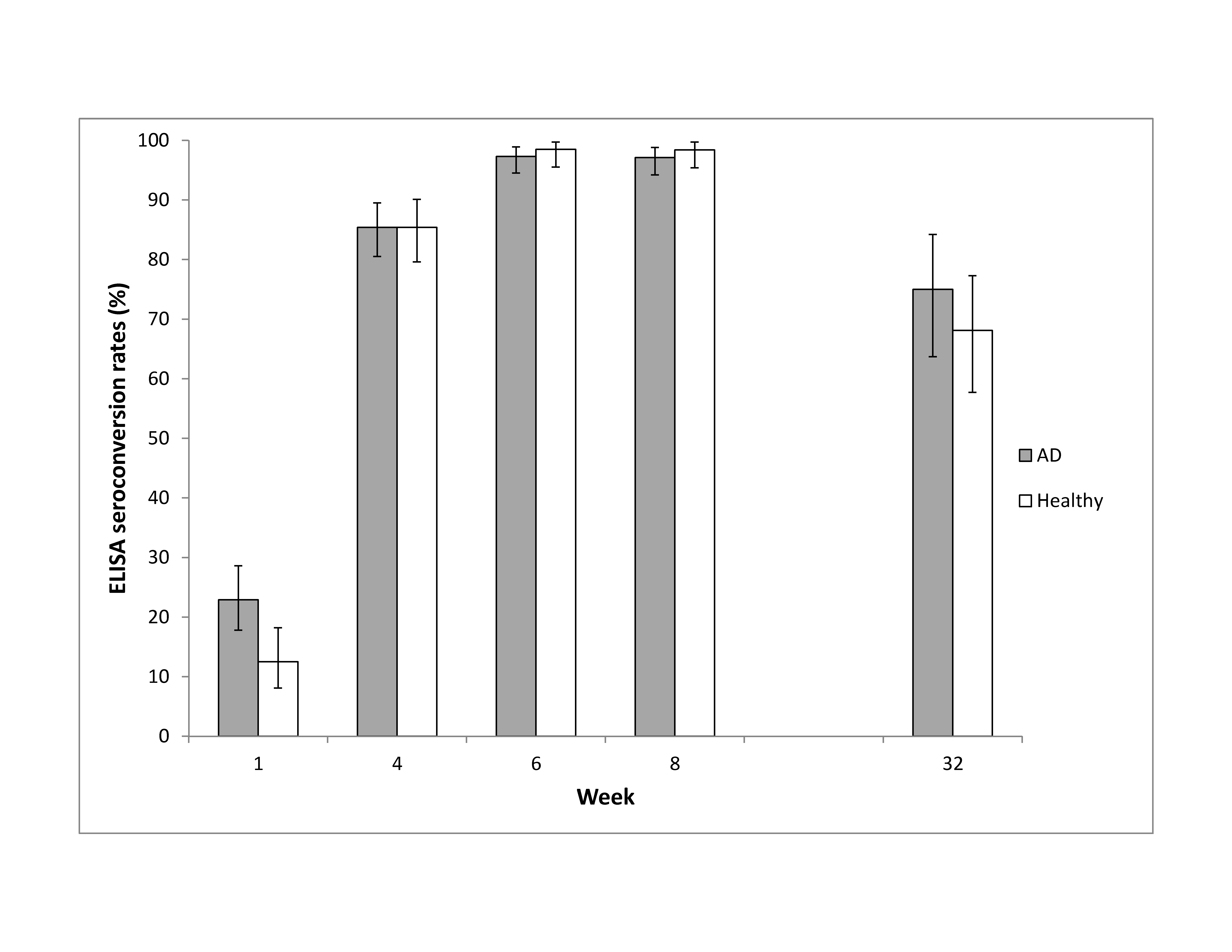

Supplement: S1 Fig — (TIF) [file pone.0138348.s002.tif]

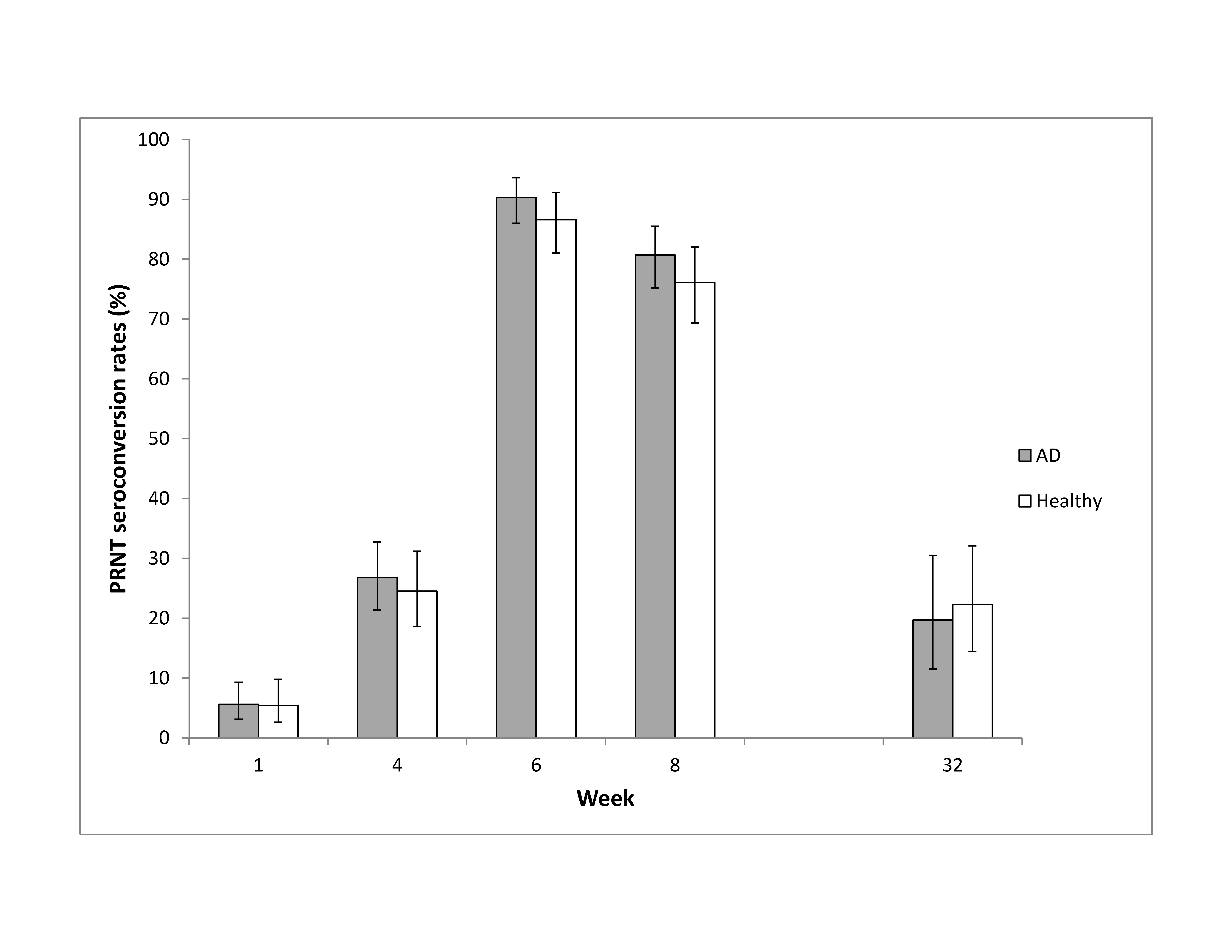

Supplement: S2 Fig — (TIF) [file pone.0138348.s003.tif]

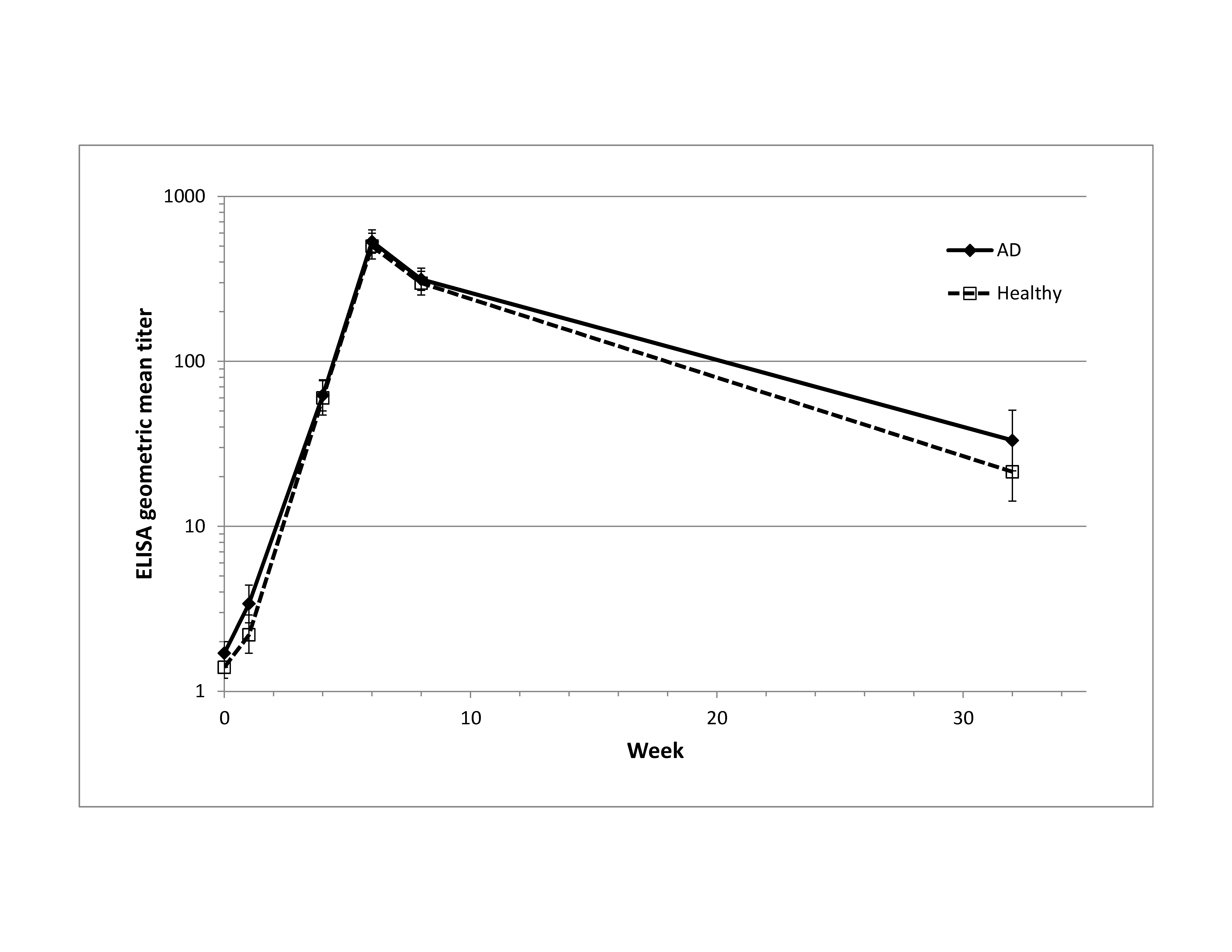

Supplement: S3 Fig — (TIF) [file pone.0138348.s004.tif]

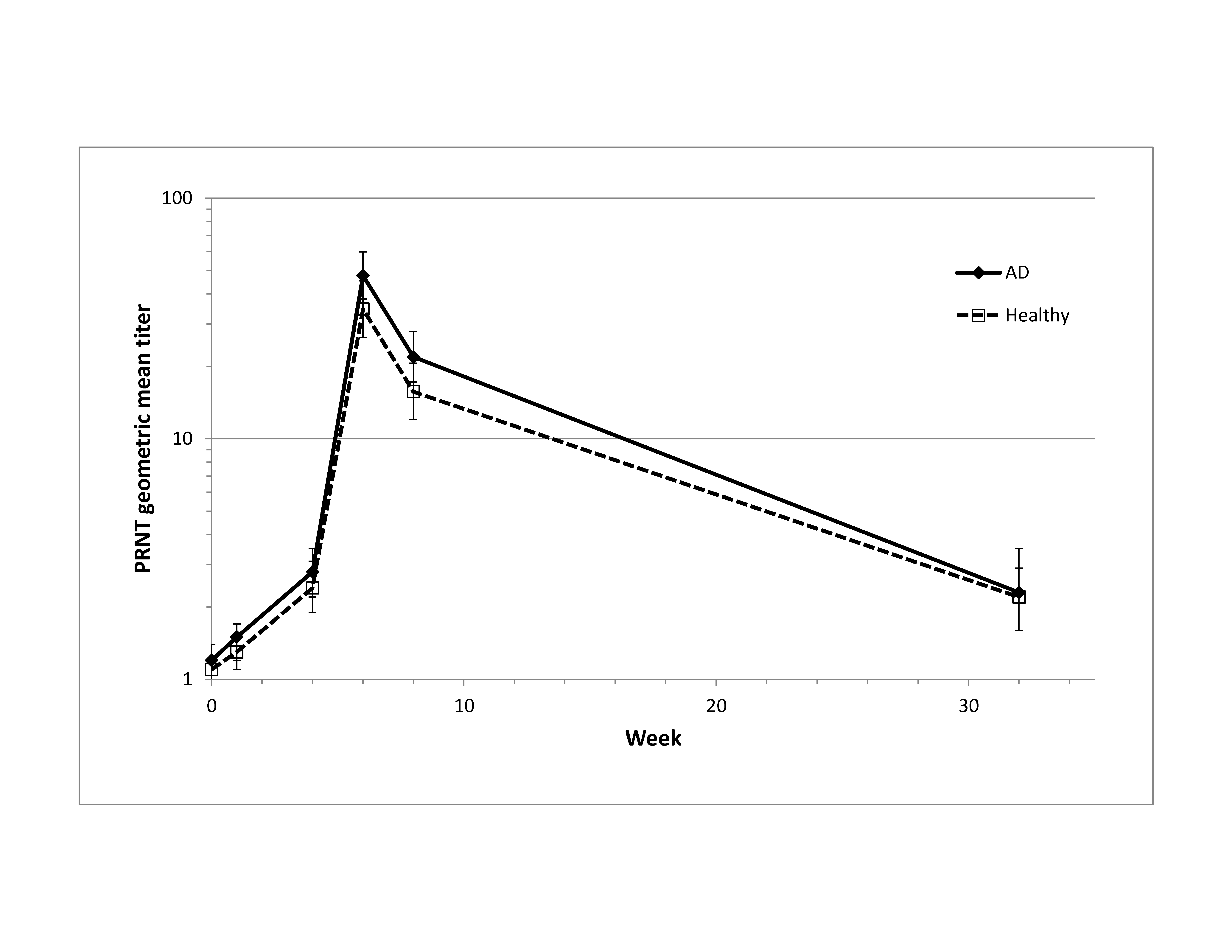

Supplement: S4 Fig — (TIF) [file pone.0138348.s005.tif]

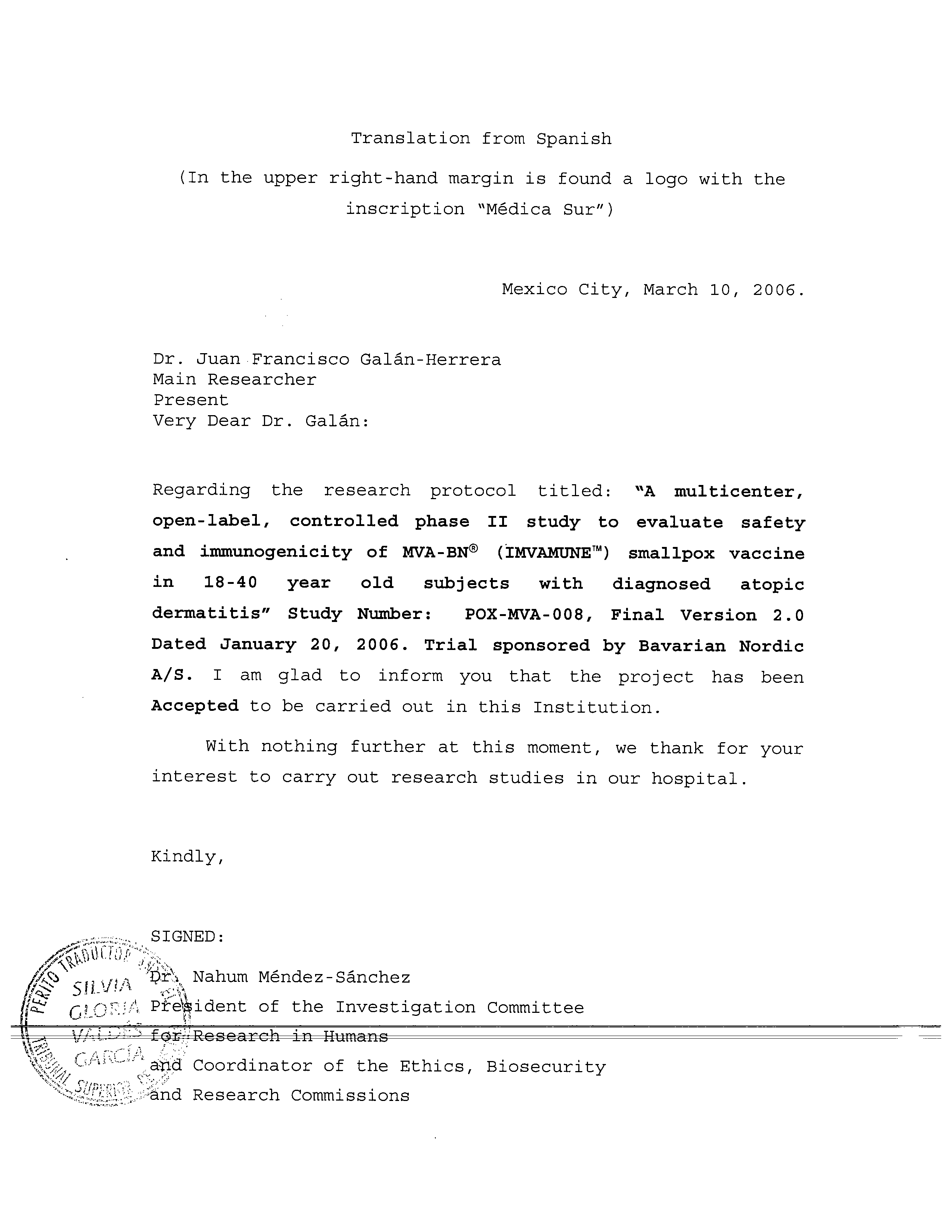

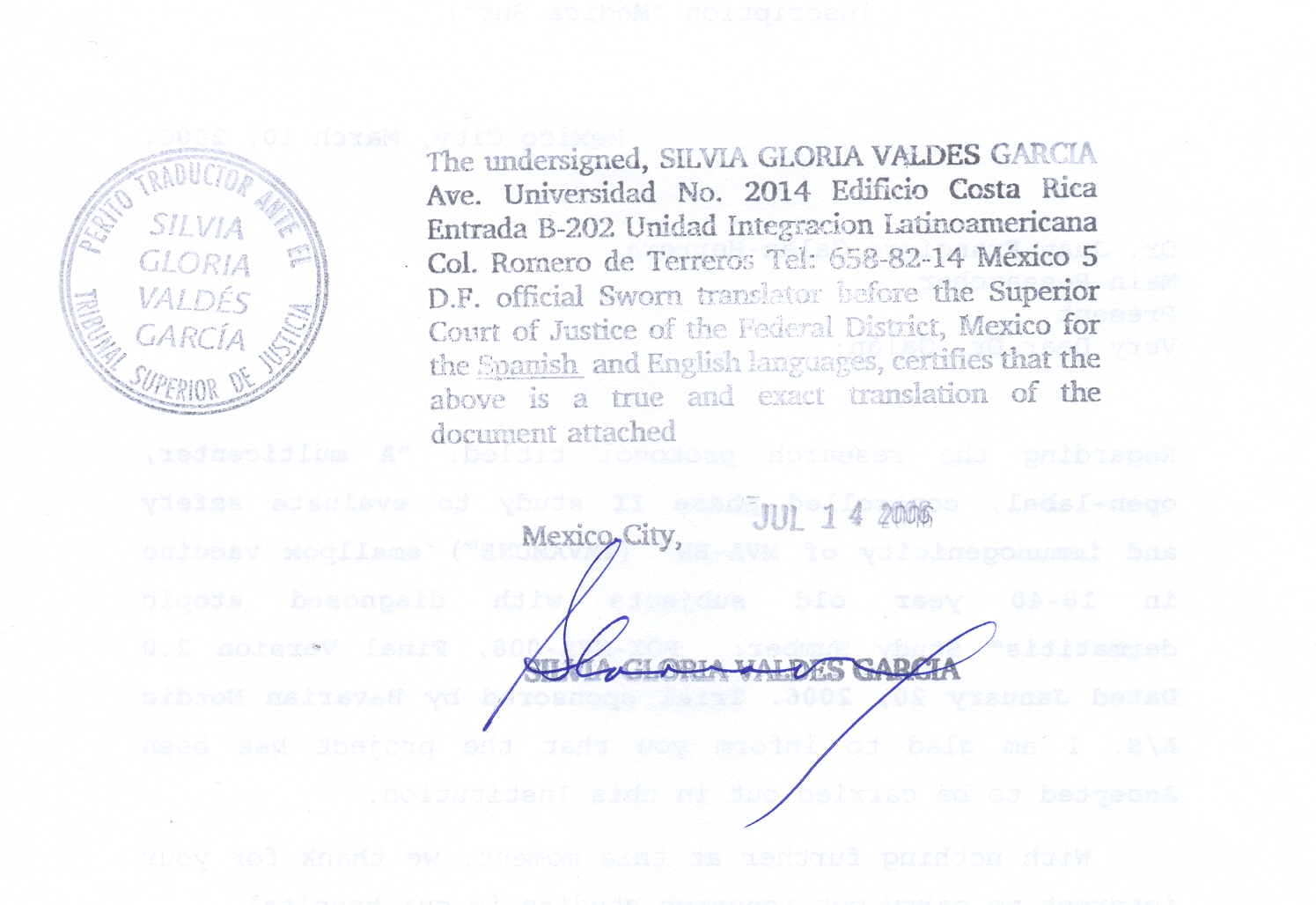


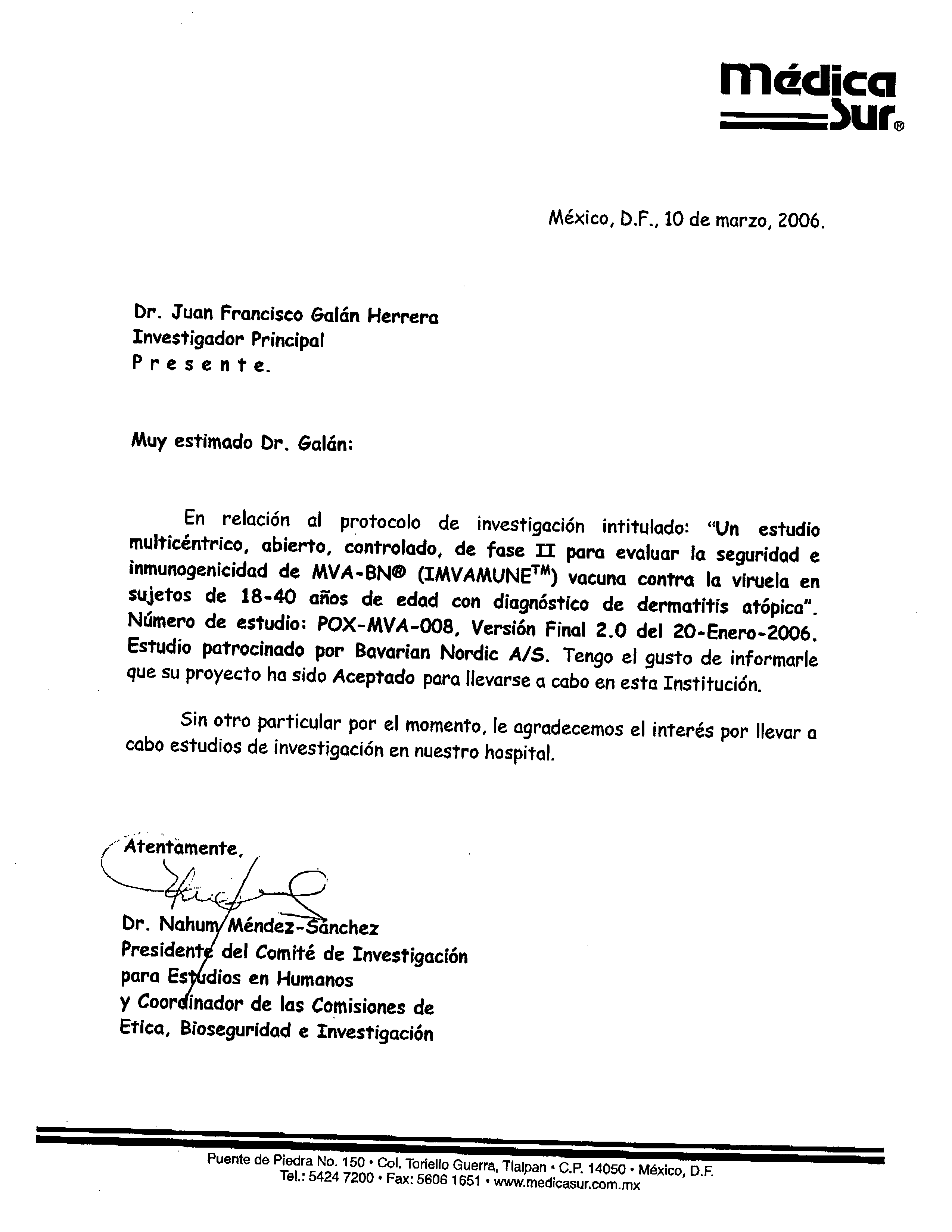


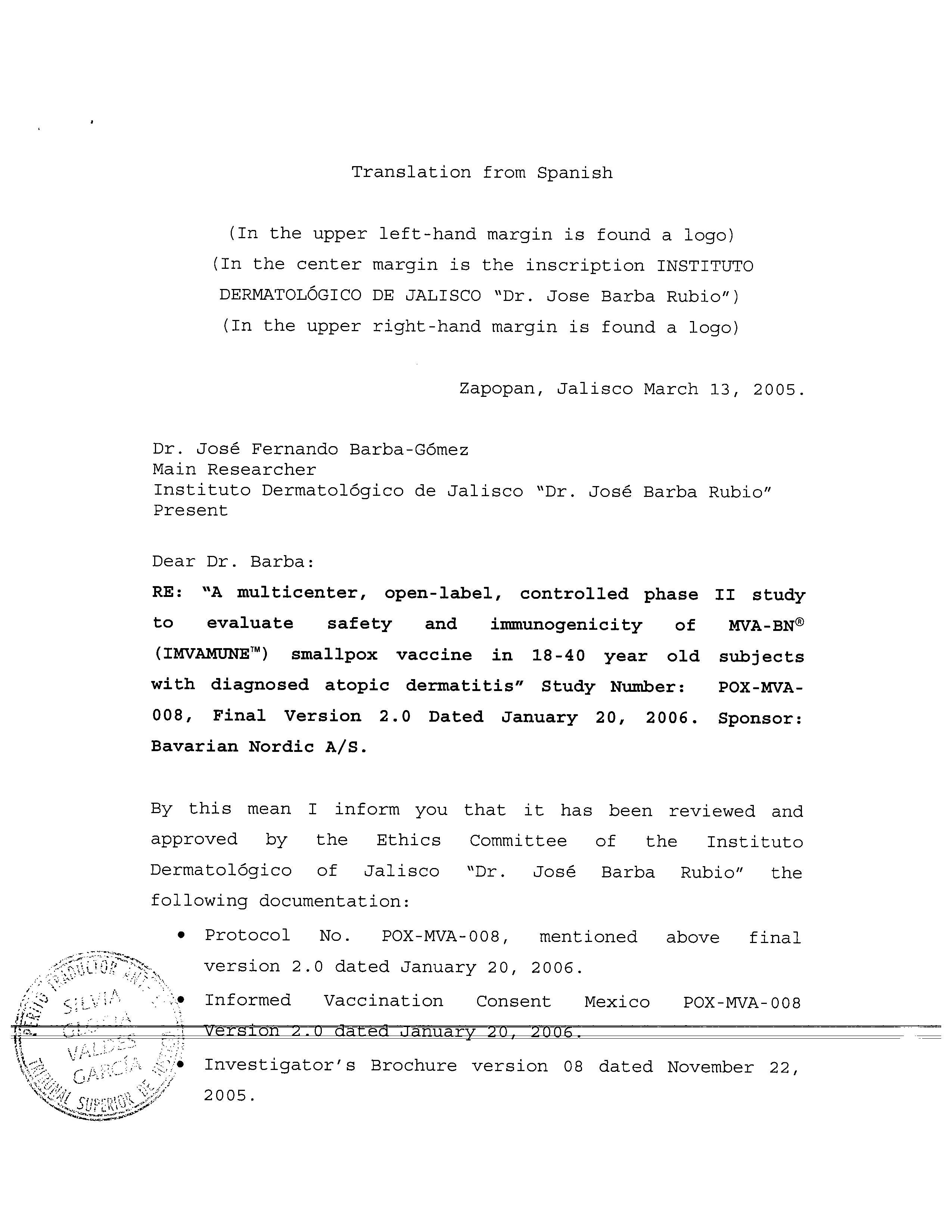

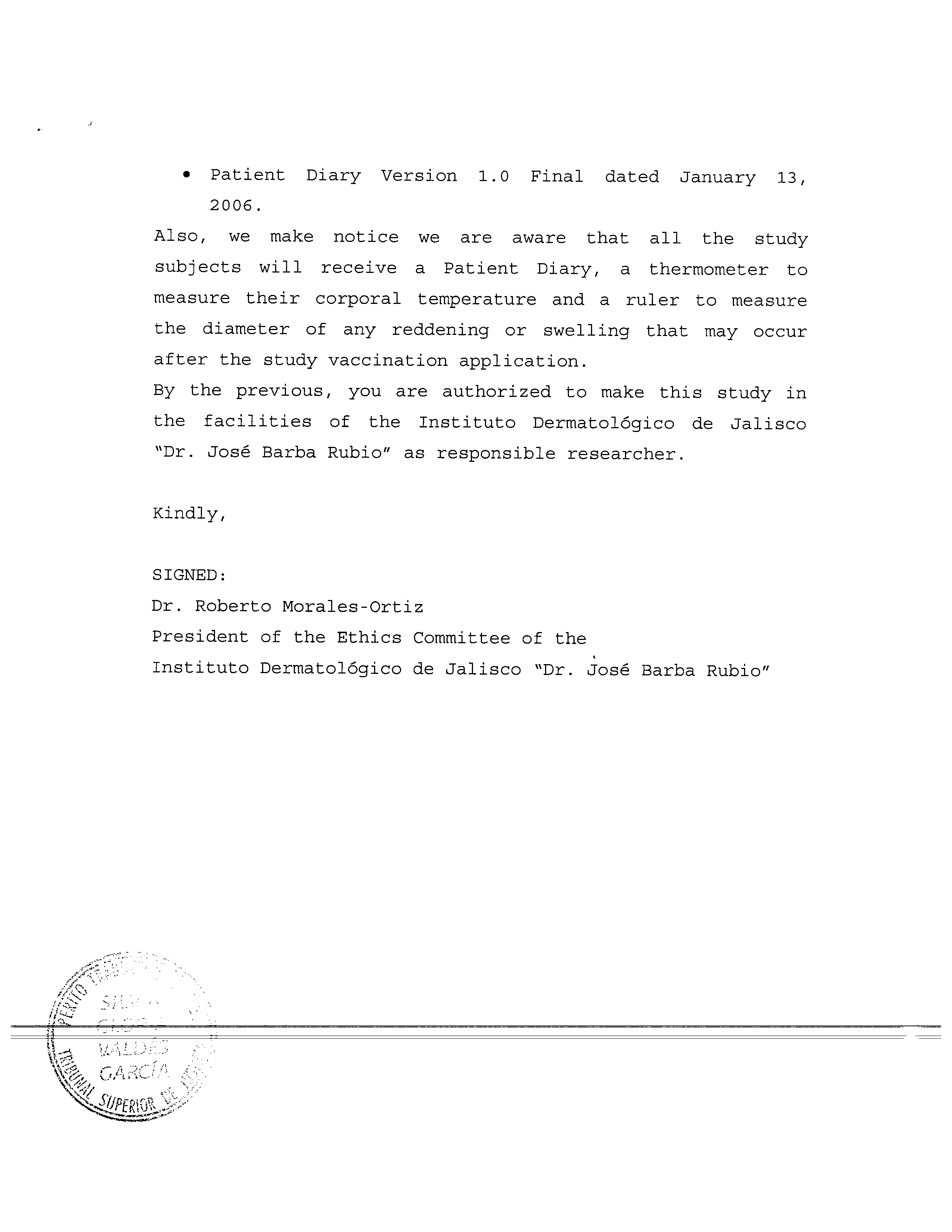

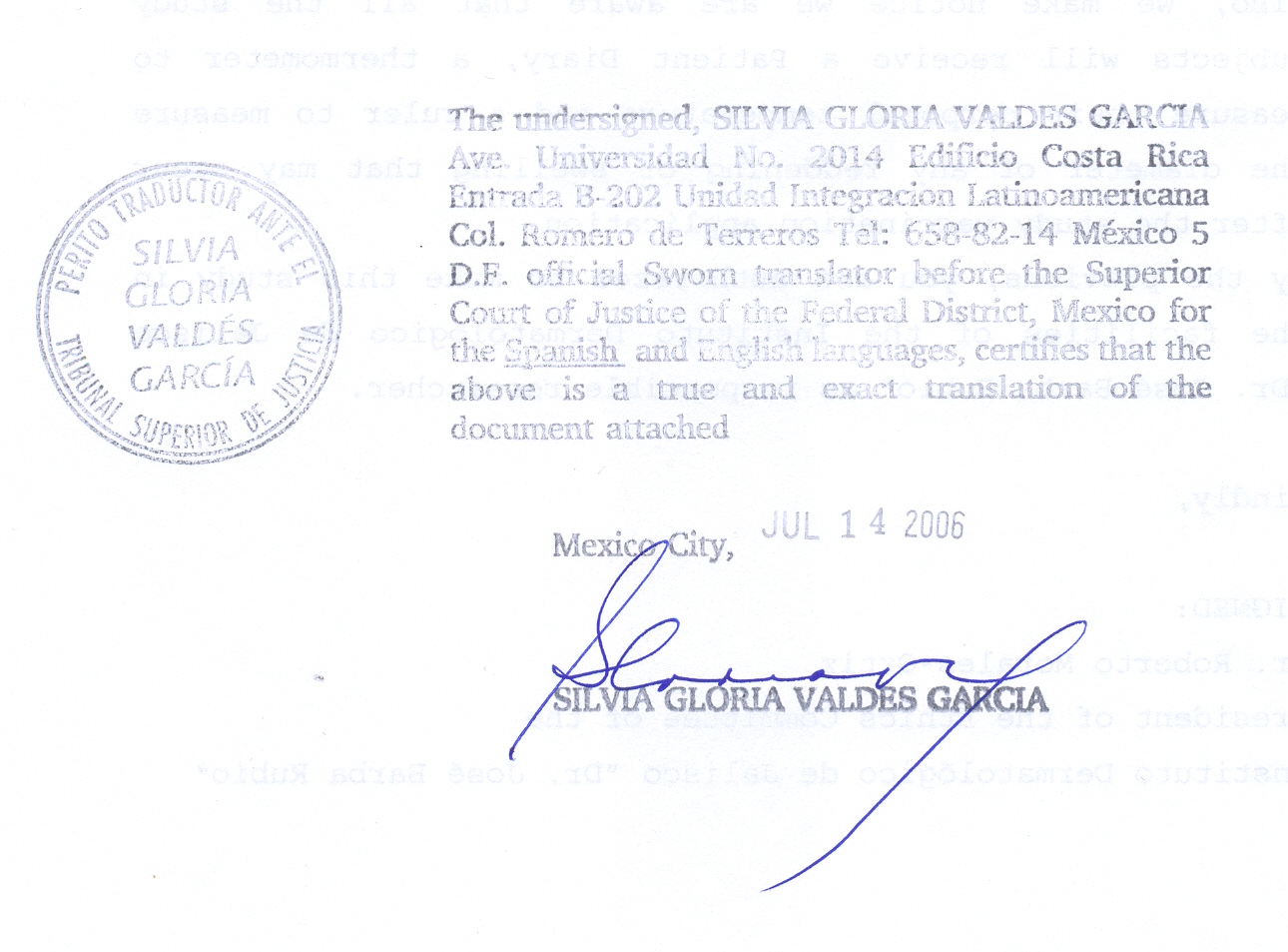


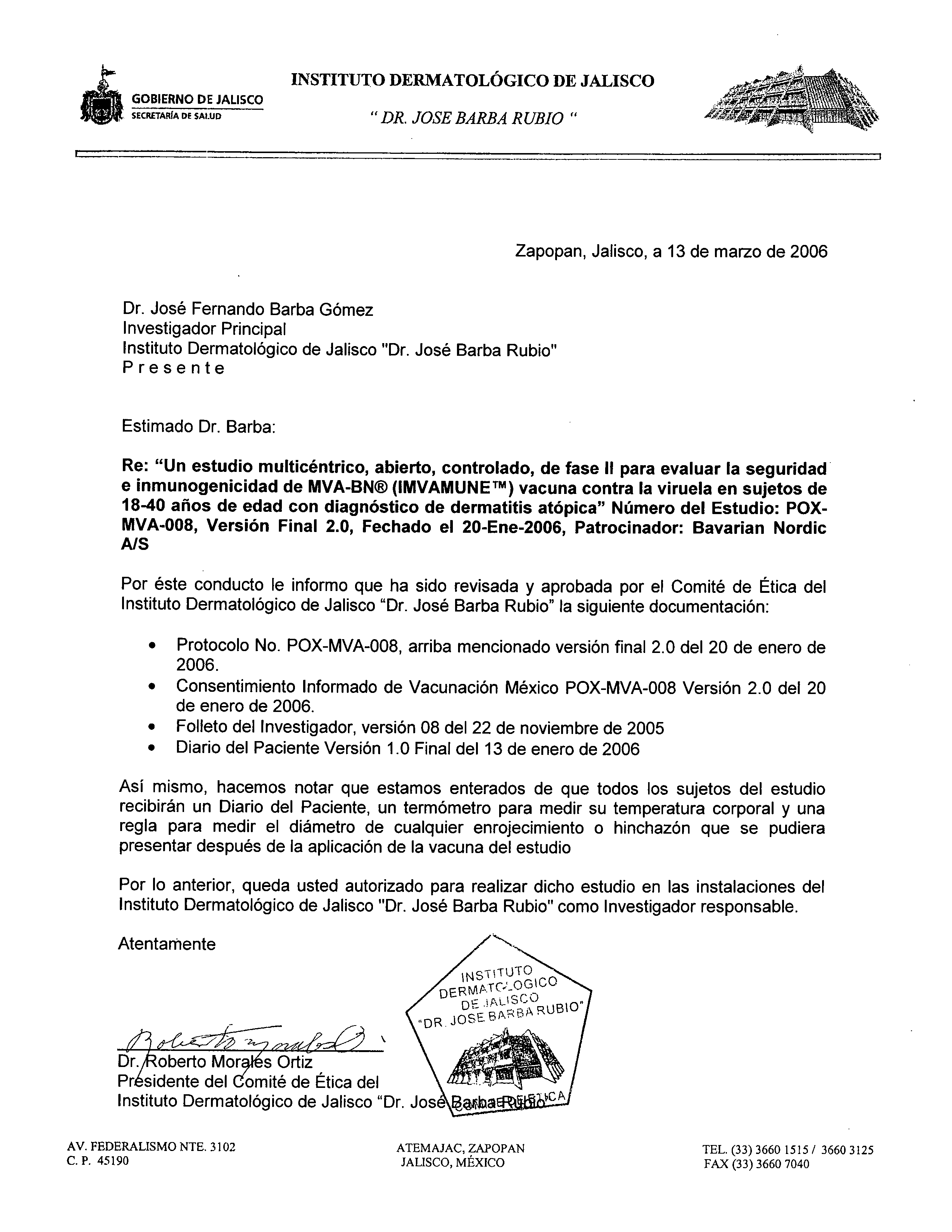


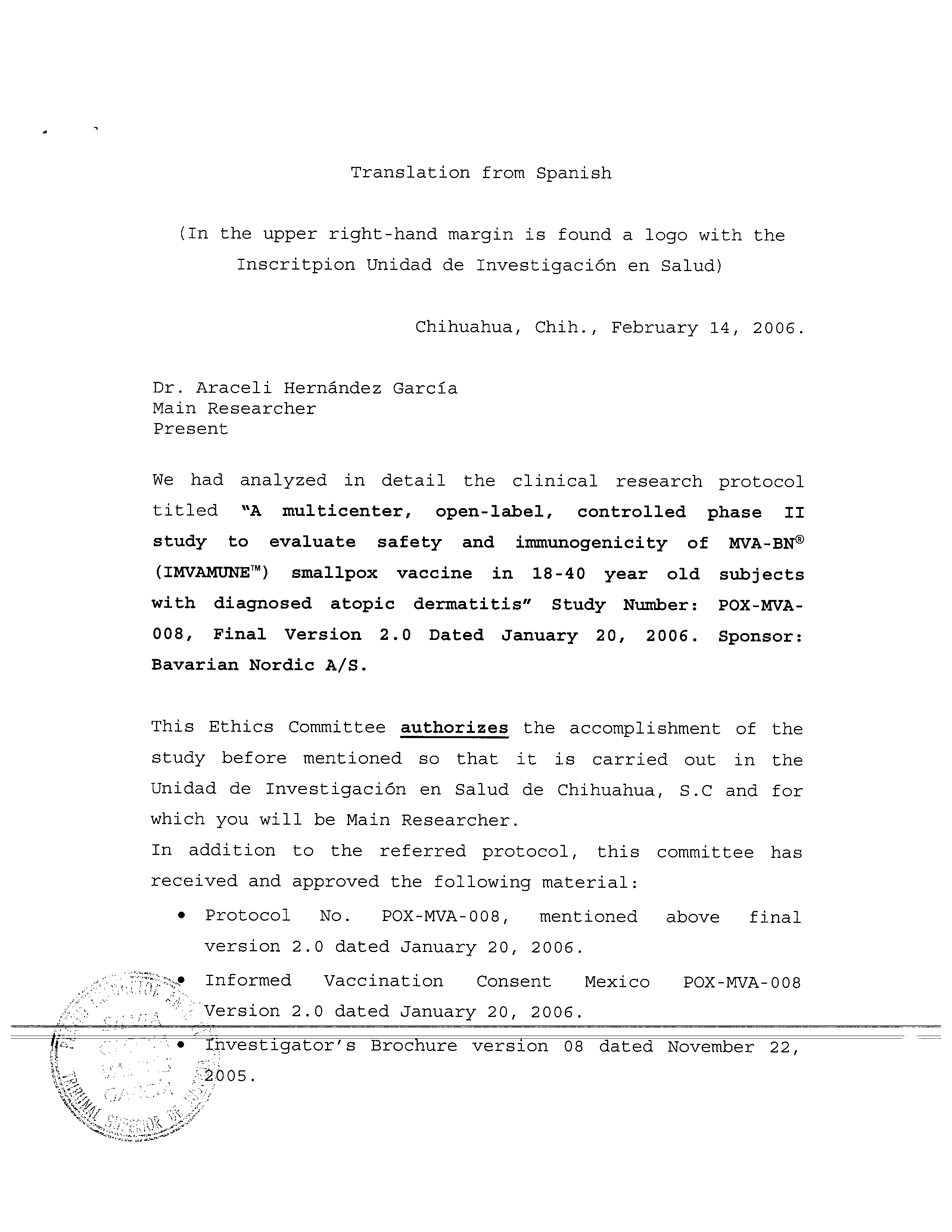

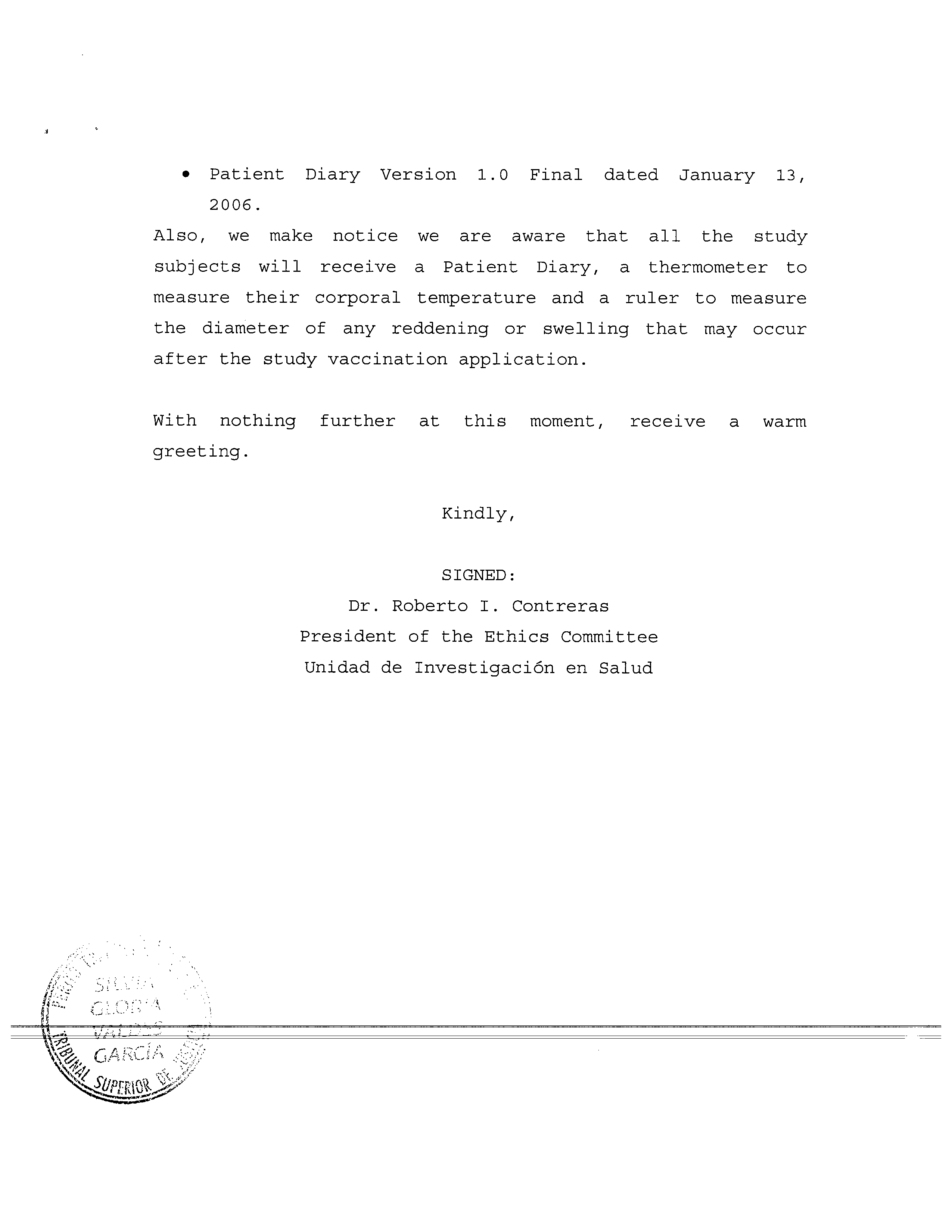

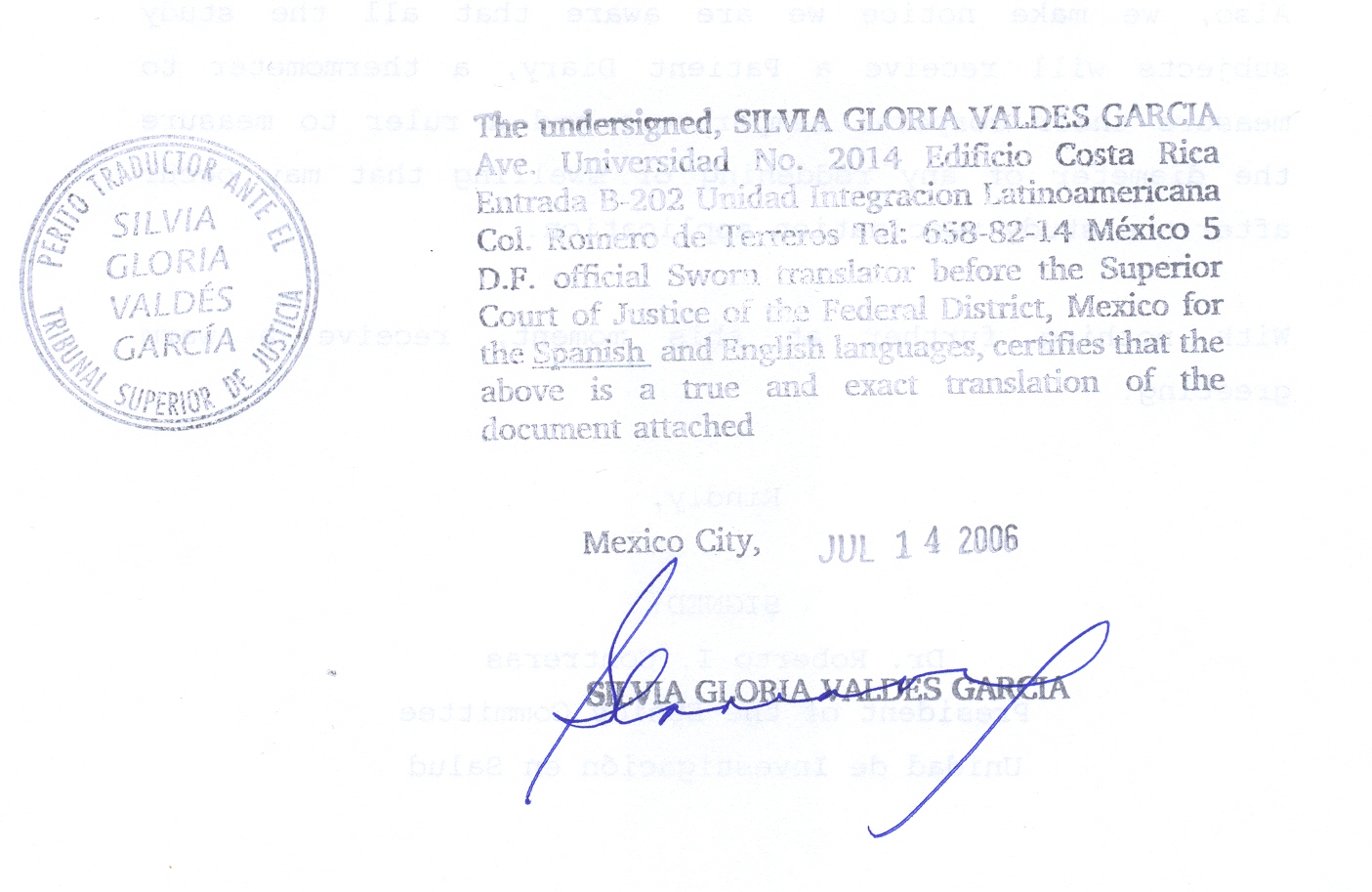


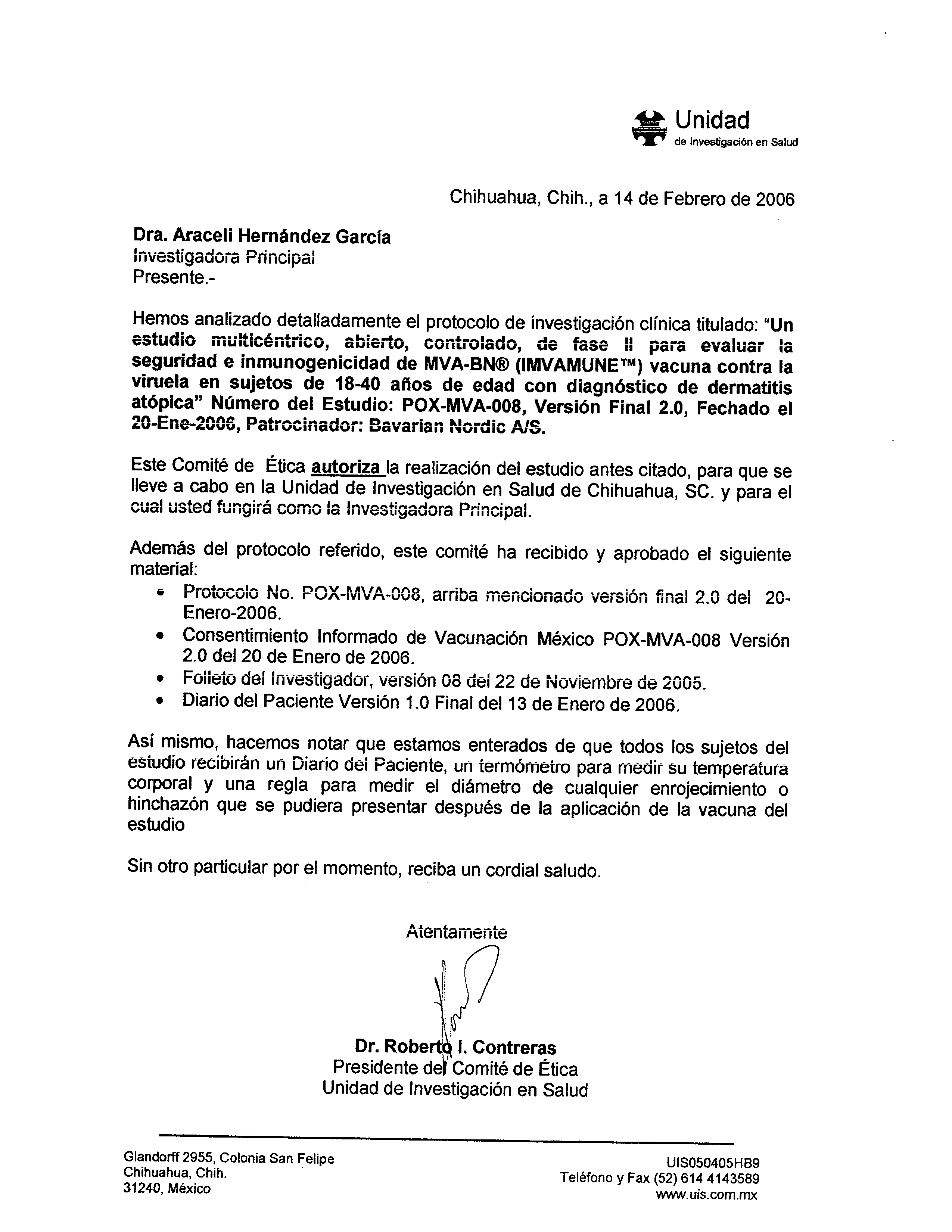


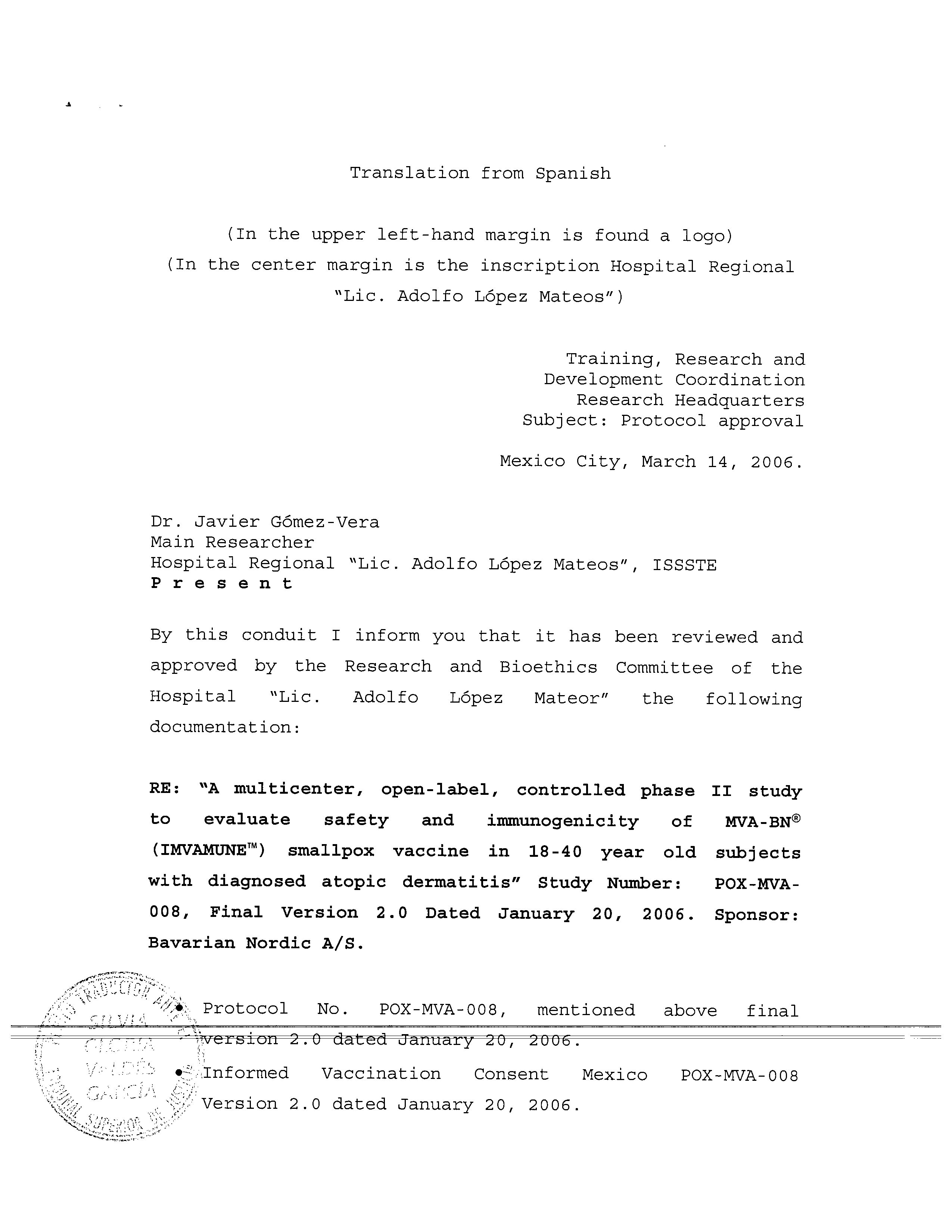

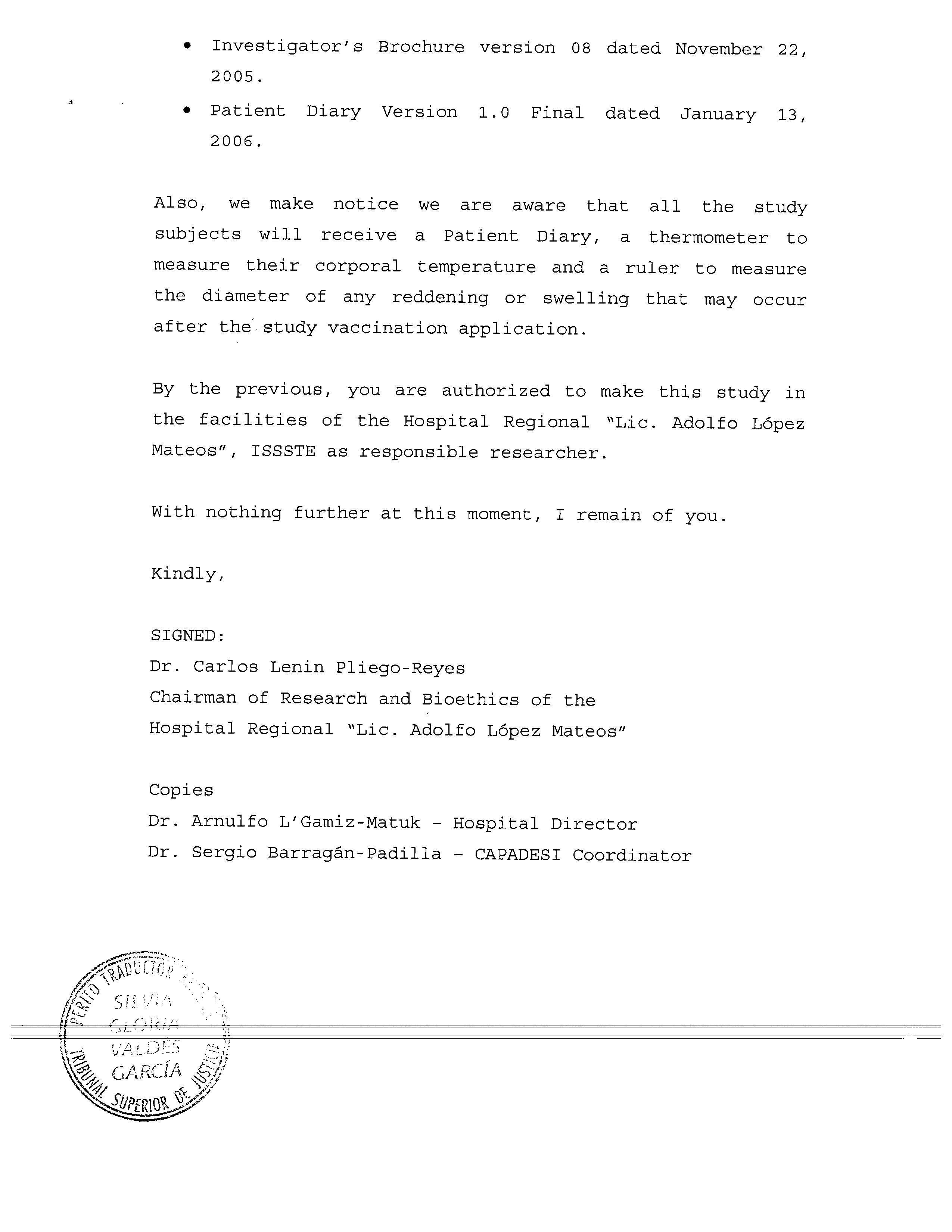

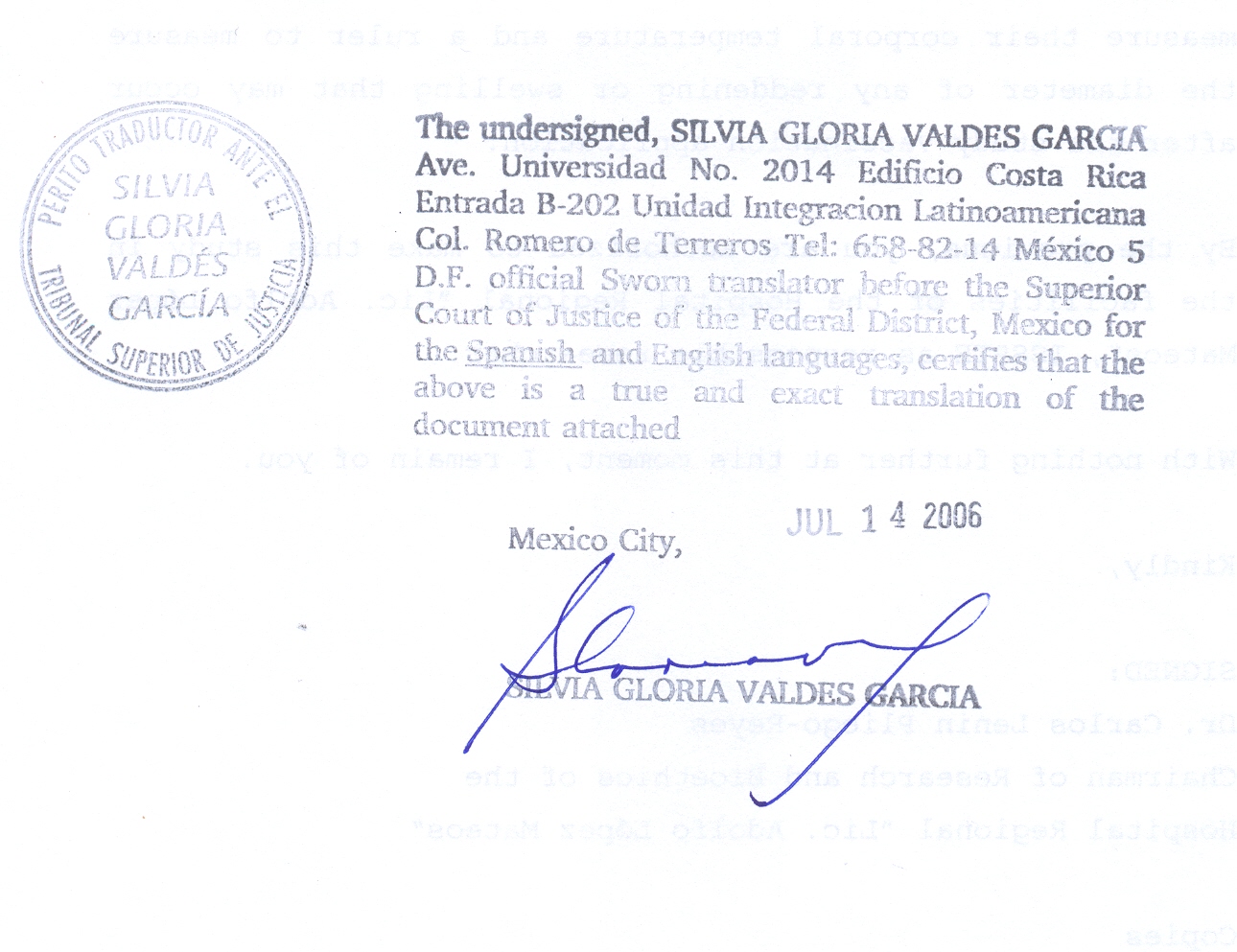


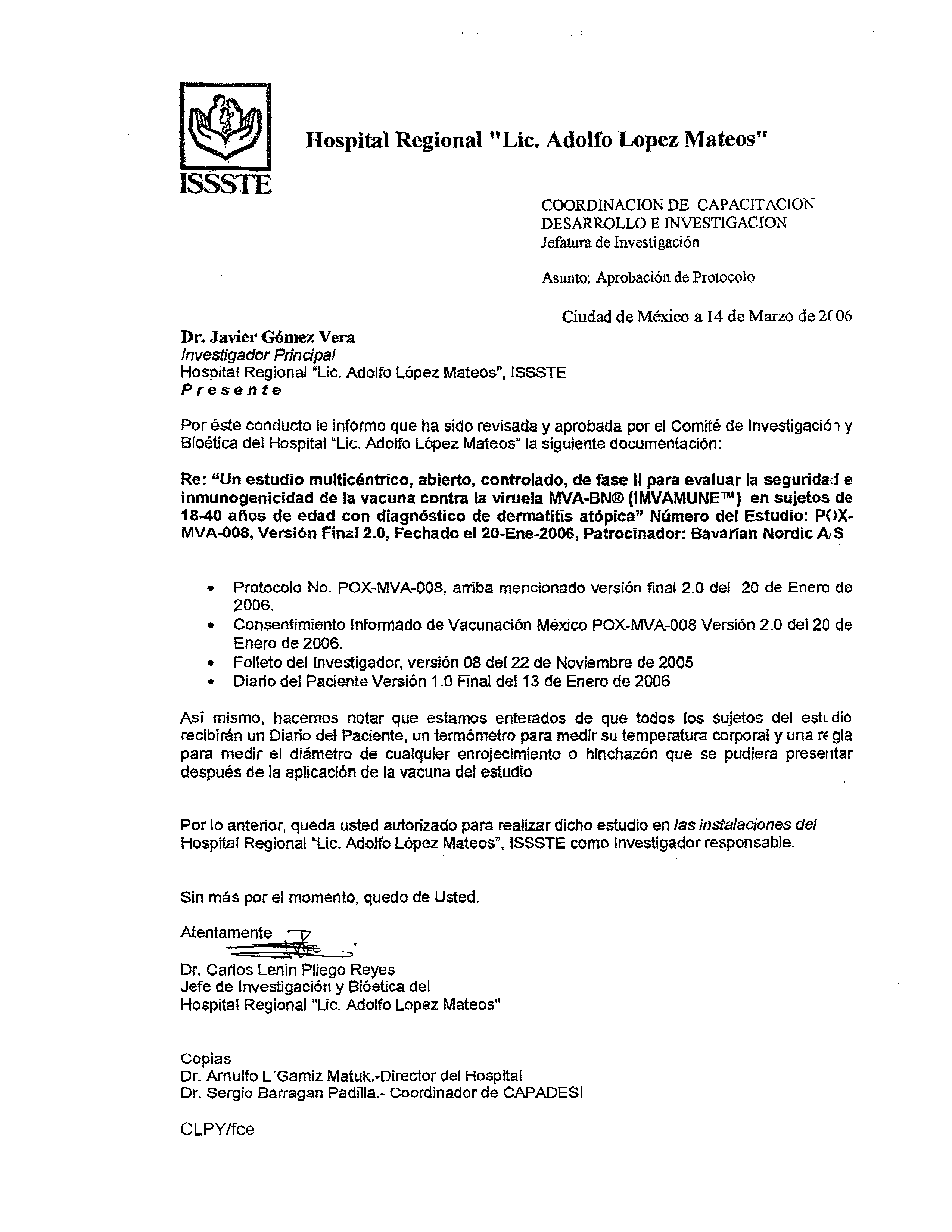


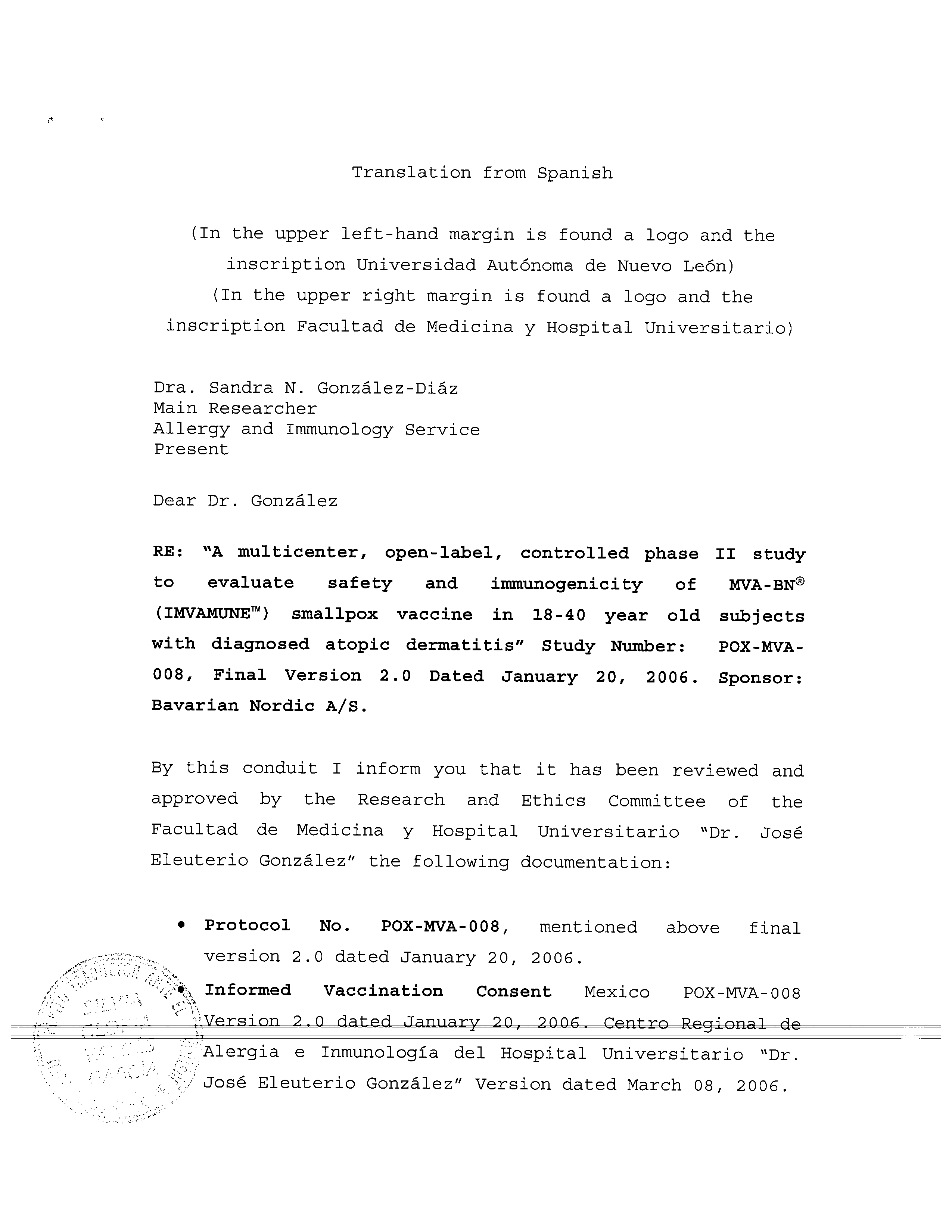

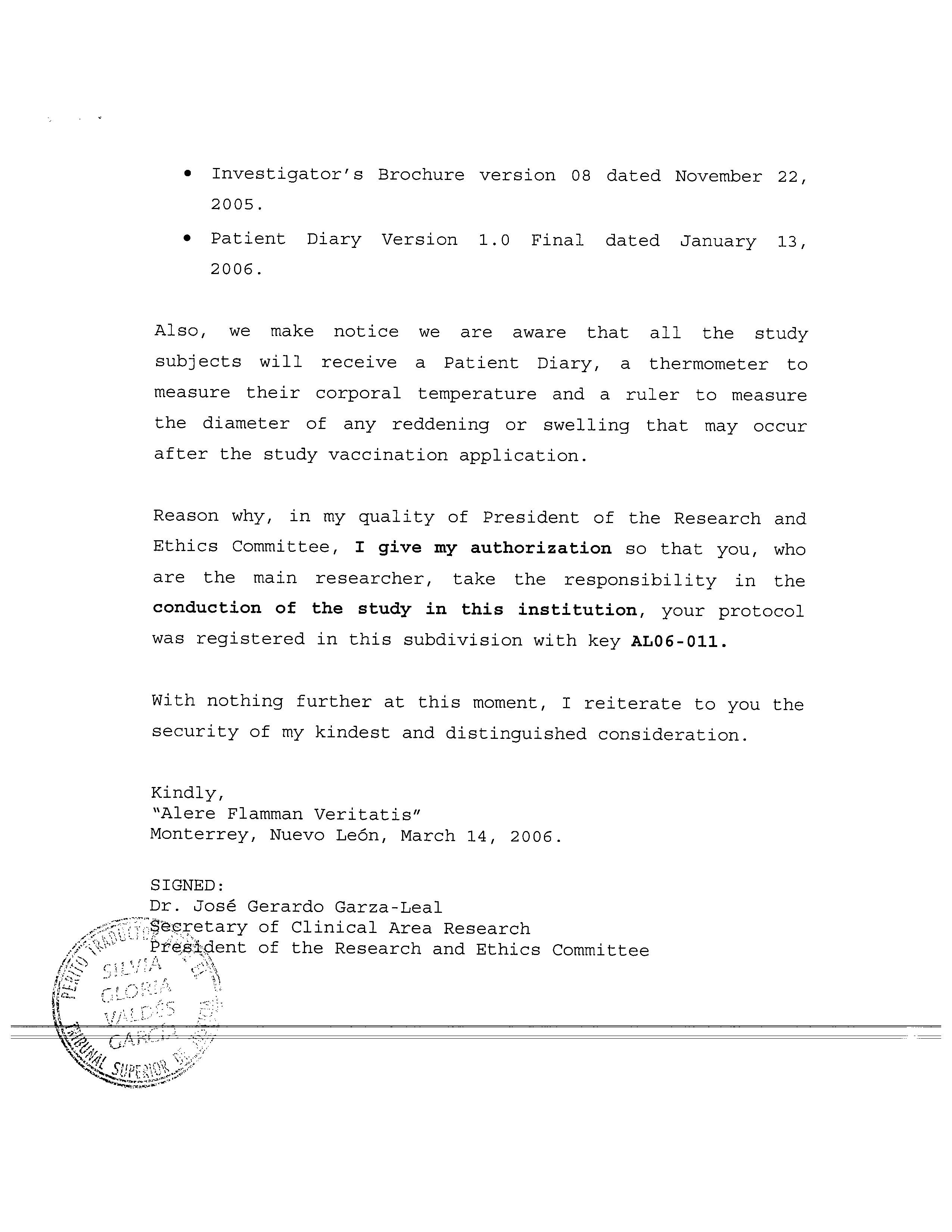

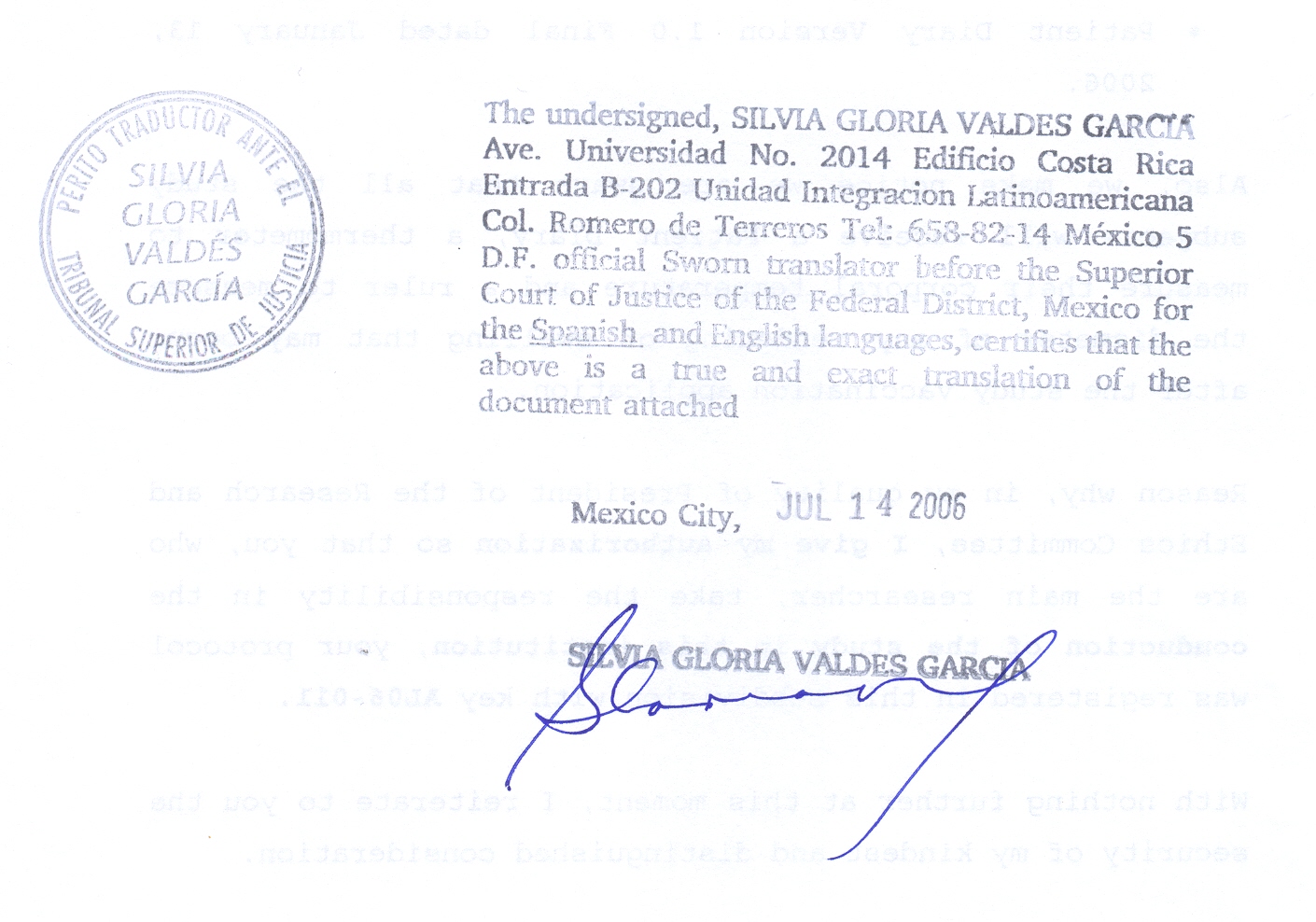


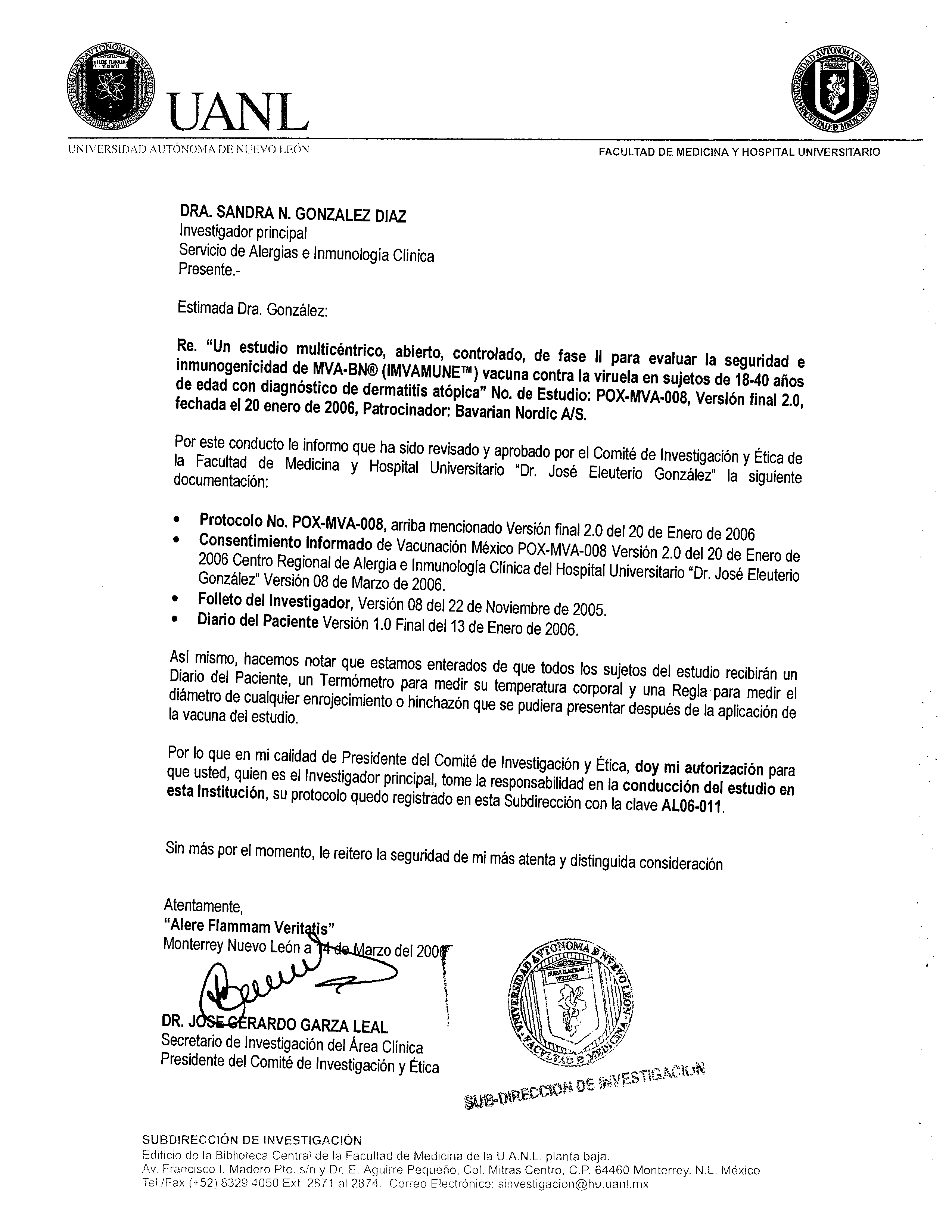

Supplement: S1 IRB — (DOC) [file pone.0138348.s007.doc]
