# Supplementary material for: A Multicenter, Open-Label, Controlled Phase II Study to Evaluate Safety and Immunogenicity of MVA Smallpox Vaccine (IMVAMUNE) in 18–40 Year Old Subjects with Diagnosed Atopic Dermatitis
Source: PLoS One. 2015 Oct 6;10(10):e0138348. doi: 10.1371/journal.pone.0138348 (PMC4595076; doi:10.1371/journal.pone.0138348)
Supplement: S5 Fig — (DOCX) [file pone.0138348.s006.docx]

**Supplemental Figures – ELISA and PRNT Seroconversion.**

Seroconversion rates determined by vaccinia-specific ELISA and PRNT. Data set is PPS (per protocol set), N = 451 (for Week 28 N = 170). Error bars represent upper and lower confidence intervals. Vaccinations were given at Week 0 and Week 4. AD = atopic dermatitis.

**Supplemental Figures – Antibody Titers.** Geometric mean titers determined by vaccinia-specific ELISA or PRNT. Data set is PPS (per protocol set), N = 451 (for Week 28 N = 170). Error bars represent upper and lower confidence intervals. Vaccinations were given at Week 0 and Week 4. AD = atopic dermatitis.
