# Supplementary material for: A Multicenter, Open-Label, Controlled Phase II Study to Evaluate Safety and Immunogenicity of MVA Smallpox Vaccine (IMVAMUNE) in 18–40 Year Old Subjects with Diagnosed Atopic Dermatitis
Source: PLoS One. 2015 Oct 6;10(10):e0138348. doi: 10.1371/journal.pone.0138348 (PMC4595076; doi:10.1371/journal.pone.0138348)
Supplement: S2 IRB — (PDF) [file pone.0138348.s008.pdf]

List of IECs / IRBs

Site No.	Principal Investigator	IEC / IRB – Site	IEC/IRB Address	Country
001	Galan Herrera, Juan F	Comité de Inv. para Estudios en Humanos	Puente de Piedra No.150, Col. Toriello Guerra, Tlalpan<br>Mexico City,<br>Mexico 14050	MEXICO
002	Barba Gomez, Jose F	Comité de Ética del Instituto Dermatológico de Jalisco	Avenida Federalismo Norte 3120 Col. Atemajac del Valle<br>Zapopan, Jalisco,<br>Mexico 45190	MEXICO
003	Hernandez Garcia, Araceli	Comité de Ética de la Unidad de Investigación en Salud de Chihuahua	Ortiz Mena 2200, Fracc. Las Palmas, Chihuahua, Chih.<br>Chihuahua 31205	MEXICO
004	Gomez Vera, Javier	Comde Inv y Bioética del Hosp Adolfo López M	Av. Universidad No. 1321, Col. Florida<br>Mexico City,<br>Mexico City 01030	MEXICO
005	Gonzalez Diaz, Sandra N	Comité de Investigación y Ética de la Facultad de Medicina y Hospital Universitario "Dr. José Eleuterio González" Universidad Autónoma de Nuevo León	Av. Francisco I. Madero Pte s/n y Av.Gonzalitos s/n. Col. Mitras Centro<br>Monterrey,<br>Nuevo León 64460	MEXICO
013	Rojo Gutierrez, Maria I	Comité de Ética en Investigación del Hospital Juárez de México	Av. Instituto Politécnico Nacional #5160,<br>Col. Magdalena de las Salinas,<br>Mexico DF,<br>CP 07760	MEXICO
015	Mata Lara, Maria G	Comité de Ética del Hospital "Doctor Ángel Leaño"	Av. Dr. Ángel Leaño #500 Esq. Av. Juan Gil, Col. Los Robles,<br>Zapopan, Jalisco,<br>CP 45200	MEXICO
008	Greenberg, Richard N	University of	315 Kinkead Hall,	USA

		Kentucky	University of Kentucky;<br>Lexington, KY 40506	
009	Rich, Phoebe	IntegReview IRB	3001 S. Lamar Blvd.,<br>Suite 210<br>Austin, TX 78704	USA
011	Mraz, Serena M	IntegReview IRB	3001 S. Lamar Blvd.,<br>Suite 210<br>Austin, TX 78704	USA
012	Lundell, Stefanie	IntegReview IRB	3001 S. Lamar Blvd.,<br>Suite 210<br>Austin, TX 78704	USA
017	Davis, Steven A	IntegReview IRB	3001 S. Lamar Blvd.,<br>Suite 210<br>Austin, TX 78704	USA
018	Dinh, Dinh V	IntegReview IRB	3001 S. Lamar Blvd.,<br>Suite 210<br>Austin, TX 78704	USA
020	Hurley, Yadira	Saint Louis<br>University IRB	3556 Caroline Street;<br>Caroline Bldg C110;<br>St. Louis, MO 63104	USA
021	Laumann, Anne	Northwestern<br>University	Rubloff Hall, 7th Floor;<br>750 N. Lake Shore Dr.,<br>Chicago, IL 60611	USA
022	Tu, John H	IntegReview IRB	3001 S. Lamar Blvd.,<br>Suite 210<br>Austin, TX 78704	USA
023	Stough, IV, Dowling B	IntegReview IRB	3001 S. Lamar Blvd.,<br>Suite 210<br>Austin, TX 78704	USA
024	Tschen, Eduardo H	IntegReview IRB	3001 S. Lamar Blvd.,<br>Suite 210<br>Austin, TX 78704	USA
025	Wadsworth, L. (Larkin)<br>Tyler T	IntegReview IRB	3001 S. Lamar Blvd.,<br>Suite 210<br>Austin, TX 78704	USA
026	Essink, Brandon J	IntegReview IRB	3001 S. Lamar Blvd.,<br>Suite 210<br>Austin, TX 78704	USA
027	Abramovits, William	IntegReview IRB	3001 S. Lamar Blvd.,<br>Suite 210<br>Austin, TX 78704	USA
028	Kaplan, David L	IntegReview IRB	3001 S. Lamar Blvd.,<br>Suite 210<br>Austin, TX 78704	USA
