# Supplementary material for: A Multicenter, Open-Label, Controlled Phase II Study to Evaluate Safety and Immunogenicity of MVA Smallpox Vaccine (IMVAMUNE) in 18–40 Year Old Subjects with Diagnosed Atopic Dermatitis
Source: PLoS One. 2015 Oct 6;10(10):e0138348. doi: 10.1371/journal.pone.0138348 (PMC4595076; doi:10.1371/journal.pone.0138348)
Supplement: S3 IRB — (PDF) [file pone.0138348.s009.pdf]

UK

UNIVERSITY OF KENTUCKY

Dream • Challenge • Succeed

OFFICE OF RESEARCH INTEGRITY

REC'D SEP 11 2006

Initial Review Approval Ends	Project Ends	IRB Number
June 5, 2007	April 30, 2011	06-0402-F2L

TO: Richard N Greenberg, M.D.
Internal Medicine & Divisions
c/o Beth Plummer
Infectious Disease Clinical Trials Office
MN672 Medical Science Bldg. 0298
(859)323-6327

FROM: Chairperson/Vice Chairperson
Medical Institutional Review Board (IRB)

SUBJECT: Approval of Protocol Number 06-0402-F2L

DATE: September 7, 2006

On September 5, 2006, the Medical Institutional Review Board approved minor revisions requested at the convened meeting on June 6, 2006 for your protocol entitled:

06-ID-175: A Multicenter, Open-label, Controlled Phase II Study to Evaluate Safety and Ummunogenicity of MVA-BN (IMVAMUNE) Smallpox Vaccine in 18-40 Year Old Subjects with Diagnosed Atopic Dermatitis

Approval is effective from June 6, 2006 until June 5, 2007. This approval extends to any consent/assent document unless the IRB has waived the requirement for documentation of informed consent. If applicable, attached is the IRB approved consent/assent document(s) to be used when enrolling subjects. **[Note, subjects can only be enrolled using consent/assent forms which have a valid "IRB Approval" stamp unless special waiver has been obtained from the IRB.]** Prior to the end of this period, you will be sent a Continuation Review Report Form which must be completed and returned to the Office of Research Integrity so that the protocol can be reviewed and approved for the next period.

In implementing the research activities, you are responsible for complying with IRB decisions, conditions and requirements. The research procedures should be implemented as approved in the IRB protocol. It is the principal investigator's responsibility to ensure any changes planned for the research are submitted for review and approval by the IRB prior to implementation. Protocol changes made without prior IRB approval to eliminate apparent hazards to the subject(s) should be reported in writing immediately to the IRB. Furthermore, discontinuing a study or completion of a study is considered a change in the protocol's status and therefore the IRB should be promptly notified in writing.

For information describing investigator responsibilities after IRB approval has been obtained, download and read the document "PI Guidance to Responsibilities, Qualifications, Records and Documentation of Human Subjects Research" from the Office of Research Integrity's Guidance/Policy Documents web page [http://www.research.uky.edu/ori/human/guidance.htm#PIresp]. Additional information regarding IRB review, federal regulations, and institutional policies may be found through ORI's web site [http://www.research.uky.edu/ori/]. If you have questions, need additional information, or would like a paper copy of the above mentioned document, contact the Office of Research Integrity at (859) 257-9428.

Edward Hirschowitz, M.D. /lds
Chairperson/Vice Chairperson

University Veterinarian, IRB, RDRC, IACUC
315 Kinkead Hall • Lexington, Kentucky 40506-0057
(859) 257-9428 • *fax* (859) 257-8995
www.research.uky.edu/ori
An Equal Opportunity University
