# Supplementary material for: A Multicenter, Open-Label, Controlled Phase II Study to Evaluate Safety and Immunogenicity of MVA Smallpox Vaccine (IMVAMUNE) in 18–40 Year Old Subjects with Diagnosed Atopic Dermatitis
Source: PLoS One. 2015 Oct 6;10(10):e0138348. doi: 10.1371/journal.pone.0138348 (PMC4595076; doi:10.1371/journal.pone.0138348)
Supplement: S1 Patents — (DOCX) [file pone.0138348.s010.docx]

Patents by Inventor Paul Chaplin regarding MVA

[**RECOMBINANT MODIFIED VACCINIA VIRUS ANKARA (MVA) RESPIRATORY SYNCYTIAL VIRUS (RSV) VACCINE**](http://patents.justia.com/patent/20150209421)

**Application number:** 20150209421

**Inventors:** Cédric Cheminay, Robin Steigerwald, Paul Chaplin

[**POXVIRAL VECTORS FOR LOW ANTIBODY RESPONSE AFTER A FIRST PRIMING IMMUNIZATION**](http://patents.justia.com/patent/20150174238)

**Application number:** 20150174238

**Inventors:** Robin Steigerwald, Kay Brinkmann, Ulrike Dirmeier, Paul Chaplin

[**USE OF A MODIFIED POXVIRUS FOR THE RAPID INDUCTION OF IMMUNITY AGAINST A POXVIRUS OR OTHER INFECTIOUS AGENTS**](http://patents.justia.com/patent/20140341946)

**Application number:** 20140341946

**Inventors:** Paul CHAPLIN, Luis MATEO

[**RECOMBINANT POXVIRUS VECTOR COMPRISING TETANUS TOXIN FRAGMENT C**](http://patents.justia.com/patent/20140322265)

**Application number:** 20140322265

**Inventors:** Paul Chaplin, Robin Steigerwald

[**Use of a modified poxvirus for the rapid induction of immunity against a poxvirus or other infectious agents**](http://patents.justia.com/patent/8808709)

**Patent number:** 8808709

**Inventors:** Paul Chaplin, Luis Mateo

[**Modified Vaccinia Ankara virus variant and cultivation method**](http://patents.justia.com/patent/8470598)

**Patent number:** 8470598

**Inventors:** Paul Chaplin, Paul Howley, Christine Meisinger-Henschel, Ingmar Rathe, Eva Felder, Karl Heller

[**Modified vaccinia virus ankara for the vaccination of neonates**](http://patents.justia.com/patent/8372622)

**Patent number:** 8372622

**Inventors:** Mark Suter, Sabine Vollstedt, Paul Chaplin

[**MODIFIED VACCINIA ANKARA VIRUS VARIANT**](http://patents.justia.com/patent/20120328650)

**Application number:** 20120328650

**Inventors:** PAUL CHAPLIN, PAUL HOWLEY, CHRISTINE MEISINGER-HENSCHEL

[**Method for generating a stable recombinant modified vaccinia virus ankara (MVA) comprising human immunodeficiency virus (HIV) genes inserted into one or more intergenic regions (IGRs)**](http://patents.justia.com/patent/8323661)

**Patent number:** 8323661

**Inventors:** Paul Howley, Sonja Leyrer, Paul Chaplin, Eva Felder

[**Recombinant modified vaccinia ankara (MVA) virus containing heterologous DNA inserts encoding human immunodeficiency virus (HIV) antigens inserted into one or more intergenic regions (IGRs)**](http://patents.justia.com/patent/8309098)

**Patent number:** 8309098

**Inventors:** Paul Howley, Sonja Leyrer, Paul Chaplin, Eva Felder

[**MODIFIED VACCINIA ANKARA VIRUS VARIANT AND CULTIVATION METHOD**](http://patents.justia.com/patent/20120276613)

**Application number:** 20120276613

**Inventors:** PAUL CHAPLIN, PAUL HOWLEY, CHRISTINE MEISINGER-HENSCHEL, INGMAR RATHE, EVA FELDER, KARL HELLER

[**Modified Vaccinia Ankara virus variant**](http://patents.justia.com/patent/8268325)

**Patent number:** 8268325

**Inventors:** Paul Chaplin, Paul Howley, Christine Meisinger-Henschel

[**Modified virus variant**](http://patents.justia.com/patent/8268329)

**Patent number:** 8268329

**Inventors:** Paul Chaplin, Paul Howley, Christine Meisinger-Henschel

[**Modified Vaccinia Ankara virus variant and cultivation method**](http://patents.justia.com/patent/8236560)

**Patent number:** 8236560

**Inventors:** Paul Chaplin, Paul Howley, Christine Meisinger-Henschel, Ingmar Rathe, Eva Felder, Karl Heller

[**MODIFIED VACCINIA VIRUS ANKARA FOR THE VACCNATION OF NEONATES**](http://patents.justia.com/patent/20120183574)

**Application number:** 20120183574

**Inventor:** Paul Chaplin

[**Method for introducing human immunodeficiency virus nucleotide sequences into a cell utilizing modified vaccinia virus Ankara (MVA) recombinants comprising HIV genes inserted into one or more intergenic regions (IGRs)**](http://patents.justia.com/patent/8198088)

**Patent number:** 8198088

**Inventors:** Paul Howley, Sonja Leyrer, Paul Chaplin, Eva Felder

[**Method for the production of human immunodeficiency virus (HIV) proteins utilizing modified vaccinia virus ankara (MVA) recombinants comprising HIV genes inserted into one or more intergenic regions (IGRs)**](http://patents.justia.com/patent/8197822)

**Patent number:** 8197822

**Inventors:** Paul Howley, Sonja Leyrer, Paul Chaplin, Eva Felder

[**GENERATION OF A BROAD T-CELL RESPONSE IN HUMANS AGAINST HIV**](http://patents.justia.com/patent/20120135032)

**Application number:** 20120135032

**Inventors:** Paul Chaplin, Richard Nichols

[**USE OF A MODIFIED POXVIRUS FOR THE RAPID INDUCTION OF IMMUNITY AGAINST A POXVIRUS OR OTHER INFECTIOUS AGENTS**](http://patents.justia.com/patent/20120107359)

**Inventors:** Paul CHAPLIN, Luis Mateo

[**Modified Vaccinia Virus Ankara for the vaccination of neonates**](http://patents.justia.com/patent/8163293)

**Patent number:** 8163293

**Inventor:** Paul Chaplin

[**INTERGENIC REGIONS AS NOVEL SITES FOR INSERTION OF HIV DNA SEQUENCES IN THE GENOME OF MODIFIED VACCINIA VIRUS ANKARA**](http://patents.justia.com/patent/20120014922)

**Application number:** 20120014922

**Inventors:** Paul Howley, Sonja Leyrer, Paul Chaplin, Eva Felder

[**INTERGENIC REGIONS AS NOVEL SITES FOR INSERTION OF HIV DNA SEQUENCES IN THE GENOME OF MODIFIED VACCINIA VIRUS ANKARA**](http://patents.justia.com/patent/20120015423)

**Application number:** 20120015423

**Inventors:** Paul HOWLEY, Sonja Leyrer, Paul Chaplin, Eva Felder

[**INTERGENIC REGIONS AS NOVEL SITES FOR INSERTION OF HIV DNA SEQUENCES IN THE GENOME OF MODIFIED VACCINIA VIRUS ANKARA**](http://patents.justia.com/patent/20120009214)

**Application number:** 20120009214

**Inventors:** Paul HOWLEY, Sonja Leyrer, Paul Chaplin, Eva Felder

[**INTERGENIC REGIONS AS NOVEL SITES FOR INSERTION OF HIV DNA SEQUENCES IN THE GENOME OF MODIFIED VACCINIA VIRUS ANKARA**](http://patents.justia.com/patent/20110306093)

**Inventors:** PAUL HOWLEY, SONJA LEYRER, PAUL CHAPLIN, EVA FELDER

[**Recombinant modified vaccinia virus ankara (MVA) comprising human immunodeficiency virus (HIV) genes inserted into one or more intergenic regions (IGRs)**](http://patents.justia.com/patent/8029800)

**Patent number:** 8029800

**Inventors:** Paul Howley, Sonja Leyrer, Paul Chaplin, Eva Felder

[**Methods for inducing an immune response in a host comprising administering modified vaccinia virus ankara (MVA) recombinants comprising human immunodeficiency virus (HIV) genes inserted into one or more intergenic regions (IGRs)**](http://patents.justia.com/patent/8021669)

**Inventors:** Paul Howley, Sonja Leyrer, Paul Chaplin, Eva Felder

[**MODIFIED VACCINIA ANKARA VIRUS VARIANT AND CULTIVATION METHOD**](http://patents.justia.com/patent/20110217757)

**Application number:** 20110217757

**Inventors:** PAUL CHAPLIN, PAUL HOWLEY, CHRISTINE MEISINGER-HENSCHEL, INGMAR RATHE, EVA FELDER, KARL HELLER

[**MODIFIED VACCINIA ANKARA VIRUS VARIANT**](http://patents.justia.com/patent/20110182933)

**Application number:** 20110182933

**Inventors:** PAUL CHAPLIN, PAUL HOWLEY, CHRISTINE MEISINGER

[**MODIFIED VACCINIA ANKARA VIRUS VARIANT**](http://patents.justia.com/patent/20110182932)

**Application number:** 20110182932

**Inventors:** PAUL CHAPLIN, PAUL HOWLEY, CHRISTINE MEISINGER

[**PHENOTYPIC AND GENOTYPIC DIFFERENCES OF MVA STRAINS**](http://patents.justia.com/patent/20110172407)

**Application number:** 20110172407

**Inventor:** PAUL CHAPLIN

[**MODIFIED VACCINIA VIRUS ANKARA FOR THE VACCINATION OF NEONATES**](http://patents.justia.com/patent/20110159032)

**Application number:** 20110159032

**Inventors:** Mark Suter, Sabine Vollstedt, Paul Chaplin

[**Modified vaccinia ankara virus variant and cultivation method**](http://patents.justia.com/patent/7964398)

**Patent number:** 7964398

**Inventors:** Paul Chaplin, Paul Howley, Christine Meisinger-Henschel, Ingmar Rathe, Eva Felder, Karl Heller

[**Modified vaccinia ankara virus variant and cultivation method**](http://patents.justia.com/patent/7964395)

**Patent number:** 7964395

**Inventors:** Paul Chaplin, Paul Howley, Christine Meisinger-Henschel, Ingmar Rathe, Eva Felder, Karl Heller

[**Modified vaccinia ankara virus variant and cultivation method**](http://patents.justia.com/patent/7964396)

**Patent number:** 7964396

**Inventors:** Paul Chaplin, Paul Howley, Christine Meisinger-Henschel, Ingmar Rathe, Eva Felder, Karl Heller

[**Use of a modified poxvirus for the rapid induction of immunity against a poxvirus or other infectious agents**](http://patents.justia.com/patent/20110142877)

**Inventors:** Paul Chaplin, Luis Mateo

[**MODIFIED VACCINIA VIRUS ANKARA FOR THE VACCNATION OF NEONATES**](http://patents.justia.com/patent/20110135683)

**Application number:** 20110135683

**Inventor:** Paul Chaplin

[**Modified Vaccinia Ankara virus variant**](http://patents.justia.com/patent/7939086)

**Patent number:** 7939086

**Inventors:** Paul Chaplin, Paul Howley, Christine Meisinger-Henschel

[**Modified Vaccinia Ankara virus variant**](http://patents.justia.com/patent/7923017)

**Inventors:** Paul Chaplin, Paul Howley, Christine Meisinger-Henschel

[**RECOMBINANT MODIFIED VACCINIA VIRUS MEASLES VACCINE**](http://patents.justia.com/patent/20110052627)

**Application number:** 20110052627

**Inventor:** Paul Chaplin

[**Modified vaccinia virus ankara for the vaccination of neonates**](http://patents.justia.com/patent/7897156)

**Patent number:** 7897156

**Inventors:** Mathias Ackermann, Mark Suter, Hans Peter Hefti, Ruth Hefti, legal representative, Marco Franchini, Sabine Vollstedt, Paul Chaplin

[**Modified vaccinia virus ankara for the vaccination of neonates**](http://patents.justia.com/patent/7892533)

**Patent number:** 7892533

**Inventors:** Mark Suter, Sabine Vollstedt, Paul Chaplin

[**MODIFIED VACCINIA ANKARA VIRUS VARIANT AND CULTIVATION METHOD**](http://patents.justia.com/patent/20100279386)

**Application number:** 20100279386

**Inventors:** PAUL CHAPLIN, PAUL HOWLEY, CHRISTINE MEISINGER-HENSCHEL, INGMAR RATHE, EVA FELDER, KARL HELLER

[**INTERGENIC REGIONS AS NOVEL SITES FOR INSERTION OF HIV DNA SEQUENCES IN THE GENOME OF MODIFIED VACCINIA VIRUS ANKARA**](http://patents.justia.com/patent/20100196992)

**Application number:** 20100196992

**Inventors:** PAUL HOWLEY, SONJA LEYRER, PAUL CHAPLIN, EVA FELDER

[**INTERGENIC REGIONS AS NOVEL SITES FOR INSERTION OF HIV DNA SEQUENCES IN THE GENOME OF MODIFIED VACCINIA VIRUS ANKARA**](http://patents.justia.com/patent/20100183663)

**Application number:** 20100183663

**Inventors:** PAUL HOWLEY, SONJA LEYRER, PAUL CHAPLIN, EVA FELDER

[**MODIFIED VACCINIA ANKARA VIRUS VARIANT**](http://patents.justia.com/patent/20100119545)

**Application number:** 20100119545

**Abstract:** The present invention provides an attenuated virus, which is derived from

**Inventors:** Paul Chaplin, Paul Howley, Christine Meisinger

[**MODIFIED VACCINIA VIRUS ANKARA FOR THE VACCINATION OF NEONATES**](http://patents.justia.com/patent/20100048683)

**Application number:** 20100048683

**Inventors:** Mark SUTER, Sabine Vollstedt, Paul Chaplin

[**PHENOTYPIC AND GENOTYPIC DIFFERENCES OF MVA STRAINS**](http://patents.justia.com/patent/20100011451)

**Application number:** 20100011451

**Inventor:** Paul Chaplin

[**Modified vaccinia virus ankara for the vaccination of neonates**](http://patents.justia.com/patent/7628980)

**Patent number:** 7628980

**Inventors:** Mark Suter, Sabine Vollstedt, Paul Chaplin

[**Modified vaccinia Ankara virus avriant**](http://patents.justia.com/patent/20090169579)

**Application number:** 20090169579

**Inventors:** Paul Chaplin, Paul Howley, Christine Meisinger

[**MODIFIED VACCINIA VIRUS ANKARA FOR THE VACCINATION OF NEONATES**](http://patents.justia.com/patent/20090104224)

**Application number:** 20090104224

**Inventors:** Mathias Ackermann, Mark Suter, Hans Peter Hefti, Marco Franchini, Sabine Vollstedt, Paul Chaplin

[**Intergenic regions as novel sites for insertion of HIV DNA sequences in the genome of Modified Vaccinia virus Ankara**](http://patents.justia.com/patent/7501127)

**Patent number:** 7501127

**Inventors:** Paul Howley, Sonja Leyrer, Paul Chaplin, Eva Felder

[**MODIFIED VACCINIA ANKARA VIRUS VARIANT AND CULTIVATION METHOD**](http://patents.justia.com/patent/20090017536)

**Application number:** 20090017536

**Inventors:** Paul Chaplin, Paul Howley, Christine Meisinger-Henschel, Ingmar Rathe, Eva Felder, Karl Heller

[**MODIFIED VACCINIA ANKARA VIRUS VARIANT AND CULTIVATION METHOD**](http://patents.justia.com/patent/20080317778)

**Application number:** 20080317778

**Inventors:** Paul Chaplin, Paul Howley, Christine Meisinger-Henschel, Ingmar Rathe, Eva Felder, Karl Heller

[**Modified Vaccinia Ankara virus variant**](http://patents.justia.com/patent/7459270)

**Patent number:** 7459270

**Inventors:** Paul Chaplin, Paul Howley, Christine Meisinger-Henschel

[**Modified Vaccinia Ankara virus variant and cultivation method**](http://patents.justia.com/patent/7445924)

**Patent number:** 7445924

**Inventors:** Paul Chaplin, Paul Howley, Christine Meisinger, Ingmar Rathe, Eva Felder, Karl Heller

[**Modified Vaccinia Ankara virus variant**](http://patents.justia.com/patent/7384644)

**Patent number:** 7384644

**Inventors:** Paul Chaplin, Paul Howley, Christine Meisinger-Henschel

[**Modified vaccinia Ankara virus variant**](http://patents.justia.com/patent/20080089907)

**Application number:** 20080089907

**Inventors:** Paul Chaplin, Paul Howley, Christine Meisinger-Henschel

[**Modified Vaccinia Ankara virus variant**](http://patents.justia.com/patent/7335364)

**Patent number:** 7335364

**Inventors:** Paul Chaplin, Paul Howley, Christine Meisinger-Henschel

[**Modified vaccinia ankara virus variant**](http://patents.justia.com/patent/7189536)

**Patent number:** 7189536

**Inventors:** Paul Chaplin, Paul Howley, Christine Meisinger

[**Modified vaccinia ankara virus variant**](http://patents.justia.com/patent/20060280758)

**Application number:** 20060280758

**Inventors:** Paul Chaplin, Paul Howley, Christine Meisinger-Henschel

[**Modified vaccinia virus ankara for the vaccination of neonates**](http://patents.justia.com/patent/7097842)

**Patent number:** 7097842

**Inventors:** Mark Suter, Sabine Vollstedt, Paul Chaplin

[**Intergenic regions as novel sites for insertion of HIV DNA sequences in the genome of modified vaccinia virus ankara**](http://patents.justia.com/patent/20060188961)

**Application number:** 20060188961

**Inventors:** Paul Howley, Sonja Leyrer, Paul Chaplin, Eva Felder

[**Modified Vaccinia virus Ankara for the vaccination of neonates**](http://patents.justia.com/patent/20060127984)

**Application number:** 20060127984

**Inventors:** Mathias Ackermann, Mark Suter, Hans Hefti, Marco Franchini, Sabine Vollstedt, Paul Chaplin

[**Modified vaccinia ankara virus variant**](http://patents.justia.com/patent/20050271688)

**Application number:** 20050271688

**Inventors:** Paul Chaplin, Paul Howley, Christine Meisinger-Henschel

[**Modified vaccinia virus ankara for the vaccination of neonates**](http://patents.justia.com/patent/20050260156)

**Application number:** 20050260156

**Inventors:** Mark Suter, Sabine Vollstedt, Paul Chaplin

[**Modified vaccinia ankara virus variant and cultivation method**](http://patents.justia.com/patent/20050214323)

**Application number:** 20050214323

**Inventors:** Paul Chaplin, Paul Howley, Christine Meisinger-Henschel, Ingmar Rathe, Eva Felder, Karl Heller

[**Modified Vaccinia Ankara virus variant**](http://patents.justia.com/patent/6913752)

**Patent number:** 6913752

**Inventors:** Paul Chaplin, Paul Howley, Christine Meisinger

[**Modified vaccinia ankara virus variant**](http://patents.justia.com/patent/6761893)

**Patent number:** 6761893

**Inventors:** Paul Chaplin, Paul Howley, Christine Meisinger

[**Modified Vaccinia Virus Ankara for the vaccination of neonates**](http://patents.justia.com/patent/20030224018)

**Application number:** 20030224018

**Abstract:** The invention concerns the use of a virus for the preparation of a

**Inventors:** Mathias Ackermann, Mark Suter, Hans Peter Hefti, Marco Franchini, Sabine Vollstedt, Paul Chaplin

[**Modified vaccinia ankara virus variant**](http://patents.justia.com/patent/20030215466)

**Application number:** 20030215466

**Inventors:** Paul Chaplin, Paul Howley, Christine Meisinger

[**Modified vaccinia ankara virus variant**](http://patents.justia.com/patent/20030206926)

**Application number:** 20030206926

**Issued:** November 6, 2003

**Inventors:** Paul Chaplin, Paul Howley, Christine Meisinger

[**Modified vaccinia ankara virus variant**](http://patents.justia.com/patent/20030202988)

**Application number:** 20030202988

**Issued:** October 30, 2003

**Inventors:** Paul Chaplin, Paul Howley, Christine Meisinger
