# Supplementary material for: A Multicenter, Open-Label, Controlled Phase II Study to Evaluate Safety and Immunogenicity of MVA Smallpox Vaccine (IMVAMUNE) in 18–40 Year Old Subjects with Diagnosed Atopic Dermatitis
Source: PLoS One. 2015 Oct 6;10(10):e0138348. doi: 10.1371/journal.pone.0138348 (PMC4595076; doi:10.1371/journal.pone.0138348)
Supplement: S1 Protocol — (PDF) [file pone.0138348.s011.pdf]

POX-MVA-008

DMID 05-0133

A multicenter, open-label, controlled phase II study to evaluate safety and immunogenicity of MVA-BN® (IMVAMUNE®) smallpox vaccine in 18-40 year old subjects with diagnosed atopic dermatitis

RESTRICTED
BUSINESS PROPRIETARY

Revised Study Protocol #6, 03-Feb-2010

I. General Information

Site Signature Page

Site Signature Page for Revised Protocol #6

Herewith I agree that I have read and fully understood the Revised Protocol #6

POX-MVA-008

A multicenter, open-label, controlled phase II study to evaluate the safety and immunogenicity of MVA-BN® (IMVAMUNE®) smallpox vaccine in 18-40 year old subjects with diagnosed atopic dermatitis, Revised Protocol #6, dated 03-Feb-2010, DMID 05-0133

which reveals all the Information necessary to conduct the study. I agree that I will conduct the study according to the instructions given by this protocol. Furthermore I agree that I will conduct this study according to ICH GCP, the current version of the declaration of Helsinki, U.S. Code of Federal Regulations applicable to clinical studies and local legal and regulatory requirements.

I agree that all Information revealed in this protocol is handled strictly confidential.

Other than that I will permit trial related monitoring, audits, Ethics Committee review and regulatory inspections, providing direct access to source data/documents.

Date	[name]<br>Principal Investigator (PI), [site]
	[Site address]
Date	[name]<br>Designee, [site]

Signature Page

By signing the Revised Study Protocol Version # 6:

A multicenter, open-label, controlled phase II study to evaluate the safety and immunogenicity of MVA-BN® (IMVAMUNE®) smallpox vaccine in 18-40 year old subjects with diagnosed atopic dermatitis, Revised Protocol #6, dated 03-Feb-2010, DMID 05-0133

the undersigned parties agree that the Protocol was written according to international ethical and scientific quality standards (ICH GCP), in compliance with the current version of the Declaration of Helsinki, the U.S. Code of Federal Regulations applicable to clinical studies and local legal and regulatory requirements.

03-Feb-2010<br>Date	D. v. Bredow<br>**Dorothea von Bredow, Ph.D.**<br>**Clinical Project Leader**<br>**Bavarian Nordic GmbH**
03-Feb-2010<br>Date	[illegible]<br>**Alfred von Krempelhuber, Ph.D.**<br>**Director Clinical Operations,**<br>**Bavarian Nordic GmbH**
03-FEB-2010<br>Date	Nathaly Arndtz-Wiedemann<br>**Nathaly Arndtz-Wiedemann, MD**<br>**Medical Monitor**<br>**Bavarian Nordic GmbH**
03-Feb-2010<br>Date	Philip Young<br>**Philip Young, PhD**<br>**Biostatistician and Data Manager**<br>**Bavarian Nordic GmbH**
03-FEB-2010<br>Date	Thomas Meyer<br>**Thomas Meyer, PhD**<br>**Manager Clinical Analysis**<br>**Bavarian Nordic GmbH**
03-Feb-2010<br>Date	[illegible]<br>**Robert Semich, PhD**<br>**GCP Manager, Quality Assurance Development**<br>**Bavarian Nordic GmbH**

Signature Page (cont.)

By signing the Revised Study Protocol Version # 6:

A multicenter, open-label, controlled phase II study to evaluate the safety and immunogenicity of MVA-BN® (IMVAMUNE®) smallpox vaccine in 18-40 year old subjects with diagnosed atopic dermatitis, Revised Protocol #6, dated 03-Feb-2010, DMID 05-0133

the undersigned parties agree that the Protocol was written according to international ethical and scientific quality standards (ICH GCP), in compliance with the current version of the Declaration of Helsinki, the U.S. Code of Federal Regulations applicable to clinical studies and local legal and regulatory requirements.

03-Feb-2010	Richard N Greenberg MD
**Date**	**Prof. Dr. Richard N. Greenberg, M.D.**<br>**Coordinating Investigator**<br>**University of Kentucky**

II. Protocol Amendment #6

POX-MVA-008
DMID 05-0133

Amendment #6 to revised study protocol #5, dated 24-SEP-2008

A multicenter, open-label, controlled phase II study to evaluate safety and immunogenicity of MVA-BN® (IMVAMUNE®) smallpox vaccine in 18-40 year old subjects with diagnosed atopic dermatitis

Date of Amendment #6: 03-Feb-2010

Rationale

This Amendment implements the following changes to Bavarian Nordic Clinical Study Protocol POX-MVA-008:

The safety report for a potential Emergency Use Authorization (EUA) application described in Revised Protocol #5 will be integrated into the final clinical study report instead of being provided as a separate report.

In the original POX-MVA-008 study protocol, a cut-off value of 6 for the plaque reduction neutralization test (PRNT; secondary endpoint) was specified to define the term seroconversion . Since the PRNT has recently been redeveloped, a new cut-off value of 15 will be applied as specified in the revision of SOP/CA/017 (“Plaque Reduction Neutralization Test Using Vaccinia Virus Western Reserve”). Due to the redevelopment and subsequent revalidation of the assay which causes a delay in the planned analysis, the data for the secondary endpoint concerned will be presented in an addendum to the final study report.

The definitions for Safety Set (SAS) and Full Analysis Set (FAS) were found to be incomplete and therefore have been clarified:

Safety Set (SAS)

The analysis of safety will be performed on the safety analysis set. It consists of all subjects

- who had received at least one dose of study vaccine and
- for whom **any** safety data are available

Full-analysis set (FAS):

This is the subset of subjects

- who had received at least one dose of study vaccine and
- **for whom any data are available**

For the analysis of the enzyme-linked immunospot (ELISPOT) data, an ELISPOT analysis set (EAS) was defined. This was included to add an alternative to the per-protocol set, which is not dependent on the availability of serum samples, and hence more relevant to the analysis of the ELISPOT data.

The EAS consists of all subjects

- with a complete ELISPOT data set (Visits 1, 2 and 4 available)
- the administration of both study vaccinations
- without any relevant major protocol violations

In section 9.4.3 (Analysis of safety and reactogenicity), the two sentences regarding the listing of adverse events of special interest and of cardiac symptoms have been summarized and reduced to one sentence, since cardiac symptoms belong to the group of adverse events of special interest as defined in section 8.4.

Revised Protocol #5 did not specify in sufficient detail which information to collect during the telephone follow-up. Therefore these details have been added to Revised Protocol # 6. They include information on new serious adverse events (SAEs), adverse events (AEs) that were still ongoing at Visit 5 and adverse events of special interest. An interview on the atopic dermatitis (AD) status is not required in the telephone follow-up.

Administrative changes within the study team:

Kendle's local Medical Monitor, Luis Armando Bojorquez, MD, was replaced by Leonard Wassermann, MD, with immediate effect.

Bavarian Nordic's Medical Monitor, Garth Virgin, MD, was replaced by Nathaly Arndtz-Wiedemann, MD, with immediate effect.

Covance' laboratory Project Manager for both countries US and Mexico is now Scott Wooten (previously: Kellie Estes and Eric Steele).

"PPS", "SAS", "SIAS" and "EAS" had been added to and explained in the "list of abbreviations".

These changes to the study protocol are considered not to have any negative influence on the study procedures in general, on the safety of the study participants or on the validity of the main study results.

Changes

Changes/ added terms are highlighted in **bold** letters in the text, removed terms are ~~strikethrough~~.

Major changes may be made as follows:

**Final Protocol # 5,<br>dated 24-Sep-2008<br><br>Previously written**	**Amendment 6, Revised Protocol<br>dated 03-Feb-2010<br><br>Changed to:**
Page 12, Responsibilities	Page 13, Responsibilities
Medical Monitor:<br>~~Garth Virgin, MD~~<br>~~Director Drug Safety~~<br>Bavarian Nordic GmbH<br>Fraunhoferstr. 13<br>82152 Martinsried, Germany<br>~~Phone: +49-89-85651319~~<br>Fax: +49-89-85651419<br>~~e-mail: garth.virgin@bavarian-nordic.com~~	Medical Monitor:<br>**Nathaly Arndtz, MD**<br>**Vice President Medical Affairs**<br>Bavarian Nordic GmbH<br>Fraunhoferstr. 13<br>82152 Martinsried, Germany<br>**Phone: +49-89-85651308**<br>Fax: +49-89-85651419<br>**e-mail: nathaly.arndtz@bavarian-nordic.com**
Pages 13/ 14, Responsibilities	Page 14, Responsibilities
Covance CLS<br>~~US Contact: Kellie Estes~~<br>Project Manager<br>8211 SciCor Drive	Covance CLS<br>**US and Mexico Contact: Scott Wooten**<br>Project Manager<br>8211 SciCor Drive

Indianapolis, IN 46221<br>USA<br>~~Phone: +1-317-273-7612~~<br>~~Fax: +1-317-616-2360~~<br>~~E-mail: kellie.estes@covance.com~~<br><br>~~Mexico Contact: Eric Steele~~<br>~~8211 SciCor Drive~~<br>~~Indianapolis, IN 46221~~<br>~~USA~~<br>~~Phone: +1-317-271-1200 x7101~~<br>~~Fax: +1-317-616-2366~~<br>~~E-mail: eric.steele@covance.com~~	Indianapolis, IN 46221<br>USA<br>**Phone: +1-317-273-7628**<br>**Fax: +1-317-616-2360**<br>**E-mail: scott.wooten@covance.com**

Page 15, Responsibilities	Page 16, Responsibilities
~~Luis Armando Bojorquez Guerrero, MD~~<br>Medical Officer Latin America<br>Kendle International<br>~~Via Gustavo Baz 2160 - Caseta 3~~<br>~~Tlalnepantla, Estado de Mexico~~<br>~~CP 54060, Mexico~~<br>~~Phone: (52-55) 2169-0302~~<br>~~Fax: (52-55) 5361-4411~~<br>~~Mobile: (151) 3387 9024~~<br>~~E-mail: bojorquez.luis@kendle.com~~<br><br>~~LOCAL MEDICAL MONITOR (MEXICO) - BACKUP: Isaias Gaona, MD~~<br>~~Medical Officer~~<br>~~Kendle International Periferico Sur 4194, 3er piso~~<br>~~Col. Jardines del Pedregal~~<br>~~Del. Alvaro Obregón~~<br>~~C.P. 01900, Mexico D.F.~~<br>~~Phone: (52-55) 5481-5944~~<br>~~Fax: (52-55) 5135-4768~~<br>~~Mobile: (52-55) 2690-7223~~<br>~~E-mail: gaona.isaias@kendle.com~~	**Leonard Wassermann, MD**<br>Medical Officer Latin America<br>Kendle International<br>**Av. Córdoba 1351 piso 12**<br>**Ciudad de Buenos Aires**<br>**C1055AAD– Argentina**<br>**Phone: +54 114136 8000 x197**<br>**Fax: +54 114136 8016**<br>**Mobile: +54 11 4136 8097**<br>**E-mail: wasserman.leonardo@kendle.com**

Pages 18 and 19 (Abbreviations)	Pages 20 - 22 (Abbreviations)
	**EAS ELISPOT Analysis Set**<br>**PPS Per Protocol Set**<br>**SAS Safety Analysis Set**<br>**SIAE Adverse event of special interest / special interest adverse event**

Page 22 (IV. Protocol Synopsis)	Page 24 (IV. Protocol Synopsis)
Seroconversion is defined as Appearance of	Seroconversion is defined as Appearance of

antibody titers ≥ ~~1:6~~ in a vaccinia specific plaque reduction neutralisation assay	antibody titers ≥ **15** in a vaccinia specific plaque reduction neutralisation assay

Page 60 (7.2.1 Humoral Immunogenicity)	Page 66 (7.2.1 Humoral Immunogenicity)
PRNT<br>Seroconversion is defined as Appearance of antibody titers ≥ ~~1:6~~ in a vaccinia specific plaque reduction neutralisation assay…	PRNT<br>Seroconversion is defined as Appearance of antibody titers ≥ **15** in a vaccinia specific plaque reduction neutralisation assay…

Page 66 (9.2.1 Immunogenicity endpoints)	Page 71 (9.2.1 Immunogenicity endpoints)
PRNT<br>Seroconversion is defined as Appearance of antibody titers ≥ ~~1:6~~ in a vaccinia specific plaque reduction neutralisation assay…	PRNT<br>Seroconversion is defined as Aappearance of antibody titers ≥ **15** in a vaccinia specific plaque reduction neutralisation assay…

Pages 66, 67 (9.3 Cohorts to be evaluated)	Pages 71, 72 (9.3 Cohorts to be evaluated)
Safety set: This is the subset of subjects who received at least one dose of study vaccine and for whom safety data are available.<br><br>The final safety analysis will be performed on this population.<br><br>Full-analysis ~~(FA)~~ ~~s~~et:<br>This is the subset of subjects who had received at least one dose of study vaccine.	Safety set **(SAS)**: This is the subset of subjects who received at least one dose of study vaccine and for whom **any** safety data are available.<br><br>The final safety analysis will be performed on this population.<br><br>Full-analysis **S**et **(FAS):**<br>This is the subset of subjects who had received at least one dose of study vaccine **and for whom any data are available.**

Per Protocol (PP) ~~s~~et:	Per Protocol (PPS) **S**et:
This is the subset of subjects who adhere to all protocol conditions, however, not relevant protocol violators can be included into this dataset.<br><br>The decision whether a protocol deviation is relevant or not for the classification of subjects to subsets should be made case-by case in a blind review meeting.<br><br>The primary population dataset will be the PP ~~set~~. All confirmatory testing is based on this ~~subgroup~~. For further descriptive purposes, the same statistical procedures will be applied to the FA~~ dataset~~.	This is the subset of subjects who adhere to all protocol conditions, however, not relevant protocol violators can be included into this dataset.<br><br>The decision whether a protocol deviation is relevant or not for the classification of subjects to subsets should be made case-by case in a blind review meeting.<br><br>The primary population dataset **for humoral immunogenicity analysis (ELISA and PRNT)** will be the PP**S**. All confirmatory testing is based on this **population**. For further descriptive purposes, the same statistical procedures will be applied to the FA**S**.<br><br>**ELISPOT Analysis Set (EAS):**<br>**This set is defined as the alternative PPS for the ELISPOT data analysis. It consists of all subjects with**<br>• **a complete ELISPOT data set (Visits 1, 2 and 4 available)**<br>• **the administration of both study vaccinations**<br>• **without any relevant major protocol violations for this data set.**<br><br>**ELISPOT data analysis will be descriptive and performed on both the EAS and the FAS.**

Page 67 (9.4 Statistical Analysis)	Page 72/ 73 (9.4 Statistical Analysis)
~~As soon as the last subject has~~ completed ~~Visit 5~~ and after any necessary settlement of queries etc. in the CRFs, data from those patients and visits will be locked.<br>For ~~submission~~ of safety data as part of~~ the~~ Emergency Use Authorization (EUA) data package, a ~~preliminary~~ safety analysis will ~~then~~ be performed on the population described in section 9.3 (‘Study cohorts/data sets to be evaluated‘).~~ These safety data will be reported in an interim safety report.~~<br>~~Once all subjects have completed the half-year follow-up (Visit F-U), a full analysis of the data available will be performed and a final clinical study report prepared.~~	**Once all subjects have** completed **the half-year follow-up (Visit F-U)** and after any necessary settlement of queries etc. in the CRFs, data from all patients and visits will be locked. **A full analysis of the data available will be performed and a final clinical study report prepared. Results of the PRNT assay will be presented in an addendum to the clinical study report.** For **preparation** of safety data as part of **a potential** Emergency Use Authorization (EUA) data package, a safety analysis will be performed on the population (MFAS) described in section 9.3 (‘Study cohorts/data sets to be evaluated‘).

Page 68 (9.4.2. Analysis of Immunogenicity)	Page 73 (9.4.2. Analysis of Immunogenicity)
Descriptive statistics for Enzyme-Linked Immunospot (ELISPOT) will be provided. Summary tables will be provided for the FAS and the ~~PP set~~.	Descriptive statistics for Enzyme-Linked Immunospot (ELISPOT) will be provided. **T cell samples will be analyzed if samples are available for the subject from Visit 1 and at least one of Visit 2 or Visit 4.** Summary tables will be provided for the **EAS** and the FAS.

Page 69 (9.4.3. Analysis of safety and reactogenicity)	Page 74 (9.4.3. Analysis of safety and reactogenicity)
Adverse events of special interest will be separately listed and tabulated. The incidence of such AEs will be compared between treatment groups by means of the exact test of Fisher (or its normal approximation, the chi-squared test).<br><br>*Serious adverse events* will be listed separately. Each SAE will be described individually in detail. The number of subjects with at least one SAE will be compared between treatment groups by means of the exact test of Fisher (or its normal approximation, the chi-squared test).<br><br>~~The occurrence, relationship and intensity of any other cardiac symptom at any time during the study will be listed. The number of subjects with at least one cardiac symptom will be compared between treatment groups by means of the exact test of Fisher (or its normal approximation, the chi-squared test).~~	**The occurrence, relationship and intensity of** *adverse events of special interest* **at any time during the study** will be separately listed and tabulated. The incidence of such AEs will be compared between treatment groups by means of the exact test of Fisher (or its normal approximation, the chi-squared test).<br><br>*Serious adverse events* will be listed separately. Each SAE will be described individually in detail. The number of subjects with at least one SAE will be compared between treatment groups by means of the exact test of Fisher (or its normal approximation, the chi-squared test).

Page 81 (16.4.2 Extension Protocol Flow Chart)

Visit	Visit 5	Telephone F-U
Day (d)	Visit 3 +28-35 d	Visit 3 +182-210 d
Brief interview on AD status[7]		~~x~~

Page 87 (16.4.2 Extension Protocol Flow Chart)

Visit	Visit 5	Telephone F-U
Day (d)	Visit 3 +28-35 d	Visit 3 +182-210 d
~~Brief interview on AD status[7]~~		

Telephone Follow-up for ~~serious adverse events~~		X	Telephone Follow-up for **SAEs, SIAEs and AEs that were still ongoing at visit 5**		X

III. Responsibilities

**STUDY NUMBER**:	POX-MVA-008
**STUDY TITLE**:	A multicenter, open-label, controlled phase II study to evaluate safety and immunogenicity of MVA-BN® (IMVAMUNE®) smallpox vaccine in 18-40 year old subjects with diagnosed atopic dermatitis

COORDINATING INVESTIGATOR:
Prof. Dr. Richard N. Greenberg, M.D.
Professor of Medicine
The Belinda Mason Carden and Paul Mason Professor of HIV/AIDS Research and Education
University of Kentucky School of Medicine
Department of Medicine, Room MN672
800 Rose Street
Lexington, KY 40536-0084, USA
Phone: +1-859-323-6327
Fax: +1-859-323-1631
Pager: +1-859-276-7352
E-mail: rngree01@email.uky.edu

SPONSOR and PRODUCT SUPPLY
Bavarian Nordic A/S
Hejreskovvej 10A
DK-3490 Kvistgård, Denmark
Phone: +45-33-268383
Fax: +45-33-268380

MEDICAL MONITOR:
Nathaly Arndtz-Wiedemann MD
VP Medical Affairs
Bavarian Nordic GmbH
Fraunhoferstrasse 13
82152 Martinsried, Germany
Phone: +49-89-85651308
Fax: +49-89-85651419
E-mail: nathaly.arndtz@bavarian-nordic.com

CLINICAL PROJECT LEADER:
Dorothea von Bredow, PhD
Bavarian Nordic GmbH
Fraunhoferstrasse 13
82152 Martinsried, Germany
Phone: +49-89-85651318
Fax: +49-89-85651333
E-mail: dorothea.bredow@bavarian-nordic.com

CLINICAL DEVELOPMENT MANAGER: Alfred von Krempelhuber, PhD
Bavarian Nordic GmbH
Fraunhoferstrasse 13
82152 Martinsried, Germany
Phone: +49-89-85651369
Fax: +49-89-85651333
E-mail: alfred.krempelhuber@bavarian-nordic.com

STUDY STATISTICIAN(S): Philip Young, PhD
Bavarian Nordic GmbH
Fraunhoferstrasse 13
82152 Martinsried, Germany
Phone: +49-89-85651462
Fax: +49-89-85651333
E-mail: philip.young@bavarian-nordic.com

CRO/PROJECT MANAGER: Emily Graham, MA
Project Leader
Global Clinical Development - North America Kendle
315 E. Eisenhower Pkwy, Suite 214
Ann Arbor, MI 48108
Phone: +1 734 274 8511
Fax: +1 866 552 9983
Email: graham.emilyr@kendle.com

CRO/LEAD MONITOR: **(MEXICO)** Margarita Candela
Kendle Mexico
Periférico Sur # 4194-2 piso
Col. Jardines del Pedregal,
C.P. 01900. México, D.F.
Phone: +52-55-5481-5900 ext 137
Fax: +52-55-5135-4571
E-mail: candela.margarita@kendle.com

CRO/LEAD MONITOR (US): Leslie Pies, MEd
Kendle International
1880 Pacific Place
Lawrenceburg, IN 47025
Phone: +1 812-637-5164
Fax: +1 866-612-5397 / 812-637-0739
E-mail: pies.leslied@kendle.com

LABORATORY: **(SAFETY)** Covance CLS
US and Mexico Contact:
Scott Wooten
Project Manager
8211 SciCor Drive
Indianapolis, IN 46221
USA
Phone: +1-317-273-7628

Fax: +1-317-616-2360
E-mail: scott.wooten@covance.com

LABORATORY (IMMUNOLOGY)

Thomas Meyer, PhD
Bavarian Nordic GmbH
Fraunhoferstrasse 13
82152 Martinsried, Germany
Phone: +49-89-85651405
Fax: +49-89-85651333
E-mail: thomas.meyer@bavarian-nordic.com

Focus Diagnostics, Inc.
Contact: Keith Gottlieb
Scientist
10703 Progress Way
Cypress,
California 90630
USA
Phone: +1-714-220-1900
Fax: +1-714-484-1296
E-mail: kgottlieb@focusdx.com

CENTRALIZED ECG ASSESSMENT:

Kendle UK Ltd.
Contact: Mary Hyman
38, Wellington Business Park
Crowthorne
Berkshire RG45 6LS
United Kingdom
Phone: +44-1344-753046
Fax: +44-1344-760995
E-mail: hyman.mary @kendle.com

CENTRAL CARDIOLOGIST:

Lamin King, MD
28A Cambridge Avenue
Kilburn
London NW6 5BA
England
Phone: +44-7931-584974
E-mail: laminking@yahoo.com

LOCAL MEDICAL MONITOR (MEXICO): Leonard Wassermann, MD
Medical Officer Latin America
Av. Córdoba 1351 piso 12
Ciudad de Buenos Aires
C1055AAD– Argentina
Phone: +54 114136 8000 x197
Fax: +54 114136 8016
Mobile: +54 11 4136 8097
E-mail: wasserman.leonardo@kendle.com

Table of contents

I. GENERAL INFORMATION 2
II. PROTOCOL AMENDMENT #6 5
III. RESPONSIBILITIES 13
IV. PROTOCOL SYNOPSIS 23
V. FLOW CHART 29
VI. SCIENTIFIC SECTION 31
1. INTRODUCTION 31
1.1 Background information 31
1.2 Origin and characteristics of MVA 32
1.3 Summary of Preclinical Data 33
1.4 Summary of Clinical Data 34
1.4.1 Clinical Experience with the MVA precursor vaccine 34
1.4.2 Clinical Experience with MVA-BN® (IMVAMUNE®) 35
1.5 Rationale 40
1.6 Dose, route of administration, vaccination schedule 41
1.7 Benefit/risk assessment 41
2. OBJECTIVES 42
3. STUDY DESIGN 44
3.1 Experimental design 44
3.2 Description of study procedures 44
3.2.1 Screening phase 44
3.2.2 Active study phase 45
3.2.3 Follow-up phase 48
3.2.4 Protocol Extension 48
3.3 Study duration 48
4. SELECTION OF SUBJECTS 49
4.1 Recruitment procedure 49
4.2 Inclusion criteria 50
4.3 Exclusion criteria 51
5. STUDY HALTING/TERMINATION AND WITHDRAWAL OF SUBJECTS 53

5.1 Study halting and termination rule **53**

5.2 Reporting of events fulfilling the study halting criteria **53**

5.3 Data Safety Monitoring Board (DSMB) **53**

5.4 Individual withdrawal criteria during the study **53**

5.5 Subject withdrawal procedure **54**

5.6 Contraindications and precautions for further study vaccinations **54**

6. STUDY TREATMENT **55**

6.1 Investigational product **55**

6.2 Packaging and labelling **55**

6.3 Vaccine storage, handling and dispensing **56**

6.4 Dose, vaccination schedule and route of administration **56**

6.5 Accountability and disposal **56**

6.6 Concomitant medication **57**

7. CLINICAL AND LABORATORY ASSESSMENTS **58**

7.1 Assessment of safety and reactogenicity **58**

7.1.1 Medical history 58

7.1.2 Physical examination and vital signs 58

7.1.3 Laboratory measurements 58

7.1.4 ECG assessments 60

7.1.5 Assessment of solicited adverse events (Diary) 61

7.1.6 Assessment of unsolicited adverse events 62

7.1.7 Assessment of intensity for adverse events 63

7.1.8 Assessment of causality for adverse events 64

7.1.9 Algorithm for assessment of cardiac events 65

7.2 Assessment of immunogenicity **66**

7.2.1 Humoral immunogenicity 66

7.2.2 Cellular immunogenicity 66

8. ADVERSE EVENT DEFINITIONS AND REPORTING **67**

8.1 Definition of adverse event **67**

8.2 Definition of solicited adverse events **67**

8.3 Definition of unsolicited adverse events **67**

8.4 Definition of “adverse event of special interest” **67**

8.5 Definition of serious adverse events (SAEs) **68**

8.6 Reporting of SAEs and adverse events of special interest **68**

9. STATISTICAL CONSIDERATIONS **70**

9.1 Sample size calculation ... 70
9.2 Endpoints ... 70
9.2.1 Immunogenicity endpoints ... 70
9.2.2 Safety and reactogenicity endpoints ... 71
9.3 Study cohorts/data sets to be evaluated ... 71
9.4 Statistical analysis ... 72
9.4.1 Analysis of demographics and baseline characteristics ... 73
9.4.2 Analysis of Immunogenicity ... 73
9.4.3 Analysis of safety and reactogenicity ... 73
9.4.4 Data handling ... 75
10. ETHICAL ASPECTS ... 76
10.1 Ethical and legal regulations ... 76
10.2 Approval of an Independent Ethics Committee (IEC) / Institutional Review Board (IRB) 76
10.3 Confidentiality and data protection ... 76
11. INFORMED CONSENT ... 77
12. CASE REPORT FORMS AND RETENTION OF RECORDS ... 77
12.1 Case report forms ... 77
12.2 Retention of records ... 77
13. MONITORING OF THE STUDY ... 78
14. RESPONSIBILITIES OF THE INVESTIGATOR ... 78
15. REFERENCES ... 80
16. APPENDICES ... 82
16.1 Appendix I: Toxicity Scale for laboratory values ... 82
16.2 Appendix II: Case Definitions Acute Myocarditis / Pericarditis ... 84
16.3 Appendix III: SCORAD Evaluation Form ... 85
16.4 Appendix IV: Proposed Extension Phase of the POX-MVA-008 trial ... 86
16.4.1 General Information and Rationale for an Extension Phase of the POX-MVA-008 trial ... 86
16.4.2 Extension Protocol Flow Chart ... 87
16.4.3 Inclusion & Exclusion Criteria ... 88
16.4.4 Statistical Considerations ... 88
16.5 Appendix V: Exchange of IMVAMUNE® vaccine lot 0170505 by IMVAMUNE® vaccine lot 0040707 ... 89

List of abbreviations

ACIP	Advisory Committee on Immunization Practices
AD	Atopic Dermatitis
ADR	Adverse Drug Reaction
AE	Adverse Event
AIDS	Acquired Immunodeficiency Syndrome
ALT	Alanine Aminotransferase
ANC	Absolute Neutrophil Count
AST	Aspartate Aminotransferase
ATP	According to Protocol
CBC	Complete Blood Count
CD	Cluster of Differentiation
CEF	Chicken Embryo Fibroblast
CI	Confidence Interval
CRF(s)	Case Report Form(s)
CRO	Contract Research Organization
CTL	Cytotoxic T-Lymphocyte
CVA	Chorioallantois Vaccinia Virus Ankara
DHHS	United States Department of Health and Human Services
DMID	Division of Microbiology and Infectious Diseases,<br>National Institute of Allergy and Infectious Diseases
DNA	Deoxyribonucleic acid
DSMB	Data Safety Monitoring Board
EAS	ELISPOT Analysis Set
ECG	Electrocardiogram
ELISA	Enzyme-linked Immunosorbent Assay
ELISPOT	Enzyme-Linked Immunospot
EUA	Emergency Use Authorization
FAS	Full Analysis Set
F-U	Follow-up
GCP	Good Clinical Practice
GMT	Geometric Mean Titer
HCG	Human Choriogonadotropin
HIV	Human Immunodeficiency Virus

ICH	International Conference of Harmonization of Technical Requirements for Registration of Pharmaceuticals for Human Use
ICS	Intracellular Cytokine Staining
i.d.	Intra-dermal
IEC	Independent Ethical Committee
IFN	Interferon
IgG	Immunoglobulin G
i.m.	Intra-muscular
i.n.	Intra-nasal
i.p.	Intra-peritoneal
IRB	Institutional Review Board
i.t.	Intra-tracheal
LL	Lower Limit
LLN	Lower Limit of Normal
MedDRA	Medical Dictionary for Regulatory Activities
MFAS	Modified Full Analysis Set
MVA	Modified Vaccinia Ankara Strain
MVA-BN®	Modified Vaccinia Ankara – Bavarian Nordic
n/N	Number
NCI	National Cancer Institute
NYCBOH	New York City Board of Health
PBMC	Peripheral Blood Mononuclear Cells
p.o.	Per-oral
PP	Per Protocol
PPS	Per Protocol Set
PRNT	Plaque Reduction Neutralization Test
PVC	Premature Ventricular Contractions
RBC	Red Blood Cells
SAE	Serious Adverse Event
SAS	Safety Analysis Set
s.c.	Subcutaneous
SCORAD	Scoring Atopic Dermatitis
SCR	Screening
SFU	Spot Forming Unit
SGOT	Serum Glutamic Oxaloacetic Transaminase

SGPT	Serum Glutamic Pyruvic Transaminase
SIAE	Adverse event of special interest / special interest adverse event
ST	ST Segment in Electrocardiogram
$TCID_{50}$	Tissue Culture Infectious Dose 50
UL	Upper Limit
ULN	Upper Limit of Normal
V	Visit
VV	Vaccinia Virus
WBC	White Blood Cell Count
WHO	World Health Organization

IV. Protocol Synopsis

Title	A multicenter, open-label, controlled phase II study to evaluate immunogenicity and safety of MVA-BN® (IMVAMUNE®) smallpox vaccine in 18-40 year old subjects with diagnosed atopic dermatitis
Clinical phase	Phase II
Sponsor	Bavarian Nordic A/S<br>Hejreskovvej 10A<br>DK-3490 Kvistgård, Denmark
Coordinating Investigator	Prof. Dr. Richard N. Greenberg<br>University of Kentucky, Lexington, KY, USA
Vaccination dose and schedule	One 0.5 ml dose MVA-BN® (IMVAMUNE®) liquid-frozen contains 1 x 10^8 $TCID_{50}$ Modified Vaccinia virus Ankara. Two vaccinations of 0.5 ml MVA-BN® (IMVAMUNE®) vaccine will be given according to schedule 0 – 4 weeks.
Route of administration	Each immunization consists of one subcutaneous (s.c.) injection in the non-dominant upper arm.
Study duration	Up to 39 weeks for each subject
Sample size	The total sample size will be 260 (130 per group).<br>In an extension phase, 100 additional healthy subjects and 200 additional subjects with diagnosed atopic dermatitis (Group 2) will be enrolled.
Study design	Open-label, controlled, 2 study groups:<br>Group 1: Healthy, vaccinia naïve subjects without atopic disease (n = 130)<br>Group 2: Vaccinia naïve subjects with diagnosed atopic dermatitis (n = 130)

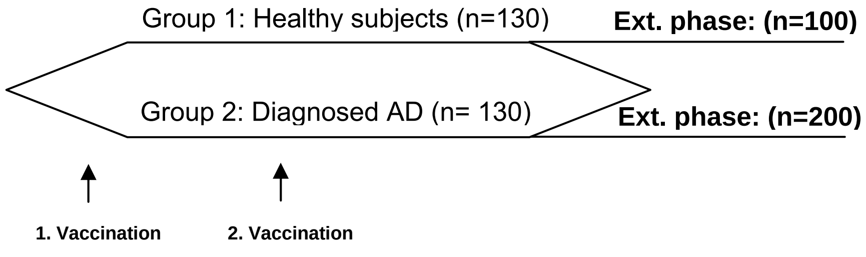

Visit Days						
-28- -1	0	7 - 10	28 – 35	V3 + 10-14	V3 + 28-35	V3 + 182–210
Scr	V1	V2	V3	V4	V5	F-U

1.1.1.1 After enrollment of 130 subjects each in Group 1 and Group

	**2, the enrollment into the two study groups will be extended by another 100 vaccinia naïve healthy subjects and 200 vaccinia naïve subjects with 1.1.1.2 diagnosed atopic dermatitis. Please refer to Appendix IV for details of the protocol extension.**
Primary objectives	To assess the humoral immune response (measured in ELISA) induced to MVA-BN® (IMVAMUNE®) in subjects with diagnosed atopic dermatitis compared to healthy subjects.
Secondary objectives	• To assess safety of MVA-BN® (IMVAMUNE®) in subjects with atopic dermatitis compared to healthy subjects.<br>• To assess reactogenicity of MVA-BN® (IMVAMUNE®) in subjects with atopic dermatitis compared to healthy subjects.<br>• To assess the immune responses measured with the plaque reduction neutralization test (PRNT) and Enzyme-Linked Immunospot (ELISPOT) induced to MVA-BN® (IMVAMUNE®) in subjects with diagnosed atopic dermatitis compared to healthy subjects. ELISPOT: Subgroup of 50 subjects from Group 1 (healthy) and 110 subjects from Group 2 (AD) with samples taken at each of the Visits 1, 2 and 4 collected at US sites. The first subjects enrolled into the study will be included in the subgroup until the numbers specified above are reached.
Primary endpoint	• ELISA specific seroconversion rate at visit 4<br>*Seroconversion is defined as the appearance of antibody titers $\geq$ 1:50 in a vaccinia specific IgG ELISA for initially seronegative subjects or twofold increase of the antibody titer compared to the pre-existing baseline titer for subjects with a pre-existing antibody titer in the ELISA.*
Secondary endpoints	• ELISA specific seroconversion rate at visits 2, 3 and 5 and F-U.<br>• Geometric mean titers (at all blood sampling time points).<br>o *The geometric mean titer (GMT) is calculated by taking the antilogarithm of the mean of the log10 titer transformations. Antibody titers below the cut-off of the assay will be given an arbitrary value of one for the purpose of calculation.*<br>• Neutralization assay specific seroconversion rates and geometric mean titers (at all blood sampling time points).<br>o *Seroconversion is defined as Appearance of antibody titers $\geq$ 15 in a vaccinia specific plaque reduction neutralisation assay for initially seronegative subjects or twofold increase of the antibody titer compared to the pre-existing baseline titer for subjects with a pre-existing antibody titer in the PRNT.*<br>o *The GMT is calculated by taking the antilogarithm of the mean of the log10 titer transformations. Antibody titers below the cut-off of the assay will be given an arbitrary value of one for the purpose of calculation.*<br>• Subgroup of 50 subjects from Group 1 (healthy) and 110 subjects from Group 2 (AD) collected at US sites: IFN gamma producing T-cells collected at Visits 1, 2 and 4 in response to stimulation with MVA-BN® (IMVAMUNE®) detected by Enzyme-Linked Immunospot (ELISPOT). The percentage of IFN gamma positive cells in T-cell populations compared to non-stimulated cells will be reported.

- Occurrence of any grade 3 adverse event probably, possibly or definitely related to the study vaccine within 28 days after vaccination.
- Occurrence, relationship and intensity of any serious adverse event at any time during the study.
- Occurrence of solicited local adverse events (erythema, swelling and pain) within 1 week after each vaccination (Days 0-7): Intensity and duration of local adverse events.
- Occurence of solicited systemic adverse events (pyrexia, headache, myalgia, nausea, fatigue and chills) within 1 week after each vaccination (Days 0-7): Intensity and duration of systemic adverse events.
- Occurrence of unsolicited non-serious adverse events within 4 weeks after each vaccination (Days 0-28): Intensity, duration and relationship to vaccination.

Inclusion criteria

Group 1:

1. Subjects without present or history of any kind of atopy. Subjects with seasonal allergies can only be enrolled if an association with other atopic disorders such as allergic rhinitis and asthma can be excluded.

Group 2:

2. Subjects with diagnosed atopic dermatitis. “Diagnosed” atopic dermatitis includes subjects with either documented history of or subjects with currently active (defined as SCORAD ≤ 30, Kunz et al., 1997) atopic dermatitis. Currently active atopic dermatitis includes relapsing forms of atopic dermatitis, also when subjects are enrolled during inflammation-free intervals.

All study subjects:

3. Male and female subjects between 18 and 40 years of age without history of smallpox vaccination.
4. Women must have a negative serum pregnancy test at screening and a negative urine pregnancy test within 24 hours prior to vaccination.
5. Women of childbearing potential must have used an acceptable method of contraception for 30 days prior to the first vaccination, must agree to use an acceptable method of contraception during the study, and must not become pregnant for at least 28 days after the last vaccination. A woman is considered of childbearing potential unless post-menopausal or surgically sterilized. (Acceptable contraception methods are restricted to abstinence, barrier contraceptives, intrauterine contraceptive devices or licensed hormonal products.)
6. Read, signed and dated informed consent document after being advised of the risks and benefits of the study in a language understood by the subject signed, and prior to performance of any study specific procedure.
7. Troponin I < 2 fold ULN.

8. White blood cells ≥ 2500/mm^3 and < 11,000/mm^3.
9. Absolute neutrophil count (ANC) within normal limits
10. Negative urine glucose by dipstick or urinalysis.
11. Hemoglobin within normal limits.
12. Platelets within normal limits.
13. Adequate renal function defined as:
    a. Serum creatinine without clinically significant findings
    b. Urine protein < 30 mg/dL or none or trace proteinuria (by urinalysis or dip stick).
14. Adequate hepatic function defined as:
    a. Total bilirubin ≤ 1.5 x ULN in the absence of other evidence of significant liver disease
    b. AST (SGOT), ALT (SGPT) and alkaline phosphatase without clinically significant findings
15. Electrocardiogram (ECG) without clinically significant findings (e.g. any kind of atrioventricular or intraventricular conditions or blocks such as complete left or right bundle branch block, AV-node block, QTc or PR prolongation, premature atrial contractions or other atrial arrhythmia, sustained ventricular arrhythmia, 2 premature ventricularcontractions (PVC) in a row, ST elevation consistent with ischemia).
16. Availability for follow-up for the planned duration of the study (26 weeks after last vaccination).

Exclusion criteria

1. Pregnant or breast-feeding women.
2. Uncontrolled serious infection i.e. not responding to antimicrobial therapy.
3. History of any serious medical condition, which in the opinion of the Investigator would compromise the safety of the subject.
4. History of or active autoimmune disease. Persons with vitiligo or thyroid disease taking thyroid replacement are not excluded.
5. Known or suspected impairment of immunologic function including, but not limited to, clinically significant liver disease; diabetes mellitus; moderate to severe kidney impairment.
6. History of malignancy, other than squamous cell or basal cell skin cancer, unless there has been surgical excision that is considered to have achieved cure. Subjects with history of skin cancer at the vaccination site are excluded.
7. History or clinical manifestation of clinically significant and severe hematological, renal, hepatic, pulmonary, central nervous, cardiovascular or gastrointestinal disorders.
8. Clinically significant mental disorder not adequately controlled by medical treatment.
9. Any condition which might interfere with study objectives or would limit the subject’s ability to complete the study or to be compliant in the opinion of the Investigator.
10. History of coronary heart disease, myocardial infarction, angina, congestive heart failure, cardiomyopathy, stroke or transient ischemic attack, uncontrolled high blood pressure.
11. History of an immediate family member (father, mother, brother, or sister) who has had onset of ischemic heart disease before age 50

years.

12. Ten percent or greater risk of developing a myocardial infarction or coronary death within the next 10 years using the National Cholesterol Education Program's risk assessment tool: (http://hp2010.nhlbihin.net/atpiii/calculator.asp?usertype=prof) NOTE: This criterion applies only to volunteers 20 years of age and older.
13. History of intravenous drug abuse.
14. History of allergic disease or reactions likely to be exacerbated by any component of the vaccine.
15. Known allergy to egg or aminoglycoside (gentamicin).
16. History of anaphylaxis or severe allergic reaction.
17. Acute disease (illness with or without a fever) at the time of enrollment.
18. Temperature ≥100.4°F (≥ 38.0°C) at the time of enrollment.
19. Subjects undergoing treatment for tuberculosis infection or disease.
20. Having received any vaccinations or planned vaccinations with a live vaccine within 30 days prior or after study vaccination.
21. Having received any vaccinations or planned vaccinations with a killed vaccine within14 days prior or after study vaccination.
22. Chronic administration (defined as more than 14 days) of systemic immuno-suppressants during a period starting from six months prior to administration of the vaccine and ending at study conclusion. Subjects receiving chronic treatment (defined as more than 14 days) with topical calcineurine inhibitors (e.g. tacrolimus, pimecrolimus) on the body or > 25% of the facial area are excluded. A wash-out period of ≥ 14 days for tacrolimus and ≥ 28 days for pimecrolimus can be considered sufficient before enrollment into the study.
    Subjects receiving chronic treatment (defined as more than 14 days) with high dose topical cortisone (> 30 grams/week of an intermediate potency [class IV] topical steroid [or equivalent], the treated area must not exceed 10% of the total body surface) are excluded.
23. Post organ transplant subjects whether or not receiving chronic immunosuppressive therapy.
24. Administration or planned administration of immunoglobulins and/or any blood products during a period starting from 3 months prior to administration of the vaccine and ending at study conclusion.
25. Use of any investigational or non-registered drug or vaccine other than the study vaccine within 30 days preceding the first dose of the study vaccine, or planned administration of such a drug during the study period.
26. Study personnel.

Statistical Considerations	The sample size calculation is based on the primary immunogenicity endpoint '**MVA-specific seroconversion rate'**.<br>o *Seroconversion is defined as the appearance of antibody titers ≥ 1:50 in a vaccinia specific IgG ELISA for initially seronegative subjects or twofold increase of the antibody titer compared to the pre-existing baseline titer for*

subjects with a pre-existing antibody titer in the ELISA.

The primary hypothesis is to show that the humoral immune response of the group with diagnosed atopic dermatitis (Group 2) is not statistically inferior compared to the group with healthy subjects (Group 1). The study should demonstrate that the Group 2 seroconversion rate is not worse than the Group 1 seroconversion rate by more than a pre-specified amount. This amount is called the non-inferiority margin (Δ).

Suppose p1 is the seroconversion rate in healthy subjects (group 1) and p2 is the seroconversion rate in the subjects with atopic dermatitis (group 2).
The test on non-inferiority will be applied for the following hypothesis:

$$H0: \quad p2 - p1 \leq -\Delta \quad \text{versus } H1: \quad p2 - p1 > -\Delta, \text{ where}$$

Δ is the non-inferiority margin and is chosen in this trial as 5%.
From the experience in the pilot trial POX-MVA-007 in subjects with atopic dermatitis subjects, it is anticipated that the seroconversion rate in both groups reaches at least 99%.

Assuming a significance level of 5%, a power of >80%, expected seroconversion rates of 99% in both groups, this yields to a sample size of 124 per group (248 in total). In order to account for about 5% drop out rate, a total of 130 subjects per group (260 in total) will be treated.
For the statistical consideration of the protocol extension, please refer to Appendix IV.

V. Flow Chart

VISIT[1]	SCREENING	VISIT 1	VISIT 2	VISIT 3	VISIT 4	VISIT 5	VISIT F-U
Day (D)	-28- -1 D	0	7-10 D	28-35 D	VISIT 3 +10-14 D	VISIT 3 +28-35 D	VISIT 3 +182-210 D
Vaccination		I		II			
Informed consent[2]	X						
Screening examinations	X						
Check incl. / excl. criteria, esp. cardiac risk	X	X		X			
Medical History (incl. history of AD)	X						
Interview on history of AD[8]	X						
Examination of AD including SCORAD[8] (active AD only)	X						
Brief interview on AD status[8] (including SCORAD for active AD only)						X	
Brief interview on AD status[8]							X
Complete Physical examination incl. evaluation of vital signs, esp. listening to heart and lung	X						
Targeted physical exam.		X	X	X	X	X	X
ECG	X		X		X	(X)[4]	(X)[4]
Pregnancy test[3]	X	X		X		X	
Safety Lab	X		X		X	(X)[4]	(X)[4]
Urine analysis	X						
Antibody analysis		X	X	X	X	X	X
T-Cell analysis (subgroup of 50 Gr.1 and 110 Gr. 2 subjects, US sites only)		X	X		X		
Handout of diary card[5]		X		X			
Collection of diary card			X		X		
Exam of vaccination site			X		X		
AE-reporting		X	X	X	X	X	X[6]
Review baseline signs and symptoms	X	X					
Blood sampling in ml[7]	*14*	*16*	*30*	*16*	*30*	*16 (+14)*[4]	*16 (+14)*[4]
Approx. blood sampling in ml[7] including T cell analysis (subgroup)	*14*	*80*	*94*	*16*	*94*	*16 (+14)*[4]	*16 (+14)*[4]

[1] If clinically indicated additional visits may be necessary between scheduled visits and possibly additional blood draws may occur. Visits occurring between indicated scheduled visits will be identified with letters to indicate sequence of the visit until the next scheduled visit occurs (e.g. 1A, 1B etc. will indicate extra visits between visit 1 and 2).
[2] Consent process must be completed and form signed before any study-related procedures are conducted.
[3] Serum or urine pregnancy test, women of childbearing potential only. At screening visit, a serum test must be performed.
[4] Only required if clinically indicated.
[5] The Diary Card should be completed daily for 7 days. If symptoms persist at day 7, temperature/symptom measurements should be recorded each day until resolved.
[6] Serious adverse events only.

[7] Approx amount of blood needed for analysis: Safety lab: app.14 ml, Antibody analysis: app. 16 ml, T-Cell analysis (subgroup): app. 64 ml. Amount of blood taken during the study: app. 166 ml (subgroup up to 358 ml)
[8] Group 2 (AD) only

VI. Scientific Section

1. Introduction

1.1 Background information

In Germany during the period from 1960 to 1974, Professor Anton Mayr succeeded in attenuating the dermal vaccinia strain Ankara (CVA) by over 500 continuous passages in primary chicken embryo fibroblast cultures (CEF). The rationale was to attenuate the smallpox vaccine so that the complications associated with vaccination could be reduced or completely eliminated and make safer parenteral vaccination possible. This highly attenuated virus of CVA was named MVA (modified vaccinia Ankara). It was demonstrated that it had retained its original immunogenicity and its protective effect against variola and general Orthopox infections and offers a greatly diminished virulence for humans and animals combined with the loss of contagiousness. It was shown that vaccination-induced disease does not occur in newborn animals (mice, rabbits, and chickens) and immunosuppressed vaccinated animals (by radiation, iatrogenic immune suppression). Local (cutaneous, oral, and intranasal) and parenteral (subcutaneous, intramuscular) administration of MVA are both possible, safe and retain immunizing efficacy (Mayr & Munz 1964; Stickl *et al.* 1974; Stickl, Hochstein-Mintzel, Mayr, Huber, Schafer, & Holzner 1974; Mayr *et al.* 1975).

In Germany, the MVA strain was officially authorized for two-stage parenteral smallpox vaccination in children in 1976. It was used in approximately 120,000 Caucasian individuals with no reported side effects, even though many of the subjects were among the population with high risk of developing complications (Mayr *et al.* 1978).

In May 1980, the World Health Organization (WHO) declared the global eradication of smallpox. Since 1982, smallpox vaccination has not been required for international travelers' vaccination and International Certificates of Vaccination no longer include smallpox. As smallpox vaccination was deleted from the vaccination schedules, production of the vaccine was no longer required.

Despite the fact that the WHO officially declared smallpox eradicated in 1980, a new threat exists from the use of variola in biological warfare and/or bio-terrorism. Indeed, as mass vaccination programs halted more than 20 years ago, most people have no existing immunity to smallpox, and as such the release of this highly contagious virus would have devastating effects. As a consequence, there is an urgent need by many Governments for a safe efficacious vaccine to protect the public against smallpox.

The original smallpox vaccines were based on a number of different vaccinia strains e.g. Lister-Elstree strain recommended by the World Health Organization (WHO) and used in Germany or the New York City Board of Health (NYCBOH) strain used in the United States. While these proved to be highly effective immunizing agents they also showed considerable side effects. Besides from local reactions with scab development and scarring, general symptoms observed frequently after smallpox vaccination have been fever, weakness, muscular pain, headache, swelling and soreness of local lymph nodes and rashes. Fever occurred in the majority of vaccinees, especially in small children: up to 70% got temperatures of 38°C, 15-20% showed even higher rates. Apart from less dramatic and transient side effects like erythematous or urticarial rashes, severe and potentially fatal cutaneous complications of vaccinia vaccination

include eczema vaccinatum and progressive vaccinia. Most feared are complications of the central nervous system, especially post-vaccinal encephalitis, followed in 15-25% by death and in 25% by neurologic sequelae (Goldstein *et al.* 1975;Lane *et al.* 1969;Lane *et al.* 1970). In Germany, the occurrence of neurological complications in primary vaccinees was reported in 1:20,000 to 30,000 vaccinees and while other countries such as the United States excluded high-risk individuals from vaccination, an average of seven persons a year still died from complications due to smallpox vaccination (McElwain 1972). Another consideration for the discontinued use of the classical smallpox vaccine is the world prevalence of HIV and AIDS. The classical replication competent smallpox vaccine could be lethal if given to immune compromised individuals. A study published in 1991 (Guillaume *et al.* 1991) reported the case of two HIV infected individuals that received an experimental immunotherapy in the form of paraformaldehyde fixed autologous T cells previously infected with recombinant vaccinia viruses. Both these patients were immune compromised and experienced necrotic skin lesions due to generalised vaccinia infections that led to death. However, complications can also occur in HIV infected individuals that have a good T cell count and appear healthy following vaccinations with vaccinia (Redfield *et al.* 1987) demonstrating the severe implications of a wide spread use of vaccinia based vaccines.

Bavarian Nordic's (BN's) proprietary Modified Vaccinia Ankara vector (MVA-BN®) is a live attenuated pox virus derived from Anton Mayr's seed virus used to vaccinate more than 120,000 people in Germany during the smallpox vaccination program in the late seventies. This vaccine possesses a number of characteristics that makes it highly suitable for prophylactic and therapeutic vaccination. The vaccine has a superior safety record in comparison to other viruses. The vaccine's immunogenic potential has been demonstrated in animal studies and is known from data in humans vaccinated during the two-step vaccination program in Germany (Mayr et al. 1978).

BN has established a state of the art current Good Manufacturing Practice (cGMP) rated production process for MVA-BN® (IMVAMUNE®) as well as for MVA-BN® derived recombinant viruses.

1.2 Origin and characteristics of MVA

Vaccinia virus (VV) is considered the best known member of the poxvirus family and the prototype live viral vaccine. VV replicates in the cytoplasm of the host cell, its DNA does not integrate into the host cell genome and it is non-oncogenic.

MVA was derived from the VV strain Ankara by 574 serial passages in CEF. From passage 530 the virus is named MVA and has a stabilized immunogenicity, extremely reduced transmission and no induction of a carrier status. During passaging, MVA has suffered a multitude of mutations within its genome and six major deletions resulting in the loss of 15% (30kbp) of original genetic information (Antoine *et al.* 1998). The deletions affect a number of virulence and host range genes as well as the gene for the Type A inclusion bodies (Antoine, Scheiflinger, Dorner, & Falkner 1998; Rosel *et al.* 1986; Meyer *et al.* 1991). As a consequence, MVA exhibits a severely restricted host range, and replicates only very poorly, if at all, in most mammalian cell types, including primary human cells and most transformed human cell lines (Sutter & Moss 1992; Carroll & Moss 1997; Blanchard *et al.* 1998; Drexler *et al.* 1998).

Although MVA exhibits a strongly attenuated replication in these cell types its genes are efficiently transcribed, with the block in viral replication being at the level of virus assembly and

egress (Sutter & Moss 1992; Carroll & Moss 1997). Genetic reconstitution of one of the deletions in the MVA genome affecting, besides other genes, the VV host range gene K1L restored MVA replication in some mammalian cell lines, but failed to restore the growth capacity of MVA in human cells (Meyer et al. 1991; Carroll & Moss 1997).

Taken together with the loss of 15% of its genome, MVA is therefore unlikely to be able to spontaneously regain its replication competency following injection into humans. Despite its high attenuation and reduced virulence, MVA has retained its stable immunogenic properties and variola-protective effect.

MVA is a potent inducer of type I interferon (IFN) in human cells and it expresses a soluble interleukin-1 receptor (Blanchard et al. 1998), which has been implicated as an anti-virulence factor for certain poxviruses (Alcami & Smith 1992; Alcami & Smith 1996). In contrast to VV, MVA does not express soluble receptors for IFN γ, IFN α/β, tumor necrosis factor and CC chemokines (Antoine et al. 1998; Blanchard et al. 1998). MVA is known to have protective immunogenic potential against any kind of orthopox virus.

Taken together with its restricted host range these factors may explain the avirulent phenotype observed for MVA *in vivo* in a wide variety of mammalian species including humans (see below). As a consequence, MVA was used in more than 120,000 human subjects as part of a two-step vaccination protocol, combined with conventional vaccinia virus, against smallpox in Germany (Mayr et al. 1978).

MVA-BN® (IMVAMUNE®) is a highly attenuated, purified live vaccine produced under serum-free conditions in CEF cells. Extensive in-house studies have demonstrated that MVA-BN® (IMVAMUNE®) has superior characteristics compared to other MVA strains, which include:

- MVA-BN® (IMVAMUNE®) has a superior attenuation compared to other MVA strains that replicate in various human cells.
- MVA-BN® (IMVAMUNE®) vaccinations are safe in severely immune compromised animals, whereas other MVA strains replicate resulting in pathology and death.
- MVA-BN® (IMVAMUNE®) has a stable genotype, whereas other MVA strains rapidly lose their attenuation when cultured on mammalian cells.

Therefore, it can be concluded that MVA-BN® (IMVAMUNE®) is a promising safe human smallpox vaccine candidate.

1.3 Summary of Preclinical Data

Bavarian Nordic has performed an extensive preclinical development program for MVA-BN® (IMVAMUNE®) that has demonstrated the safety, efficacy and bio-equivalence of MVA-BN® (IMVAMUNE®) compared to other traditional smallpox vaccines (e.g. Dryvax® and MVA-571). Bavarian Nordic has also developed challenge models including an intratracheal (i.t.) monkeypox challenge model and two murine orthopox challenge models. Some key features include:

- MVA-BN® (IMVAMUNE®) has a superior attenuation compared to other MVA strains that replicate in various human cells.
- MVA-BN® (IMVAMUNE®) vaccinations are safe in rabbits with minimal and (in case of subcutaneous administration: “potentially”) reversible effects.

- Administration of MVA-BN® (IMVAMUNE®) resulted in the detection of vaccine transcripts in distinct tissues with clearance of those transcripts within 7 days (with only the injection site skin and muscle being positive to a low frequency)
- MVA-BN® (IMVAMUNE®) vaccinations are safe in severely immune compromised animals, whereas other MVA strains replicate resulting in pathology and death.
- MVA-BN® (IMVAMUNE®) demonstrated a superior efficacy in a lethal vaccinia virus challenge model in mice compared to other MVA strains.
- MVA-BN® (IMVAMUNE®) vaccinations induce the same efficacy as traditional smallpox vaccine (e.g. DryVax®) against a lethal orthopox challenge given via various routes (intraperitoneal [i.p.] and intranasal [i.n.]).
- A single vaccination with MVA-BN® (IMVAMUNE®) induces equivalent protection in mice following intranasal challenge with high lethal doses of Ectromelia virus compared to a traditional smallpox vaccine (Dryvax®)
- MVA-BN® (IMVAMUNE®) vaccinated mice are protected from a lethal vaccinia virus challenge applied within 4 days, whereas Dryvax® vaccinated mice are not protected.
- MVA-BN® (IMVAMUNE®) is a potent inducer of both antibody and T cells responses and induces equivalent levels of immunity as traditional smallpox vaccines (Dryvax® and Elstree) in both murine and non-human primates.
- BN has developed the first i.t. monkexpox challenge model that more closely mimics the natural route of a smallpox infection and results in a smallpox-like disease
- Non-human primates vaccinated with MVA-BN® (IMVAMUNE®) are protected from a lethal i.t. monkeypox challenge demonstrating an equivalent protection to traditional smallpox vaccines (Dryvax®).
- BN is in the process of validating the monkeypox and selected murine orthopox virus challenge models according to FDA approved protocols.

For more detailed information on preclinical data please refer to the respective section of the Investigator Brochure.

1.4 Summary of Clinical Data

1.4.1 Clinical Experience with the MVA precursor vaccine

Non-recombinant MVA has been extensively used in humans, forming part of a two-step smallpox vaccination program in combination with wild-type vaccinia virus in Germany in 1978. More than 120,000 human subjects received i.d. and s.c. injections with MVA as part of this program, with the majority of subjects being children and adults at risk for adverse reaction from the wild-type vaccine without prior immunization with an avirulent MVA (Mahnel & Mayr 1994; Mayr, Stickl, Muller, Danner, & Singer 1978; Stickl, Hochstein-Mintzel, Mayr, Huber, Schafer, & Holzner 1974; Stickl & Hochstein-Mintzel 1971). Mild local reactions including local reddening of the skin and infiltration were observed at the site of injection in ~75% of individuals vaccinated by the intradermal route (Mayr et al. 1978). Side effects on the injection site after MVA vaccination seen in 5308 vaccinia-naive individuals (0.2 ml intradermally in the forearm) are shown in table I.

Table I: Local reactogenicity 24-48 hours and 5-7 days after vaccination with MVA

LOCAL REACTION	24-48 HOURS	5-7 DAYS
No reaction	7.25%	9.17%
Reddening up to 10 mm in diameter	54.9%	75,45%

Reddening 10-20 mm in diameter	31.45%	14,05%
Reddening more than 20 mm in diameter	6.40%	1.53 %

No blistering, pustula or ulceration were observed at virus doses that produce such reactions in approximately 50% of naive individuals after application of conventional vaccinia. This indicated that the virulence of MVA for skin is very low. No encephalitic reaction was observed (Stickl et al. 1974).

Approximately 2-4% of the subjects (n = 7098) had slight fever and felt unwell (fever > 38°C in 2.28%, non-specific systemic symptoms in 4.11%), but the symptoms were much less than those observed with the normal smallpox vaccine. No serious adverse events were reported. In addition, vaccination with MVA before administering the normal smallpox vaccine (replication competent wild-type vaccinia virus) resulted in a reduced number of side effects from this administration and the development of smaller wild-type vaccinia-derived pocks.

Single dose vaccination with MVA elicited only weak hemagglutination inhibiting or virus neutralizing antibody response. However, after vaccination with wild-type vaccinia virus vaccinees presented with a strong booster effect, indicating the priming by MVA with specific humoral and cellular immune response (Mayr et al. 1978). In vaccinees having received a second dose of MVA, an increased level of antibody response was observed as well. It could be shown in animal experiments that protection against variola was provided even in the absence of an antibody response (Stickl et al. 1974).

1.4.2 Clinical Experience with MVA-BN® (IMVAMUNE®)

To date, 10 clinical studies using IMVAMUNE® have been initiated and more than 1800 subjects were vaccinated. The populations vaccinated include > 1200 healthy subjects as well as risk groups for which conventional smallpox vaccines are contraindicated such as > 500 HIV infected patients and > 100 patients with atopic dermatitis.

Vaccination of healthy population with MVA-BN® (IMVAMUNE®)

Study POX-MVA-001: 86 healthy volunteers were vaccinated in this clinical phase I trial conducted in Europe. The subjects were healthy males, stratified based on the presence or absence of a prior history of smallpox vaccination. Primary objective was to assess safety and tolerability of MVA-BN® (IMVAMUNE®) with secondary objectives comparing various routes of administration (subcutaneous [s.c.] versus intramuscular [i.m.]) with three different doses of vaccine being tested. There were five cohorts, with three cohorts receiving the vaccine s.c. at a dose of 10^6, 10^7, and 10^8 tissue culture infectious dose 50 ($TCID_{50}$), respectively. A fourth cohort received the vaccine i.m. at a dose of 10^8 $TCID_{50}$. All four cohorts were naïve to smallpox vaccination and received vaccine at baseline and week 4. A fifth cohort of subjects, who had been previously vaccinated against smallpox, received a single dose of $1x10^8$ $TCID_{50}$ of MVA-BN® (IMVAMUNE®) s.c. In this study the vaccine induced a strong and dose dependent immune response and was safe and well tolerated even at high doses (Vollmar et al. 2005)

Study POX-MVA-004: This double-blind randomized, phase II, dose finding trial in 165 healthy, vaccinia naïve male and female volunteers aged 18–32 years evaluated the immunogenicity and safety of MVA-BN® (IMVAMUNE®) at doses of $2x10^7$, $5x10^7$ or $1x10^8$ $TCID_{50}$. Subjects were

randomly assigned to one of three treatment groups and were immunized twice at a 4 week interval using MVA-BN® (IMVAMUNE®) (s.c.). Results of PRNT and ELISA, detecting neutralizing antibodies and total IgG against vaccinia respectively are shown in Table II.

This study revealed that all three dose levels induced a 100% seroconversion after the second vaccination. The strongest immune response with earliest onset of seroconversion was achieved with the highest dose tested. In this group, 94% of the subjects already seroconverted after only 1 vaccination. The data obtained with neutralizing antibodies closely correlated to the ELISA results (Figure I). A significance test of the correlation shows a highly significant result: $p = 1.5^{-18}$ and confirms that both assays are highly correlated. T cell response data: Cellular immune responses were analyzed in this trial with intracellular cytokine staining (ICS) detecting vaccinia-specific IFN-γ producing CD4+ / CD8+ cells. Cellular immune response resulted in a dose-dependent CD8+ response (Figure II).

Table II: POX-MVA-004 Humoral immune response (per protocol population

			ELISA			PRNT		
**Group**	**Visit Day**	**N**	**n (%)**	**GMT**	**95% CI (LL / UL)**	**n (%)**	**GMT**	**95% CI (LL / UL)**
Group 1 (N=54) $2x10^7$ $TCID_{50}$	0	54	0 (0%)	1.00	- / -	0 (0%)	1.00	- / -
	28	54	32 (59.3%)	14.37	7.71 / 26.8	4 (7.4%)	1.31	1.01 / 1.70
	42	54	54 (100%)	377.22	288.33 / 493.53	23 (42.6%)	5.51	3.17 / 9.59
	84	54	51 (94.4%)	134.33	91.05 / 198.19	10 (18.5%)	1.94	1.31 / 2.85
Group 2 (N=49) $5x10^7$ $TCID_{50}$	0	49	0 (0%)	1.00	- / -	0 (0%)	1.00	- / -
	28	49	40 (81.6%)	53.21	29.86 / 94.87	6 (12.2%)	1.55	1.10 / 2.19
	42	49	49 (100%)	583.62	461.58 / 737.94	29 (59.2%)	10.31	5.78 / 18.40
	84	49	49 (100%)	227.76	176.40 / 294.07	11 (22.4%)	2.32	1.47 / 3.66
Group 3 (N=52) $1x10^8$ $TCID_{50}$	0	52	0 (0%)	1.00	- / -	0 (0%)	1.00	- / -
	28	52	49 (94.2%)	98.52	67.57 / 143.65	5 (9.6%)	1.39	1.05 / 1.85
	42	52	52 (100%)	813.77	628.74 / 1053.26	37 (71.2%)	19.43	11.05 / 34.16
	84	52	52 (100%)	323.63	246.84 / 424.30	15 (28.8%)	2.94	1.81 / 4.76

N = number of subjects with samples for antibody response, n (%) = number of seropositive subjects / percentage of seropositive subjects based on N, GMT = Geometric Mean Titer, n (%) = number/percentage of seropositive subjects, CI = Confidence Interval, LL: Lower Limit, UL: Upper Limit
Seropositivity rate was defined as the percentage of subjects with antibody titers ≥ the assay cut-off value.
ELISA: The assay cut-off value is 1:50.
PRNT: The assay cut-off value is 1:20.

Figure I: POX-MVA-004 ELISA/PRNT Correlation analysis

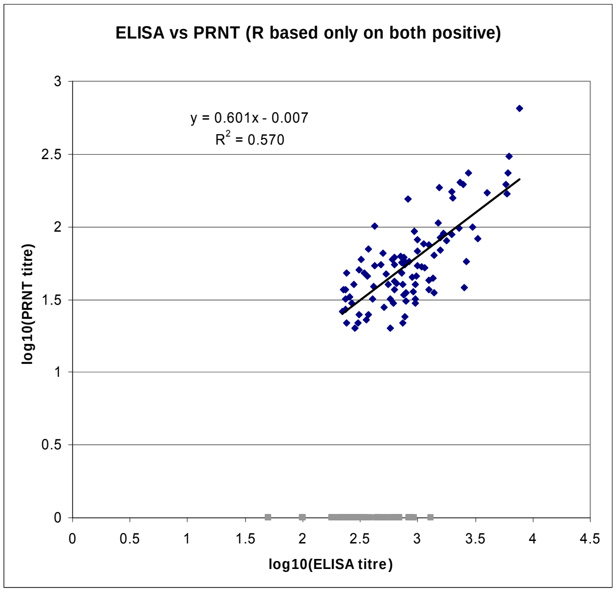

The ELISA titer versus the PRNT titer for all 162 available day 42 samples. The correlation calculation is based only on the samples where both titer values are positive – 94 positive pairs in all. 75% of the negative PRNT values had (log10 of) ELISA titers below 2.73, whereas all the samples with a positive PRNT titer had (log10 of) ELISA titers above 2.34.

Figure II: POX-MVA-004 Intracellular cytokine staining / vaccinia specific immunity

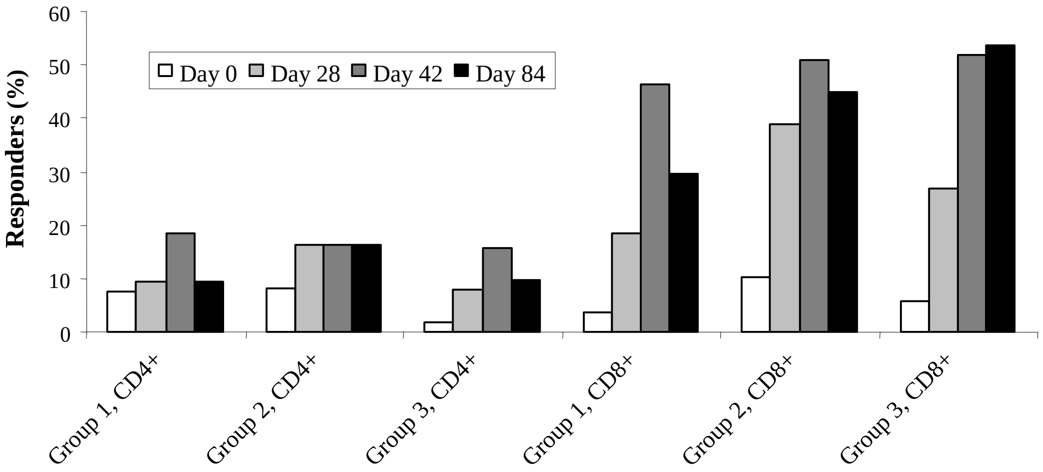

Group 1: 2x 10^7 $TCID_{50}$, subcutaneous in subjects not vaccinated against smallpox
Group 2: 5x 10^7 $TCID_{50}$, subcutaneous in subjects not vaccinated against smallpox
Group 3: 1x 10^8 $TCID_{50}$, subcutaneous in subjects not vaccinated against smallpox
Analysis was performed on the per protocol population

Study POX-MVA-002: In addition MVA-BN® (IMVAMUNE®) is currently being tested in the US in a Phase I study (POX-MVA-002) in healthy subjects in combination with the licensed smallpox vaccine Dryvax®.

A total of 90 subjects were enrolled 75 of which have received MVA-BN® (IMVAMUNE®) in different doses. Currently, the study is still blinded and it is expected that final results of the study will become available during 2006. However no serious and unexpected adverse events related to MVA-BN® (IMVAMUNE®) have been reported. One subject reported shortness of breath 2 weeks after vaccination with Dryvax® and this cardiac adverse event was assessed by the Investigator as definitely being related to Dryvax®.

MVA-BN®-based vaccination of populations for which Dryvax® is contraindicated

Patients belonging to risk populations such as immune compromised (e.g. HIV infected patients) or otherwise immunologically impaired (e.g. patients with atopic disorders) are normally excluded from vaccination with traditional smallpox vaccines (e.g. Dryvax®). To show that MVA-BN® (IMVAMUNE®) is a safe and immunogenic even in these patients, Bavarian Nordic up to date initiated three clinical trials in HIV infected subjects and/or subjects with atopic dermatitis with MVA-BN® (IMVAMUNE®) or MVA-BN® based vaccines. Initial trials in HIV-1 infected subjects using the recombinant MVA-BN® based Nef vaccine are completed and study reports available.

Studies HIV-NEF-001 and HIV-NEF-002: Both phase I vaccination studies conducted in Germany had the objective to evaluate safety and tolerability of a recombinant MVA vaccine expressing the HIV-1 nef-gene (MVA-nef) in HIV-1 infected patients on HAART. In study HIV-NEF-002 the subjects underwent a structured therapy interruption after the third vaccination.
24 patients with CD4 counts ≥ 400/µl were enrolled and immunized with $5x10^8$ $TCID_{50}$ of the MVA-nef vaccine, administered subcutaneously.
In both studies it was demonstrated that $5x10^8$ $TCID_{50}$ MVA-nef was safe and well tolerated in HIV-1 infected patients with CD4 counts ≥ 400/µl. The most frequent adverse reactions were mild injection site reactions such as induration or pain.

Study POX-MVA-007: To evaluate safety and immunogenicity of MVA-BN® (IMVAMUNE®) smallpox vaccine in subjects with atopic disorders, this open-label, controlled phase I pilot study was started in Germany in May 2004.

Primary objective of the trial is to expand the available data on the safety of MVA-BN® (IMVAMUNE®) in a population with atopic disorders. Vaccinia-naïve volunteers between 18 and 40 years were assigned to the following study groups depending on their medical history and current health status:

- Healthy subjects (n=15)
- Subjects with history of atopic dermatitis (n=16)
- Subjects with mild active atopic dermatitis (n=15)
- Subjects with mild allergic rhinitis (n=14)

All subjects are immunized twice at a 4 week interval using MVA-BN® (IMVAMUNE®) subcutaneously in a dose of $1x10^8$ $TCID_{50.}$ The study duration is up to 36 weeks for each subject. All subjects will be followed for at least 24 weeks after having received the last study vaccination

A full safety report of the first nineteen subjects in this study is available. This interim safety report demonstrates a low number of grade 3 adverse events (AEs) and confirms a favorable safety profile of MVA-BN® (IMVAMUNE®) even in a population in which conventional smallpox vaccines are contraindicated. Non-serious unsolicited and solicited AEs documented in this

report were comparable to typical adverse reactions expected with other modern injectable vaccines (Table III).

Table III:Adverse reactions with an occurrence of > 10% in at least one subject group (29-day follow-up period after vaccination) - per subject (full analysis set, N = 60)

Symptom	Healthy (N* = 15)			History of atopic dermatitis (N* = 16)			Active atopic dermatitis (N* = 15)			Allergic rhinitis (N* = 14)		
	n	(%)	n*	n	(%)	n*	n	(%)	n*	n	(%)	n*
Injection site pain	14	(93.3)	27	14	(87.5)	25	15	(100.0)	30	14	(100.0)	27
Injection site induration	13	(86.7)	23	11	(68.8)	17	15	(100.0)	23	12	(85.7)	18
Injection site swelling	12	(80.0)	22	13	(81.3)	20	11	(73.3)	18	11	(78.6)	17
Injection site erythema	12	(80.0)	23	15	(93.8)	26	15	(100.0)	25	14	(100.0)	26
Injection site pruritus	3	(20.0)	5	4	(25.0)	5	5	(33.3)	7	1	(7.1)	1
Inj. site discolouration	2	(13.3)	3	0	(0.0)	0	1	(6.7)	1	1	(7.1)	1
Injection site warmth	0	(0.0)	0	2	(12.5)	2	1	(6.7)	1	0	(0.0)	0
Fatigue	5	(33.3)	6	10	(62.5)	13	7	(46.7)	9	9	(64.3)	13
Headache	6	(40.0)	7	7	(43.8)	12	5	(33.3)	6	6	(42.9)	6
Myalgia	0	(0.0)	0	6	(37.5)	9	2	(13.3)	4	4	(28.6)	4
Nausea	1	(6.7)	1	1	(6.3)	1	2	(13.3)	2	2	(14.3)	2
Pyrexia	1	(6.7)	1	2	(12.5)	2	3	(20.0)	3	0	(0.0)	0
Pruritus	0	(0.0)	0	0	(0.0)	0	2	(13.3)	3	0	(0.0)	0
Dermatitis atopic	0	(0.0)	0	0	(0.0)	0	2	(13.3)	2	0	(0.0)	0

% = Percentages based on N*, N = number of subjects, N* = number of subjects with documented vaccination periods, n = number of subjects with findings in specific category, n* = number of events

Study POX-MVA-010: In July 2005, BN initiated a multi-center, open-label phase I/II study with the objective to generate safety and immunogenicity data with MVA-BN® (IMVAMUNE®) in HIV infected subjects with CD4+ counts >350 / µl compared to healthy subjects. 150 male and female vaccinia-naïve and pre-immune volunteers are assigned to the following study groups depending on their medical history and health status:

Group 1: HIV infected subjects without history of previous smallpox vaccination (n=30)
Group 2: HIV infected subjects with history of previous smallpox vaccination (n=60)
Group 3: Healthy subjects without history of previous smallpox vaccination (n=30)
Group 4: Healthy subjects with history of previous smallpox vaccination (n=30)

The subjects are immunized (s.c.) with a MVA-BN® (IMVAMUNE®) dose of 1 x 10^8 $TCID_{50}$. Group 1 and 3 will receive two vaccine administrations 4 weeks apart; group 2 and 4 a single administration.
The study is being performed at 5 study centers in the US and enrollment of volunteers has been completed.
Solicited systemic adverse reactions ≥ grade 3 observed to date are shown in Table IV.

Table IV:POX-MVA-010 (un-cleaned data) -- Solicited systemic adverse reactions ≥ grade 3

**Symptom**	**HIV vacc. naïve (N=30)**	**HIV pre-immune (N=61)**	**Healthy vacc. naïve (N=30)**	**Healthy pre-immune (N=30)**
	**n (%)**	**n (%)**	**n (%)**	**n (%)**
Injection site pain	0 (0%)	2 (3.3%)	2 (6.7%)	1 (3.3%)
Injection site erythema	0 (0%)	1 (1.6%)	0 (0%)	1 (3.3%)
Injection site swelling	0 (0%)	0 (0%)	0 (0%)	0 (0%)
Injection site induration	0 (0%)	0 (0%)	0 (0%)	0 (0%)
Fever	0 (0%)	1 (1.6%)	0 (0%)	1 (3.3%)
Headache	1 (3.3%)	0 (0%)	2 (6.7%)	1 (3.3%)
Myalgia	0 (0%)	1 (1.6%)	1 (3.3%)	1 (3.3%)
Chills	1 (3.3%)	1 (1.6%)	2 (6.7%)	1 (3.3%)
Nausea	0 (0%)	1 (1.6%)	0 (0%)	2 (6.7%)
Fatigue	1 (3.3%)	3 (4.9%)	2 (6.7%)	0 (0%)

N=Number of subjects, n=Number of subjects fulfilling criteria, %=n/N

Study HIV-NEF-004: This phase II study is currently being conducted in Germany to evaluate immunogenicity and safety of two doses of a recombinant MVA vaccine expressing the HIV-1 nef-gene ($1x10^8$ and $5x10^8$ MVA-nef) and one dose of $1x10^8$ $TCID_{50}$ MVA-BN®. To date, a total of 77 HIV-1 infected subjects with CD4 counts > 250/µl have been enrolled and have received all 3 vaccinations.. To date, MVA-nef and MVA-BN® (IMVAMUNE®) are well tolerated. Most frequent adverse reactions are mild injection site reactions like induration and pain.

1.5 Rationale

The fact that conventional smallpox vaccines have an unfavourable safety profile with vaccine-associated adverse events such as death, permanent disability and scarring, has lead to a considerable reluctance to vaccinate major parts of the population even on a voluntary basis. Current guidelines from the Advisory Committee on Immunization Practices (ACIP) for administering the smallpox vaccine state that subjects with eczema of any degree and those with a past history of eczema should not be vaccinated under routine circumstances because of the higher risk of developing eczema vaccinatum. Given that the lifetime prevalence of atopic dermatitis is ~10% to 15%, that probably even more people have a “history of eczema” and that about twice this many people have a contact with the above, it is conceivable that up to half of the population currently would not be eligible to routinely receive smallpox vaccination.

Although it is known that people with atopic dermatitis are at increased risk to develop eczema vaccinatum there are not many studies published. One publication documented the incidence of eczema vaccinatum in England and Wales during a mass vaccination campaign in 1962. Of the 6.5 million people vaccinated (with approximately 50% primary vaccinees), 185 developed

eczema vaccinatum and 11 of these 185 patients (6%) died. Rash anamnesis (active or past) indicated that 80% of those with eczema vaccinatum suffered from atopic dermatitis. Two thirds of the patients with atopic dermatitis did not have active disease at the time of exposure (Copeman and Wallace, 1964). Smallpox vaccine complication rates documented in the United States show an occurrence of eczema vaccinatum in 123 per million primary vaccinees, with case fatality rates of approx. 1% (Lane et al., 1971).

Relatively few recent research is available on potential causes of eczema vaccinatum, though specific immune defects in patients with atopic dermatitis are presumed to be causative (Leung et al., 1983; Lever et al, 1985; Kimura et al., 1998; Campbell et al., 1999; Higashi et al., 2001). Patients with atopic dermatitis have also been shown to have reduced cytotoxic T-cell generation and decreased IFN γ production. Both components of the immune response have been found to be critical for protection against vaccinia infection (Engler et al., 2002).

Due to the fact that MVA-BN® (IMVAMUNE®) is based on a non-replicating virus it is presumed to be safe in patients with atopic dermatitis, although very rare but potentially severe side effects cannot be excluded in a clinical trial setting. However, due to the fact that proposed causes of eczema vaccinatum after vaccination with conventional vaccinia vaccines include specific immune defects, it will be important to evaluate whether patients with atopic dermatitis will generate an adequate immune response after vaccination with highly attenuated vaccinia strains such as MVA-BN® (IMVAMUNE®).

1.6 Dose, route of administration, vaccination schedule

MVA-BN® (IMVAMUNE®) will be given subcutaneously. The rationale for the selected dose and the route of administration comes from historical use as well as preclinical and clinical data (study V-POX-00.1 (POX-MVA-001)) that showed satisfactory immune responses after a $1x10^8$ $TCID_{50}$ dose and a good safety profile of MVA-BN® (IMVAMUNE®) if administered via the subcutaneous route.

All subjects will receive two vaccinations each with a dose of 0.5 ml MVA-BN® (IMVAMUNE™) containing 1×10^8 $TCID_{50}$ vaccine administered subcutaneously according to a 0 – 4 weeks schedule (Day 0 / Day 28-35).

1.7 Benefit/risk assessment

Preclinical data with MVA-BN® (IMVAMUNE®) reveal no special hazard for humans based on conventional studies of safety. Over 1000 healthy and over 100 subjects with HIV or Melanoma have been vaccinated in clinical trials with vaccines using MVA-BN® (IMVAMUNE®) alone (to develop an improved smallpox vaccine) or with MVA-BN® as vector (with HIV specific inserts or inserts to be used as melanoma vaccine).

The good safety profile of MVA-BN® as a viral vector vaccine is supported by the historical experience with the MVA-BN® (IMVAMUNE®) precursor vaccine, which was used in Germany during the smallpox vaccination for two-stage parenteral vaccination in children in 1976. MVA was used in over 120,000 Caucasian individuals with no reported major side effects, even though many of the subjects were among the population with high risk of developing complications (Mayr et al., 1978).

It could be concluded that from the previous experience with MVA based vaccines adverse reactions to MVA-BN® (IMVAMUNE®) are expected to be comparable to typical adverse reactions seen with other modern vaccines.

The severe and life-threatening adverse reactions such as progressive vaccinia, eczema vaccinatum, generalized vaccinia and inadvertent inoculation that are observed after the administration of conventional smallpox vaccines are due to the replication of the vaccinia strains. Since MVA-BN® (IMVAMUNE®) is replication incompetent in human cells it has a better safety and tolerability profile and can consequently not induce the severe side effects such as progressive vaccinia which is associated with replication competent vaccinia viruses. Apart from the better safety profile with regard to severe reactions the available clinical experience with MVA-BN® (IMVAMUNE®) shows that it is generally better tolerated (e.g. local reactions) than conventional smallpox vaccines.

Based on the available data with MVA-BN® (IMVAMUNE®) the risk for the study participants is not expected to be higher than the risk observed with other modern vaccines. The main risk is the development of local reactions at the vaccination site (e.g. erythema, pain, swelling and induration).

In addition, the study participants can contribute significantly to the development of a safe smallpox vaccine. They might acquire a potential protection against smallpox. However, it cannot be concluded that the vaccine is efficacious against this disease, as MVA based vaccines are currently in an early phase of development. In view of the expected side effects of MVA-BN® (IMVAMUNE®) the potential risks associated for the study participants in this study seems to be limited and justify the potential benefit for society.

2. Objectives

The primary objective of the trial is:

- To assess the humoral immune responses (measured by ELISA) induced to MVA-BN® (IMVAMUNE®) in subjects with diagnosed atopic dermatitis compared to healthy subjects.

The secondary objectives of the trial are:

- To assess safety of MVA-BN® (IMVAMUNE®) in subjects with atopic dermatitis compared to healthy subjects
- To assess reactogenicity of MVA-BN® (IMVAMUNE®) in subjects with atopic dermatitis compared to healthy subjects
- To assess the immune responses measured with the plaque reduction neutralization test (PRNT) and the Enzyme-Linked Immunospot (ELISPOT) assay induced to MVA-BN® (IMVAMUNE®) in subjects with diagnosed atopic dermatitis compared to healthy subjects. ELISPOT: Subgroup of 50 subjects from Group 1 (healthy) and 110 subjects from Group 2 (AD) with samples taken at each of the Visits 1, 2 and 4 collected at US sites. The first subjects enrolled into the study will be included in the subgroup until the numbers specified above are reached.

3. Study design

3.1 Experimental design

This study is an open-label, controlled, phase II clinical trial to evaluate immunogenicity and safety of MVA-BN® (IMVAMUNE®) smallpox vaccine in 18-40 year old subjects with diagnosed atopic dermatitis.

The following two subject groups are compared:
Group 1: Healthy, vaccinia naïve subjects without atopic disease
Group 2: Vaccinia naïve subjects with diagnosed atopic dermatitis. “Diagnosed” atopic dermatitis includes subjects with either history of or subjects with currently active (defined as SCORAD ≤ 30, Kunz et al., 1997) atopic dermatitis.

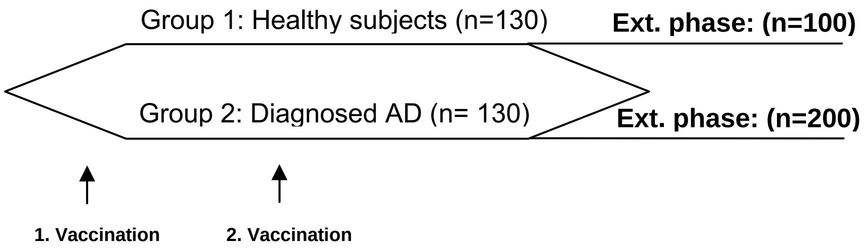

Visit Days						
-28- -1	0	7 - 10	28 – 35	V3 + 10-14	V3 + 28-35	V3 + 182–210
Scr	V1	V2	V3	V4	V5	F-U

3.2 Description of study procedures

The study will be conducted according to the Study Flow Chart (section III).

Visits should be scheduled within the given intervals.

3.2.1 Screening phase

Screening Visit (SCR, day -28 to –1 / week –4 to -1)

The informed consent must be reviewed with and signed and dated by the subject (volunteer) prior to the initiation of any evaluations or procedures required by the protocol. All subjects must be thoroughly informed of all aspects of the study (e.g. study visit schedule, required evaluations and procedures) as described in the informed consent document.

After informed consent has been collected, subjects will enter a screening period of up to four weeks before the first vaccination.

An extensive screening assessment will be performed during the screening period (visit SCR).

VISIT SCR (DAYS -28 TO -1)
• Informed Consent<br>• Check inclusion/exclusion criteria<br>• Screening examinations<br>• Check cardiac risk factors (including Total, HDL and LDL cholesterol)<br>• Check medical history<br>• Complete physical examination including evaluation of vital signs, especially listening to heart and lung<br>• Interview on history of AD (Group 2 only)<br>• Examination of AD including SCORAD (Group 2, active AD only)<br>• Baseline ECG<br>• Serum pregnancy test (if applicable)<br>• Urine analysis<br>• Safety laboratory<br>• Recording and documentation of Baseline Signs and Symptoms

The assessment of the atopic dermatitis group will be done by interviewing the subjects concerning their history of AD and/or doing a SCORAD evaluation in subjects with currently active AD. A photograph of the most prominent active AD area(s) could be taken.

All conditions leading to the decision to enroll a subject either into Group 1 or Group 2 have to be thoroughly documented in the source data!

If a subject was screened and could not be enrolled because of a certain transient condition (e.g. abnormal lab value due to an acute condition or a missing lab evaluation due to mishandling of the sample), then the subject could be re-screened and the respective tests should be repeated as a "partial" re-screening rather than a full re-screening. The re-screening visit must be within the 28 days window started by the first screening visit and the window -28 to -1 before 1st vaccination must not be exceeded. A “partial” re-screening visit is indicated by filling only the respective re-screening CRF pages.

If a subject could not be enrolled due to other circumstances (e.g. intermediate closure of the study group because of an interim safety analysis) and the 28 day period is over, a complete re-screening assessment including physical examination, lab examination, ECG must be performed. The clock then re-starts at the re-screening visit with day -28 before 1st vaccination. A “complete” re-screening visit is indicated by filling all re-screening CRF pages.

3.2.2 Active study phase

After successful screening period the subject will enter the active study phase starting with Visit 1.
The procedures performed at Visit 1 and all following visits are listed below. Blood draws and all other examinations listed above the vaccination events must always be performed **prior** to vaccination!
Investigators will be informed, if blood for T cell analysis will be drawn from their study subjects.

Subjects of both study groups will receive two s.c. vaccinations with 0.5 ml MVA-BN® (IMVAMUNE®) vaccine containing 1 x 10^8 $TCID_{50}$, four weeks apart (Visit 1 / Day 0 and Visit 3 / Visit 1 + 28-35 days) in the non-dominant upper arm (deltoid region).

Following vaccination subjects will be kept under close observation for at least 30 minutes, with appropriate medical treatment readily available in case of a rare anaphylactic reaction following the administration of vaccines.

Any adverse events (AE) that occur during or after vaccination will be recorded. A diary card will be provided to each subject for the recording of solicited AEs occurring on the vaccination day and the seven days following vaccination.

At Visit 5, the subjects of study group 2 will be interviewed about the status of their AD. In case of significant changes (improvement / worsening), a photograph of the most prominent active AD area(s) could be taken.

**VISIT 1 (DAY 0)**
• Re-check inclusion/exclusion criteria<br>• Check cardiac risk factors<br>• Targeted physical examination with focus on evaluation of vital signs, especially listening to the heart and lungs<br>• Recording and documentation of baseline signs and symptoms<br>• Urine pregnancy test (if applicable)<br>• Blood draw for antibody analysis<br>• Blood draw for T cell analysis (subgroup)<br><br>Blood draws and all other tasks mentioned above must always be performed prior to vaccination!<br><br>• 1st vaccination<br>• Handout of diary card for 1st vaccination<br>• Recording and documentation of AEs

**VISIT 2 (VISIT 1 + 7-10 DAYS)**
• Examination of the vaccination site<br>• Targeted physical examination with focus on evaluation of vital signs, especially listening to the heart and lungs<br>• ECG<br>• Safety Lab<br>• Blood draw for antibody analysis<br>• Blood draw for T cell analysis (subgroup)<br>• Collection of the diary card for 1st vaccination, review together with subject and transfer to the Case Report Form (CRF)<br>• Recording and documentation of AEs

**VISIT 3 (VISIT 1 + 28-35 DAYS)**
• Re-check inclusion/exclusion criteria<br>• Check cardiac risk factors<br>• Targeted physical examination with focus on evaluation of vital signs, especially listening to the heart and lungs<br>• Urine pregnancy test (if applicable)<br>• Blood draw for antibody analysis<br><br>Blood draws and all other tasks mentioned above must always be performed prior to vaccination!<br><br>• 2nd vaccination<br>• Handout of diary card for 2nd vaccination<br>• Recording and documentation of AEs

VISIT 4 (VISIT 3 + 10-14 DAYS)
• Examination of the vaccination site<br>• Targeted physical examination with focus on evaluation of vital signs, especially listening to the heart and lungs<br>• ECG<br>• Safety Lab<br>• Blood draw for antibody analysis<br>• Blood draw for T cell analysis (subgroup)<br>• Collection of the diary card for 2nd vaccination, review together with subject and transfer to the Case Report Form (CRF)<br>• Recording and documentation of AEs

VISIT 5 (VISIT 3 + 28-35 DAYS)
• Targeted physical examination including evaluation of vital signs, especially listening to the heart and lungs<br>• Brief interview concerning AD status- including SCORAD (Group 2, active AD only)<br>• ECG (only if clinically indicated)<br>• Urine pregnancy test (if applicable)<br>• Safety Lab (only if clinically indicated)<br>• Blood draw for antibody analysis<br>• Recording and documentation of AEs

3.2.3 Follow-up phase

To secure long-term safety, the subject has to come in for an examination in a follow-up phase 26 (+4) weeks after the last vaccination.

VISIT F-U (VISIT 3 + 182 – 210 DAYS)
• Targeted physical examination including evaluation of vital signs, especially listening to the heart and lungs<br>• Brief interview concerning AD status<br>• ECG (only if clinically indicated)<br>• Safety laboratory (only if clinically indicated)<br>• Blood draw for antibody analysis<br>• Recording and documentation of Serious Adverse Events (SAEs)

3.2.4 Protocol Extension

After enrollment of 130 subjects each in Group 1 and Group 2, the enrollment into the two study groups will be extended by another 100 vaccinia naïve healthy subjects and 200 vaccinia naïve subjects with diagnosed atopic dermatitis. Please refer to Appendices IV and V for details of the protocol extension.

3.3 Study duration

The total duration of the study for each subject including the screening period and follow-up visit will be up to 39 weeks. The duration of the study as a whole is dependent on the recruitment period. It is intended to perform recruitment within a reasonable time frame to limit the total study duration.
All subjects will be followed for at least 26 weeks after having received the last study vaccination. Please refer to Appendix IV for details of the protocol extension.

4. Selection of subjects

4.1 Recruitment procedure

Volunteers will be recruited actively. Methods of recruitment mainly include Investigator databases and referrals from dermatological clinics and general practitioners specialized on dermatology. Recruitment strategies including paid advertisements, announcements and distribution of flyers will be evaluated.

In total, 560 individuals (260 subjects in the main study and then a further 100 healthy and 200 AD subjects in the extension phase) as defined in Section 4.2 will be enrolled. After signing the Informed Consent, subjects undergo screening procedures to check eligibility due to the following inclusion/exclusion criteria. In the event of a screen failure secondary to mild or limited acute illness or abnormal laboratory values the patient may be re-screened after resolution of the event. Re-screening may require only an additional blood draw or a complete re-screening evaluation, depending on the circumstances of and the time interval from the initial screening failure.

The Investigator will keep a log of subjects screened for the study, and provide the reason in case of exclusion.

Information about every patient entering the study will be provided to the contracted CRO.

Enrollment will be tracked to ensure that at least 50% of the subjects enrolled into Group 2 of the trial have active Atopic Dermatitis rather than history of Atopic Dermatitis. If it becomes apparent that less than 50% of group 2 have active AD then further enrollment of subjects with history of AD will be temporarily halted until the balance is once again achieved.

4.2 Inclusion criteria

Group 1:

1. Subjects without present or history of any kind of atopy. Subjects with seasonal allergies can only be enrolled if an association with other atopic disorders such as allergic rhinitis and asthma can be excluded.

Group 2:

2. Subjects with diagnosed atopic dermatitis. “Diagnosed” atopic dermatitis includes subjects with either documented history of or subjects with currently active (defined as SCORAD ≤ 30, Kunz et al., 1997) atopic dermatitis. Currently active atopic dermatitis includes relapsing forms of atopic dermatitis, also when subjects are enrolled during inflammation-free intervals.

All study subjects:

3. Male and female subjects between 18 and 40 years of age without history of smallpox vaccination.
4. Women must have a negative serum pregnancy test at screening and a negative urine pregnancy test within 24 hours prior to vaccination.
5. Women of childbearing potential must have used an acceptable method of contraception for 30 days prior to the first vaccination, must agree to use an acceptable method of contraception during the study, and must not become pregnant for at least 28 days after the last vaccination. A woman is considered of childbearing potential unless post-menopausal or surgically sterilized. (Acceptable contraception methods are restricted to abstinence, barrier contraceptives, intrauterine contraceptive devices or licensed hormonal products.)
6. Read, signed and dated informed consent document after being advised of the risks and benefits of the study in a language understood by the subject signed, and prior to performance of any study specific procedure.
7. Troponin I < 2 fold ULN.
8. White blood cells ≥ 2500/mm^3 and < 11,000/mm^3.
9. Absolute neutrophil count (ANC) within normal limits.
10. Negative urine glucose by dipstick or urinalysis.
11. Hemoglobin within normal limits.
12. Platelets within normal limits.
13. Adequate renal function defined as:
    a. Serum creatinine without clinically significant findings
    b. Urine protein < 30 mg/dL or none or trace proteinuria (by urinalysis or dip stick)
14. Adequate hepatic function defined as:
    a. Total bilirubin ≤ 1.5 x ULN in the absence of other evidence of significant liver disease
    b. AST (SGOT), ALT (SGPT) and alkaline phosphatase without clinically significant findings
15. Electrocardiogram (ECG) without clinically significant findings (e.g. any kind of atrioventricular or intraventricular conditions or blocks such as complete left or right bundle branch block, AV-node block, QTc or PR prolongation, premature atrial contractions or other atrial arrhythmia, sustained ventricular arrhythmia, 2 premature ventricularcontractions (PVC) in a row, ST elevation consistent with ischemia).
16. Availability for follow-up for the planned duration of the study (26 weeks after last vaccination).

4.3 Exclusion criteria

1. Pregnant or breast-feeding women.
2. Uncontrolled serious infection i.e. not responding to antimicrobial therapy.
3. History of any serious medical condition, which in the opinion of the Investigator would compromise the safety of the subject.
4. History of or active autoimmune disease. Persons with vitiligo or thyroid disease taking thyroid replacement are not excluded.
5. Known or suspected impairment of immunologic function including, but not limited to, clinically significant liver disease; diabetes mellitus; moderate to severe kidney impairment.
6. History of malignancy, other than squamous cell or basal cell skin cancer, unless there has been surgical excision that is considered to have achieved cure. Subjects with history of skin cancer at the vaccination site are excluded.
7. History or clinical manifestation of clinically significant and severe hematological, renal, hepatic, pulmonary, central nervous, cardiovascular or gastrointestinal disorders.
8. Clinically significant mental disorder not adequately controlled by medical treatment.
9. Any condition which might interfere with study objectives or would limit the subject's ability to complete the study or to be compliant in the opinion of the Investigator.
10. History of coronary heart disease, myocardial infarction, angina, congestive heart failure, cardiomyopathy, stroke or transient ischemic attack, uncontrolled high blood pressure.
11. History of an immediate family member (father, mother, brother, or sister) who has had onset of ischemic heart disease before age 50 years.
12. Ten percent or greater risk of developing a myocardial infarction or coronary death within the next 10 years using the National Cholesterol Education Program's risk assessment tool. (http://hp2010.nhlbihin.net/atpiii/calculator.asp?usertype=prof) NOTE: This criterion applies only to volunteers 20 years of age and older.
13. History of intravenous drug abuse.
14. History of allergic disease or reactions likely to be exacerbated by any component of the vaccine.
15. Known allergy to egg or aminoglycoside (gentamicin).
16. History of anaphylaxis or severe allergic reaction.
17. Acute disease (illness with or without a fever) at the time of enrollment.
18. Temperature $\geq$100.4°F ($\geq$ 38.0°C) at the time of enrollment.
19. Subjects undergoing treatment for tuberculosis infection or disease.
20. Having received any vaccinations or planned vaccinations with a live vaccine within 30 days prior or after study vaccination.
21. Having received any vaccinations or planned vaccinations with a killed vaccine within14 days prior or after study vaccination.
22. Chronic administration (defined as more than 14 days) of systemic immuno-suppressants during a period starting from six months prior to administration of the vaccine and ending at study conclusion. Subjects receiving chronic treatment (defined as more than 14 days) with topical calcineurine inhibitors (eg tacrolimus, pimecrolimus) on the body or > 25% of the facial area are excluded. A wash-out period of ≥ 14 days for tacrolimus and ≥ 28 days for pimecrolimus can be considered sufficient before enrollment into the study.
    Subjects receiving chronic treatment (defined as more than 14 days) with high dose topical cortisone (> 30 grams/week of an intermediate potency [class IV] topical steroid [or equivalent], the treated area must not exceed 10% of the total body surface) are excluded.

23. Post organ transplant subjects whether or not receiving chronic immunosuppressive therapy.
24. Administration or planned administration of immunoglobulins and/or any blood products during a period starting from 3 months prior to administration of the vaccine and ending at study conclusion.
25. Use of any investigational or non-registered drug or vaccine other than the study vaccine within 30 days preceding the first dose of the study vaccine, or planned administration of such a drug during the study period.
26. Study personnel.

5. Study halting/termination and withdrawal of subjects

5.1 Study halting and termination rule

A temporary halting or termination for single study subjects or for the study as a whole can be decided in case of an occurrence of

- a serious adverse event (SAE)
- an unexpected grade 3 or higher systemic reaction or lab toxicity

with an at least reasonable possibility of a causal relationship to the administration of MVA-BN® (IMVAMUNE®), i.e. the relationship cannot be ruled out.
These parameters are not all-inclusive. Other adverse events could occur that would trigger a Data Safety Monitoring Board (DSMB) review. Any member of the DSMB, the Principal Investigator, BN Safety Officer, or DMID Medical Officer could request a Safety Monitoring Committee review based on any observation.

5.2 Reporting of events fulfilling the study halting criteria

If an event fulfilling the study halting criteria reaches the Investigator's attention, the Investigator has the liability to alert Kendle's Safety Department immediately (within 24 hours) and provide a comprehensive documentation of the event.

5.3 Data Safety Monitoring Board (DSMB)

The DSMB is an independent board that oversees the safety of volunteers participating in the study. The members of the DSMB are selected by BN and the DMID in accordance with the DMID guidelines. The primary responsibilities of the DSMB are to periodically review and evaluate the accumulated study data for participant safety, study conduct and progress, and make recommendations to BN, the DMID and the Principal Investigator(s) concerning the continuation, modification, or termination of the trial program. The DSMB considers study specific data as well as relevant background knowledge about the disease, test agent, and patient population under study. A separate charter describes in detail relevant operational procedures, communication pathways, roles and responsibilities of the DSMB.

In case an event occurs which fulfills the study halting criteria the DSMB will review the event in a timely manner and give a recommendation to BN, the DMID and the Principal Investigator(s) to halt, resume or terminate the study participation of the affected subject and/or the study as a whole.

5.4 Individual withdrawal criteria during the study

Subjects may withdraw or be removed from the study for any of the reasons cited below:

- An adverse event occurs that, in the opinion of the Investigator, makes it unsafe for the subject to continue the study. In this case, the appropriate measures will be taken.
- Subject's request to withdraw.

- Subject unwilling or unable to comply with study requirements.
- Clinical need for concomitant or ancillary therapy not permitted in the study.
- Unrelated intercurrent illness that, in the judgment of the Principal Investigator, will affect assessment of clinical status to a significant degree.

The following criteria should be checked at any visit after enrollment into the study. If any become applicable during the study, the subject may be withdrawn:

Use of any investigational or non-registered drug or vaccine other than the study vaccine(s).
Administration of a licensed vaccine not foreseen by the study protocol during the study period.
Start of chronic administration (defined as more than 14 days) of systemic immuno-suppressants during the study period.
Administration of immunoglobulins and/or any blood products during the study period.

5.5 Subject withdrawal procedure

If a subject discontinues prior to completion of the study, the date and reason for the discontinuation will be obtained. The date of the last dose of study medication will also be obtained.

Once a subject receives MVA-BN® (IMVAMUNE®), he/she must be followed for safety as stated in the protocol. From the time of discontinuation, all diagnostic procedures and evaluations scheduled for the last main study visit (Visit 5) should be performed (see III. FLOW CHART).

As a general rule, subjects who discontinued the trial after having received at least one vaccination will not be replaced.

Subjects included in the study but already discontinued the trial prior to the first vaccination should be replaced.

5.6 Contraindications and precautions for further study vaccinations

Absolute contra-indications:
The following events constitute absolute contra-indications to further administration of the study vaccine; if any of these occur during the study, the subject must not receive additional doses of vaccine, but may continue other study procedures at the discretion of the Investigator. The subject must be followed until resolution of the event, as with any adverse event:

- Anaphylactic reaction following the administration of vaccine(s).

- Pregnancy.

Subjects who become pregnant during the study period (up to and including one month [minimum 28 days] after receiving the last vaccine dose) must not receive additional doses of vaccine but may continue other study procedures at the discretion of the Investigator.

Subjects should be instructed to notify the Investigator if it is determined after completion of the study that they became pregnant either during the study or within one month (minimum 28 days) after receiving the last vaccine dose. Pregnancy must be reported to BN on a Pregnancy Form within 24 hours of the Investigator's becoming aware of the event.

A pregnancy should be followed to term, any premature terminations reported, and the health status of the mother and child including date of delivery and the child's gender and weight should be reported to Bavarian Nordic after delivery.

Temporary deferral of vaccination:
The following adverse event constitutes a contra-indication to administration of the study vaccine at that point in time; if this adverse event occurs at the time scheduled for vaccination, the subject may be vaccinated at a later date or withdrawn at the discretion of the Investigator. The subject must be followed until resolution of the event, as with any adverse event:

- Acute disease at the time of vaccination. (Acute disease is defined as the presence of a moderate or severe illness with or without fever. The vaccine can be administered to persons with a minor illness such as diarrhea, mild upper respiratory infection with or without low-grade febrile illness, i.e., oral temperature ≤ 100.4°F (≤ 38.0°C).

6. Study treatment

6.1 Investigational product

MVA-BN® (IMVAMUNE®) is a highly attenuated live vaccinia virus. MVA-BN® (IMVAMUNE®) will be provided in liquid-frozen aliquots. One dose of 0.5 ml liquid-frozen vaccine contains 1 x 10^8 $TCID_{50}$ Modified Vaccinia virus Ankara. MVA-BN® (IMVAMUNE®) is provided by BN. For details see current version of Investigator's Brochure.

Bavarian Nordic decided to exchange the IMVAMUNE® vaccine lot 170505 by IMVAMUNE® lot 0040707 during the study. For details, please refer to Appendix V.

6.2 Packaging and labelling

MVA-BN® (IMVAMUNE®) lot 0040707 is produced at Bavarian Nordic A/S and filled and labeled at the contract manufacturer IDT Biologika GmbH.

Address:
IDT Biologika GmbH
Dr. Margrit Gehrt
Am Pharmapark
06861 Dessau-Rosslau, Germany
Phone: +49-34901-885 0

The packages and vials will be labeled according to the national law of the respective study site.

6.3 Vaccine storage, handling and dispensing

The liquid frozen MVA-BN® (IMVAMUNE®) vaccine has to be stored at -20°C ± 5°C / -4°F ± 9°F. Do not re-freeze a vial once it has been thawed.

For administration, the vaccine vial will be thawed at room temperature. To ensure homogeneity, upon thawing the vial will be swirled gently (not shaken!) for at least 30 seconds. After thawing, the drug product should appear as a pale milky colored suspension. The liquid vaccine should be visually inspected for any foreign particulate matter prior to administration. In case foreign particulate matter is visible, the vaccine must not be used anymore.
The injection volume of 0.5 ml per dose will be withdrawn with a syringe using an injection needle long enough to reach the bottom of the vial. After withdrawal of the vaccine, the injection needle should be changed and the vaccine administered to the subject immediately.

If the vaccine cannot be administered immediately, it is recommended to administer the product within 12 hours after thawing. During this time the thawed vaccine vial has to be stored at +2°C to +8°C / 35.6°F to 46.4°F in the dark.

Details on vaccine handling can be found in Bavarian Nordic’s SOP, SOP/CLIN/16, entitled “Storage, Handling and Preparation of Liquid Frozen MVA-BN for Vaccination”.

6.4 Dose, vaccination schedule and route of administration

Subjects of both study groups will receive two s.c. vaccinations with 0.5 ml MVA-BN® (IMVAMUNE®) vaccine containing 1 x 10^8 $TCID_{50}$, four weeks apart (Visit 1 / Day 0 and Visit 3 / Visit 1 + 28-35 days) in the non-dominant upper arm (deltoid region). Details on vaccine administration can be found in Bavarian Nordic’s SOP, SOP/CLIN/16, entitled “Storage, Handling and Preparation of Liquid Frozen MVA-BN for Vaccination”.

6.5 Accountability and disposal

Used and unused vials should be stored in a safe place and remain the property of BN. The Principal Investigator of the respective site or his designee is responsible for ensuring adequate accountability of all used and unused study drug. This includes acknowledgement of receipt of each shipment of study drug (quantity and condition) and drug accountability using a drug inventory log. The drug inventory log will document quantity of study drug received from BN, quantity of study drug used for vaccination (including lot number, date dispensed, subject identification number and initials of the person dispensing the study medication) and quantity of study drug returned to BN or destroyed.
Additionally, the quantity of study drug returned to Bavarian Nordic or destroyed has to be documented on a study drug return form. In case destruction is agreed, material should be autoclaved or incinerated and discarded at site according to local regulations.
Additionally, used-syringes should be autoclaved or incinerated and discarded at site according to local regulations.

6.6 Concomitant medication

All concomitant medication must be recorded in the CRF with the reason for administration, the dosage regimen, and the onset and end of treatment.
For subjects with active AD, the use of calcineurin inhibitors during the study defined as ≤ 10 grams/week of tacrolimus (0.1% ointment) or pimecrolimus (1% cream) only on small areas of the face (defined as ≤ 25% of facial surface area) is allowed. The use of calcineurin inhibitors on body areas other than the face is not allowed.
Low dose topical cortisone therapy defined as ≤ 30 grams/week of an intermediate potency (class IV) topical steroid (or equivalent) is allowed, but the treated area must not exceed 10% of total body surface.

7. Clinical and laboratory assessments

7.1 Assessment of safety and reactogenicity

Taking into account the medical history of the subject, safety will be monitored by performing physical examinations including vital signs, routine laboratory measurements and ECGs as well as by evaluating local and systemic solicited adverse events and unsolicited adverse events.

Details regarding definitions and reporting of adverse events are described in chapter 8 of this protocol.

7.1.1 Medical history

The baseline assessment for safety parameters will be performed during screening visit. Medical history will be documented during the screening period (visit SCR). History will focus particularly on any important diseases and in case of infections or tumors, the pathogen involved or the pathological diagnosis, respectively, if available. A special attention should be given to history of prior allergic reactions, especially to vaccines.

7.1.2 Physical examination and vital signs

A physical examination and a check of vital signs will be performed during the screening period (visit SCR) for baseline assessment and on every following visit (Visits 1 – 5 and F-U).

PHYSICAL EXAMINATION:
The complete physical examination will be performed at the screening visit. The examination includes a review of major organ systems and weight. The examination should be directed at finding evidence of any infections, tumors and lymphadenopathy.
In addition, listening to the heart and lungs specifically for heart failure, presence of rubs, gallops, murmurs, crackles, and rales will be performed.

TARGETED PHYSICAL EXAMINATION:
A targeted physical examination, guided by any signs or symptoms previously identified or any new symptoms that the subject has experienced since the last visit, is required at all visits of the treatment period (Visits 1 – 5) and at the F-U Visit. Blood pressure, pulse rate, temperature will be taken. In addition, listening to the heart and lungs specifically for heart failure, presence of rubs, gallops, murmurs, crackles and rales will be performed.

VITAL SIGNS:
Blood pressure and pulse rate will be taken after the subject was seated for two minutes. Temperature will be measured orally.

7.1.3 Laboratory measurements

The intensity of laboratory / systemic quantitatively measured toxicities will be graded according to the toxicity scale in Appendix I. These grading scales include the laboratory values determined with the routine safety parameters. In case of other laboratory values not included in the routine safety laboratory and not listed in Appendix I, the National Cancer Institute (NCI) Common Toxicity Criteria table, Version 2.0, published April 30, 1999 will be used for grading of laboratory toxicities.

SAFETY LABORATORY:

Safety laboratory is determined at the screening visit (Visit SCR) and 7 – 14 days after each vaccination (for Visit 2: 7-10 days and Visit 4: V3+10-14 days). At Visit 5 and at follow-up visit (Visit F-U) safety laboratory is only done if clinically indicated. The safety laboratory measurements are performed at the central laboratory . Laboratory normal ranges are provided by the central laboratory and filed in the Investigator file. Safety laboratory parameters to be evaluated are:

Hematology:
Red blood cell count, hemoglobin, total and differential WBC, platelet count..

Serum chemistry:
Total bilirubin, alkaline phosphatase, AST, ALT, serum creatinine, sodium, potassium, calcium, troponin I.

PREGNANCY TEST:

β-HCG pregnancy test will be conducted for all women with reproductive potential at screening (Visit SCR), within 24 hours prior to each vaccination (Visits 1 and 3) and at Visit 5. At screening, a serum β-HCG pregnancy test will be conducted, all other pregnancy test will be conducted as urine β-HCG test.

The following parameters will only be evaluated during the screening period (Visit SCR) for check of inclusion / exclusion criteria:

CHOLESTEROL:

Total, HDL and LDL.

URINE ANALYSIS (E.G. WITH COMBUR-10 STICK):

Protein, glucose, occult blood, nitrites, leucocytes, pH, bilirubin, ketones, urobilinogen, specific gravity.

7.1.4 ECG assessments

A standard 12-lead ECG will be taken on all subjects at screening (Visit SCR) and 7 - 14 days after each vaccination (for Visit 2: 7-10 days and Visit 4: V3+10-14 days). For Visit 5 and the follow-up visit, an ECG is only done if clinically indicated.

ECGs will be evaluated by a centralized procedure. Unclear ECG abnormalities will additionally be evaluated by a central Cardiologist. The workflow / communication flow will be provided in a separate manual.

If clinically indicated, or in case of any kind of cardiac symptoms such as but not limited to chest pain, dyspnoea, arrhythmia, or edema subjects will be referred for a cardiac evaluation by a Cardiologist and further diagnostic tests will be done as recommended by this Cardiologist (e.g. (treadmill) ECG, cardiac enzymes, echocardiogram). Subjects will then be followed up for at least one year in a frequency determined by the Cardiologist.

Such adverse events fulfill the definition of an “adverse event of special interest” and are to be handled respectively reported as described under section 8.4.

Using replication-competent vaccinia-based smallpox vaccines during smallpox vaccination programs in the US during the last years, cases of acute myocarditis and pericarditis were observed (Grabenstein and Winkenwerder, 2003).

Case definitions as published by the US Center of Disease Control (CDC) ("Update: Cardiac-Related Events During the Civilian Smallpox Vaccination Program --- United States, MMWR May 30, 2003, Vol. 52, No. 21, p. 494") are provided in Appendix II in order to:

- help Investigators to recognize possible events of acute myocarditis and/or pericarditis and
- distinguish from unspecific and isolated ECG changes without or with unclear clinical meaning

Subjects who develop any kind of cardiac symptoms during the study such as but not limited to chest pain, dyspnea, arrhythmia, or edema are referred to a Cardiologist for cardiac evaluation including (treadmill) ECG, cardiac enzymes and echocardiogram. Depending on the results of this evaluation further diagnostic tests will be done as recommended by the Cardiologist and patients will be followed up for at least one year in a frequency determined by the Cardiologist. Furthermore unclear ECG abnormalities observed during the study will be examined upon recommendation of the cardiologist.
The observation of an adverse event as defined in section 8 will be rated by the investigator as "adverse event of special interest" and then be reported as described under section 8.7.

7.1.5 Assessment of solicited adverse events (Diary)

After each vaccination each subject receives a diary to record solicited local and systemic adverse events most likely to occur on the day of vaccination or the following 7 days. All solicited symptoms observed after vaccination with details concerning the intensity and the course of the reaction should be documented there. The Investigator will collect this information during the next scheduled visit and transfer it to the CRF.

In case of severe and unexpected local and systemic reactions, the study physician should be contacted outside from scheduled study visits.

Ongoing local and systemic reactions will be followed up and documented until resolved.

SOLICITED LOCAL ADVERSE EVENTS

The solicited local symptoms erythema, swelling and pain at the injection site have to be documented in the diary by the subject and intensity is assessed in the following way:
Injection site

Erythema:	size measured in diameter
Swelling:	size measured in diameter

The maximum intensity will be scored as follows:

0 =	0
1 =	< 30 mm
2 =	≥ 30 – <100 mm
3 =	≥ 100 mm

Injection site
Pain: 0 = Absent
1 = Painful on touch
2 = Painful when limb is moved
3 = Spontaneously painful / prevents normal activity

SOLICITED SYSTEMIC ADVERSE EVENTS

Subjects are asked to document the solicited systemic adverse events as described in the table below on the day of vaccination and the following 7 days on their diary card. If symptoms persist at day 7, temperature/symptom measurements should be recorded each day until resolved.

In the subject´s diary, the grading of maximum symptom intensity is described in basic, easily understood language based on the following descriptions:

**MedDRA coded Prefered Term Systemic Adverse Events**	**Grade**	**Maximum Intensity**
Body temperature*	0	< 99.5°F (< 37.5°C)
	1	≥ 99.5 - <100.4°F (≥ 37.5° – < 38.0°C)
	2	≥ 100.4 – 102.2°F (≥ 38.0 – < 39.0°C)
	3	≥ 102.2 - 104°F (≥ 39.0 - < 40.0°C)
	4	≥ 104°F (40.0°C)
Headache, Chills, Myalgia, Nausea and	0	None
Fatigue	1	Mild: Easily tolerated, minimal discomfort and no interference with daily activity
	2	Moderate: Some interference with daily activity
	3	Severe: Prevents daily activity
	4	Life-threatening or disabling

***Pyrexia** is defined as oral temperature ≥ 100.4°F (≥38.0°C).

7.1.6 Assessment of unsolicited adverse events

During every study visit following screening, the Investigator has to report any unsolicited adverse event experienced by the subject.

Unsolicited adverse events following the vaccination will generally be recorded by the subjects in a special section of the diary card and transferred by the Investigator to the adverse event section of the CRF. In addition, all intercurrent diseases not recorded on the diary card but reported when the Investigator actively inquires of the subject will be documented in the respective section of the case report form.

Adverse Events will be assessed and documented at all visits of the active study phase and if ongoing followed until resolution or F-U visit at the latest.
Serious Adverse Events will be assessed and documented at all study visits, including the F-U-Visit. Ongoing SAEs will be followed up until resolution or achievement of stable clinical conditions.

7.1.7 Assessment of intensity for adverse events

The scale for grading the maximum intensity of all adverse events will be based on the following descriptions:

1 = An adverse event which is easily tolerated by the subject, causing minimal discomfort and not interfering with daily activities.
2 = An adverse event which is sufficiently discomforting to interfere with daily activities.
3 = An adverse event which prevents daily activities. (Such an adverse event would, for example, prevent attendance at work and would necessitate the administration of corrective therapy.)
4 = Life threatening or disabling

7.1.8 Assessment of causality for adverse events

The relationship between the occurrence of an adverse event and the study drug will be assessed using the following categories:

**None**	The time interval between the administration of the study drug and the occurrence or worsening of the AE rules out a relationship
	and/or
	another cause is established and there is no evidence of a (concomitant) causal connection with or worsening caused by the study medication.
**Unlikely**	The time interval between administration of the study drug and the occurrence or worsening of the AE makes a causal relationship unlikely
	and/or
	the known effects of the study medication or substance class provide no indication of a (concomitant) causal connection with or worsening caused by the study medication and there is another cause which serves as an adequate explanation
	and/or
	although the known effects of the study medication or substance class make it possible to derive a plausible causal chain with regard to a (concomitant) causal connection or worsening, however, another cause is considerably more likely
	and/or
	another cause of the AE has been identified and a (concomitant) causal connection with or worsening caused by the study medication is unlikely.
**Possible**	A plausible causal chain with regard to a (concomitant) causal connection with / worsening of the AE can be derived from the pharmacological properties of the study medication or substance class. However, other approximately equally likely causes are known
	or
	although the pharmacological properties of the study medication or substance class provide no indication of a (concomitant) causal connection with / worsening of the AE, there is no other known cause which provides an adequate explanation.
**Probable**	The pharmacological properties of the study medication or substance class
	and/or
	the course of the AE after discontinuation of the study drug and possible subsequent re-exposure
	and/or
	specific findings (e.g. positive allergy test or antibodies against the trial drug / metabolites) suggest a (concomitant) causal connection with / worsening of the AE resulting from the study medication, however another cause cannot completely be ruled out.
**Definite**	The pharmacological properties of the study medication or substance class
	and/or
	the course of the AE after discontinuation of the study drug and possible subsequent re-exposure
	and/or
	specific findings (e.g. positive allergy test or antibodies against the trial drug / metabolites) definitely indicate that there is a (concomitant) causal connection with / worsening of the AE resulting from the study medication and there are no indications of other causes.

7.1.9 Algorithm for assessment of cardiac events

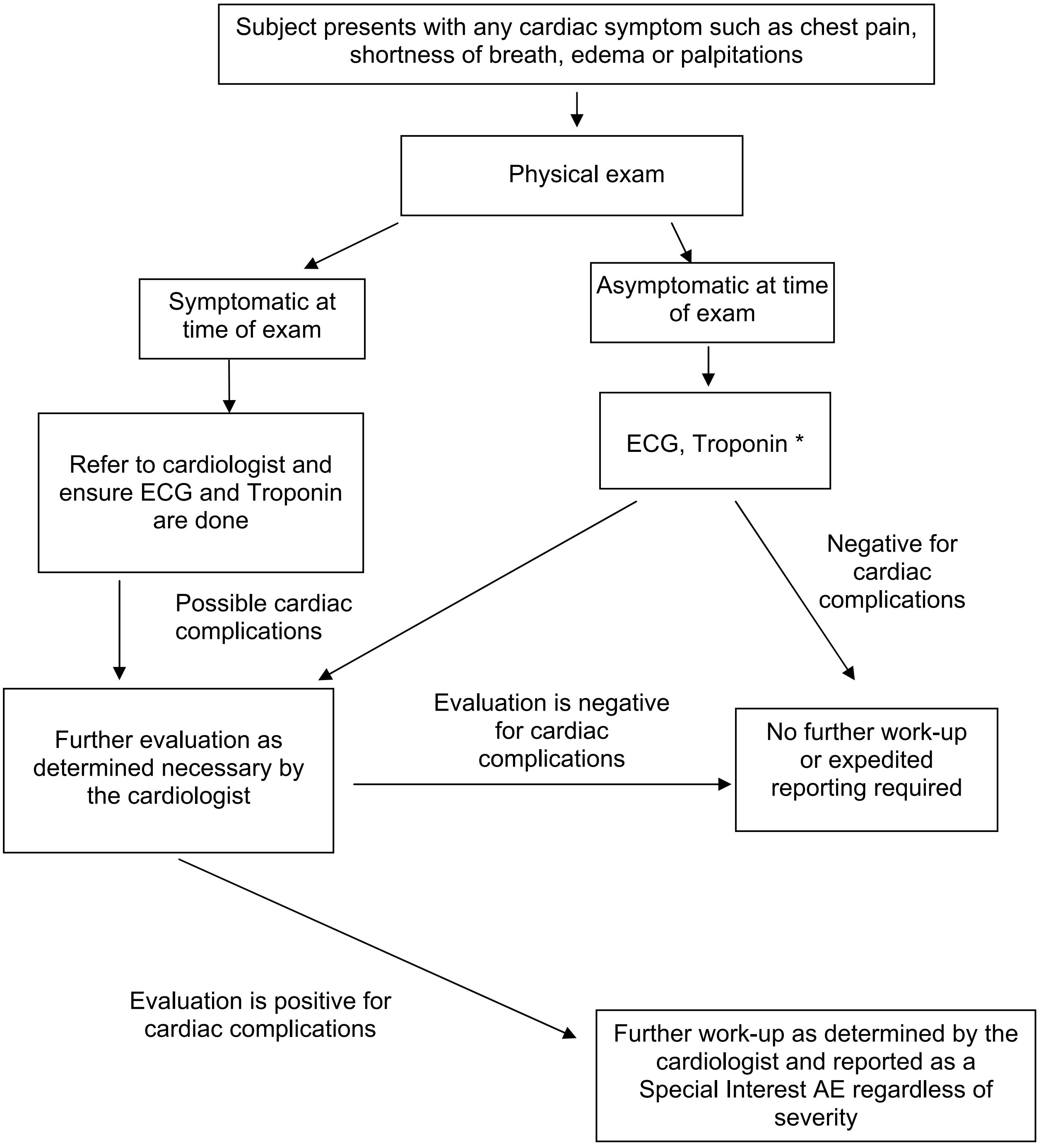

*At any protocol-scheduled ECG and/or Troponin abnormality the algorithm will begin at this point.

7.2 Assessment of immunogenicity

Immune response analysis is planned at any study visit except the screening visit. The baseline assessment for immunogenicity parameters will be performed during Visit 1 (before vaccination).

The methods of collection, storage and handling of lab specimen for the immune analysis are specified in the Covance (Central Laboratory) Laboratory Manual which will be provided to the investigators before enrollment. Additionally, training will be provided on the procedures during the investigator meeting and/or at the initiation visit.

7.2.1 Humoral immunogenicity

Antibody responses against MVA-BN® (IMVAMUNE®) will be measured using a direct ELISA and a plaque reduction neutralization test (PRNT), both validated assays. Tests will be performed at Bavarian Nordic GmbH in 82152 Martinsried, Germany. Focus Diagnostics Inc., Cypress, CA 90630, USA, acts as a back-up laboratory.

ELISA
Seroconversion is defined as:
appearance of antibody titers ≥ 1:50 in a vaccinia specific IgG ELISA for initially seronegative subjects or
twofold increase of the antibody titer compared to the pre-existing baseline titer for subjects with a pre-existing antibody titer in the ELISA.
The geometric mean titer (GMT) is calculated by taking the antilogarithm of the mean of the log10 titer transformations. Antibody titers below the cut-off of the assay will be given an arbitrary value of one for the purpose of calculation.

PRNT
Seroconversion is defined as Appearance of antibody titers ≥ 15 in a vaccinia specific plaque reduction neutralisation assay for initially seronegative subjects or twofold increase of the antibody titer compared to the pre-existing baseline titer for subjects with a pre-existing antibody titer in the PRNT.
The GMT is calculated by taking the antilogarithm of the mean of the log10 titer transformations. Antibody titers below the cut-off of the assay will be given an arbitrary value of one for the purpose of calculation.

7.2.2 Cellular immunogenicity

MVA-specific T-cell responses will be monitored in a subgroup of 50 subjects from Group 1 (healthy) and 110 subjects from Group 2 (AD) with samples taken at each of the Visits 1, 2 and 4 collected at US sites by measurement of IFN-γ production after stimulation with MVA-BN using an IFN-γ ELISPOT assay. A MVA-specific response is defined by a frequency of at least 50 SFU (spot forming units) / 10^6 cells after correction for background (SFU / 10^6 non-stimulated cells).

The immune cell analyses will be performed at BN´s laboratory at Bavarian Nordic GmbH, 82152 Martinsried, Germany.

8. Adverse event definitions and reporting

Any change in health observed before first vaccination will be recorded in the “baseline signs and symptoms” section of the CRF and will not be considered an adverse event.

8.1 Definition of adverse event

Adverse events are defined as any untoward (undesirable) medical occurrence in a clinical trial subject administered a medicinal product and which does not necessarily have a causal relationship with this medication. All adverse events (e.g. feeling of ill-health, subjective symptoms and objective signs, intercurrent diseases, accidents, etc.) observed by the Investigator and/or reported by the subject must be recorded in the CRF regardless of the assessment of causality in relationship with the study drug.

Abnormal laboratory values that were assessed as clinically significant by the Investigator are to be documented as AEs. In addition, abnormal laboratory values fulfilling the Grade 3 criterion according to the toxicity scale (Appendix I) are to be documented as AEs, regardless of whether they are considered clinically relevant or not.

8.2 Definition of solicited adverse events

Within this study protocol solicited adverse events are defined as all events recorded by the subjects in the diary provided to them following every vaccination. For each of the 7 days post-vaccination the subjects are requested to monitor and record local symptoms, i.e. erythema, swelling and pain at the site of injection as well as systemic symptoms, i.e. body temperature, headache, chills, myalgia, nausea and fatigue.

8.3 Definition of unsolicited adverse events

At every study visit the Investigator should ask the subject if they have experienced any adverse events since their last visit. All intercurrent diseases reported by the subject, regardless of whether recorded in the subject diary or not, need to be recorded by the Investigator in the appropriate page of the case report form.

The intensity and causality of the events will be graded according to the procedures described under 7.1.7 and 7.1.8 respectively.

8.4 Definition of “adverse event of special interest”

An “adverse event of special interest” is defined in this study as an adverse event fulfilling any of the following features:

- Any cardiac symptom
- ECG changes, which are determined to be clinically significant
- Cardiac enzymes elevated above ULN

Adverse events of special interest are to be reported according to the procedures and timelines applicable for serious adverse events.

8.5 Definition of serious adverse events (SAEs)

A serious adverse event (experience) or reaction is any untoward medical occurrence or effect that at any dose:

1. Results in death,
2. Is life-threatening*,
3. Requires inpatient hospitalization or prolongation of existing hospitalization,
4. Results in persistent or significant disability or incapacity,
5. Is a congenital anomaly or birth defect,
6. or is an otherwise important medical event.

**The term "life-threatening" in the definition of "serious" refers to an event in which the patient was at risk of death at the time of the event; it does not refer to an event which hypothetically might have caused death if it were more severe.*

Medical and scientific judgment should be exercised in deciding whether expedited reporting is appropriate in other situations, such as important medical events that may not be immediately life-threatening or result in death or hospitalization but may jeopardize the patient or may require intervention to prevent one of the other outcomes listed in the definition above. These should also usually be considered serious.

Examples of such events are intensive treatment in an emergency room or at home for allergic bronchospasm; blood dyscrasias or convulsions that do not result in hospitalization; or development of drug dependency or drug abuse.

8.6 Reporting of SAEs and adverse events of special interest

All serious adverse events (SAEs) or adverse events of special interest (see definition 8.4) occurring throughout the entire course of the study have to be reported to Kendle Safety Department. The study site has to send by e-mail or fax the completed serious adverse event form to Kendle Safety Department within 24 hours of becoming aware of the adverse event.

Kendle Safety forwards all serious adverse events and adverse events of special interest within 24 hours to Bavarian Nordic Drug Safety. Bavarian Nordic is responsible for expedited reporting to the US regulatory authorities (FDA) in compliance with regulations and to the responsible NIH-DMID representatives. For Mexico, all expedited cases will be forwarded by Bavarian Nordic Drug Safety to Kendle Mexico for submission to the Mexican Ministry of Health with an accompanying cover letter in Spanish.

The flowchart below outlines the reporting process and timelines:

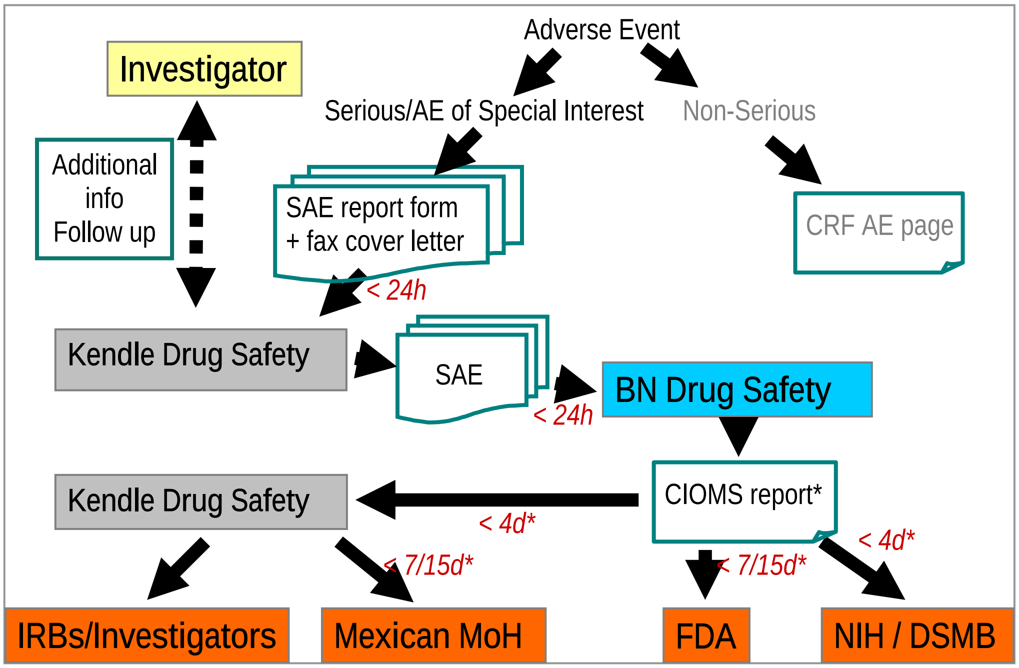

* if at least possibly causally related

Please fax SAE reports to the following number depending upon the country of origin:

Kendle Safety US:

fax: +1-866-395-9505
(Tel: +1- 866-395-8568)

Kendle Safety Mexico:

From sites outside Mexico City,
fax: 01-800-714-8106
(Tel: 01-800-714-9244)

From sites inside Mexico City,
fax: 51 35 47 68
(Tel: 54 81 59 44)

The Investigator should not delay reporting because of missing information. Nonetheless, the report should be as complete as possible. This initial notification should include, as a minimum, sufficient information to permit identification of the following:

the reporter
adverse event(s)
involved study medication
the subject
date of onset

9. Statistical Considerations

The primary objective of this trial is to compare the two vaccination groups with regard to total antibodies.

9.1 Sample size calculation

The sample size calculation is based on the primary immunogenicity endpoint '**MVA-specific seroconversion rate'**.
The primary hypothesis is to show that the humoral immune response of the group with diagnosed atopic dermatitis (Group 2) is not statistically inferior compared to the group with healthy subjects (Group 1) at 10-14 days after the second vaccination (Visit 4). The study should demonstrate that the Group 2 seroconversion rate is not worse than the Group 1 seroconversion rate by more than a pre-specified amount. This amount is called the non-inferiority margin (Δ).

Suppose p1 is the seroconversion rate in healthy subjects (group 1) and p2 is the seroconversion rate in the subjects with atopic dermatitis (group 2).
The test on non-inferiority will be applied for the following hypothesis:

H0: $p2 - p1 \leq -\Delta$ versus H1: $p2 - p1 > -\Delta$, where

Δ is the non-inferiority margin and is chosen in this trial as 5%.
From the experience in the pilot trial POX-MVA-007 in subjects with atopic dermatitis subjects, it is anticipated that the seroconversion rate in both groups reaches at least 99%.

The above hypothesis will be tested based on an exact, unconditional test for binomial differences. In addition an exact one-sided 97.5% unconditional confidence interval for the difference of proportions will be calculated. If the lower limit of this confidence interval is greater than 5% (or equivalent the p-value of the non-inferiority test is less than 5%) then the null hypothesis will be rejected (StatXact®, Chan 1998, Agresti and Min 2001).

Assuming a significance level of 5%, a power of > 80%, expected seroconversion rates of 99% in both groups, this yields to a sample size of 124 per group (248 in total). In order to account for drop outs, a total of 130 subjects per group (260 in total) will be treated.

For the statistical consideration of the protocol extension, please refer to Appendix IV.

9.2 Endpoints

9.2.1 Immunogenicity endpoints

Primary endpoint

- ELISA specific seroconversion rate at visit 4
  - *Seroconversion is defined as the appearance of antibody titers ≥ 1:50 in a vaccinia specific IgG ELISA for initially seronegative subjects or twofold increase of the antibody titer compared to the pre-existing baseline titer for subjects with a pre-existing antibody titer in the ELISA.*

Secondary endpoints

- ELISA specific seroconversion rate at visits 2, 3 ,5 and F-U.
- Geometric mean titers (at all blood sampling time points).
    - *The geometric mean titer (GMT) is calculated by taking the antilogarithm of the mean of the log10 titer transformations. Antibody titers below the cut-off of the assay will be given an arbitrary value of one for the purpose of calculation.*

- Neutralization assay specific seroconversion rates and geometric mean titers (at all blood sampling time points).
    - *Seroconversion is defined as Appearance of antibody titers ≥ 15 in a vaccinia specific plaque reduction neutralisation assay for initially seronegative subjects or twofold increase of the antibody titer compared to the pre-existing baseline titer for subjects with a pre-existing antibody titer in the PRNT.*
    - *The GMT is calculated by taking the antilogarithm of the mean of the log10 titer transformations. Antibody titers below the cut-off of the assay will be given an arbitrary value of one for the purpose of calculation.*

- Subgroup of 50 subject samples from Group 1 (healthy) and 110 subject samples from Group 2 (AD) collected at US sites: IFN gamma producing T-cells collected at Visits 1, 2 and 4 in response to stimulation with MVA-BN® (IMVAMUNE®) detected by Enzyme-Linked Immunospot (ELISPOT). The percentage of IFN gamma positive cells in T-cell populations compared to non-stimulated cells will be reported.

The primary time point for data analysis will be 10 – 14 days after the last vaccination (Visit 4) . Other time points at Visit 2, 3, 5 and F-U are used as secondary analysis time points.

9.2.2 Safety and reactogenicity endpoints

Secondary endpoints

- Occurrence of any grade 3 adverse event probably, possibly or definitely related to the study vaccine within 28 days after vaccination.
- Occurrence, relationship and intensity of any serious adverse event at any time during the study.
- Occurrence of solicited local adverse events (redness, swelling and pain) within 1 week after each vaccination (Days 0-7): Intensity, duration, and relationship to vaccination.
- Occurrence of solicited systemic adverse events (redness, swelling and pain) within 1 week after each vaccination (Days 0-7): Intensity, duration, and relationship to vaccination.
- Occurrence of unsolicited non-serious adverse events within 4 weeks after each vaccination (Days 0-28): Intensity, duration and relationship to vaccination.

9.3 Study cohorts/data sets to be evaluated

For the statistical analysis the included subjects will be divided up into the following datasets:

Safety set (SAS): This is the subset of subjects who received at least one dose of study vaccine and for whom any safety data are available.

The final safety analysis will be performed on this population.

Full-analysis set (FAS): This is the subset of subjects who had received at least one dose of study vaccine and for whom any data are available.

Modified Full Analysis Set (MFAS):
This set is defined to consist of all enrolled vaccinia-naïve healthy (Group 1) and at least 300 atopic dermatitis (Group 2) subjects.

- who have been enrolled as vaccinia-naïve based on the verbal confirmation of the volunteer not to have had a smallpox vaccination in the past and on examination of both arms for absence of a typical smallpox vaccination scar.
- who had received both doses of study vaccine and completed all study visits including Visit 4 and
- for whom safety data are available.

Per Protocol (PP) set: This is the subset of subjects who adhere to all protocol conditions, however, not relevant protocol violators can be included into this dataset.

The decision whether a protocol deviation is relevant or not for the classification of subjects to subsets should be made case-by case in a blind review meeting.

The primary population dataset for humoral immunogenicity analysis (ELISA and PRNT) will be the PP set. All confirmatory testing is based on this population. For further descriptive purposes, the same statistical procedures will be applied to the FA dataset.

ELISPOT Analysis Set (EAS):
This set is defined as the alternative PPS for the ELISPOT data analysis. It consists of all subjects with

- a complete ELISPOT data set (Visits 1, 2 and 4 available)
- the administration of both study vaccinations
- without any relevant major protocol violations for this data set.

ELISPOT data analysis will be descriptive and performed on both the EAS and the FAS.

Since two different vaccine lots will be used during the conduct of the study, a stratified subgroup analysis comparing subjects vaccinated either with vaccine lot 0170505 or vaccine lot 0040707 will be performed.

Any subject not included in the Full Analysis Set (FAS) will be considered a drop-out.

9.4 Statistical analysis

Once all subjects have completed the half-year follow-up (Visit F-U)and after any necessary settlement of queries etc. in the CRFs, data from all patients and visits will be locked. A full

analysis of the data available will be performed and a final clinical study report prepared. Results of the PRNT assay will be presented in an addendum to the clinical study report. For preparation of safety data as part of a potential Emergency Use Authorization (EUA) data package, a safety analysis will be performed on the population (MFAS) described in section 9.3 (‘Study cohorts/data sets to be evaluated‘).

9.4.1 Analysis of demographics and baseline characteristics

The results will be compared descriptively (shown in a table). If large differences become apparent then a test will be done to check the significance.

9.4.2 Analysis of Immunogenicity

Antibody titers and resulting seroconversion rates will be assessed by direct ELISA and PRNT method as described in section 7.2.
The primary immunogenicity endpoint is the MVA-specific seroconversion rate derived from the ELISA specific antibody titers.
The primary hypothesis is to show that the humoral immune response of the study group with atopic dermatitis (Group 2) is not statistically inferior compared to the study group of healthy subjects (Group 1).

The above hypothesis will be tested based on an exact, unconditional test for binomial differences. In addition an exact one-sided 97.5% unconditional confidence interval for the difference of proportions will be calculated. If the lower limit of this confidence interval is greater than ̃ 5% (or equivalent the p-value of the non-inferiority test is less than 5%) then the null hypothesis will be rejected (StatXact®, Chan (1998), Agresti and Min (2001)).

In a first step, the primary null hypothesis as stated in section 9.1 will be tested 2 weeks after the last vaccination.
Secondary analysis will be done with the timepoints of Visit 2, Visit 3, Visit 5 and F-U and with the neutralization assay specific seroconversion rates (from PRNT) as above.

Descriptive statistics for Enzyme-Linked Immunospot (ELISPOT) will be provided.
T-cell samples will be analyzed if samples are available for the subject from Visit 1 and at least one of Visit 2 or Visit 4.
Summary tables will be provided for the FAS and the EAS.

All statistical tests for secondary time points and comparisons are regarded descriptive. No adjustment for multiple testing will therefore be done.

Seroconversion rates for each group at all visits will be tabulated and presented graphically by means of bar charts.
Tables showing geometric mean titer (GMT) with 95% confidence limits for each group at all blood sampling time points will be prepared. Additionally, the distribution of antibody titers will be displayed graphically by means of reverse cumulative curves.

9.4.3 Analysis of safety and reactogenicity

Solicited local adverse events

The occurrence of solicited local adverse events within 1 week after each vaccination will be summarized on a per patient and per vaccination basis.
The maximum intensity over the 7-day period after vaccination will be used and categorized as follows:

Pain: Grade > 0
Grade ≥ 2
Grade ≥ 3
For measurements of diameter size: Diameter > 0 mm
Diameter ≥ 30 mm
Diameter ≥ 100 mm

These categories will be compared between treatment groups by means of the exact test of Fisher (or its normal approximation, the chi-squared test).

Solicited systemic adverse events

Occurrence of solicited systemic adverse events within 1 week after each vaccination will be summarized per patient and vaccination.

The maximum intensity over the 7-day period after vaccination will be used and categorized as follows:
Grade > 0
Grade ≥ 2
Grade ≥ 3
These categories will be compared between treatment groups by means of the exact test of Fisher (or its normal approximation, the chi-squared test).

Unsolicited adverse events:

Unsolicited adverse events will be coded with the MedDRA coding terminology. The intensity of adverse events will be graded according to section 7.1.7.
The number of adverse events and number of patients with at least one adverse event for each preferred term will be descriptively compared between treatment groups.

The occurrence of any grade 3 or higher adverse reaction probably, possibly or definitely related to the study vaccine within 28 days after vaccination will be compared between treatment groups.

The occurrence, relationship and intensity of *adverse events of special interest* at any time during the study will be separately listed and tabulated. The incidence of such AEs will be compared between treatment groups by means of the exact test of Fisher (or its normal approximation, the chi-squared test).

Serious adverse events will be listed separately. Each SAE will be described individually in detail. The number of subjects with at least one SAE will be compared between treatment groups by means of the exact test of Fisher (or its normal approximation, the chi-squared test).

Safety laboratory and urine analysis:

Clinical laboratory test results will be marked whether the result is below, within or above the respective reference range. The number of values outside of the reference range will be counted.

ECG

The ECG will be evaluated by a centralized procedure. The transmitted standard ECG results like PQ, QRS, QT and QTc duration and heart rate will be summarized per visit and treatment group.

The number of subjects with normal/abnormal ECGs and clinically significant ECGs (resulting in AEs) will be compared between treatment groups by means of the exact test of Fisher (or its normal approximation, the chi-squared test).
Detailed descriptive analysis of the reasons (category) of abnormality will be done.

9.4.4 Data handling

All data obtained in this study and documented in the case report forms (CRFs) will be listed. For parameters of interest, summary tables with descriptive group statistics (mean, standard deviation, minimum, maximum, number of valid cases) for metrical variables will be prepared. For ordinal/dichotomous variables summary tables showing the absolute and relative count in each category will be prepared.
Further statistical analysis may be performed as appropriate.

Kendle Inc. and GmbH will be responsible for data management and statistical evaluation. Data will be analysed using SAS® PC. The procedure for accounting for missing, unused and spurious data will be given in the Statistical Analysis Plan.

10. Ethical aspects

10.1 Ethical and legal regulations

The Principal Investigators make sure that this clinical trial is conducted in complete accordance with the provisions of the Declaration of Helsinki (and its amendments of Tokyo, Venice, Hong Kong, Somerset West and Edinburgh), the national laws and other guidelines for the conduct of clinical studies like the ICH Harmonised Tripartite Guideline for Good Clinical Practice to guarantee the greatest possible subject protection.

10.2 Approval of an Independent Ethics Committee (IEC) / Institutional Review Board (IRB)

The protocol must be reviewed by the competent IEC / IRB according to the national law of the respective site before the first subject is included in this study.

If one of the Investigators is a member of one of these committees, he may not vote on any aspect of the review of this protocol.

The Sponsor will assure that the IEC / IRB is informed of any amendment to the protocol and any unanticipated problems involving risks to human subjects included in the study. Such information will be provided to the committee at intervals appropriate to the degree of subject risk involved, but not less than once a year. Copies of all correspondence between the Investigator and the committee must be forwarded immediately to the sponsor. In case of withdrawal of IEC / IRB approval of the study, the sponsor has to be contacted immediately by facsimile or telephone.

10.3 Confidentiality and data protection

The Principal Investigator of the respective site is obliged to ensure anonymity of the subject. He/she has to make sure that all documents including CRFs provided (e.g. in the course of a marketing authorization procedure) to third parties (in this case: to the manufacturer of MVA-BN® (IMVAMUNE®) or to an authority) contain no subject names.
Only a subject and center number, not by their name or clinic and subject's file number, may identify subjects respectively. The Principal Investigators keep separate confidential subject logs for study enrollment, which allows subject numbers to be matched with names and addresses of subjects at any time. Documents not meant to be passed on to third parties have to be stored confidentially by the Principal Investigator.

Any information collected in the course of the study may be made available only to persons directly involved in this study (Principal Investigator and his staff members, monitor, statistician) or to authorized persons by the sponsor or the Principal Investigator or authorities.

11. Informed consent

No subject can participate in this study without having given informed consent in writing after the Investigator or his delegate has informed the subject clearly and completely, verbally and in writing, over the purpose, procedures, potential benefits and risks of the current study and prior to study drug administration.

One signed original of the informed consent must be given to each subject and one signed original must remain in the study documentation file and be available for verification by the monitor or competent regulatory authorities at any time.

Subjects must be informed unequivocally that they may refuse participation in the study and that they may withdraw from the study at any time and for whatever reason and that withdrawal of consent will not affect their subsequent medical treatment or relationship with the treating physician.

Subjects also consent to authorize the monitor, quality assurance personnel and regulatory authorities to inspect source documents for quality assurance purposes. Such verifications will always be conducted on site and under the ethical supervision of the Investigator. All aspects of the confidentiality of the subject’s data will be guaranteed.

The informed consent form will be prepared in accordance with ICH-GCP guidelines and must be approved by the appropriate IEC / IRB.

12. Case report forms and retention of records

12.1 Case report forms

All CRFs are to be filled out completely by the examining personnel, then reviewed and signed by the Principal Investigator to indicate their correctness.

It is the Principal Investigators responsibility to ensure that all subject discontinuations or changes in study or other medications entered on the subject’s CRF are also made on the subjects medical records.

The CRFs for any subject leaving the study should be completed at the time of the final visit or shortly thereafter.

12.2 Retention of records

The Investigator shall maintain the records of disposition of drug receipts, drug accountability forms, his copies of the CRFs, subject files and regulatory documents (e.g. informed consents, IEC / IRB approval, FDA1572, Financial Disclosure Forms) according to applicable national law after the end of the study.

13. Monitoring of the study

The monitor is responsible for obtaining an overview of the course of the trial in co-operation with the Investigator, checking if the trial protocol is being observed, and helping the Investigators to solve any problems which may arise. All documents in the context with this clinical trial will be handled confidentially at any time.

The Investigator has agreed to give the monitor access to relevant hospital or clinical records to confirm their consistency with the CRF entries and to obtain an adequate overview of the course of the trial. The monitor checks entries on the CRF for completeness, accuracy and correctness. The entries on the CRF will be verified against source documents with respect to the following criteria and in the following frequencies:

Informed consent	100%
Demographic data	100%
In-/exclusion criteria	100%
Primary target parameters	100%
Administration of study vaccine	100%
Adverse events	100%
Time points of visits	100%
Laboratory parameters	100%
Vital signs	100%

This will be done under preservation of data protection.

The source data verification must be performed by direct insight. If a subject refuses to consent to this procedure he/she must not be enrolled in the study.
The Investigator (or a representative) has further agreed to support the monitor in solving any problems he/she discovers during his/her visits.

14. Responsibilities of the Investigator

The Investigator agrees to carry out the study in accordance with the guidelines and procedures outlined in this trial protocol. The Investigator especially consents to strictly adhere to the ethical principles (see section 10 of this protocol).

The Investigator knows that he/she must, according to professional regulations for physicians, obtain the approval of the competent IEC / IRB.

Any deviation from the trial protocol must, before its implementation, be agreed to by the sponsor in writing, and by the IEC / IRB initially consulted.

Changes to the protocol require written ”Amendments to the protocol” and written approval by Principal Investigator. Changes are allowed only if study value is not reduced and if they are ethically justifiable. The statistician must agree to the amendment, if appropriate, his statement is to be submitted to the IEC / IRB. The amendment must be passed on to all participating Investigators with the obligation to adhere to its provisions. If warranted, the subject information has to be changed accordingly.

It is within the responsibility of the Investigator that a CRF is completed and signed after the subject has finished the trial for each subject participating in the study.

All entries on the CRFs must be made in black ball-point pen; corrections are made by placing a single horizontal line through the incorrect entry, so that it can still be seen, and placing the revised entry beside it. The revised entry must be filled in, initialled and dated by a member of the Investigator's research team authorized to make CRF entries. Correction fluid must not be used.

At the conclusion of the study, the Investigator will return all partly used, unused and empty drug containers to the sponsor.

The Investigator may ask to terminate the study due to administrative or other reasons. If this should be the case, appropriate measures which safeguard the interests of the participating subjects must be taken after verification and consultation with the Principal Investigator.

By signing this protocol, the Investigator confirms that he/she has read the entire trial protocol, agrees to its procedures, and will comply strictly with the formulated guidelines.

15. References

1. Agresti A, Min Y. (2001). On small-sample confidence intervals for parameters in discretedistributions. *Biometrics. 2001 Sep;57(3):963-71*

2. Alcami A & Smith GL (1992) A soluble receptor for interleukin-1 beta encoded by vaccinia virus: a novel mechanism of virus modulation of the host response to infection.*Cell* 71, 153-167.

3. Alcami A & Smith GL (1996) A mechanism for the inhibition of fever by a virus.*Proc.Natl.Acad.Sci.U.S.A* 93, 11029-11034.

4. Antoine G, Scheiflinger F, Dorner F, & Falkner FG (1998) The complete genomic sequence of the modified vaccinia Ankara strain: comparison with other orthopoxviruses.*Virology* 244, 365-396.

5. Blanchard TJ, Alcami A, Andrea P, & Smith GL (1998) Modified vaccinia virus Ankara undergoes limited replication in human cells and lacks several immunomodulatory proteins: implications for use as a human vaccine.*J.Gen.Virol.* 79 (Pt 5), 1159-1167.

6. Carroll MW & Moss B (1997) Host range and cytopathogenicity of the highly attenuated MVA strain of vaccinia virus: propagation and generation of recombinant viruses in a nonhuman mammalian cell line.*Virology* 238, 198-211.

7. Chan I (1998). Exact tests of equivalence and efficacy with a non-zero lower bound for comparative studies. *Statistics in Medicine* 17, 1403-1413.

8. Drexler I, Heller K, Wahren B, Erfle V, & Sutter G (1998) Highly attenuated modified vaccinia virus Ankara replicates in baby hamster kidney cells, a potential host for virus propagation, but not in various human transformed and primary cells.*J.Gen.Virol.* 79 (Pt 2), 347-352.

9. Goldstein JA, Neff JM, Lane JM, & Koplan JP (1975) Smallpox vaccination reactions, prophylaxis, and therapy of complications.*Pediatrics* 55, 342-347.

10. Guillaume JC, Saiag P, Wechsler J, Lescs MC, & Roujeau JC (1991) Vaccinia from recombinant virus expressing HIV genes.*Lancet* 337, 1034-1035.

11. Grabenstein JD, Winkenwerder W. Jr. (2003) US Military Smallpox Vaccination Program Experience. JAMA 2003, Vol. 289, No. 24, 3278 - 3282

12. Kunz B, Oranje AP, Labreze L, Stadler JF, Ring J, Taieb A. Clinical validation and guidelines for the SCORAD index: consensus report of the European Task Force on Atopic Dermatitis. *Dermatology* 1997, 195:10-19

13. Lane JM, Ruben FL, Neff JM, & Millar JD (1969) Complications of smallpox vaccination, 1968.*N.Engl.J.Med.* 281, 1201-1208.

14. Lane JM, Ruben FL, Neff JM, & Millar JD (1970) Complications of smallpox vaccination, 1968: Results of ten statewide surveys.*J.Infect.Dis.* 122, 303-309.

15. Mahnel H & Mayr A (1994) [Experiences with immunization against orthopox viruses of humans and animals using vaccine strain MVA].*Berl Munch.Tierarztl.Wochenschr.* 107, 253-256.

16. Mayr A, Hochstein-Mintzel V, & Stickl H (1975) Passage History: Abstammung, Eigenschaften und Verwendung des attenuierten Vaccina-Stammes MVA.*Infection* 3, 6-14.

17. Mayr A & Munz E (1964) [Changes in the vaccinia virus through continuing passages in chick embryo fibroblast cultures].*Zentralbl.Bakteriol.[Orig.]* 195, 24-35.

18. Mayr A, Stickl H, Muller HK, Danner K, & Singer H (1978) [The smallpox vaccination strain MVA: marker, genetic structure, experience gained with the parenteral vaccination and behavior in organisms with a debilitated defence mechanism (author's transl)].*Zentralbl.Bakteriol.[B]* 167, 375-390.

19. McElwain WP (1972) Complications of smallpox vaccination.*J.Ky.Med.Assoc.* 70, 165-166.

20. Meyer H, Sutter G, & Mayr A (1991) Mapping of deletions in the genome of the highly attenuated vaccinia virus MVA and their influence on virulence.*J.Gen.Virol.* 72 (Pt 5), 1031-1038.

21. Redfield RR, Wright DC, James WD *et al.* (1987) Disseminated vaccinia in a military recruit with human immunodeficiency virus (HIV) disease.*N.Engl.J.Med* 316, 673-676.

22. Rosel JL, Earl PL, Weir JP, & Moss B (1986) Conserved TAAATG sequence at the transcriptional and translational initiation sites of vaccinia virus late genes deduced by structural and functional analysis of the HindIII H genome fragment.*J.Virol.* 60, 436-449.

23. Stickl, H. A. (1974) Smallpox vaccination and its consequences: first experiences with the highly attenuated smallpox vaccine "MVA". Prev.Med, 3[1], 97-101.

24. Stickl H & Hochstein-Mintzel V (1971) [Intracutaneous smallpox vaccination with a weak pathogenic vaccinia virus ("MVA virus")].*Munch.Med.Wochenschr.* 113, 1149-1153.

25. Stickl H, Hochstein-Mintzel V, Mayr A *et al.* (1974) [MVA vaccination against smallpox: clinical tests with an attenuated live vaccinia virus strain (MVA) (author's transl)].*Dtsch.Med.Wochenschr.* 99, 2386-2392.

26. Sutter G & Moss B (1992) Nonreplicating vaccinia vector efficiently expresses recombinant genes.*Proc.Natl.Acad.Sci.U.S.A* 89, 10847-10851.

27. Vollmar, J *et al.* (2005) Safety and Immunogenicity of IMVAMUNE: A promising candidate as a third generation smallpox vaccine. *Vaccine, in press*.

16. Appendices

16.1 Appendix I: Toxicity Scale for laboratory values

SERUM CHEMISTRY				
**Lab value**	**Mild (Grade 1)**	**Moderate (Grade 2)**	**Severe (Grade 3)**	**Potentially Life Threatening (Grade 4)**
Sodium – Hyponatremia mmol/L	< LLN - ≥ 132	< 132 - ≥ 130	< 129 - ≥ 125	<125
Sodium – Hypernatremia mmol/L	≥ ULN - < 150	≥ 150 - < 155	≥ 155 - < 160	≥ 160
Potassium – Hyperkalemia mmol/L	≥ ULN - < 5.5	≥ 5.5 - < 6.0	≥ 6.0 - < 7.0	≥ 7.0
Potassium – Hypokalemia mmol/L	< LLN - ≥ 3.5	< 3.5 - ≥ 3.4	< 3.4 - ≥ 3.2	< 3.2
Calcium – Hypercalcaemia mmol/L	≥ ULN - < 2.9	≥ 2.9 - < 3.1	≥ 3.1 - < 3.4	≥ 3.4
Calcium - Hypocalcaemia mmol/L	< LLN - ≥ 2.0	< 1.75 - ≥ 2.0	< 1.6 - ≥ 1.75	< 1.5
Serum creatinine - mg/dL	≥ ULN - < 1.5 x ULN	≥ 1.5 - < 3 x ULN	≥ 3 - 6 x ULN	> 6 x ULN
Alkaline Phosphatase - increase by factor	> 1.25 - < 2.0 x ULN	≥ 2.0 - < 3.0 x ULN	≥ 3.0 x ULN	
Liver Function Tests - increase by factor	> 1.0 - < 2.5 x ULN	≥ 2.5 - < 4 x ULN	≥ 4 x ULN	
Total Bilirubin - increase by factor	> ULN – 1.5 x ULN	> 1.5 - 3.0 x ULN	> 3.0 - 10.0 x ULN	> 10.0 x ULN
Cardiac Troponin I	>ULN – <2.0 x ULN	≥ 2.0 - < 5.0 x ULN	≥ 5.0 x ULN	
Total Cholesterol mg/dL	> ULN - 300	> 300 - 400	> 400	

HEMATOLOGY				
**Lab Value**	**Mild (Grade 1)**	**Moderate (Grade 2)**	**Severe (Grade 3)**	**Potentially Life Threatening (Grade 4)**
Hemoglobin (Female) - g/dl	< LLN - ≥ 10.5	< 10.5 - ≥ 10.0	< 10.0	
Hemoglobin (Male) – g/dl	< LLN - ≥ 12.0	< 12.0 - ≥ 11.0	< 11.0	
WBC Increase - cell/mm^3	≥ ULN - < 15,000	≥ 15,000 - < 20,000	≥ 20,000	
WBC Decrease - cell/mm^3	< LLN - ≥ 2,500	< 2,500 - ≥ 1,500	< 1,500	
Lymphocytes Decrease - cell/mm^3	< 1,000 - ≥ 750	< 750 - ≥ 500	< 500	
Neutrophils Decrease - cell/mm^3	< 2,000 - ≥ 1,500	< 1,500 - ≥ 1,000	< 1,000	
Platelets Decreased - cell/mm^3	< LLN - ≥ 100,000	< 100,000 - ≥ 75,000	< 75.000	
**URINE**				
Protein	Trace - ≤ 1+	> 1+ - ≤ 2+	> 2+ - ≤ 3+	> 3+ - nephritic syndrome
Glucose	Trace	1+	2+	>2+
Blood (microscopic) – red blood cells per high power field (rbc/hpf)	≥ 0- <10	≥ 10 - < 50	≥ 50	Gross
Blood (measured by Combur urinsticks)	Trace - ≤ 1+	> 1+ - ≤ 2+	> 2+ - ≤ 3+	> 3+
**SYSTEMIC QUANTITATIVE**				
Tachycardia - beats per minute	101 - 115	116 – 130	≥ 131	
Bradycardia - beats per minute	54 - 50	49 – 45	≤ 44	
Hypertension (systolic) - mm Hg	141 - 155	156 – 165	≥ 166	
Hypertension (diastolic) - mm Hg	91 - 95	96 – 100	≥ 101	
Hypotension (systolic) - mm Hg	89 - 85	84 – 80	≤ 79	

16.2 Appendix II: Case Definitions Acute Myocarditis / Pericarditis

Case Definition for Acute Myocarditis

A possible case of acute myocarditis is defined by the following criteria and the absence of evidence of any other likely cause of symptoms:
Presence of dyspnea, palpitations, or chest pain of probable cardiac origin in a patient with either one of the following:

- Electrocardiogram (ECG) abnormalities beyond normal variants, not documented previously, including
    - ST-segment or T-wave abnormalities,
    - Paroxysmal or sustained atrial or ventricular arrhythmias,
    - AV nodal conduction delays or intraventricular conduction defects, or
    - Continuous ambulatory electrocardiographic monitoring that detects frequent atrial or ventricular ectopy

Or

- Evidence of focal or diffuse depressed left-ventricular (LV) function of indeterminate age identified by an imaging study (e.g., echocardiography or radionuclide ventriculography).

A probable case of acute myocarditis, in addition to the above symptoms and in the absence of evidence of any other likely cause of symptoms, has one of the following:

- Elevated cardiac enzymes, specifically, abnormal levels of cardiac troponin I, troponin T, or creatine kinase myocardial band (a troponin test is preferred);
- Evidence of focal or diffuse depressed LV function identified by an imaging study (e.g., echocardiography or radionuclide ventriculography) that is documented to be of new onset or of increased degree of severity (in the absence of a previous study, findings of depressed LV function are considered of new onset if, on follow-up studies, these findings resolve, improve, or worsen); or
- Abnormal result of cardiac radionuclide imaging (e.g., cardiac MRI with gadolinium or gallium-67 imaging) indicating myocardial inflammation.

A case of acute myocarditis is confirmed if histopathologic evidence of myocardial inflammation is found at endomyocardial biopsy or autopsy.

Case Definition for Acute Pericarditis

A possible case of acute pericarditis is defined by the presence of

- Typical chest pain (i.e., pain made worse by lying down and relieved by sitting up and/or leaning forward) and no evidence of any other likely cause of such chest pain.

A probable case of acute pericarditis is a possible case of pericarditis, or a case in a person with pleuritic or other chest pain not characteristic of any other disease, that, in addition, has one or more of the following:

- Pericardial rub, an auscultatory sign with one to three components per beat,
- ECG with diffuse ST-segment elevations or PR depressions without reciprocal ST depressions that are not previously documented, or
- Echocardiogram indicating the presence of an abnormal collection of pericardial fluid (e.g., anterior and posterior pericardial effusion or a large posterior pericardial effusion alone).

A case of acute pericarditis is confirmed if histopathologic evidence of pericardial inflammation is evident from pericardial tissue obtained at surgery or autopsy.

16.3 Appendix III: SCORAD Evaluation Form

POX-MVA-008

SCORAD Evaluation Form

Visit No. |__|

Visit date |__|__|.|__|__|__|.|_2|_0|__|__|
D D M M M Y Y Y Y

Subject Study No. |__|__|__|__|

Examiner *(Name)* ___________________________

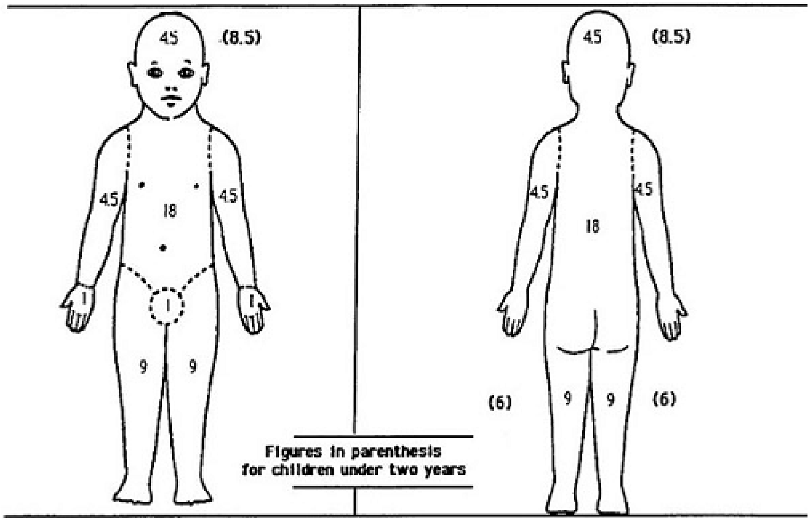

A: EXTENT. Please indicate the area involved ____________

B: INTENSITY. (as rated below) ____________

C: SUBJECTIVE SYMPTOMS
PRURITUS + SLEEP LOSS
____

CRITERIA	INTENSITY
Erythema	
Edema/Papulation	
Oozing/crust	
Excoriation	
Lichenification	
Dryness<br>is evaluated on uninvolved areas	

MEANS OF CALCULATION
INTENSITY ITEMS<br>(average representative area)<br>0=absence<br>1=mild<br>2=moderate<br>3=severe

SCORAD A/5 + 7B/2 + C
____

Visual analog scale (average for the last 3 days or nights)			
	**PRURITUS (0 to 10)**	0 1 2 3 4 5 6 7 8 9 **10**	☐
	**SLEEP LOSS (0 to 10)**	0 1 2 3 4 5 6 7 8 9 **10**	☐

16.4 Appendix IV: Proposed Extension Phase of the POX-MVA-008 trial

16.4.1 General Information and Rationale for an Extension Phase of the POX-MVA-008 trial

- The original protocol was designed to evaluate immunogenicity and safety of MVA-BN® (IMVAMUNE®) in vaccinia-naïve subjects with diagnosed atopic dermatitis compared to healthy subjects.

This extension phase implements the following major changes to Bavarian Nordic protocol POX-MVA-008:

- A protocol extension, which allows the recruitment of a further 100 healthy and 200 vaccinia-naïve subjects with diagnosed atopic dermatitis. A visit schedule describing examinations performed is detailed in section 16.4.2 “Extension Protocol Flowchart”.
- An additional interim analysis (proposed in Amendment #2) will no longer be conducted at the end of the main phase of the study (once 260 subjects have been enrolled in the main study and have completed their Visit 5). An analysis of the complete study population will be done once all 560 subjects have been enrolled and have completed their Visit 5, and will be performed as described under section 9.4. Once all 560 subjects have completed their Follow-Up assessment an addendum will be prepared including all follow-up data.
- The enrollment of a total of 300 subjects with diagnosed atopic dermatitis will allow the detection of 1 (one) unexpected adverse reaction in 100 (one hundred) subjects with 95% confidence, provided there is no background incidence of the respective adverse reaction.
- The sample size of 300 subjects with atopic dermatitis will meet the requirements suggested by CBER’s “Current Thinking” position paper regarding the data recommended in support of the use of MVA vaccines in a post-event setting under an EUA for individuals with contraindications to vaccinia vaccines.

The changes are not considered to have any negative influence on the study procedure in general, on the safety of the participants or on the validity of the main study results.

16.4.2 Extension Protocol Flow Chart

VISIT[1]	SCREENING	VISIT 1	VISIT 2	VISIT 3	VISIT 4	VISIT 5	TELEPHONE F-U
Day (D)	-28- -1 D	0	7-10 D	28-35	VISIT 3 +10-14 D	VISIT 3 +28-35 D	VISIT 3 +182-210 D
Vaccination		I		II			
Informed consent[2]	X						
Screening examinations	X						
Check incl. / excl. criteria, esp. cardiac risk	X	X		X			
Medical History (incl. history of atopic dermatitis)	X						
Interview on history of AD[7]	X						
Examination of AD including SCORAD[7] (active AD only)	X						
Brief interview on AD status[7] (including SCORAD for active AD only)						X	
Brief interview on AD status[7]							
Complete Physical examination incl. evaluation of vital signs, esp. listening to heart and lung	X						
Targeted physical exam.		X	X	X	X	X	
ECG	X		X		X	(X)[4]	(X)[4]
Pregnancy test[3]	X	X		X		X	
Safety Lab	X		X		X	(X)[4]	(X)[4]
Urine analysis	X						
Antibody analysis		X	X	X	X	X	
Handout of diary card[5]		X		X			
Collection of diary card			X		X		
Exam of vaccination site			X		X		
AE-reporting		X	X	X	X	X	
Telephone Follow-up for SAEs, SIAEs and AEs that were still ongoing at visit 5							X
Review baseline signs and symptoms	X	X					
Approx. blood sampling in ml[6]	*14*	*16*	*30*	*16*	*30*	*16 (+14)*[4]	*(14)*[4]

[1] If clinically indicated additional visits may be necessary between scheduled visits and possibly additional blood draws may occur.. Visits occurring between indicated scheduled visits will be identified with letters to indicate sequence of the visit until the next scheduled visit occurs (e.g. 1A, 1B etc. will indicate extra visits between visit 1 and 2).
[2] Consent process must be completed and form signed before any study-related procedures are conducted.
[3] Serum or urine pregnancy test, women of childbearing potential only. At screening visit, a serum test must be performed.
[4] Only required if clinically indicated.
[5] The Diary Card should be completed daily for 7 days. If symptoms persist at day 7, temperature/symptom measurements should be recorded each day until resolved.
[6] Approximate amount of blood needed for analysis: Safety lab: app.14 ml, Antibody analysis: app. 16 ml.
Amount of blood taken during the extension phase: app. 150 ml
[7] Group 2 (AD) only

For clarification:

Lot 170505: 1st enrolment period is from subject 1 to subject 177 (main phase)

Lot exchange: 2nd enrolment period is from subject 178 to subject 260 (main phase)

Lot exchange: 3rd enrolment period is from subject 261 to subject 530 (extension study)

16.4.3 Inclusion & Exclusion Criteria

The same inclusion and exclusion criteria from the main protocol apply, please refer to section "II Protocol Synopsis" (pages 13-15) and "4.2 Inclusion Criteria" and "4.3 Exclusion Criteria" (pages 32-33). .

16.4.4 Statistical Considerations

The primary objective of this protocol extension is to estimate the frequency of common and uncommon reactions in 300 vaccinia-naïve subjects with diagnosed atopic dermatitis. This sample size will allow the detection of 1 (one) unexpected adverse reaction in 100 (one hundred) subjects with 95% confidence, provided there is no background incidence of the respective adverse reaction.

The below table provides information on different scenarios for this assumption.
The following sample sizes are needed to detect 1, 2 or 3 ADR with 95% certainty[1]:

Expected incidence of ADR	Number of subjects required to detect 1 ADR	Number of subjects required to detect 2 ADR	Number of subjects required to detect 3 ADR
1:10	28	46	61
1:50	148	235	313
1:100	298	473	627

[1] Calculated by solving the following equation for *N*: $B(p_a; N, N_A - 1) = \beta$, where *B* is the accumulated binomial distribution, N_A is the number of adverse events we wish to (be able to) detect and $\beta = 0.05$.

16.5 Appendix V: Exchange of IMVAMUNE® vaccine lot 0170505 by IMVAMUNE® vaccine lot 0040707

The FDA has agreed with BN that lot 0170505 can be used to generate clinical data to support an EUA and have further agreed with BN's strategy to demonstrate comparability between IMVAMUNE® lots manufactured at different sites. However, in recent discussions with the US Department of Health and Human Services (DHHS), the importance of using a vaccine lot from Good Manufacturing Practice (GMP) lot-consistent manufacturing runs was re-emphasized again to comply with their own definition of a "usable product". Therefore, even though BN's previous strategy of using lot 0170505 had been approved by FDA, to ease the decision-making at DHHS to accept IMVAMUNE® for the national stockpile, BN has decided to change to IMVAMUNE® lot 0040707 for all subsequently enrolled study subjects in Bavarian Nordic's clinical study protocols.

For the study POX-MVA-008 this means that the currently used IMVAMUNE® vaccine lot 0170505 will be exchanged by IMVAMUNE® vaccine lot 0040707 with effective date Februar 1st, 2008.

The difference between the two IMVAMUNE® vaccine lots mainly lays in a so-called "re-derivation step" reducing the anyway minimal risk of transmission of particles causing the disease "transmissible/bovine spongiform encephalopathy" (TSE/BSE). Summarizing the process of re-derivation, the original MVA-BN® virus seed stock was re-cloned through 5 rounds of plaque purification by limited dilution reducing the risk of TSE/BSE contamination further down to a factor of 3.33×10^{-36}. The re-derived Master Seed Virus (MSV) is now being used as the starting material for lot 0040707.
Materials from the currently used lot 0170505 and from the new virus seed stock used for the production of lot 0040707 were compared both genetically and phenotypically and were confirmed to be 100% identical. Additionally, a GLP animal comparability study in mice revealed that IMVAMUNE® vaccine from both the original and the re-derived MSV induces an identical humoral immune response in mice resulting in equal protection of the animals against an orthopox challenge.

In Protocol Amendment #2 dated 12 December 2006 it was stated and explained that the IMVAMUNE® vaccine lot 0170505 would be exchanged by lot 0031105. With regard to an optimized clinical comparability program Bavarian Nordic has since then decided that it would be most ideal in this situation to take advantage of replacing the original vaccine lot 0170505 with a vaccine lot from the **final production run** planned to be supplied to the US Government for use under the Emergency Use Authorization guideline. This final batch is currently in the final stages of production at Bavarian Nordic's vaccine manufacturing facility in Denmark and the currently expected re-start and extension phase of the POX-MVA-008 clinical study is now scheduled for February 2008.

The final production run of IMVAMUNE® vaccine, lot 0040707, also uses re-derived Master Seed Virus, hence reducing the anyway minimal risk of transmission of particles causing the disease "transmissible/bovine spongiform encephalopathy" (TSE/BSE) referred to in Protocol Amendment #2.

All vials of the previously used IMVAMUNE® vaccine lot 0170505 have been retrieved from sites and destroyed and all of the subjects already enrolled into the study have now completed their follow-up visits.

To secure an adequate exchange of the currently used vaccine lot 0170505 against lot 0040707, the following rules in on-going trials with respect to the treatment of study subjects should apply:

1. Subjects currently in screening can be vaccinated with the currently delivered vaccine lot 0170505 until December 22nd, 2006.

2. Subjects who have been in screening and could not be vaccinated by December 22nd, 2006 have to wait until vaccine lot 0040707 is available. Due to the extended time of availability of vaccine lot 0040707 at the investigational site, these subjects will have to be re-screened.

3. Subjects who received the first vaccination using vaccine lot 0170505 will get the second vaccination also with vaccine lot 0170505. Vaccination of a subject with two different vaccine lots is not permitted.

4. Vaccine lot 0040707 will be delivered to clinical sites once FDA and IRB approval has been obtained.

5. As soon as the vaccine lot 0040707 is available at the investigational site, subjects will be exclusively vaccinated with this vaccine lot.

For evaluation of possible influence of the lot exchange, a stratified subgroup analysis comparing subjects vaccinated either with vaccine lot 170505 or vaccine lot 0040707 will be performed during the statistical analysis of the study.

The following table outlines the current enrollment status and the plans to include 100 additional healthy subjects in the trial to guarantee a statistically valid analysis of the trials using two different vaccine lots:

	**Initially Planned**	**Subjects vaccinated with lot 0170505 until December 22nd, 2006**	**Planned no. of subjects vaccinated with lot 0040707 starting as soon as the vaccine is available at sites**	**Total**
**Group1: Healthy**	130	105	125	**230**
**Group 2: Diagnosed AD**	300	72	258	**330**
**Total**	430	177	383	**560**

The sample size of 230 in the group enrolling healthy subjects will ensure that 125 healthy subjects are vaccinated with IMVAMUNE® lot 0040707 and could subsequently be statistically evaluated according to the statistical considerations in Section 9 of the study protocol.
The total sample size including 200 AD subjects enrolled in the extension phase (outlined in Clinical Protocol Amendment #1) and 100 additional healthy subjects (outlined in Clinical Protocol Amendment #2) will therefore increase to 560 subjects.
