# Supplementary material for: A Multicenter, Open-Label, Controlled Phase II Study to Evaluate Safety and Immunogenicity of MVA Smallpox Vaccine (IMVAMUNE) in 18–40 Year Old Subjects with Diagnosed Atopic Dermatitis
Source: PLoS One. 2015 Oct 6;10(10):e0138348. doi: 10.1371/journal.pone.0138348 (PMC4595076; doi:10.1371/journal.pone.0138348)
Supplement: S1 Table — (DOCX) [file pone.0138348.s012.docx]

Supplemental Tables

Unsolicited Adverse Events: Relationship (FAS=632)

| Relationship | Healthy (N=282) n (%) | Atopic Dermatitis (N=350) n (%) |
| --- | --- | --- |
| None | 144 (33.1) | 171 (31.7) |
| Unlikely | 117 (26.9) | 86 (16.0) |
| Possible | 76 (17.5) | 111 (20.6) |
| Probable | 60 (13.8) | 92 (17.1 |
| Definite | 38 (8.7) | 79 (14.7) |
| Total | 435 (100.0) | 539 (100.0) |

N: Number of subjects; n: Number of events; %: Percentage based on total number of events

Unsolicited Adverse Events: Intensity (FAS=632)

| Intensity | Healthy (N=282) n (%) | Atopic Dermatitis (N=350) n (%) |
| --- | --- | --- |
| Grade 1 | 298 (68.5) | 432 (80.1) |
| Grade 2 | 108 (24.8) | 86 (16.0) |
| Grade 3 | 29 (6.7) | 21 (3.9) |
| Grade 4 | 0 (0.0) | 0 (0.0) |
| Total | 435 (100.0) | 539 (100.0) |

N: Number of subjects; n: Number of events; %: Percentage based on total number of events

Intensity: Grade 1 = AE tolerated by subject; Grade 2 = AE discomforting activities; Grade 3 = AE prevents activities; Grade 4 = Life threatening (SAE)
