# Supplementary material for: A Multicenter, Open-Label, Controlled Phase II Study to Evaluate Safety and Immunogenicity of MVA Smallpox Vaccine (IMVAMUNE) in 18–40 Year Old Subjects with Diagnosed Atopic Dermatitis
Source: PLoS One. 2015 Oct 6;10(10):e0138348. doi: 10.1371/journal.pone.0138348 (PMC4595076; doi:10.1371/journal.pone.0138348)
Supplement: S2 Table — (DOCX) [file pone.0138348.s013.docx]

| **Unique Subject Identifier** | **Visit name** | **Results for ELISA MVA Titre** | **Results for PRNT MVA Titre** | **Stratified Study Group** | **Per Protocol Population** | **Number of vaccinations** |
| --- | --- | --- | --- | --- | --- | --- |
| POX-MVA-008-2001-002 | VISIT 1 | 1 | 1 | HEALTHY, VACCINIA NAIVE SUBJECTS WITHOUT ATOPIC DISEASE | N | 2 |
| POX-MVA-008-2001-002 | VISIT 3 | 100 | 15 | HEALTHY, VACCINIA NAIVE SUBJECTS WITHOUT ATOPIC DISEASE | N | 2 |
| POX-MVA-008-2001-002 | VISIT 4 | 6369 | 183 | HEALTHY, VACCINIA NAIVE SUBJECTS WITHOUT ATOPIC DISEASE | N | 2 |
| POX-MVA-008-2001-002 | VISIT 5 | 6308 | 280 | HEALTHY, VACCINIA NAIVE SUBJECTS WITHOUT ATOPIC DISEASE | N | 2 |
| POX-MVA-008-2001-010 | VISIT 1 | 1 | 1 | VACCINIA NAIVE SUBJECTS WITH ACTIVE ATOPIC DERMATITIS | N | 1 |
| POX-MVA-008-2001-013 | VISIT 1 | 1 | 1 | HEALTHY, VACCINIA NAIVE SUBJECTS WITHOUT ATOPIC DISEASE | N | 2 |
| POX-MVA-008-2001-013 | VISIT 2 | 1 | 1 | HEALTHY, VACCINIA NAIVE SUBJECTS WITHOUT ATOPIC DISEASE | N | 2 |
| POX-MVA-008-2001-013 | VISIT 3 | 100 | 1 | HEALTHY, VACCINIA NAIVE SUBJECTS WITHOUT ATOPIC DISEASE | N | 2 |
| POX-MVA-008-2001-013 | VISIT 4 | 1719 | 15 | HEALTHY, VACCINIA NAIVE SUBJECTS WITHOUT ATOPIC DISEASE | N | 2 |
| POX-MVA-008-2001-013 | VISIT 5 | 735 | 15 | HEALTHY, VACCINIA NAIVE SUBJECTS WITHOUT ATOPIC DISEASE | N | 2 |
| POX-MVA-008-2001-013 | FOLLOW-UP VISIT | 224 | 15 | HEALTHY, VACCINIA NAIVE SUBJECTS WITHOUT ATOPIC DISEASE | N | 2 |
| POX-MVA-008-2001-014 | VISIT 1 | 1 | 1 | HEALTHY, VACCINIA NAIVE SUBJECTS WITHOUT ATOPIC DISEASE | N | 2 |
| POX-MVA-008-2001-014 | VISIT 2 | 1 | 1 | HEALTHY, VACCINIA NAIVE SUBJECTS WITHOUT ATOPIC DISEASE | N | 2 |
| POX-MVA-008-2001-014 | VISIT 3 | 50 | 1 | HEALTHY, VACCINIA NAIVE SUBJECTS WITHOUT ATOPIC DISEASE | N | 2 |
| POX-MVA-008-2001-014 | VISIT 4 | 347 | 328 | HEALTHY, VACCINIA NAIVE SUBJECTS WITHOUT ATOPIC DISEASE | N | 2 |
| POX-MVA-008-2001-014 | VISIT 5 | 200 | 98 | HEALTHY, VACCINIA NAIVE SUBJECTS WITHOUT ATOPIC DISEASE | N | 2 |
| POX-MVA-008-2001-014 | FOLLOW-UP VISIT | 50 | 15 | HEALTHY, VACCINIA NAIVE SUBJECTS WITHOUT ATOPIC DISEASE | N | 2 |
| POX-MVA-008-2001-016 | VISIT 1 | 1 | 1 | HEALTHY, VACCINIA NAIVE SUBJECTS WITHOUT ATOPIC DISEASE | N | 2 |
| POX-MVA-008-2001-016 | VISIT 2 | 1 | 1 | HEALTHY, VACCINIA NAIVE SUBJECTS WITHOUT ATOPIC DISEASE | N | 2 |
| POX-MVA-008-2001-016 | VISIT 3 | 1 | 1 | HEALTHY, VACCINIA NAIVE SUBJECTS WITHOUT ATOPIC DISEASE | N | 2 |
| POX-MVA-008-2001-016 | VISIT 4 | 538 | 185 | HEALTHY, VACCINIA NAIVE SUBJECTS WITHOUT ATOPIC DISEASE | N | 2 |
| POX-MVA-008-2001-016 | VISIT 5 | 223 | 113 | HEALTHY, VACCINIA NAIVE SUBJECTS WITHOUT ATOPIC DISEASE | N | 2 |
| POX-MVA-008-2001-016 | FOLLOW-UP VISIT | 50 | 1 | HEALTHY, VACCINIA NAIVE SUBJECTS WITHOUT ATOPIC DISEASE | N | 2 |
| POX-MVA-008-2001-022 | VISIT 1 | 1 | 1 | VACCINIA NAIVE SUBJECTS WITH ACTIVE ATOPIC DERMATITIS | N | 2 |
| POX-MVA-008-2001-022 | VISIT 2 | 1 | 1 | VACCINIA NAIVE SUBJECTS WITH ACTIVE ATOPIC DERMATITIS | N | 2 |
| POX-MVA-008-2001-022 | VISIT 3 | 1 | 1 | VACCINIA NAIVE SUBJECTS WITH ACTIVE ATOPIC DERMATITIS | N | 2 |
| POX-MVA-008-2001-022 | VISIT 4 | 224 | 15 | VACCINIA NAIVE SUBJECTS WITH ACTIVE ATOPIC DERMATITIS | N | 2 |
| POX-MVA-008-2001-022 | VISIT 5 | 100 | 15 | VACCINIA NAIVE SUBJECTS WITH ACTIVE ATOPIC DERMATITIS | N | 2 |
| POX-MVA-008-2001-022 | FOLLOW-UP VISIT | 1 | 15 | VACCINIA NAIVE SUBJECTS WITH ACTIVE ATOPIC DERMATITIS | N | 2 |
| POX-MVA-008-2001-036 | VISIT 1 | 1 | 1 | HEALTHY, VACCINIA NAIVE SUBJECTS WITHOUT ATOPIC DISEASE | N | 2 |
| POX-MVA-008-2001-036 | VISIT 2 | 1 | 1 | HEALTHY, VACCINIA NAIVE SUBJECTS WITHOUT ATOPIC DISEASE | N | 2 |
| POX-MVA-008-2001-036 | VISIT 3 | 219 | 1 | HEALTHY, VACCINIA NAIVE SUBJECTS WITHOUT ATOPIC DISEASE | N | 2 |
| POX-MVA-008-2001-036 | VISIT 4 | 1576 | 379 | HEALTHY, VACCINIA NAIVE SUBJECTS WITHOUT ATOPIC DISEASE | N | 2 |
| POX-MVA-008-2001-036 | VISIT 5 | 1700 | 247 | HEALTHY, VACCINIA NAIVE SUBJECTS WITHOUT ATOPIC DISEASE | N | 2 |
| POX-MVA-008-2001-041 | VISIT 1 | 1 | 1 | VACCINIA NAIVE SUBJECTS WITH ACTIVE ATOPIC DERMATITIS | N | 2 |
| POX-MVA-008-2001-041 | VISIT 2 | 100 | 1 | VACCINIA NAIVE SUBJECTS WITH ACTIVE ATOPIC DERMATITIS | N | 2 |
| POX-MVA-008-2001-041 | VISIT 3 | 100 | 15 | VACCINIA NAIVE SUBJECTS WITH ACTIVE ATOPIC DERMATITIS | N | 2 |
| POX-MVA-008-2001-041 | VISIT 4 | 1459 | 584 | VACCINIA NAIVE SUBJECTS WITH ACTIVE ATOPIC DERMATITIS | N | 2 |
| POX-MVA-008-2001-041 | VISIT 5 | 612 | 113 | VACCINIA NAIVE SUBJECTS WITH ACTIVE ATOPIC DERMATITIS | N | 2 |
| POX-MVA-008-2001-041 | FOLLOW-UP VISIT | 100 | 1 | VACCINIA NAIVE SUBJECTS WITH ACTIVE ATOPIC DERMATITIS | N | 2 |
| POX-MVA-008-2001-042 | VISIT 1 | 1 | 1 | VACCINIA NAIVE SUBJECTS WITH ACTIVE ATOPIC DERMATITIS | N | 2 |
| POX-MVA-008-2001-042 | VISIT 2 | 100 | 1 | VACCINIA NAIVE SUBJECTS WITH ACTIVE ATOPIC DERMATITIS | N | 2 |
| POX-MVA-008-2001-042 | VISIT 3 | 50 | 1 | VACCINIA NAIVE SUBJECTS WITH ACTIVE ATOPIC DERMATITIS | N | 2 |
| POX-MVA-008-2001-042 | VISIT 4 | 647 | 420 | VACCINIA NAIVE SUBJECTS WITH ACTIVE ATOPIC DERMATITIS | N | 2 |
| POX-MVA-008-2001-042 | VISIT 5 | 656 | 144 | VACCINIA NAIVE SUBJECTS WITH ACTIVE ATOPIC DERMATITIS | N | 2 |
| POX-MVA-008-2001-042 | FOLLOW-UP VISIT | 50 | 1 | VACCINIA NAIVE SUBJECTS WITH ACTIVE ATOPIC DERMATITIS | N | 2 |
| POX-MVA-008-2001-044 | VISIT 1 | 50 | 1 | VACCINIA NAIVE SUBJECTS WITH ACTIVE ATOPIC DERMATITIS | N | 2 |
| POX-MVA-008-2001-044 | VISIT 2 | 258 | 1 | VACCINIA NAIVE SUBJECTS WITH ACTIVE ATOPIC DERMATITIS | N | 2 |
| POX-MVA-008-2001-044 | VISIT 3 | 245 | 1 | VACCINIA NAIVE SUBJECTS WITH ACTIVE ATOPIC DERMATITIS | N | 2 |
| POX-MVA-008-2001-044 | VISIT 4 | 998 | 15 | VACCINIA NAIVE SUBJECTS WITH ACTIVE ATOPIC DERMATITIS | N | 2 |
| POX-MVA-008-2001-044 | VISIT 5 | 2080 | 15 | VACCINIA NAIVE SUBJECTS WITH ACTIVE ATOPIC DERMATITIS | N | 2 |
| POX-MVA-008-2001-044 | FOLLOW-UP VISIT | 50 | 15 | VACCINIA NAIVE SUBJECTS WITH ACTIVE ATOPIC DERMATITIS | N | 2 |
| POX-MVA-008-2002-002 | VISIT 1 | 50 | 1 | VACCINIA NAIVE SUBJECTS WITH ACTIVE ATOPIC DERMATITIS | N | 2 |
| POX-MVA-008-2002-002 | VISIT 2 | 1 | 1 | VACCINIA NAIVE SUBJECTS WITH ACTIVE ATOPIC DERMATITIS | N | 2 |
| POX-MVA-008-2002-002 | VISIT 3 | 1 | 1 | VACCINIA NAIVE SUBJECTS WITH ACTIVE ATOPIC DERMATITIS | N | 2 |
| POX-MVA-008-2002-002 | VISIT 4 | 100 | 15 | VACCINIA NAIVE SUBJECTS WITH ACTIVE ATOPIC DERMATITIS | N | 2 |
| POX-MVA-008-2002-002 | VISIT 5 | 50 | 1 | VACCINIA NAIVE SUBJECTS WITH ACTIVE ATOPIC DERMATITIS | N | 2 |
| POX-MVA-008-2002-002 | FOLLOW-UP VISIT | 1 | 1 | VACCINIA NAIVE SUBJECTS WITH ACTIVE ATOPIC DERMATITIS | N | 2 |
| POX-MVA-008-2002-004 | VISIT 1 | 1 | 1 | VACCINIA NAIVE SUBJECTS WITH ACTIVE ATOPIC DERMATITIS | N | 2 |
| POX-MVA-008-2002-004 | VISIT 2 | 1 | 1 | VACCINIA NAIVE SUBJECTS WITH ACTIVE ATOPIC DERMATITIS | N | 2 |
| POX-MVA-008-2002-004 | VISIT 3 | 50 | 1 | VACCINIA NAIVE SUBJECTS WITH ACTIVE ATOPIC DERMATITIS | N | 2 |
| POX-MVA-008-2002-004 | VISIT 4 | 282 | 1 | VACCINIA NAIVE SUBJECTS WITH ACTIVE ATOPIC DERMATITIS | N | 2 |
| POX-MVA-008-2002-004 | VISIT 5 | 294 | 15 | VACCINIA NAIVE SUBJECTS WITH ACTIVE ATOPIC DERMATITIS | N | 2 |
| POX-MVA-008-2002-004 | FOLLOW-UP VISIT | 1 | 1 | VACCINIA NAIVE SUBJECTS WITH ACTIVE ATOPIC DERMATITIS | N | 2 |
| POX-MVA-008-2002-005 | VISIT 1 | 1 | 1 | VACCINIA NAIVE SUBJECTS WITH ACTIVE ATOPIC DERMATITIS | N | 2 |
| POX-MVA-008-2002-005 | VISIT 2 | 1 | 1 | VACCINIA NAIVE SUBJECTS WITH ACTIVE ATOPIC DERMATITIS | N | 2 |
| POX-MVA-008-2002-005 | VISIT 3 | 1 | . | VACCINIA NAIVE SUBJECTS WITH ACTIVE ATOPIC DERMATITIS | N | 2 |
| POX-MVA-008-2002-005 | VISIT 4 | 50 | 1 | VACCINIA NAIVE SUBJECTS WITH ACTIVE ATOPIC DERMATITIS | N | 2 |
| POX-MVA-008-2002-005 | VISIT 5 | 100 | 1 | VACCINIA NAIVE SUBJECTS WITH ACTIVE ATOPIC DERMATITIS | N | 2 |
| POX-MVA-008-2002-005 | FOLLOW-UP VISIT | 50 | 1 | VACCINIA NAIVE SUBJECTS WITH ACTIVE ATOPIC DERMATITIS | N | 2 |
| POX-MVA-008-2002-006 | VISIT 1 | 1 | 1 | VACCINIA NAIVE SUBJECTS WITH ACTIVE ATOPIC DERMATITIS | N | 2 |
| POX-MVA-008-2002-006 | VISIT 2 | 1 | 1 | VACCINIA NAIVE SUBJECTS WITH ACTIVE ATOPIC DERMATITIS | N | 2 |
| POX-MVA-008-2002-006 | VISIT 3 | 1 | 1 | VACCINIA NAIVE SUBJECTS WITH ACTIVE ATOPIC DERMATITIS | N | 2 |
| POX-MVA-008-2002-006 | UNSCHEDULED VISIT 4A | 100 | 15 | VACCINIA NAIVE SUBJECTS WITH ACTIVE ATOPIC DERMATITIS | N | 2 |
| POX-MVA-008-2002-006 | VISIT 5 | 100 | 1 | VACCINIA NAIVE SUBJECTS WITH ACTIVE ATOPIC DERMATITIS | N | 2 |
| POX-MVA-008-2002-006 | FOLLOW-UP VISIT | 50 | 1 | VACCINIA NAIVE SUBJECTS WITH ACTIVE ATOPIC DERMATITIS | N | 2 |
| POX-MVA-008-2002-007 | VISIT 1 | 1 | 1 | VACCINIA NAIVE SUBJECTS WITH HISTORY OF ATOPIC DERMATITIS | N | 2 |
| POX-MVA-008-2002-007 | VISIT 2 | 1 | 1 | VACCINIA NAIVE SUBJECTS WITH HISTORY OF ATOPIC DERMATITIS | N | 2 |
| POX-MVA-008-2002-007 | VISIT 3 | 1 | 1 | VACCINIA NAIVE SUBJECTS WITH HISTORY OF ATOPIC DERMATITIS | N | 2 |
| POX-MVA-008-2002-007 | VISIT 4 | 250 | 1 | VACCINIA NAIVE SUBJECTS WITH HISTORY OF ATOPIC DERMATITIS | N | 2 |
| POX-MVA-008-2002-007 | VISIT 5 | 260 | 1 | VACCINIA NAIVE SUBJECTS WITH HISTORY OF ATOPIC DERMATITIS | N | 2 |
| POX-MVA-008-2002-007 | FOLLOW-UP VISIT | 1 | 1 | VACCINIA NAIVE SUBJECTS WITH HISTORY OF ATOPIC DERMATITIS | N | 2 |
| POX-MVA-008-2002-008 | VISIT 1 | 1 | 1 | VACCINIA NAIVE SUBJECTS WITH ACTIVE ATOPIC DERMATITIS | N | 2 |
| POX-MVA-008-2002-008 | VISIT 2 | 1 | 1 | VACCINIA NAIVE SUBJECTS WITH ACTIVE ATOPIC DERMATITIS | N | 2 |
| POX-MVA-008-2002-008 | VISIT 3 | 100 | 1 | VACCINIA NAIVE SUBJECTS WITH ACTIVE ATOPIC DERMATITIS | N | 2 |
| POX-MVA-008-2002-008 | VISIT 4 | 236 | 1 | VACCINIA NAIVE SUBJECTS WITH ACTIVE ATOPIC DERMATITIS | N | 2 |
| POX-MVA-008-2002-008 | VISIT 5 | 200 | 1 | VACCINIA NAIVE SUBJECTS WITH ACTIVE ATOPIC DERMATITIS | N | 2 |
| POX-MVA-008-2002-008 | FOLLOW-UP VISIT | 1 | 1 | VACCINIA NAIVE SUBJECTS WITH ACTIVE ATOPIC DERMATITIS | N | 2 |
| POX-MVA-008-2002-041 | VISIT 1 | 1 | 1 | VACCINIA NAIVE SUBJECTS WITH HISTORY OF ATOPIC DERMATITIS | N | 2 |
| POX-MVA-008-2002-041 | VISIT 2 | 1 | 1 | VACCINIA NAIVE SUBJECTS WITH HISTORY OF ATOPIC DERMATITIS | N | 2 |
| POX-MVA-008-2002-041 | VISIT 3 | 50 | 1 | VACCINIA NAIVE SUBJECTS WITH HISTORY OF ATOPIC DERMATITIS | N | 2 |
| POX-MVA-008-2002-041 | VISIT 4 | 328 | 15 | VACCINIA NAIVE SUBJECTS WITH HISTORY OF ATOPIC DERMATITIS | N | 2 |
| POX-MVA-008-2002-041 | VISIT 5 | 421 | 105 | VACCINIA NAIVE SUBJECTS WITH HISTORY OF ATOPIC DERMATITIS | N | 2 |
| POX-MVA-008-2002-041 | FOLLOW-UP VISIT | 100 | 15 | VACCINIA NAIVE SUBJECTS WITH HISTORY OF ATOPIC DERMATITIS | N | 2 |
| POX-MVA-008-2002-054 | VISIT 1 | 1 | 1 | HEALTHY, VACCINIA NAIVE SUBJECTS WITHOUT ATOPIC DISEASE | N | 2 |
| POX-MVA-008-2002-054 | VISIT 2 | 50 | 1 | HEALTHY, VACCINIA NAIVE SUBJECTS WITHOUT ATOPIC DISEASE | N | 2 |
| POX-MVA-008-2002-054 | VISIT 3 | 100 | 1 | HEALTHY, VACCINIA NAIVE SUBJECTS WITHOUT ATOPIC DISEASE | N | 2 |
| POX-MVA-008-2002-054 | VISIT 4 | 1208 | 864 | HEALTHY, VACCINIA NAIVE SUBJECTS WITHOUT ATOPIC DISEASE | N | 2 |
| POX-MVA-008-2002-054 | VISIT 5 | 1796 | 619 | HEALTHY, VACCINIA NAIVE SUBJECTS WITHOUT ATOPIC DISEASE | N | 2 |
| POX-MVA-008-2002-058 | VISIT 1 | 1 | 1 | VACCINIA NAIVE SUBJECTS WITH ACTIVE ATOPIC DERMATITIS | N | 2 |
| POX-MVA-008-2002-058 | VISIT 2 | 1 | 1 | VACCINIA NAIVE SUBJECTS WITH ACTIVE ATOPIC DERMATITIS | N | 2 |
| POX-MVA-008-2002-058 | VISIT 3 | 50 | 1 | VACCINIA NAIVE SUBJECTS WITH ACTIVE ATOPIC DERMATITIS | N | 2 |
| POX-MVA-008-2002-058 | VISIT 4 | 515 | 162 | VACCINIA NAIVE SUBJECTS WITH ACTIVE ATOPIC DERMATITIS | N | 2 |
| POX-MVA-008-2002-058 | VISIT 5 | 213 | 101 | VACCINIA NAIVE SUBJECTS WITH ACTIVE ATOPIC DERMATITIS | N | 2 |
| POX-MVA-008-2002-063 | VISIT 1 | 1 | 1 | VACCINIA NAIVE SUBJECTS WITH HISTORY OF ATOPIC DERMATITIS | N | 2 |
| POX-MVA-008-2002-063 | VISIT 2 | 1 | 1 | VACCINIA NAIVE SUBJECTS WITH HISTORY OF ATOPIC DERMATITIS | N | 2 |
| POX-MVA-008-2002-063 | VISIT 3 | 100 | 15 | VACCINIA NAIVE SUBJECTS WITH HISTORY OF ATOPIC DERMATITIS | N | 2 |
| POX-MVA-008-2002-063 | VISIT 4 | 689 | 90 | VACCINIA NAIVE SUBJECTS WITH HISTORY OF ATOPIC DERMATITIS | N | 2 |
| POX-MVA-008-2002-063 | VISIT 5 | 324 | 1 | VACCINIA NAIVE SUBJECTS WITH HISTORY OF ATOPIC DERMATITIS | N | 2 |
| POX-MVA-008-2002-070 | VISIT 1 | 1 | 1 | VACCINIA NAIVE SUBJECTS WITH HISTORY OF ATOPIC DERMATITIS | N | 2 |
| POX-MVA-008-2002-070 | VISIT 2 | 1 | 1 | VACCINIA NAIVE SUBJECTS WITH HISTORY OF ATOPIC DERMATITIS | N | 2 |
| POX-MVA-008-2002-070 | VISIT 3 | 100 | 1 | VACCINIA NAIVE SUBJECTS WITH HISTORY OF ATOPIC DERMATITIS | N | 2 |
| POX-MVA-008-2002-070 | VISIT 4 | 5752 | 248 | VACCINIA NAIVE SUBJECTS WITH HISTORY OF ATOPIC DERMATITIS | N | 2 |
| POX-MVA-008-2002-070 | VISIT 5 | 2798 | 114 | VACCINIA NAIVE SUBJECTS WITH HISTORY OF ATOPIC DERMATITIS | N | 2 |
| POX-MVA-008-2002-071 | VISIT 1 | 1 | 1 | VACCINIA NAIVE SUBJECTS WITH ACTIVE ATOPIC DERMATITIS | N | 2 |
| POX-MVA-008-2002-071 | VISIT 2 | 1 | 1 | VACCINIA NAIVE SUBJECTS WITH ACTIVE ATOPIC DERMATITIS | N | 2 |
| POX-MVA-008-2002-071 | VISIT 3 | 100 | 1 | VACCINIA NAIVE SUBJECTS WITH ACTIVE ATOPIC DERMATITIS | N | 2 |
| POX-MVA-008-2002-071 | VISIT 4 | 579 | 15 | VACCINIA NAIVE SUBJECTS WITH ACTIVE ATOPIC DERMATITIS | N | 2 |
| POX-MVA-008-2002-071 | VISIT 5 | 278 | 15 | VACCINIA NAIVE SUBJECTS WITH ACTIVE ATOPIC DERMATITIS | N | 2 |
| POX-MVA-008-2002-071 | FOLLOW-UP VISIT | 1 | 1 | VACCINIA NAIVE SUBJECTS WITH ACTIVE ATOPIC DERMATITIS | N | 2 |
| POX-MVA-008-2002-076 | VISIT 1 | 1 | 1 | VACCINIA NAIVE SUBJECTS WITH ACTIVE ATOPIC DERMATITIS | N | 1 |
| POX-MVA-008-2002-076 | VISIT 2 | 1 | 1 | VACCINIA NAIVE SUBJECTS WITH ACTIVE ATOPIC DERMATITIS | N | 1 |
| POX-MVA-008-2003-006 | VISIT 1 | 1 | 1 | HEALTHY, VACCINIA NAIVE SUBJECTS WITHOUT ATOPIC DISEASE | N | 2 |
| POX-MVA-008-2003-006 | VISIT 2 | 1 | 1 | HEALTHY, VACCINIA NAIVE SUBJECTS WITHOUT ATOPIC DISEASE | N | 2 |
| POX-MVA-008-2003-006 | VISIT 3 | 200 | 1 | HEALTHY, VACCINIA NAIVE SUBJECTS WITHOUT ATOPIC DISEASE | N | 2 |
| POX-MVA-008-2003-006 | VISIT 4 | 1619 | 15 | HEALTHY, VACCINIA NAIVE SUBJECTS WITHOUT ATOPIC DISEASE | N | 2 |
| POX-MVA-008-2003-006 | VISIT 5 | 1588 | 15 | HEALTHY, VACCINIA NAIVE SUBJECTS WITHOUT ATOPIC DISEASE | N | 2 |
| POX-MVA-008-2003-006 | FOLLOW-UP VISIT | 100 | 1 | HEALTHY, VACCINIA NAIVE SUBJECTS WITHOUT ATOPIC DISEASE | N | 2 |
| POX-MVA-008-2003-010 | VISIT 1 | 1 | 1 | HEALTHY, VACCINIA NAIVE SUBJECTS WITHOUT ATOPIC DISEASE | N | 2 |
| POX-MVA-008-2003-010 | VISIT 2 | 1 | 1 | HEALTHY, VACCINIA NAIVE SUBJECTS WITHOUT ATOPIC DISEASE | N | 2 |
| POX-MVA-008-2003-010 | VISIT 3 | 50 | 1 | HEALTHY, VACCINIA NAIVE SUBJECTS WITHOUT ATOPIC DISEASE | N | 2 |
| POX-MVA-008-2003-010 | VISIT 5 | 100 | 1 | HEALTHY, VACCINIA NAIVE SUBJECTS WITHOUT ATOPIC DISEASE | N | 2 |
| POX-MVA-008-2003-010 | FOLLOW-UP VISIT | 100 | 1393 | HEALTHY, VACCINIA NAIVE SUBJECTS WITHOUT ATOPIC DISEASE | N | 2 |
| POX-MVA-008-2003-011 | VISIT 1 | 1 | 1 | HEALTHY, VACCINIA NAIVE SUBJECTS WITHOUT ATOPIC DISEASE | N | 2 |
| POX-MVA-008-2003-011 | VISIT 2 | 50 | 1 | HEALTHY, VACCINIA NAIVE SUBJECTS WITHOUT ATOPIC DISEASE | N | 2 |
| POX-MVA-008-2003-011 | VISIT 3 | 100 | 1 | HEALTHY, VACCINIA NAIVE SUBJECTS WITHOUT ATOPIC DISEASE | N | 2 |
| POX-MVA-008-2003-011 | VISIT 5 | 386 | 15 | HEALTHY, VACCINIA NAIVE SUBJECTS WITHOUT ATOPIC DISEASE | N | 2 |
| POX-MVA-008-2003-011 | FOLLOW-UP VISIT | 1 | 1 | HEALTHY, VACCINIA NAIVE SUBJECTS WITHOUT ATOPIC DISEASE | N | 2 |
| POX-MVA-008-2004-001 | VISIT 1 | 1 | 1 | VACCINIA NAIVE SUBJECTS WITH ACTIVE ATOPIC DERMATITIS | N | 2 |
| POX-MVA-008-2004-001 | VISIT 2 | 1 | 1 | VACCINIA NAIVE SUBJECTS WITH ACTIVE ATOPIC DERMATITIS | N | 2 |
| POX-MVA-008-2004-001 | VISIT 3 | 1 | 1 | VACCINIA NAIVE SUBJECTS WITH ACTIVE ATOPIC DERMATITIS | N | 2 |
| POX-MVA-008-2004-001 | VISIT 4 | 3705 | 293 | VACCINIA NAIVE SUBJECTS WITH ACTIVE ATOPIC DERMATITIS | N | 2 |
| POX-MVA-008-2004-001 | VISIT 5 | 451 | 155 | VACCINIA NAIVE SUBJECTS WITH ACTIVE ATOPIC DERMATITIS | N | 2 |
| POX-MVA-008-2004-001 | FOLLOW-UP VISIT | 100 | 15 | VACCINIA NAIVE SUBJECTS WITH ACTIVE ATOPIC DERMATITIS | N | 2 |
| POX-MVA-008-2004-002 | VISIT 1 | 1 | 1 | VACCINIA NAIVE SUBJECTS WITH ACTIVE ATOPIC DERMATITIS | N | 2 |
| POX-MVA-008-2004-002 | VISIT 2 | 1 | 1 | VACCINIA NAIVE SUBJECTS WITH ACTIVE ATOPIC DERMATITIS | N | 2 |
| POX-MVA-008-2004-002 | VISIT 3 | 50 | 1 | VACCINIA NAIVE SUBJECTS WITH ACTIVE ATOPIC DERMATITIS | N | 2 |
| POX-MVA-008-2004-002 | VISIT 4 | 50 | 1 | VACCINIA NAIVE SUBJECTS WITH ACTIVE ATOPIC DERMATITIS | N | 2 |
| POX-MVA-008-2004-002 | VISIT 5 | 294 | 15 | VACCINIA NAIVE SUBJECTS WITH ACTIVE ATOPIC DERMATITIS | N | 2 |
| POX-MVA-008-2004-002 | FOLLOW-UP VISIT | 1 | 1 | VACCINIA NAIVE SUBJECTS WITH ACTIVE ATOPIC DERMATITIS | N | 2 |
| POX-MVA-008-2004-004 | VISIT 1 | 1 | 1 | VACCINIA NAIVE SUBJECTS WITH HISTORY OF ATOPIC DERMATITIS | N | 2 |
| POX-MVA-008-2004-004 | VISIT 2 | 388 | 15 | VACCINIA NAIVE SUBJECTS WITH HISTORY OF ATOPIC DERMATITIS | N | 2 |
| POX-MVA-008-2004-004 | VISIT 3 | 463 | 15 | VACCINIA NAIVE SUBJECTS WITH HISTORY OF ATOPIC DERMATITIS | N | 2 |
| POX-MVA-008-2004-004 | VISIT 4 | 937 | 94 | VACCINIA NAIVE SUBJECTS WITH HISTORY OF ATOPIC DERMATITIS | N | 2 |
| POX-MVA-008-2004-004 | VISIT 5 | 993 | 102 | VACCINIA NAIVE SUBJECTS WITH HISTORY OF ATOPIC DERMATITIS | N | 2 |
| POX-MVA-008-2004-004 | FOLLOW-UP VISIT | 342 | 15 | VACCINIA NAIVE SUBJECTS WITH HISTORY OF ATOPIC DERMATITIS | N | 2 |
| POX-MVA-008-2004-005 | VISIT 1 | 1 | 1 | HEALTHY, VACCINIA NAIVE SUBJECTS WITHOUT ATOPIC DISEASE | N | 2 |
| POX-MVA-008-2004-005 | VISIT 2 | 1 | 1 | HEALTHY, VACCINIA NAIVE SUBJECTS WITHOUT ATOPIC DISEASE | N | 2 |
| POX-MVA-008-2004-005 | VISIT 3 | 50 | 1 | HEALTHY, VACCINIA NAIVE SUBJECTS WITHOUT ATOPIC DISEASE | N | 2 |
| POX-MVA-008-2004-005 | VISIT 4 | 952 | 15 | HEALTHY, VACCINIA NAIVE SUBJECTS WITHOUT ATOPIC DISEASE | N | 2 |
| POX-MVA-008-2004-005 | VISIT 5 | 721 | 15 | HEALTHY, VACCINIA NAIVE SUBJECTS WITHOUT ATOPIC DISEASE | N | 2 |
| POX-MVA-008-2004-005 | FOLLOW-UP VISIT | 100 | 1 | HEALTHY, VACCINIA NAIVE SUBJECTS WITHOUT ATOPIC DISEASE | N | 2 |
| POX-MVA-008-2004-006 | VISIT 1 | 1 | 1 | HEALTHY, VACCINIA NAIVE SUBJECTS WITHOUT ATOPIC DISEASE | N | 2 |
| POX-MVA-008-2004-006 | VISIT 2 | 1 | 1 | HEALTHY, VACCINIA NAIVE SUBJECTS WITHOUT ATOPIC DISEASE | N | 2 |
| POX-MVA-008-2004-006 | VISIT 3 | 1 | 1 | HEALTHY, VACCINIA NAIVE SUBJECTS WITHOUT ATOPIC DISEASE | N | 2 |
| POX-MVA-008-2004-006 | VISIT 4 | 381 | 15 | HEALTHY, VACCINIA NAIVE SUBJECTS WITHOUT ATOPIC DISEASE | N | 2 |
| POX-MVA-008-2004-006 | VISIT 5 | 235 | 15 | HEALTHY, VACCINIA NAIVE SUBJECTS WITHOUT ATOPIC DISEASE | N | 2 |
| POX-MVA-008-2004-006 | FOLLOW-UP VISIT | 1 | 1 | HEALTHY, VACCINIA NAIVE SUBJECTS WITHOUT ATOPIC DISEASE | N | 2 |
| POX-MVA-008-2004-008 | VISIT 1 | 1 | 1 | VACCINIA NAIVE SUBJECTS WITH HISTORY OF ATOPIC DERMATITIS | N | 2 |
| POX-MVA-008-2004-008 | VISIT 2 | 1 | 1 | VACCINIA NAIVE SUBJECTS WITH HISTORY OF ATOPIC DERMATITIS | N | 2 |
| POX-MVA-008-2004-008 | VISIT 3 | 1 | 1 | VACCINIA NAIVE SUBJECTS WITH HISTORY OF ATOPIC DERMATITIS | N | 2 |
| POX-MVA-008-2004-008 | VISIT 4 | 828 | 1 | VACCINIA NAIVE SUBJECTS WITH HISTORY OF ATOPIC DERMATITIS | N | 2 |
| POX-MVA-008-2004-008 | VISIT 5 | 355 | 15 | VACCINIA NAIVE SUBJECTS WITH HISTORY OF ATOPIC DERMATITIS | N | 2 |
| POX-MVA-008-2004-008 | FOLLOW-UP VISIT | 1 | 1 | VACCINIA NAIVE SUBJECTS WITH HISTORY OF ATOPIC DERMATITIS | N | 2 |
| POX-MVA-008-2004-009 | VISIT 1 | 50 | 1 | HEALTHY, VACCINIA NAIVE SUBJECTS WITHOUT ATOPIC DISEASE | N | 2 |
| POX-MVA-008-2004-009 | VISIT 2 | 444 | 104 | HEALTHY, VACCINIA NAIVE SUBJECTS WITHOUT ATOPIC DISEASE | N | 2 |
| POX-MVA-008-2004-009 | VISIT 3 | 261 | 15 | HEALTHY, VACCINIA NAIVE SUBJECTS WITHOUT ATOPIC DISEASE | N | 2 |
| POX-MVA-008-2004-009 | VISIT 4 | 333 | 79 | HEALTHY, VACCINIA NAIVE SUBJECTS WITHOUT ATOPIC DISEASE | N | 2 |
| POX-MVA-008-2004-009 | VISIT 5 | 309 | 15 | HEALTHY, VACCINIA NAIVE SUBJECTS WITHOUT ATOPIC DISEASE | N | 2 |
| POX-MVA-008-2004-009 | FOLLOW-UP VISIT | 100 | 1 | HEALTHY, VACCINIA NAIVE SUBJECTS WITHOUT ATOPIC DISEASE | N | 2 |
| POX-MVA-008-2004-011 | VISIT 1 | 100 | 15 | VACCINIA NAIVE SUBJECTS WITH HISTORY OF ATOPIC DERMATITIS | N | 2 |
| POX-MVA-008-2004-011 | VISIT 2 | 786 | 280 | VACCINIA NAIVE SUBJECTS WITH HISTORY OF ATOPIC DERMATITIS | N | 2 |
| POX-MVA-008-2004-011 | VISIT 3 | 292 | 80 | VACCINIA NAIVE SUBJECTS WITH HISTORY OF ATOPIC DERMATITIS | N | 2 |
| POX-MVA-008-2004-011 | VISIT 4 | 343 | 253 | VACCINIA NAIVE SUBJECTS WITH HISTORY OF ATOPIC DERMATITIS | N | 2 |
| POX-MVA-008-2004-011 | VISIT 5 | 273 | 129 | VACCINIA NAIVE SUBJECTS WITH HISTORY OF ATOPIC DERMATITIS | N | 2 |
| POX-MVA-008-2004-011 | FOLLOW-UP VISIT | 209 | 76 | VACCINIA NAIVE SUBJECTS WITH HISTORY OF ATOPIC DERMATITIS | N | 2 |
| POX-MVA-008-2004-012 | VISIT 1 | 1 | 1 | VACCINIA NAIVE SUBJECTS WITH ACTIVE ATOPIC DERMATITIS | N | 2 |
| POX-MVA-008-2004-012 | VISIT 2 | 1 | 1 | VACCINIA NAIVE SUBJECTS WITH ACTIVE ATOPIC DERMATITIS | N | 2 |
| POX-MVA-008-2004-012 | VISIT 3 | 50 | 1 | VACCINIA NAIVE SUBJECTS WITH ACTIVE ATOPIC DERMATITIS | N | 2 |
| POX-MVA-008-2004-012 | VISIT 4 | 206 | 1 | VACCINIA NAIVE SUBJECTS WITH ACTIVE ATOPIC DERMATITIS | N | 2 |
| POX-MVA-008-2004-012 | VISIT 5 | 100 | 1 | VACCINIA NAIVE SUBJECTS WITH ACTIVE ATOPIC DERMATITIS | N | 2 |
| POX-MVA-008-2004-012 | FOLLOW-UP VISIT | 50 | 1 | VACCINIA NAIVE SUBJECTS WITH ACTIVE ATOPIC DERMATITIS | N | 2 |
| POX-MVA-008-2004-013 | VISIT 1 | 1 | 1 | VACCINIA NAIVE SUBJECTS WITH HISTORY OF ATOPIC DERMATITIS | N | 2 |
| POX-MVA-008-2004-013 | VISIT 2 | 1 | 1 | VACCINIA NAIVE SUBJECTS WITH HISTORY OF ATOPIC DERMATITIS | N | 2 |
| POX-MVA-008-2004-013 | VISIT 3 | 100 | 1 | VACCINIA NAIVE SUBJECTS WITH HISTORY OF ATOPIC DERMATITIS | N | 2 |
| POX-MVA-008-2004-013 | VISIT 4 | 491 | 15 | VACCINIA NAIVE SUBJECTS WITH HISTORY OF ATOPIC DERMATITIS | N | 2 |
| POX-MVA-008-2004-013 | VISIT 5 | 379 | 15 | VACCINIA NAIVE SUBJECTS WITH HISTORY OF ATOPIC DERMATITIS | N | 2 |
| POX-MVA-008-2004-013 | FOLLOW-UP VISIT | 50 | 1 | VACCINIA NAIVE SUBJECTS WITH HISTORY OF ATOPIC DERMATITIS | N | 2 |
| POX-MVA-008-2004-014 | VISIT 1 | 1 | 1 | VACCINIA NAIVE SUBJECTS WITH HISTORY OF ATOPIC DERMATITIS | N | 2 |
| POX-MVA-008-2004-014 | VISIT 2 | 1 | 1 | VACCINIA NAIVE SUBJECTS WITH HISTORY OF ATOPIC DERMATITIS | N | 2 |
| POX-MVA-008-2004-014 | VISIT 3 | 50 | 1 | VACCINIA NAIVE SUBJECTS WITH HISTORY OF ATOPIC DERMATITIS | N | 2 |
| POX-MVA-008-2004-014 | VISIT 4 | 1009 | 15 | VACCINIA NAIVE SUBJECTS WITH HISTORY OF ATOPIC DERMATITIS | N | 2 |
| POX-MVA-008-2004-014 | VISIT 5 | 293 | 1 | VACCINIA NAIVE SUBJECTS WITH HISTORY OF ATOPIC DERMATITIS | N | 2 |
| POX-MVA-008-2004-014 | FOLLOW-UP VISIT | 100 | 1 | VACCINIA NAIVE SUBJECTS WITH HISTORY OF ATOPIC DERMATITIS | N | 2 |
| POX-MVA-008-2004-015 | VISIT 1 | 1 | 1 | HEALTHY, VACCINIA NAIVE SUBJECTS WITHOUT ATOPIC DISEASE | N | 2 |
| POX-MVA-008-2004-015 | VISIT 2 | 1 | 1 | HEALTHY, VACCINIA NAIVE SUBJECTS WITHOUT ATOPIC DISEASE | N | 2 |
| POX-MVA-008-2004-015 | VISIT 3 | 1 | 1 | HEALTHY, VACCINIA NAIVE SUBJECTS WITHOUT ATOPIC DISEASE | N | 2 |
| POX-MVA-008-2004-015 | VISIT 4 | 224 | 15 | HEALTHY, VACCINIA NAIVE SUBJECTS WITHOUT ATOPIC DISEASE | N | 2 |
| POX-MVA-008-2004-015 | VISIT 5 | 220 | 1 | HEALTHY, VACCINIA NAIVE SUBJECTS WITHOUT ATOPIC DISEASE | N | 2 |
| POX-MVA-008-2004-015 | FOLLOW-UP VISIT | 1 | 1 | HEALTHY, VACCINIA NAIVE SUBJECTS WITHOUT ATOPIC DISEASE | N | 2 |
| POX-MVA-008-2004-022 | VISIT 1 | 1 | 1 | VACCINIA NAIVE SUBJECTS WITH ACTIVE ATOPIC DERMATITIS | N | 2 |
| POX-MVA-008-2004-022 | VISIT 2 | 1 | 1 | VACCINIA NAIVE SUBJECTS WITH ACTIVE ATOPIC DERMATITIS | N | 2 |
| POX-MVA-008-2004-022 | VISIT 3 | 50 | 1 | VACCINIA NAIVE SUBJECTS WITH ACTIVE ATOPIC DERMATITIS | N | 2 |
| POX-MVA-008-2004-022 | VISIT 4 | 446 | 81 | VACCINIA NAIVE SUBJECTS WITH ACTIVE ATOPIC DERMATITIS | N | 2 |
| POX-MVA-008-2004-022 | VISIT 5 | 213 | 180 | VACCINIA NAIVE SUBJECTS WITH ACTIVE ATOPIC DERMATITIS | N | 2 |
| POX-MVA-008-2004-022 | FOLLOW-UP VISIT | 50 | 1 | VACCINIA NAIVE SUBJECTS WITH ACTIVE ATOPIC DERMATITIS | N | 2 |
| POX-MVA-008-2004-023 | VISIT 1 | 50 | 1 | VACCINIA NAIVE SUBJECTS WITH HISTORY OF ATOPIC DERMATITIS | N | 1 |
| POX-MVA-008-2004-023 | VISIT 2 | 50 | 1 | VACCINIA NAIVE SUBJECTS WITH HISTORY OF ATOPIC DERMATITIS | N | 1 |
| POX-MVA-008-2004-049 | VISIT 1 | 1 | 1 | VACCINIA NAIVE SUBJECTS WITH ACTIVE ATOPIC DERMATITIS | N | 2 |
| POX-MVA-008-2004-049 | VISIT 2 | 228 | 1 | VACCINIA NAIVE SUBJECTS WITH ACTIVE ATOPIC DERMATITIS | N | 2 |
| POX-MVA-008-2004-049 | VISIT 3 | 309 | 1 | VACCINIA NAIVE SUBJECTS WITH ACTIVE ATOPIC DERMATITIS | N | 2 |
| POX-MVA-008-2004-049 | VISIT 4 | 450 | 92 | VACCINIA NAIVE SUBJECTS WITH ACTIVE ATOPIC DERMATITIS | N | 2 |
| POX-MVA-008-2004-049 | VISIT 5 | 470 | 88 | VACCINIA NAIVE SUBJECTS WITH ACTIVE ATOPIC DERMATITIS | N | 2 |
| POX-MVA-008-2005-001 | VISIT 1 | 1 | 1 | HEALTHY, VACCINIA NAIVE SUBJECTS WITHOUT ATOPIC DISEASE | N | 2 |
| POX-MVA-008-2005-001 | VISIT 2 | 1 | 1 | HEALTHY, VACCINIA NAIVE SUBJECTS WITHOUT ATOPIC DISEASE | N | 2 |
| POX-MVA-008-2005-001 | VISIT 3 | 50 | 1 | HEALTHY, VACCINIA NAIVE SUBJECTS WITHOUT ATOPIC DISEASE | N | 2 |
| POX-MVA-008-2005-001 | VISIT 4 | 290 | 1 | HEALTHY, VACCINIA NAIVE SUBJECTS WITHOUT ATOPIC DISEASE | N | 2 |
| POX-MVA-008-2005-001 | VISIT 5 | 210 | 1 | HEALTHY, VACCINIA NAIVE SUBJECTS WITHOUT ATOPIC DISEASE | N | 2 |
| POX-MVA-008-2005-002 | VISIT 1 | 1 | 1 | VACCINIA NAIVE SUBJECTS WITH ACTIVE ATOPIC DERMATITIS | N | 2 |
| POX-MVA-008-2005-002 | VISIT 2 | 50 | 1 | VACCINIA NAIVE SUBJECTS WITH ACTIVE ATOPIC DERMATITIS | N | 2 |
| POX-MVA-008-2005-002 | VISIT 3 | 100 | 1 | VACCINIA NAIVE SUBJECTS WITH ACTIVE ATOPIC DERMATITIS | N | 2 |
| POX-MVA-008-2005-002 | VISIT 4 | 449 | 15 | VACCINIA NAIVE SUBJECTS WITH ACTIVE ATOPIC DERMATITIS | N | 2 |
| POX-MVA-008-2005-002 | VISIT 5 | 454 | 1 | VACCINIA NAIVE SUBJECTS WITH ACTIVE ATOPIC DERMATITIS | N | 2 |
| POX-MVA-008-2005-003 | VISIT 1 | 50 | 15 | VACCINIA NAIVE SUBJECTS WITH ACTIVE ATOPIC DERMATITIS | N | 2 |
| POX-MVA-008-2005-003 | VISIT 2 | 201 | 15 | VACCINIA NAIVE SUBJECTS WITH ACTIVE ATOPIC DERMATITIS | N | 2 |
| POX-MVA-008-2005-003 | VISIT 3 | 293 | 80 | VACCINIA NAIVE SUBJECTS WITH ACTIVE ATOPIC DERMATITIS | N | 2 |
| POX-MVA-008-2005-003 | VISIT 4 | 268 | 112 | VACCINIA NAIVE SUBJECTS WITH ACTIVE ATOPIC DERMATITIS | N | 2 |
| POX-MVA-008-2005-003 | VISIT 5 | 223 | 93 | VACCINIA NAIVE SUBJECTS WITH ACTIVE ATOPIC DERMATITIS | N | 2 |
| POX-MVA-008-2005-004 | VISIT 1 | 1 | 1 | HEALTHY, VACCINIA NAIVE SUBJECTS WITHOUT ATOPIC DISEASE | N | 2 |
| POX-MVA-008-2005-004 | VISIT 2 | 1 | 1 | HEALTHY, VACCINIA NAIVE SUBJECTS WITHOUT ATOPIC DISEASE | N | 2 |
| POX-MVA-008-2005-004 | VISIT 3 | 50 | 1 | HEALTHY, VACCINIA NAIVE SUBJECTS WITHOUT ATOPIC DISEASE | N | 2 |
| POX-MVA-008-2005-004 | VISIT 4 | 463 | 87 | HEALTHY, VACCINIA NAIVE SUBJECTS WITHOUT ATOPIC DISEASE | N | 2 |
| POX-MVA-008-2005-004 | VISIT 5 | 290 | 1 | HEALTHY, VACCINIA NAIVE SUBJECTS WITHOUT ATOPIC DISEASE | N | 2 |
| POX-MVA-008-2005-005 | VISIT 1 | 1 | 1 | HEALTHY, VACCINIA NAIVE SUBJECTS WITHOUT ATOPIC DISEASE | N | 2 |
| POX-MVA-008-2005-005 | VISIT 2 | 1 | 1 | HEALTHY, VACCINIA NAIVE SUBJECTS WITHOUT ATOPIC DISEASE | N | 2 |
| POX-MVA-008-2005-005 | VISIT 3 | 50 | 1 | HEALTHY, VACCINIA NAIVE SUBJECTS WITHOUT ATOPIC DISEASE | N | 2 |
| POX-MVA-008-2005-005 | VISIT 4 | 639 | 15 | HEALTHY, VACCINIA NAIVE SUBJECTS WITHOUT ATOPIC DISEASE | N | 2 |
| POX-MVA-008-2005-005 | VISIT 5 | 348 | 15 | HEALTHY, VACCINIA NAIVE SUBJECTS WITHOUT ATOPIC DISEASE | N | 2 |
| POX-MVA-008-2005-006 | VISIT 1 | 1 | 1 | VACCINIA NAIVE SUBJECTS WITH ACTIVE ATOPIC DERMATITIS | N | 2 |
| POX-MVA-008-2005-006 | VISIT 2 | 1 | 1 | VACCINIA NAIVE SUBJECTS WITH ACTIVE ATOPIC DERMATITIS | N | 2 |
| POX-MVA-008-2005-006 | VISIT 3 | 100 | 1 | VACCINIA NAIVE SUBJECTS WITH ACTIVE ATOPIC DERMATITIS | N | 2 |
| POX-MVA-008-2005-006 | VISIT 4 | 780 | 15 | VACCINIA NAIVE SUBJECTS WITH ACTIVE ATOPIC DERMATITIS | N | 2 |
| POX-MVA-008-2005-006 | VISIT 5 | 301 | 1 | VACCINIA NAIVE SUBJECTS WITH ACTIVE ATOPIC DERMATITIS | N | 2 |
| POX-MVA-008-2005-007 | VISIT 1 | 1 | 1 | VACCINIA NAIVE SUBJECTS WITH ACTIVE ATOPIC DERMATITIS | N | 2 |
| POX-MVA-008-2005-007 | VISIT 2 | 1 | 1 | VACCINIA NAIVE SUBJECTS WITH ACTIVE ATOPIC DERMATITIS | N | 2 |
| POX-MVA-008-2005-007 | VISIT 3 | 50 | 1 | VACCINIA NAIVE SUBJECTS WITH ACTIVE ATOPIC DERMATITIS | N | 2 |
| POX-MVA-008-2005-007 | VISIT 4 | 230 | 1 | VACCINIA NAIVE SUBJECTS WITH ACTIVE ATOPIC DERMATITIS | N | 2 |
| POX-MVA-008-2005-007 | VISIT 5 | 100 | 1 | VACCINIA NAIVE SUBJECTS WITH ACTIVE ATOPIC DERMATITIS | N | 2 |
| POX-MVA-008-2005-009 | VISIT 1 | 1 | 1 | HEALTHY, VACCINIA NAIVE SUBJECTS WITHOUT ATOPIC DISEASE | N | 2 |
| POX-MVA-008-2005-009 | VISIT 2 | 1 | 1 | HEALTHY, VACCINIA NAIVE SUBJECTS WITHOUT ATOPIC DISEASE | N | 2 |
| POX-MVA-008-2005-009 | VISIT 3 | 50 | 1 | HEALTHY, VACCINIA NAIVE SUBJECTS WITHOUT ATOPIC DISEASE | N | 2 |
| POX-MVA-008-2005-009 | VISIT 4 | 221 | 1 | HEALTHY, VACCINIA NAIVE SUBJECTS WITHOUT ATOPIC DISEASE | N | 2 |
| POX-MVA-008-2005-009 | VISIT 5 | 272 | 1 | HEALTHY, VACCINIA NAIVE SUBJECTS WITHOUT ATOPIC DISEASE | N | 2 |
| POX-MVA-008-2005-011 | VISIT 1 | 1 | 1 | HEALTHY, VACCINIA NAIVE SUBJECTS WITHOUT ATOPIC DISEASE | N | 1 |
| POX-MVA-008-2005-011 | VISIT 2 | 1 | 1 | HEALTHY, VACCINIA NAIVE SUBJECTS WITHOUT ATOPIC DISEASE | N | 1 |
| POX-MVA-008-2005-013 | VISIT 1 | 1 | 1 | HEALTHY, VACCINIA NAIVE SUBJECTS WITHOUT ATOPIC DISEASE | N | 2 |
| POX-MVA-008-2005-013 | VISIT 2 | 1 | 1 | HEALTHY, VACCINIA NAIVE SUBJECTS WITHOUT ATOPIC DISEASE | N | 2 |
| POX-MVA-008-2005-013 | VISIT 3 | 1 | 1 | HEALTHY, VACCINIA NAIVE SUBJECTS WITHOUT ATOPIC DISEASE | N | 2 |
| POX-MVA-008-2005-013 | VISIT 4 | 324 | 1 | HEALTHY, VACCINIA NAIVE SUBJECTS WITHOUT ATOPIC DISEASE | N | 2 |
| POX-MVA-008-2005-013 | VISIT 5 | 100 | 1 | HEALTHY, VACCINIA NAIVE SUBJECTS WITHOUT ATOPIC DISEASE | N | 2 |
| POX-MVA-008-2005-014 | VISIT 1 | 1 | 1 | HEALTHY, VACCINIA NAIVE SUBJECTS WITHOUT ATOPIC DISEASE | N | 2 |
| POX-MVA-008-2005-014 | VISIT 2 | 100 | 1 | HEALTHY, VACCINIA NAIVE SUBJECTS WITHOUT ATOPIC DISEASE | N | 2 |
| POX-MVA-008-2005-014 | VISIT 3 | 200 | 1 | HEALTHY, VACCINIA NAIVE SUBJECTS WITHOUT ATOPIC DISEASE | N | 2 |
| POX-MVA-008-2005-014 | VISIT 4 | 438 | 15 | HEALTHY, VACCINIA NAIVE SUBJECTS WITHOUT ATOPIC DISEASE | N | 2 |
| POX-MVA-008-2005-014 | VISIT 5 | 334 | 15 | HEALTHY, VACCINIA NAIVE SUBJECTS WITHOUT ATOPIC DISEASE | N | 2 |
| POX-MVA-008-2005-014 | FOLLOW-UP VISIT | 100 | 15 | HEALTHY, VACCINIA NAIVE SUBJECTS WITHOUT ATOPIC DISEASE | N | 2 |
| POX-MVA-008-2005-015 | VISIT 1 | 1 | 1 | HEALTHY, VACCINIA NAIVE SUBJECTS WITHOUT ATOPIC DISEASE | N | 2 |
| POX-MVA-008-2005-015 | VISIT 2 | 50 | 1 | HEALTHY, VACCINIA NAIVE SUBJECTS WITHOUT ATOPIC DISEASE | N | 2 |
| POX-MVA-008-2005-015 | VISIT 3 | 50 | 1 | HEALTHY, VACCINIA NAIVE SUBJECTS WITHOUT ATOPIC DISEASE | N | 2 |
| POX-MVA-008-2005-015 | VISIT 4 | 100 | 1 | HEALTHY, VACCINIA NAIVE SUBJECTS WITHOUT ATOPIC DISEASE | N | 2 |
| POX-MVA-008-2005-015 | VISIT 5 | 100 | 15 | HEALTHY, VACCINIA NAIVE SUBJECTS WITHOUT ATOPIC DISEASE | N | 2 |
| POX-MVA-008-2005-015 | FOLLOW-UP VISIT | 50 | 1 | HEALTHY, VACCINIA NAIVE SUBJECTS WITHOUT ATOPIC DISEASE | N | 2 |
| POX-MVA-008-2005-021 | VISIT 1 | 1 | 1 | VACCINIA NAIVE SUBJECTS WITH HISTORY OF ATOPIC DERMATITIS | N | 2 |
| POX-MVA-008-2005-021 | VISIT 2 | 100 | 1 | VACCINIA NAIVE SUBJECTS WITH HISTORY OF ATOPIC DERMATITIS | N | 2 |
| POX-MVA-008-2005-021 | VISIT 3 | 100 | 1 | VACCINIA NAIVE SUBJECTS WITH HISTORY OF ATOPIC DERMATITIS | N | 2 |
| POX-MVA-008-2005-021 | VISIT 4 | 1122 | 593 | VACCINIA NAIVE SUBJECTS WITH HISTORY OF ATOPIC DERMATITIS | N | 2 |
| POX-MVA-008-2005-021 | VISIT 5 | 494 | 222 | VACCINIA NAIVE SUBJECTS WITH HISTORY OF ATOPIC DERMATITIS | N | 2 |
| POX-MVA-008-2009-003 | VISIT 1 | 50 | 1 | VACCINIA NAIVE SUBJECTS WITH ACTIVE ATOPIC DERMATITIS | N | 2 |
| POX-MVA-008-2009-003 | VISIT 2 | 1 | 1 | VACCINIA NAIVE SUBJECTS WITH ACTIVE ATOPIC DERMATITIS | N | 2 |
| POX-MVA-008-2009-003 | VISIT 3 | 100 | 1 | VACCINIA NAIVE SUBJECTS WITH ACTIVE ATOPIC DERMATITIS | N | 2 |
| POX-MVA-008-2009-004 | VISIT 1 | 1 | 1 | HEALTHY, VACCINIA NAIVE SUBJECTS WITHOUT ATOPIC DISEASE | N | 2 |
| POX-MVA-008-2009-004 | VISIT 2 | 1 | 1 | HEALTHY, VACCINIA NAIVE SUBJECTS WITHOUT ATOPIC DISEASE | N | 2 |
| POX-MVA-008-2009-004 | VISIT 3 | 1 | 1 | HEALTHY, VACCINIA NAIVE SUBJECTS WITHOUT ATOPIC DISEASE | N | 2 |
| POX-MVA-008-2009-004 | VISIT 4 | . | . | HEALTHY, VACCINIA NAIVE SUBJECTS WITHOUT ATOPIC DISEASE | N | 2 |
| POX-MVA-008-2009-004 | FOLLOW-UP VISIT | 1 | 1 | HEALTHY, VACCINIA NAIVE SUBJECTS WITHOUT ATOPIC DISEASE | N | 2 |
| POX-MVA-008-2009-005 | VISIT 1 | 1 | 1 | HEALTHY, VACCINIA NAIVE SUBJECTS WITHOUT ATOPIC DISEASE | N | 2 |
| POX-MVA-008-2009-005 | VISIT 2 | . | . | HEALTHY, VACCINIA NAIVE SUBJECTS WITHOUT ATOPIC DISEASE | N | 2 |
| POX-MVA-008-2009-005 | VISIT 3 | 100 | 1 | HEALTHY, VACCINIA NAIVE SUBJECTS WITHOUT ATOPIC DISEASE | N | 2 |
| POX-MVA-008-2009-005 | VISIT 4 | . | . | HEALTHY, VACCINIA NAIVE SUBJECTS WITHOUT ATOPIC DISEASE | N | 2 |
| POX-MVA-008-2009-005 | FOLLOW-UP VISIT | 50 | 1 | HEALTHY, VACCINIA NAIVE SUBJECTS WITHOUT ATOPIC DISEASE | N | 2 |
| POX-MVA-008-2009-006 | VISIT 1 | 1 | 1 | HEALTHY, VACCINIA NAIVE SUBJECTS WITHOUT ATOPIC DISEASE | N | 2 |
| POX-MVA-008-2009-006 | VISIT 2 | 1 | 1 | HEALTHY, VACCINIA NAIVE SUBJECTS WITHOUT ATOPIC DISEASE | N | 2 |
| POX-MVA-008-2009-006 | VISIT 3 | 50 | 15 | HEALTHY, VACCINIA NAIVE SUBJECTS WITHOUT ATOPIC DISEASE | N | 2 |
| POX-MVA-008-2009-006 | FOLLOW-UP VISIT | 50 | 1 | HEALTHY, VACCINIA NAIVE SUBJECTS WITHOUT ATOPIC DISEASE | N | 2 |
| POX-MVA-008-2009-007 | VISIT 1 | 1 | 1 | VACCINIA NAIVE SUBJECTS WITH ACTIVE ATOPIC DERMATITIS | N | 2 |
| POX-MVA-008-2009-007 | VISIT 2 | 1 | 1 | VACCINIA NAIVE SUBJECTS WITH ACTIVE ATOPIC DERMATITIS | N | 2 |
| POX-MVA-008-2009-007 | VISIT 3 | 100 | 1 | VACCINIA NAIVE SUBJECTS WITH ACTIVE ATOPIC DERMATITIS | N | 2 |
| POX-MVA-008-2009-007 | VISIT 4 | . | . | VACCINIA NAIVE SUBJECTS WITH ACTIVE ATOPIC DERMATITIS | N | 2 |
| POX-MVA-008-2009-007 | VISIT 5 | 200 | 15 | VACCINIA NAIVE SUBJECTS WITH ACTIVE ATOPIC DERMATITIS | N | 2 |
| POX-MVA-008-2009-007 | FOLLOW-UP VISIT | 50 | 1 | VACCINIA NAIVE SUBJECTS WITH ACTIVE ATOPIC DERMATITIS | N | 2 |
| POX-MVA-008-2009-010 | VISIT 1 | 1 | 1 | HEALTHY, VACCINIA NAIVE SUBJECTS WITHOUT ATOPIC DISEASE | N | 2 |
| POX-MVA-008-2009-010 | VISIT 2 | . | . | HEALTHY, VACCINIA NAIVE SUBJECTS WITHOUT ATOPIC DISEASE | N | 2 |
| POX-MVA-008-2009-010 | VISIT 3 | 50 | 1 | HEALTHY, VACCINIA NAIVE SUBJECTS WITHOUT ATOPIC DISEASE | N | 2 |
| POX-MVA-008-2009-010 | VISIT 4 | . | . | HEALTHY, VACCINIA NAIVE SUBJECTS WITHOUT ATOPIC DISEASE | N | 2 |
| POX-MVA-008-2009-010 | FOLLOW-UP VISIT | 100 | 1 | HEALTHY, VACCINIA NAIVE SUBJECTS WITHOUT ATOPIC DISEASE | N | 2 |
| POX-MVA-008-2009-012 | VISIT 1 | 1 | 1 | HEALTHY, VACCINIA NAIVE SUBJECTS WITHOUT ATOPIC DISEASE | N | 2 |
| POX-MVA-008-2009-012 | VISIT 2 | . | . | HEALTHY, VACCINIA NAIVE SUBJECTS WITHOUT ATOPIC DISEASE | N | 2 |
| POX-MVA-008-2009-012 | VISIT 4 | . | . | HEALTHY, VACCINIA NAIVE SUBJECTS WITHOUT ATOPIC DISEASE | N | 2 |
| POX-MVA-008-2009-012 | FOLLOW-UP VISIT | 50 | 1 | HEALTHY, VACCINIA NAIVE SUBJECTS WITHOUT ATOPIC DISEASE | N | 2 |
| POX-MVA-008-2009-014 | VISIT 1 | 1 | 1 | HEALTHY, VACCINIA NAIVE SUBJECTS WITHOUT ATOPIC DISEASE | N | 2 |
| POX-MVA-008-2009-014 | VISIT 2 | . | . | HEALTHY, VACCINIA NAIVE SUBJECTS WITHOUT ATOPIC DISEASE | N | 2 |
| POX-MVA-008-2009-014 | VISIT 3 | 100 | 15 | HEALTHY, VACCINIA NAIVE SUBJECTS WITHOUT ATOPIC DISEASE | N | 2 |
| POX-MVA-008-2009-014 | VISIT 4 | . | . | HEALTHY, VACCINIA NAIVE SUBJECTS WITHOUT ATOPIC DISEASE | N | 2 |
| POX-MVA-008-2009-020 | VISIT 2 | 1 | 1 | VACCINIA NAIVE SUBJECTS WITH HISTORY OF ATOPIC DERMATITIS | N | 1 |
| POX-MVA-008-2009-026 | VISIT 2 | 1 | 1 | VACCINIA NAIVE SUBJECTS WITH ACTIVE ATOPIC DERMATITIS | N | 2 |
| POX-MVA-008-2009-026 | VISIT 3 | 100 | 1 | VACCINIA NAIVE SUBJECTS WITH ACTIVE ATOPIC DERMATITIS | N | 2 |
| POX-MVA-008-2009-026 | VISIT 4 | 318 | 15 | VACCINIA NAIVE SUBJECTS WITH ACTIVE ATOPIC DERMATITIS | N | 2 |
| POX-MVA-008-2009-026 | VISIT 5 | 261 | 15 | VACCINIA NAIVE SUBJECTS WITH ACTIVE ATOPIC DERMATITIS | N | 2 |
| POX-MVA-008-2011-009 | VISIT 1 | 1 | 1 | HEALTHY, VACCINIA NAIVE SUBJECTS WITHOUT ATOPIC DISEASE | N | 1 |
| POX-MVA-008-2011-009 | VISIT 2 | 1 | 1 | HEALTHY, VACCINIA NAIVE SUBJECTS WITHOUT ATOPIC DISEASE | N | 1 |
| POX-MVA-008-2011-009 | VISIT 3 | 1 | 1 | HEALTHY, VACCINIA NAIVE SUBJECTS WITHOUT ATOPIC DISEASE | N | 1 |
| POX-MVA-008-2011-025 | VISIT 1 | 1 | 1 | HEALTHY, VACCINIA NAIVE SUBJECTS WITHOUT ATOPIC DISEASE | N | 2 |
| POX-MVA-008-2011-025 | VISIT 2 | 1 | 1 | HEALTHY, VACCINIA NAIVE SUBJECTS WITHOUT ATOPIC DISEASE | N | 2 |
| POX-MVA-008-2011-025 | VISIT 3 | 1 | 1 | HEALTHY, VACCINIA NAIVE SUBJECTS WITHOUT ATOPIC DISEASE | N | 2 |
| POX-MVA-008-2011-025 | VISIT 4 | 50 | 15 | HEALTHY, VACCINIA NAIVE SUBJECTS WITHOUT ATOPIC DISEASE | N | 2 |
| POX-MVA-008-2011-025 | VISIT 5 | 1 | 1 | HEALTHY, VACCINIA NAIVE SUBJECTS WITHOUT ATOPIC DISEASE | N | 2 |
| POX-MVA-008-2011-025 | FOLLOW-UP VISIT | 1 | 1 | HEALTHY, VACCINIA NAIVE SUBJECTS WITHOUT ATOPIC DISEASE | N | 2 |
| POX-MVA-008-2011-032 | VISIT 1 | 1 | 1 | VACCINIA NAIVE SUBJECTS WITH HISTORY OF ATOPIC DERMATITIS | N | 2 |
| POX-MVA-008-2011-032 | VISIT 2 | 1 | 1 | VACCINIA NAIVE SUBJECTS WITH HISTORY OF ATOPIC DERMATITIS | N | 2 |
| POX-MVA-008-2011-032 | VISIT 3 | 50 | 1 | VACCINIA NAIVE SUBJECTS WITH HISTORY OF ATOPIC DERMATITIS | N | 2 |
| POX-MVA-008-2011-032 | VISIT 4 | 100 | 1 | VACCINIA NAIVE SUBJECTS WITH HISTORY OF ATOPIC DERMATITIS | N | 2 |
| POX-MVA-008-2011-032 | VISIT 5 | 298 | 1 | VACCINIA NAIVE SUBJECTS WITH HISTORY OF ATOPIC DERMATITIS | N | 2 |
| POX-MVA-008-2011-032 | FOLLOW-UP VISIT | 50 | 1 | VACCINIA NAIVE SUBJECTS WITH HISTORY OF ATOPIC DERMATITIS | N | 2 |
| POX-MVA-008-2011-039 | VISIT 1 | 1 | 1 | HEALTHY, VACCINIA NAIVE SUBJECTS WITHOUT ATOPIC DISEASE | N | 2 |
| POX-MVA-008-2011-039 | VISIT 2 | 1 | 1 | HEALTHY, VACCINIA NAIVE SUBJECTS WITHOUT ATOPIC DISEASE | N | 2 |
| POX-MVA-008-2011-039 | VISIT 3 | 50 | 1 | HEALTHY, VACCINIA NAIVE SUBJECTS WITHOUT ATOPIC DISEASE | N | 2 |
| POX-MVA-008-2011-039 | UNSCHEDULED VISIT 4A | 100 | 1 | HEALTHY, VACCINIA NAIVE SUBJECTS WITHOUT ATOPIC DISEASE | N | 2 |
| POX-MVA-008-2011-039 | VISIT 5 | 100 | 1 | HEALTHY, VACCINIA NAIVE SUBJECTS WITHOUT ATOPIC DISEASE | N | 2 |
| POX-MVA-008-2011-039 | FOLLOW-UP VISIT | 50 | 1 | HEALTHY, VACCINIA NAIVE SUBJECTS WITHOUT ATOPIC DISEASE | N | 2 |
| POX-MVA-008-2011-059 | VISIT 1 | 1 | 1 | HEALTHY, VACCINIA NAIVE SUBJECTS WITHOUT ATOPIC DISEASE | N | 1 |
| POX-MVA-008-2011-059 | VISIT 2 | 1 | 1 | HEALTHY, VACCINIA NAIVE SUBJECTS WITHOUT ATOPIC DISEASE | N | 1 |
| POX-MVA-008-2011-059 | VISIT 5 | 225 | 1 | HEALTHY, VACCINIA NAIVE SUBJECTS WITHOUT ATOPIC DISEASE | N | 1 |
| POX-MVA-008-2011-069 | VISIT 1 | 1 | 1 | HEALTHY, VACCINIA NAIVE SUBJECTS WITHOUT ATOPIC DISEASE | N | 2 |
| POX-MVA-008-2011-069 | VISIT 2 | 100 | 1 | HEALTHY, VACCINIA NAIVE SUBJECTS WITHOUT ATOPIC DISEASE | N | 2 |
| POX-MVA-008-2011-069 | VISIT 3 | 100 | 1 | HEALTHY, VACCINIA NAIVE SUBJECTS WITHOUT ATOPIC DISEASE | N | 2 |
| POX-MVA-008-2011-069 | VISIT 4 | 478 | 130 | HEALTHY, VACCINIA NAIVE SUBJECTS WITHOUT ATOPIC DISEASE | N | 2 |
| POX-MVA-008-2011-069 | VISIT 5 | 424 | 148 | HEALTHY, VACCINIA NAIVE SUBJECTS WITHOUT ATOPIC DISEASE | N | 2 |
| POX-MVA-008-2011-077 | VISIT 1 | 1 | 1 | HEALTHY, VACCINIA NAIVE SUBJECTS WITHOUT ATOPIC DISEASE | N | 2 |
| POX-MVA-008-2011-077 | VISIT 2 | 1 | 1 | HEALTHY, VACCINIA NAIVE SUBJECTS WITHOUT ATOPIC DISEASE | N | 2 |
| POX-MVA-008-2011-077 | VISIT 3 | 1 | 1 | HEALTHY, VACCINIA NAIVE SUBJECTS WITHOUT ATOPIC DISEASE | N | 2 |
| POX-MVA-008-2011-077 | VISIT 4 | 505 | 15 | HEALTHY, VACCINIA NAIVE SUBJECTS WITHOUT ATOPIC DISEASE | N | 2 |
| POX-MVA-008-2011-077 | VISIT 5 | 278 | 15 | HEALTHY, VACCINIA NAIVE SUBJECTS WITHOUT ATOPIC DISEASE | N | 2 |
| POX-MVA-008-2011-100 | VISIT 1 | 1 | 1 | VACCINIA NAIVE SUBJECTS WITH ACTIVE ATOPIC DERMATITIS | N | 1 |
| POX-MVA-008-2011-100 | VISIT 2 | 1 | 1 | VACCINIA NAIVE SUBJECTS WITH ACTIVE ATOPIC DERMATITIS | N | 1 |
| POX-MVA-008-2011-100 | VISIT 5 | 50 | 1 | VACCINIA NAIVE SUBJECTS WITH ACTIVE ATOPIC DERMATITIS | N | 1 |
| POX-MVA-008-2012-001 | VISIT 1 | 1 | 1 | HEALTHY, VACCINIA NAIVE SUBJECTS WITHOUT ATOPIC DISEASE | N | 1 |
| POX-MVA-008-2012-001 | VISIT 2 | 1 | 1 | HEALTHY, VACCINIA NAIVE SUBJECTS WITHOUT ATOPIC DISEASE | N | 1 |
| POX-MVA-008-2012-001 | VISIT 5 | 100 | 1 | HEALTHY, VACCINIA NAIVE SUBJECTS WITHOUT ATOPIC DISEASE | N | 1 |
| POX-MVA-008-2012-015 | VISIT 1 | 1 | 1 | HEALTHY, VACCINIA NAIVE SUBJECTS WITHOUT ATOPIC DISEASE | N | 2 |
| POX-MVA-008-2012-015 | VISIT 2 | 1 | 1 | HEALTHY, VACCINIA NAIVE SUBJECTS WITHOUT ATOPIC DISEASE | N | 2 |
| POX-MVA-008-2012-015 | VISIT 3 | 333 | 1 | HEALTHY, VACCINIA NAIVE SUBJECTS WITHOUT ATOPIC DISEASE | N | 2 |
| POX-MVA-008-2013-003 | VISIT 1 | 50 | 15 | VACCINIA NAIVE SUBJECTS WITH ACTIVE ATOPIC DERMATITIS | N | 2 |
| POX-MVA-008-2013-003 | VISIT 2 | 199 | 109 | VACCINIA NAIVE SUBJECTS WITH ACTIVE ATOPIC DERMATITIS | N | 2 |
| POX-MVA-008-2013-003 | VISIT 3 | 454 | 810 | VACCINIA NAIVE SUBJECTS WITH ACTIVE ATOPIC DERMATITIS | N | 2 |
| POX-MVA-008-2013-003 | VISIT 4 | 1383 | 3529 | VACCINIA NAIVE SUBJECTS WITH ACTIVE ATOPIC DERMATITIS | N | 2 |
| POX-MVA-008-2013-003 | VISIT 5 | 985 | 2797 | VACCINIA NAIVE SUBJECTS WITH ACTIVE ATOPIC DERMATITIS | N | 2 |
| POX-MVA-008-2013-005 | VISIT 1 | 50 | 1 | VACCINIA NAIVE SUBJECTS WITH ACTIVE ATOPIC DERMATITIS | N | 2 |
| POX-MVA-008-2013-005 | VISIT 2 | 50 | 1 | VACCINIA NAIVE SUBJECTS WITH ACTIVE ATOPIC DERMATITIS | N | 2 |
| POX-MVA-008-2013-005 | VISIT 3 | 417 | 1 | VACCINIA NAIVE SUBJECTS WITH ACTIVE ATOPIC DERMATITIS | N | 2 |
| POX-MVA-008-2013-005 | VISIT 4 | 1830 | 299 | VACCINIA NAIVE SUBJECTS WITH ACTIVE ATOPIC DERMATITIS | N | 2 |
| POX-MVA-008-2013-005 | VISIT 5 | 1122 | 387 | VACCINIA NAIVE SUBJECTS WITH ACTIVE ATOPIC DERMATITIS | N | 2 |
| POX-MVA-008-2013-007 | VISIT 1 | 1 | 1 | VACCINIA NAIVE SUBJECTS WITH HISTORY OF ATOPIC DERMATITIS | N | 2 |
| POX-MVA-008-2013-007 | VISIT 2 | 1 | 1 | VACCINIA NAIVE SUBJECTS WITH HISTORY OF ATOPIC DERMATITIS | N | 2 |
| POX-MVA-008-2013-007 | VISIT 3 | 1 | 1 | VACCINIA NAIVE SUBJECTS WITH HISTORY OF ATOPIC DERMATITIS | N | 2 |
| POX-MVA-008-2013-007 | VISIT 4 | 212 | 15 | VACCINIA NAIVE SUBJECTS WITH HISTORY OF ATOPIC DERMATITIS | N | 2 |
| POX-MVA-008-2013-007 | VISIT 5 | 284 | 15 | VACCINIA NAIVE SUBJECTS WITH HISTORY OF ATOPIC DERMATITIS | N | 2 |
| POX-MVA-008-2015-006 | VISIT 1 | 1 | 1 | VACCINIA NAIVE SUBJECTS WITH HISTORY OF ATOPIC DERMATITIS | N | 2 |
| POX-MVA-008-2015-006 | VISIT 2 | 1 | 1 | VACCINIA NAIVE SUBJECTS WITH HISTORY OF ATOPIC DERMATITIS | N | 2 |
| POX-MVA-008-2015-006 | VISIT 3 | 50 | 1 | VACCINIA NAIVE SUBJECTS WITH HISTORY OF ATOPIC DERMATITIS | N | 2 |
| POX-MVA-008-2015-006 | VISIT 4 | 100 | 1 | VACCINIA NAIVE SUBJECTS WITH HISTORY OF ATOPIC DERMATITIS | N | 2 |
| POX-MVA-008-2015-006 | VISIT 5 | 50 | 1 | VACCINIA NAIVE SUBJECTS WITH HISTORY OF ATOPIC DERMATITIS | N | 2 |
| POX-MVA-008-2015-009 | VISIT 1 | 1 | 1 | VACCINIA NAIVE SUBJECTS WITH ACTIVE ATOPIC DERMATITIS | N | 2 |
| POX-MVA-008-2015-009 | VISIT 2 | 1 | 1 | VACCINIA NAIVE SUBJECTS WITH ACTIVE ATOPIC DERMATITIS | N | 2 |
| POX-MVA-008-2015-009 | VISIT 3 | 50 | 1 | VACCINIA NAIVE SUBJECTS WITH ACTIVE ATOPIC DERMATITIS | N | 2 |
| POX-MVA-008-2015-009 | VISIT 4 | 200 | 15 | VACCINIA NAIVE SUBJECTS WITH ACTIVE ATOPIC DERMATITIS | N | 2 |
| POX-MVA-008-2015-009 | VISIT 5 | 100 | 15 | VACCINIA NAIVE SUBJECTS WITH ACTIVE ATOPIC DERMATITIS | N | 2 |
| POX-MVA-008-2015-010 | VISIT 1 | 1 | 1 | VACCINIA NAIVE SUBJECTS WITH ACTIVE ATOPIC DERMATITIS | N | 2 |
| POX-MVA-008-2015-010 | VISIT 2 | 100 | 1 | VACCINIA NAIVE SUBJECTS WITH ACTIVE ATOPIC DERMATITIS | N | 2 |
| POX-MVA-008-2015-010 | VISIT 3 | 100 | 1 | VACCINIA NAIVE SUBJECTS WITH ACTIVE ATOPIC DERMATITIS | N | 2 |
| POX-MVA-008-2015-010 | VISIT 4 | 222 | 15 | VACCINIA NAIVE SUBJECTS WITH ACTIVE ATOPIC DERMATITIS | N | 2 |
| POX-MVA-008-2015-010 | VISIT 5 | 217 | 15 | VACCINIA NAIVE SUBJECTS WITH ACTIVE ATOPIC DERMATITIS | N | 2 |
| POX-MVA-008-2015-012 | VISIT 1 | 1 | 1 | VACCINIA NAIVE SUBJECTS WITH ACTIVE ATOPIC DERMATITIS | N | 2 |
| POX-MVA-008-2015-012 | VISIT 2 | 1 | 1 | VACCINIA NAIVE SUBJECTS WITH ACTIVE ATOPIC DERMATITIS | N | 2 |
| POX-MVA-008-2015-012 | VISIT 3 | 100 | 1 | VACCINIA NAIVE SUBJECTS WITH ACTIVE ATOPIC DERMATITIS | N | 2 |
| POX-MVA-008-2015-012 | VISIT 4 | 740 | 223 | VACCINIA NAIVE SUBJECTS WITH ACTIVE ATOPIC DERMATITIS | N | 2 |
| POX-MVA-008-2015-012 | VISIT 5 | 256 | 15 | VACCINIA NAIVE SUBJECTS WITH ACTIVE ATOPIC DERMATITIS | N | 2 |
| POX-MVA-008-2017-002 | VISIT 1 | 1 | 1 | HEALTHY, VACCINIA NAIVE SUBJECTS WITHOUT ATOPIC DISEASE | N | 2 |
| POX-MVA-008-2017-002 | VISIT 2 | 1 | 1 | HEALTHY, VACCINIA NAIVE SUBJECTS WITHOUT ATOPIC DISEASE | N | 2 |
| POX-MVA-008-2017-002 | VISIT 3 | 100 | 1 | HEALTHY, VACCINIA NAIVE SUBJECTS WITHOUT ATOPIC DISEASE | N | 2 |
| POX-MVA-008-2017-002 | VISIT 4 | 454 | 15 | HEALTHY, VACCINIA NAIVE SUBJECTS WITHOUT ATOPIC DISEASE | N | 2 |
| POX-MVA-008-2017-002 | VISIT 5 | 283 | 15 | HEALTHY, VACCINIA NAIVE SUBJECTS WITHOUT ATOPIC DISEASE | N | 2 |
| POX-MVA-008-2017-007 | VISIT 1 | 1 | 1 | VACCINIA NAIVE SUBJECTS WITH ACTIVE ATOPIC DERMATITIS | N | 2 |
| POX-MVA-008-2017-007 | VISIT 2 | 1 | 1 | VACCINIA NAIVE SUBJECTS WITH ACTIVE ATOPIC DERMATITIS | N | 2 |
| POX-MVA-008-2017-007 | VISIT 3 | 50 | 1 | VACCINIA NAIVE SUBJECTS WITH ACTIVE ATOPIC DERMATITIS | N | 2 |
| POX-MVA-008-2017-007 | VISIT 4 | 100 | 15 | VACCINIA NAIVE SUBJECTS WITH ACTIVE ATOPIC DERMATITIS | N | 2 |
| POX-MVA-008-2017-007 | VISIT 5 | 100 | 15 | VACCINIA NAIVE SUBJECTS WITH ACTIVE ATOPIC DERMATITIS | N | 2 |
| POX-MVA-008-2018-008 | VISIT 1 | 1 | 1 | HEALTHY, VACCINIA NAIVE SUBJECTS WITHOUT ATOPIC DISEASE | N | 2 |
| POX-MVA-008-2018-008 | VISIT 2 | 1 | 1 | HEALTHY, VACCINIA NAIVE SUBJECTS WITHOUT ATOPIC DISEASE | N | 2 |
| POX-MVA-008-2018-008 | VISIT 3 | 100 | 15 | HEALTHY, VACCINIA NAIVE SUBJECTS WITHOUT ATOPIC DISEASE | N | 2 |
| POX-MVA-008-2018-008 | VISIT 4 | 1646 | 286 | HEALTHY, VACCINIA NAIVE SUBJECTS WITHOUT ATOPIC DISEASE | N | 2 |
| POX-MVA-008-2018-008 | VISIT 5 | 1260 | 183 | HEALTHY, VACCINIA NAIVE SUBJECTS WITHOUT ATOPIC DISEASE | N | 2 |
| POX-MVA-008-2018-009 | VISIT 1 | 1 | 1 | HEALTHY, VACCINIA NAIVE SUBJECTS WITHOUT ATOPIC DISEASE | N | 2 |
| POX-MVA-008-2018-009 | VISIT 2 | 50 | 1 | HEALTHY, VACCINIA NAIVE SUBJECTS WITHOUT ATOPIC DISEASE | N | 2 |
| POX-MVA-008-2018-009 | VISIT 3 | 100 | 1 | HEALTHY, VACCINIA NAIVE SUBJECTS WITHOUT ATOPIC DISEASE | N | 2 |
| POX-MVA-008-2018-009 | VISIT 4 | 1493 | 284 | HEALTHY, VACCINIA NAIVE SUBJECTS WITHOUT ATOPIC DISEASE | N | 2 |
| POX-MVA-008-2018-009 | VISIT 5 | 436 | 15 | HEALTHY, VACCINIA NAIVE SUBJECTS WITHOUT ATOPIC DISEASE | N | 2 |
| POX-MVA-008-2018-013 | VISIT 1 | 200 | 15 | VACCINIA NAIVE SUBJECTS WITH ACTIVE ATOPIC DERMATITIS | N | 2 |
| POX-MVA-008-2018-013 | VISIT 2 | 200 | 15 | VACCINIA NAIVE SUBJECTS WITH ACTIVE ATOPIC DERMATITIS | N | 2 |
| POX-MVA-008-2018-013 | VISIT 3 | 297 | 1 | VACCINIA NAIVE SUBJECTS WITH ACTIVE ATOPIC DERMATITIS | N | 2 |
| POX-MVA-008-2018-013 | VISIT 4 | 279 | 15 | VACCINIA NAIVE SUBJECTS WITH ACTIVE ATOPIC DERMATITIS | N | 2 |
| POX-MVA-008-2018-013 | VISIT 5 | 100 | 1 | VACCINIA NAIVE SUBJECTS WITH ACTIVE ATOPIC DERMATITIS | N | 2 |
| POX-MVA-008-2018-013 | FOLLOW-UP VISIT | 200 | 1 | VACCINIA NAIVE SUBJECTS WITH ACTIVE ATOPIC DERMATITIS | N | 2 |
| POX-MVA-008-2018-014 | VISIT 1 | 200 | 15 | HEALTHY, VACCINIA NAIVE SUBJECTS WITHOUT ATOPIC DISEASE | N | 2 |
| POX-MVA-008-2018-014 | VISIT 2 | 233 | 15 | HEALTHY, VACCINIA NAIVE SUBJECTS WITHOUT ATOPIC DISEASE | N | 2 |
| POX-MVA-008-2018-014 | VISIT 3 | 326 | 1 | HEALTHY, VACCINIA NAIVE SUBJECTS WITHOUT ATOPIC DISEASE | N | 2 |
| POX-MVA-008-2018-014 | VISIT 4 | 1179 | 89 | HEALTHY, VACCINIA NAIVE SUBJECTS WITHOUT ATOPIC DISEASE | N | 2 |
| POX-MVA-008-2018-014 | VISIT 5 | 100 | 1 | HEALTHY, VACCINIA NAIVE SUBJECTS WITHOUT ATOPIC DISEASE | N | 2 |
| POX-MVA-008-2018-016 | VISIT 1 | 1 | 1 | HEALTHY, VACCINIA NAIVE SUBJECTS WITHOUT ATOPIC DISEASE | N | 2 |
| POX-MVA-008-2018-016 | VISIT 2 | 100 | 1 | HEALTHY, VACCINIA NAIVE SUBJECTS WITHOUT ATOPIC DISEASE | N | 2 |
| POX-MVA-008-2018-016 | VISIT 3 | 228 | 1 | HEALTHY, VACCINIA NAIVE SUBJECTS WITHOUT ATOPIC DISEASE | N | 2 |
| POX-MVA-008-2018-016 | VISIT 4 | 1207 | 15 | HEALTHY, VACCINIA NAIVE SUBJECTS WITHOUT ATOPIC DISEASE | N | 2 |
| POX-MVA-008-2018-016 | VISIT 5 | 594 | 15 | HEALTHY, VACCINIA NAIVE SUBJECTS WITHOUT ATOPIC DISEASE | N | 2 |
| POX-MVA-008-2018-017 | VISIT 1 | 1 | 1 | HEALTHY, VACCINIA NAIVE SUBJECTS WITHOUT ATOPIC DISEASE | N | 2 |
| POX-MVA-008-2018-017 | VISIT 2 | 50 | 1 | HEALTHY, VACCINIA NAIVE SUBJECTS WITHOUT ATOPIC DISEASE | N | 2 |
| POX-MVA-008-2018-017 | VISIT 3 | 50 | 1 | HEALTHY, VACCINIA NAIVE SUBJECTS WITHOUT ATOPIC DISEASE | N | 2 |
| POX-MVA-008-2018-017 | VISIT 4 | 3500 | 1172 | HEALTHY, VACCINIA NAIVE SUBJECTS WITHOUT ATOPIC DISEASE | N | 2 |
| POX-MVA-008-2018-017 | VISIT 5 | 2733 | 15 | HEALTHY, VACCINIA NAIVE SUBJECTS WITHOUT ATOPIC DISEASE | N | 2 |
| POX-MVA-008-2018-025 | VISIT 1 | 100 | 1 | VACCINIA NAIVE SUBJECTS WITH HISTORY OF ATOPIC DERMATITIS | N | 2 |
| POX-MVA-008-2018-025 | VISIT 2 | 100 | 1 | VACCINIA NAIVE SUBJECTS WITH HISTORY OF ATOPIC DERMATITIS | N | 2 |
| POX-MVA-008-2018-025 | VISIT 3 | 50 | 1 | VACCINIA NAIVE SUBJECTS WITH HISTORY OF ATOPIC DERMATITIS | N | 2 |
| POX-MVA-008-2018-025 | VISIT 4 | 100 | 1 | VACCINIA NAIVE SUBJECTS WITH HISTORY OF ATOPIC DERMATITIS | N | 2 |
| POX-MVA-008-2018-025 | VISIT 5 | 100 | 1 | VACCINIA NAIVE SUBJECTS WITH HISTORY OF ATOPIC DERMATITIS | N | 2 |
| POX-MVA-008-2018-028 | VISIT 1 | 100 | 86 | HEALTHY, VACCINIA NAIVE SUBJECTS WITHOUT ATOPIC DISEASE | N | 2 |
| POX-MVA-008-2018-028 | VISIT 2 | 100 | 15 | HEALTHY, VACCINIA NAIVE SUBJECTS WITHOUT ATOPIC DISEASE | N | 2 |
| POX-MVA-008-2018-028 | VISIT 3 | 100 | 1 | HEALTHY, VACCINIA NAIVE SUBJECTS WITHOUT ATOPIC DISEASE | N | 2 |
| POX-MVA-008-2018-028 | VISIT 4 | 525 | 260 | HEALTHY, VACCINIA NAIVE SUBJECTS WITHOUT ATOPIC DISEASE | N | 2 |
| POX-MVA-008-2018-028 | VISIT 5 | 1 | 1 | HEALTHY, VACCINIA NAIVE SUBJECTS WITHOUT ATOPIC DISEASE | N | 2 |
| POX-MVA-008-2018-029 | VISIT 1 | 100 | 15 | HEALTHY, VACCINIA NAIVE SUBJECTS WITHOUT ATOPIC DISEASE | N | 2 |
| POX-MVA-008-2018-029 | VISIT 2 | 100 | 1 | HEALTHY, VACCINIA NAIVE SUBJECTS WITHOUT ATOPIC DISEASE | N | 2 |
| POX-MVA-008-2018-029 | VISIT 3 | 1046 | 269 | HEALTHY, VACCINIA NAIVE SUBJECTS WITHOUT ATOPIC DISEASE | N | 2 |
| POX-MVA-008-2018-029 | VISIT 4 | 806 | 265 | HEALTHY, VACCINIA NAIVE SUBJECTS WITHOUT ATOPIC DISEASE | N | 2 |
| POX-MVA-008-2018-029 | VISIT 5 | 407 | 206 | HEALTHY, VACCINIA NAIVE SUBJECTS WITHOUT ATOPIC DISEASE | N | 2 |
| POX-MVA-008-2018-032 | VISIT 1 | 100 | 15 | HEALTHY, VACCINIA NAIVE SUBJECTS WITHOUT ATOPIC DISEASE | N | 2 |
| POX-MVA-008-2018-032 | VISIT 2 | 2372 | 478 | HEALTHY, VACCINIA NAIVE SUBJECTS WITHOUT ATOPIC DISEASE | N | 2 |
| POX-MVA-008-2018-032 | VISIT 3 | 1055 | 445 | HEALTHY, VACCINIA NAIVE SUBJECTS WITHOUT ATOPIC DISEASE | N | 2 |
| POX-MVA-008-2018-032 | VISIT 4 | 1086 | 207 | HEALTHY, VACCINIA NAIVE SUBJECTS WITHOUT ATOPIC DISEASE | N | 2 |
| POX-MVA-008-2018-032 | VISIT 5 | 100 | 1 | HEALTHY, VACCINIA NAIVE SUBJECTS WITHOUT ATOPIC DISEASE | N | 2 |
| POX-MVA-008-2018-036 | VISIT 1 | 50 | 15 | VACCINIA NAIVE SUBJECTS WITH HISTORY OF ATOPIC DERMATITIS | N | 2 |
| POX-MVA-008-2018-036 | VISIT 3 | 1968 | 252 | VACCINIA NAIVE SUBJECTS WITH HISTORY OF ATOPIC DERMATITIS | N | 2 |
| POX-MVA-008-2018-036 | VISIT 4 | 1024 | 77 | VACCINIA NAIVE SUBJECTS WITH HISTORY OF ATOPIC DERMATITIS | N | 2 |
| POX-MVA-008-2018-036 | VISIT 5 | 1 | 1 | VACCINIA NAIVE SUBJECTS WITH HISTORY OF ATOPIC DERMATITIS | N | 2 |
| POX-MVA-008-2018-038 | VISIT 1 | 50 | 1 | VACCINIA NAIVE SUBJECTS WITH HISTORY OF ATOPIC DERMATITIS | N | 1 |
| POX-MVA-008-2018-038 | VISIT 2 | 50 | 15 | VACCINIA NAIVE SUBJECTS WITH HISTORY OF ATOPIC DERMATITIS | N | 1 |
| POX-MVA-008-2018-038 | VISIT 5 | 936 | 391 | VACCINIA NAIVE SUBJECTS WITH HISTORY OF ATOPIC DERMATITIS | N | 1 |
| POX-MVA-008-2018-050 | VISIT 1 | 1 | 1 | VACCINIA NAIVE SUBJECTS WITH HISTORY OF ATOPIC DERMATITIS | N | 1 |
| POX-MVA-008-2018-050 | VISIT 2 | 1 | 1 | VACCINIA NAIVE SUBJECTS WITH HISTORY OF ATOPIC DERMATITIS | N | 1 |
| POX-MVA-008-2018-054 | VISIT 1 | 1 | 1 | VACCINIA NAIVE SUBJECTS WITH HISTORY OF ATOPIC DERMATITIS | N | 1 |
| POX-MVA-008-2018-055 | VISIT 1 | 1 | 1 | VACCINIA NAIVE SUBJECTS WITH HISTORY OF ATOPIC DERMATITIS | N | 2 |
| POX-MVA-008-2018-055 | VISIT 2 | 1 | 1 | VACCINIA NAIVE SUBJECTS WITH HISTORY OF ATOPIC DERMATITIS | N | 2 |
| POX-MVA-008-2018-055 | VISIT 3 | 1 | 1 | VACCINIA NAIVE SUBJECTS WITH HISTORY OF ATOPIC DERMATITIS | N | 2 |
| POX-MVA-008-2018-055 | VISIT 5 | 50 | 1 | VACCINIA NAIVE SUBJECTS WITH HISTORY OF ATOPIC DERMATITIS | N | 2 |
| POX-MVA-008-2018-062 | VISIT 1 | 1 | 15 | VACCINIA NAIVE SUBJECTS WITH HISTORY OF ATOPIC DERMATITIS | N | 1 |
| POX-MVA-008-2018-062 | VISIT 5 | 50 | 1 | VACCINIA NAIVE SUBJECTS WITH HISTORY OF ATOPIC DERMATITIS | N | 1 |
| POX-MVA-008-2018-067 | VISIT 1 | 1 | 1 | VACCINIA NAIVE SUBJECTS WITH ACTIVE ATOPIC DERMATITIS | N | 1 |
| POX-MVA-008-2018-067 | VISIT 2 | 1 | 1 | VACCINIA NAIVE SUBJECTS WITH ACTIVE ATOPIC DERMATITIS | N | 1 |
| POX-MVA-008-2018-067 | VISIT 5 | 682 | 184 | VACCINIA NAIVE SUBJECTS WITH ACTIVE ATOPIC DERMATITIS | N | 1 |
| POX-MVA-008-2018-071 | VISIT 1 | 1 | 1 | VACCINIA NAIVE SUBJECTS WITH HISTORY OF ATOPIC DERMATITIS | N | 1 |
| POX-MVA-008-2018-071 | VISIT 2 | 1 | 1 | VACCINIA NAIVE SUBJECTS WITH HISTORY OF ATOPIC DERMATITIS | N | 1 |
| POX-MVA-008-2018-071 | VISIT 5 | 50 | 15 | VACCINIA NAIVE SUBJECTS WITH HISTORY OF ATOPIC DERMATITIS | N | 1 |
| POX-MVA-008-2018-076 | VISIT 1 | 1 | 1 | VACCINIA NAIVE SUBJECTS WITH HISTORY OF ATOPIC DERMATITIS | N | 1 |
| POX-MVA-008-2018-076 | VISIT 2 | 1 | 1 | VACCINIA NAIVE SUBJECTS WITH HISTORY OF ATOPIC DERMATITIS | N | 1 |
| POX-MVA-008-2018-076 | VISIT 5 | 602 | 127 | VACCINIA NAIVE SUBJECTS WITH HISTORY OF ATOPIC DERMATITIS | N | 1 |
| POX-MVA-008-2018-084 | VISIT 1 | 1 | 1 | VACCINIA NAIVE SUBJECTS WITH ACTIVE ATOPIC DERMATITIS | N | 2 |
| POX-MVA-008-2018-084 | VISIT 2 | 1 | 1 | VACCINIA NAIVE SUBJECTS WITH ACTIVE ATOPIC DERMATITIS | N | 2 |
| POX-MVA-008-2018-084 | VISIT 3 | 1 | 1 | VACCINIA NAIVE SUBJECTS WITH ACTIVE ATOPIC DERMATITIS | N | 2 |
| POX-MVA-008-2018-084 | VISIT 4 | 50 | 1 | VACCINIA NAIVE SUBJECTS WITH ACTIVE ATOPIC DERMATITIS | N | 2 |
| POX-MVA-008-2018-084 | VISIT 5 | 1 | 1 | VACCINIA NAIVE SUBJECTS WITH ACTIVE ATOPIC DERMATITIS | N | 2 |
| POX-MVA-008-2018-085 | VISIT 1 | 1 | 1 | VACCINIA NAIVE SUBJECTS WITH HISTORY OF ATOPIC DERMATITIS | N | 1 |
| POX-MVA-008-2018-085 | VISIT 5 | 100 | 15 | VACCINIA NAIVE SUBJECTS WITH HISTORY OF ATOPIC DERMATITIS | N | 1 |
| POX-MVA-008-2018-102 | VISIT 1 | 1 | 1 | VACCINIA NAIVE SUBJECTS WITH ACTIVE ATOPIC DERMATITIS | N | 2 |
| POX-MVA-008-2018-102 | VISIT 2 | 1 | 1 | VACCINIA NAIVE SUBJECTS WITH ACTIVE ATOPIC DERMATITIS | N | 2 |
| POX-MVA-008-2018-102 | VISIT 3 | 50 | 1 | VACCINIA NAIVE SUBJECTS WITH ACTIVE ATOPIC DERMATITIS | N | 2 |
| POX-MVA-008-2018-102 | VISIT 4 | 328 | 15 | VACCINIA NAIVE SUBJECTS WITH ACTIVE ATOPIC DERMATITIS | N | 2 |
| POX-MVA-008-2018-102 | VISIT 5 | 211 | 15 | VACCINIA NAIVE SUBJECTS WITH ACTIVE ATOPIC DERMATITIS | N | 2 |
| POX-MVA-008-2018-103 | VISIT 1 | 1 | 1 | VACCINIA NAIVE SUBJECTS WITH ACTIVE ATOPIC DERMATITIS | N | 2 |
| POX-MVA-008-2018-103 | VISIT 2 | 1 | 1 | VACCINIA NAIVE SUBJECTS WITH ACTIVE ATOPIC DERMATITIS | N | 2 |
| POX-MVA-008-2018-103 | VISIT 3 | 50 | 1 | VACCINIA NAIVE SUBJECTS WITH ACTIVE ATOPIC DERMATITIS | N | 2 |
| POX-MVA-008-2018-103 | VISIT 5 | 1 | 1 | VACCINIA NAIVE SUBJECTS WITH ACTIVE ATOPIC DERMATITIS | N | 2 |
| POX-MVA-008-2018-105 | VISIT 1 | 1 | 1 | VACCINIA NAIVE SUBJECTS WITH ACTIVE ATOPIC DERMATITIS | N | 1 |
| POX-MVA-008-2018-105 | VISIT 5 | 351 | 129 | VACCINIA NAIVE SUBJECTS WITH ACTIVE ATOPIC DERMATITIS | N | 1 |
| POX-MVA-008-2018-116 | VISIT 1 | 1 | 1 | VACCINIA NAIVE SUBJECTS WITH ACTIVE ATOPIC DERMATITIS | N | 1 |
| POX-MVA-008-2018-116 | VISIT 2 | 50 | 1 | VACCINIA NAIVE SUBJECTS WITH ACTIVE ATOPIC DERMATITIS | N | 1 |
| POX-MVA-008-2018-116 | VISIT 5 | 50 | 15 | VACCINIA NAIVE SUBJECTS WITH ACTIVE ATOPIC DERMATITIS | N | 1 |
| POX-MVA-008-2020-003 | VISIT 1 | 1 | 1 | HEALTHY, VACCINIA NAIVE SUBJECTS WITHOUT ATOPIC DISEASE | N | 2 |
| POX-MVA-008-2020-003 | VISIT 2 | 1 | 1 | HEALTHY, VACCINIA NAIVE SUBJECTS WITHOUT ATOPIC DISEASE | N | 2 |
| POX-MVA-008-2020-003 | VISIT 3 | 50 | 15 | HEALTHY, VACCINIA NAIVE SUBJECTS WITHOUT ATOPIC DISEASE | N | 2 |
| POX-MVA-008-2020-003 | VISIT 4 | 200 | 15 | HEALTHY, VACCINIA NAIVE SUBJECTS WITHOUT ATOPIC DISEASE | N | 2 |
| POX-MVA-008-2020-003 | VISIT 5 | 100 | 15 | HEALTHY, VACCINIA NAIVE SUBJECTS WITHOUT ATOPIC DISEASE | N | 2 |
| POX-MVA-008-2020-003 | FOLLOW-UP VISIT | 1 | 1 | HEALTHY, VACCINIA NAIVE SUBJECTS WITHOUT ATOPIC DISEASE | N | 2 |
| POX-MVA-008-2020-004 | VISIT 1 | 1 | 1 | HEALTHY, VACCINIA NAIVE SUBJECTS WITHOUT ATOPIC DISEASE | N | 2 |
| POX-MVA-008-2020-004 | VISIT 2 | 1 | 1 | HEALTHY, VACCINIA NAIVE SUBJECTS WITHOUT ATOPIC DISEASE | N | 2 |
| POX-MVA-008-2020-004 | VISIT 3 | 50 | 15 | HEALTHY, VACCINIA NAIVE SUBJECTS WITHOUT ATOPIC DISEASE | N | 2 |
| POX-MVA-008-2020-004 | VISIT 4 | 333 | 109 | HEALTHY, VACCINIA NAIVE SUBJECTS WITHOUT ATOPIC DISEASE | N | 2 |
| POX-MVA-008-2020-004 | VISIT 5 | 203 | 15 | HEALTHY, VACCINIA NAIVE SUBJECTS WITHOUT ATOPIC DISEASE | N | 2 |
| POX-MVA-008-2020-004 | FOLLOW-UP VISIT | 100 | 15 | HEALTHY, VACCINIA NAIVE SUBJECTS WITHOUT ATOPIC DISEASE | N | 2 |
| POX-MVA-008-2020-007 | VISIT 1 | 1 | 1 | HEALTHY, VACCINIA NAIVE SUBJECTS WITHOUT ATOPIC DISEASE | N | 2 |
| POX-MVA-008-2020-007 | VISIT 2 | 1 | 1 | HEALTHY, VACCINIA NAIVE SUBJECTS WITHOUT ATOPIC DISEASE | N | 2 |
| POX-MVA-008-2020-007 | VISIT 3 | 100 | 15 | HEALTHY, VACCINIA NAIVE SUBJECTS WITHOUT ATOPIC DISEASE | N | 2 |
| POX-MVA-008-2020-007 | VISIT 4 | 593 | 158 | HEALTHY, VACCINIA NAIVE SUBJECTS WITHOUT ATOPIC DISEASE | N | 2 |
| POX-MVA-008-2020-007 | VISIT 5 | 249 | 254 | HEALTHY, VACCINIA NAIVE SUBJECTS WITHOUT ATOPIC DISEASE | N | 2 |
| POX-MVA-008-2020-007 | FOLLOW-UP VISIT | 50 | 15 | HEALTHY, VACCINIA NAIVE SUBJECTS WITHOUT ATOPIC DISEASE | N | 2 |
| POX-MVA-008-2020-009 | VISIT 1 | 1 | 1 | VACCINIA NAIVE SUBJECTS WITH HISTORY OF ATOPIC DERMATITIS | N | 2 |
| POX-MVA-008-2020-009 | VISIT 2 | 1 | 1 | VACCINIA NAIVE SUBJECTS WITH HISTORY OF ATOPIC DERMATITIS | N | 2 |
| POX-MVA-008-2020-009 | VISIT 3 | 50 | 1 | VACCINIA NAIVE SUBJECTS WITH HISTORY OF ATOPIC DERMATITIS | N | 2 |
| POX-MVA-008-2020-009 | VISIT 4 | 251 | 15 | VACCINIA NAIVE SUBJECTS WITH HISTORY OF ATOPIC DERMATITIS | N | 2 |
| POX-MVA-008-2020-009 | VISIT 5 | 100 | 15 | VACCINIA NAIVE SUBJECTS WITH HISTORY OF ATOPIC DERMATITIS | N | 2 |
| POX-MVA-008-2020-009 | FOLLOW-UP VISIT | 50 | 15 | VACCINIA NAIVE SUBJECTS WITH HISTORY OF ATOPIC DERMATITIS | N | 2 |
| POX-MVA-008-2020-013 | VISIT 1 | 1 | 1 | VACCINIA NAIVE SUBJECTS WITH ACTIVE ATOPIC DERMATITIS | N | 2 |
| POX-MVA-008-2020-013 | VISIT 2 | 1 | 1 | VACCINIA NAIVE SUBJECTS WITH ACTIVE ATOPIC DERMATITIS | N | 2 |
| POX-MVA-008-2020-013 | VISIT 3 | 50 | 1 | VACCINIA NAIVE SUBJECTS WITH ACTIVE ATOPIC DERMATITIS | N | 2 |
| POX-MVA-008-2020-013 | VISIT 4 | 470 | 15 | VACCINIA NAIVE SUBJECTS WITH ACTIVE ATOPIC DERMATITIS | N | 2 |
| POX-MVA-008-2020-013 | VISIT 5 | 261 | 15 | VACCINIA NAIVE SUBJECTS WITH ACTIVE ATOPIC DERMATITIS | N | 2 |
| POX-MVA-008-2020-013 | FOLLOW-UP VISIT | 50 | 1 | VACCINIA NAIVE SUBJECTS WITH ACTIVE ATOPIC DERMATITIS | N | 2 |
| POX-MVA-008-2020-014 | VISIT 1 | 1 | 1 | HEALTHY, VACCINIA NAIVE SUBJECTS WITHOUT ATOPIC DISEASE | N | 2 |
| POX-MVA-008-2020-014 | VISIT 2 | 200 | 1 | HEALTHY, VACCINIA NAIVE SUBJECTS WITHOUT ATOPIC DISEASE | N | 2 |
| POX-MVA-008-2020-014 | VISIT 3 | 249 | 1 | HEALTHY, VACCINIA NAIVE SUBJECTS WITHOUT ATOPIC DISEASE | N | 2 |
| POX-MVA-008-2020-014 | VISIT 4 | 1892 | 15 | HEALTHY, VACCINIA NAIVE SUBJECTS WITHOUT ATOPIC DISEASE | N | 2 |
| POX-MVA-008-2020-014 | VISIT 5 | 890 | 15 | HEALTHY, VACCINIA NAIVE SUBJECTS WITHOUT ATOPIC DISEASE | N | 2 |
| POX-MVA-008-2020-015 | VISIT 1 | 1 | 1 | VACCINIA NAIVE SUBJECTS WITH HISTORY OF ATOPIC DERMATITIS | N | 2 |
| POX-MVA-008-2020-015 | VISIT 2 | 1 | 1 | VACCINIA NAIVE SUBJECTS WITH HISTORY OF ATOPIC DERMATITIS | N | 2 |
| POX-MVA-008-2020-015 | VISIT 3 | 100 | 15 | VACCINIA NAIVE SUBJECTS WITH HISTORY OF ATOPIC DERMATITIS | N | 2 |
| POX-MVA-008-2020-015 | VISIT 4 | 379 | 15 | VACCINIA NAIVE SUBJECTS WITH HISTORY OF ATOPIC DERMATITIS | N | 2 |
| POX-MVA-008-2020-015 | VISIT 5 | 285 | 15 | VACCINIA NAIVE SUBJECTS WITH HISTORY OF ATOPIC DERMATITIS | N | 2 |
| POX-MVA-008-2020-015 | FOLLOW-UP VISIT | 100 | 15 | VACCINIA NAIVE SUBJECTS WITH HISTORY OF ATOPIC DERMATITIS | N | 2 |
| POX-MVA-008-2020-017 | VISIT 1 | 50 | 1 | HEALTHY, VACCINIA NAIVE SUBJECTS WITHOUT ATOPIC DISEASE | N | 2 |
| POX-MVA-008-2020-017 | VISIT 2 | 50 | 1 | HEALTHY, VACCINIA NAIVE SUBJECTS WITHOUT ATOPIC DISEASE | N | 2 |
| POX-MVA-008-2020-017 | VISIT 3 | 267 | 1 | HEALTHY, VACCINIA NAIVE SUBJECTS WITHOUT ATOPIC DISEASE | N | 2 |
| POX-MVA-008-2020-017 | VISIT 4 | 378 | 15 | HEALTHY, VACCINIA NAIVE SUBJECTS WITHOUT ATOPIC DISEASE | N | 2 |
| POX-MVA-008-2020-017 | UNSCHEDULED VISIT 5A | 322 | 15 | HEALTHY, VACCINIA NAIVE SUBJECTS WITHOUT ATOPIC DISEASE | N | 2 |
| POX-MVA-008-2020-017 | FOLLOW-UP VISIT | 100 | 1 | HEALTHY, VACCINIA NAIVE SUBJECTS WITHOUT ATOPIC DISEASE | N | 2 |
| POX-MVA-008-2020-020 | VISIT 1 | 1 | 1 | HEALTHY, VACCINIA NAIVE SUBJECTS WITHOUT ATOPIC DISEASE | N | 2 |
| POX-MVA-008-2020-020 | VISIT 2 | 1 | 1 | HEALTHY, VACCINIA NAIVE SUBJECTS WITHOUT ATOPIC DISEASE | N | 2 |
| POX-MVA-008-2020-020 | VISIT 3 | 50 | 1 | HEALTHY, VACCINIA NAIVE SUBJECTS WITHOUT ATOPIC DISEASE | N | 2 |
| POX-MVA-008-2020-020 | VISIT 4 | 236 | 15 | HEALTHY, VACCINIA NAIVE SUBJECTS WITHOUT ATOPIC DISEASE | N | 2 |
| POX-MVA-008-2020-020 | VISIT 5 | 233 | 15 | HEALTHY, VACCINIA NAIVE SUBJECTS WITHOUT ATOPIC DISEASE | N | 2 |
| POX-MVA-008-2020-022 | VISIT 1 | 1 | 1 | HEALTHY, VACCINIA NAIVE SUBJECTS WITHOUT ATOPIC DISEASE | N | 2 |
| POX-MVA-008-2020-022 | VISIT 2 | 1 | 1 | HEALTHY, VACCINIA NAIVE SUBJECTS WITHOUT ATOPIC DISEASE | N | 2 |
| POX-MVA-008-2020-022 | VISIT 3 | 100 | 15 | HEALTHY, VACCINIA NAIVE SUBJECTS WITHOUT ATOPIC DISEASE | N | 2 |
| POX-MVA-008-2020-022 | VISIT 4 | 2573 | 259 | HEALTHY, VACCINIA NAIVE SUBJECTS WITHOUT ATOPIC DISEASE | N | 2 |
| POX-MVA-008-2020-022 | VISIT 5 | 778 | 159 | HEALTHY, VACCINIA NAIVE SUBJECTS WITHOUT ATOPIC DISEASE | N | 2 |
| POX-MVA-008-2020-025 | VISIT 1 | 1 | 1 | HEALTHY, VACCINIA NAIVE SUBJECTS WITHOUT ATOPIC DISEASE | N | 2 |
| POX-MVA-008-2020-025 | VISIT 2 | 1 | 1 | HEALTHY, VACCINIA NAIVE SUBJECTS WITHOUT ATOPIC DISEASE | N | 2 |
| POX-MVA-008-2020-025 | VISIT 3 | 200 | 1 | HEALTHY, VACCINIA NAIVE SUBJECTS WITHOUT ATOPIC DISEASE | N | 2 |
| POX-MVA-008-2020-025 | VISIT 4 | 861 | 101 | HEALTHY, VACCINIA NAIVE SUBJECTS WITHOUT ATOPIC DISEASE | N | 2 |
| POX-MVA-008-2020-025 | VISIT 5 | 315 | 15 | HEALTHY, VACCINIA NAIVE SUBJECTS WITHOUT ATOPIC DISEASE | N | 2 |
| POX-MVA-008-2020-025 | FOLLOW-UP VISIT | 100 | 1 | HEALTHY, VACCINIA NAIVE SUBJECTS WITHOUT ATOPIC DISEASE | N | 2 |
| POX-MVA-008-2020-026 | VISIT 1 | 1 | 1 | HEALTHY, VACCINIA NAIVE SUBJECTS WITHOUT ATOPIC DISEASE | N | 2 |
| POX-MVA-008-2020-026 | VISIT 2 | 1 | 1 | HEALTHY, VACCINIA NAIVE SUBJECTS WITHOUT ATOPIC DISEASE | N | 2 |
| POX-MVA-008-2020-026 | VISIT 3 | 50 | 1 | HEALTHY, VACCINIA NAIVE SUBJECTS WITHOUT ATOPIC DISEASE | N | 2 |
| POX-MVA-008-2020-026 | VISIT 4 | 273 | 15 | HEALTHY, VACCINIA NAIVE SUBJECTS WITHOUT ATOPIC DISEASE | N | 2 |
| POX-MVA-008-2020-026 | VISIT 5 | 253 | 1 | HEALTHY, VACCINIA NAIVE SUBJECTS WITHOUT ATOPIC DISEASE | N | 2 |
| POX-MVA-008-2020-030 | VISIT 1 | 1 | 1 | HEALTHY, VACCINIA NAIVE SUBJECTS WITHOUT ATOPIC DISEASE | N | 2 |
| POX-MVA-008-2020-030 | VISIT 2 | 265 | 1 | HEALTHY, VACCINIA NAIVE SUBJECTS WITHOUT ATOPIC DISEASE | N | 2 |
| POX-MVA-008-2020-030 | VISIT 3 | 728 | 1 | HEALTHY, VACCINIA NAIVE SUBJECTS WITHOUT ATOPIC DISEASE | N | 2 |
| POX-MVA-008-2020-030 | VISIT 4 | 889 | 109 | HEALTHY, VACCINIA NAIVE SUBJECTS WITHOUT ATOPIC DISEASE | N | 2 |
| POX-MVA-008-2020-030 | VISIT 5 | 846 | 15 | HEALTHY, VACCINIA NAIVE SUBJECTS WITHOUT ATOPIC DISEASE | N | 2 |
| POX-MVA-008-2020-032 | VISIT 1 | 1 | 1 | HEALTHY, VACCINIA NAIVE SUBJECTS WITHOUT ATOPIC DISEASE | N | 1 |
| POX-MVA-008-2020-032 | VISIT 2 | 100 | 15 | HEALTHY, VACCINIA NAIVE SUBJECTS WITHOUT ATOPIC DISEASE | N | 1 |
| POX-MVA-008-2020-036 | VISIT 1 | 1 | 1 | VACCINIA NAIVE SUBJECTS WITH HISTORY OF ATOPIC DERMATITIS | N | 2 |
| POX-MVA-008-2020-036 | VISIT 2 | 1 | 1 | VACCINIA NAIVE SUBJECTS WITH HISTORY OF ATOPIC DERMATITIS | N | 2 |
| POX-MVA-008-2020-036 | VISIT 3 | 204 | 1 | VACCINIA NAIVE SUBJECTS WITH HISTORY OF ATOPIC DERMATITIS | N | 2 |
| POX-MVA-008-2020-036 | VISIT 4 | 370 | 82 | VACCINIA NAIVE SUBJECTS WITH HISTORY OF ATOPIC DERMATITIS | N | 2 |
| POX-MVA-008-2020-036 | VISIT 5 | 433 | 15 | VACCINIA NAIVE SUBJECTS WITH HISTORY OF ATOPIC DERMATITIS | N | 2 |
| POX-MVA-008-2020-043 | VISIT 1 | 1 | 1 | HEALTHY, VACCINIA NAIVE SUBJECTS WITHOUT ATOPIC DISEASE | N | 2 |
| POX-MVA-008-2020-043 | VISIT 2 | 1 | 1 | HEALTHY, VACCINIA NAIVE SUBJECTS WITHOUT ATOPIC DISEASE | N | 2 |
| POX-MVA-008-2020-043 | VISIT 3 | 200 | 1 | HEALTHY, VACCINIA NAIVE SUBJECTS WITHOUT ATOPIC DISEASE | N | 2 |
| POX-MVA-008-2020-043 | VISIT 4 | 22172 | 239 | HEALTHY, VACCINIA NAIVE SUBJECTS WITHOUT ATOPIC DISEASE | N | 2 |
| POX-MVA-008-2020-043 | VISIT 5 | 7052 | 297 | HEALTHY, VACCINIA NAIVE SUBJECTS WITHOUT ATOPIC DISEASE | N | 2 |
| POX-MVA-008-2020-047 | VISIT 1 | 1 | 1 | VACCINIA NAIVE SUBJECTS WITH ACTIVE ATOPIC DERMATITIS | N | 2 |
| POX-MVA-008-2020-047 | VISIT 2 | 1 | 1 | VACCINIA NAIVE SUBJECTS WITH ACTIVE ATOPIC DERMATITIS | N | 2 |
| POX-MVA-008-2020-047 | VISIT 3 | 50 | 15 | VACCINIA NAIVE SUBJECTS WITH ACTIVE ATOPIC DERMATITIS | N | 2 |
| POX-MVA-008-2020-047 | VISIT 4 | 1010 | 411 | VACCINIA NAIVE SUBJECTS WITH ACTIVE ATOPIC DERMATITIS | N | 2 |
| POX-MVA-008-2020-047 | VISIT 5 | 305 | 161 | VACCINIA NAIVE SUBJECTS WITH ACTIVE ATOPIC DERMATITIS | N | 2 |
| POX-MVA-008-2020-049 | VISIT 1 | 1 | 1 | VACCINIA NAIVE SUBJECTS WITH ACTIVE ATOPIC DERMATITIS | N | 2 |
| POX-MVA-008-2020-049 | VISIT 2 | 244 | 1 | VACCINIA NAIVE SUBJECTS WITH ACTIVE ATOPIC DERMATITIS | N | 2 |
| POX-MVA-008-2020-049 | VISIT 3 | 200 | 1 | VACCINIA NAIVE SUBJECTS WITH ACTIVE ATOPIC DERMATITIS | N | 2 |
| POX-MVA-008-2020-049 | VISIT 4 | 391 | 76 | VACCINIA NAIVE SUBJECTS WITH ACTIVE ATOPIC DERMATITIS | N | 2 |
| POX-MVA-008-2020-049 | VISIT 5 | 332 | 15 | VACCINIA NAIVE SUBJECTS WITH ACTIVE ATOPIC DERMATITIS | N | 2 |
| POX-MVA-008-2020-049 | FOLLOW-UP VISIT | 1 | 1 | VACCINIA NAIVE SUBJECTS WITH ACTIVE ATOPIC DERMATITIS | N | 2 |
| POX-MVA-008-2020-050 | VISIT 1 | 1 | 1 | VACCINIA NAIVE SUBJECTS WITH ACTIVE ATOPIC DERMATITIS | N | 2 |
| POX-MVA-008-2020-050 | VISIT 2 | 1 | 1 | VACCINIA NAIVE SUBJECTS WITH ACTIVE ATOPIC DERMATITIS | N | 2 |
| POX-MVA-008-2020-050 | VISIT 3 | 371 | 15 | VACCINIA NAIVE SUBJECTS WITH ACTIVE ATOPIC DERMATITIS | N | 2 |
| POX-MVA-008-2020-050 | VISIT 4 | 1128 | 139 | VACCINIA NAIVE SUBJECTS WITH ACTIVE ATOPIC DERMATITIS | N | 2 |
| POX-MVA-008-2020-050 | VISIT 5 | 592 | 309 | VACCINIA NAIVE SUBJECTS WITH ACTIVE ATOPIC DERMATITIS | N | 2 |
| POX-MVA-008-2020-051 | VISIT 1 | 1 | 1 | VACCINIA NAIVE SUBJECTS WITH ACTIVE ATOPIC DERMATITIS | N | 2 |
| POX-MVA-008-2020-051 | VISIT 2 | 1 | 1 | VACCINIA NAIVE SUBJECTS WITH ACTIVE ATOPIC DERMATITIS | N | 2 |
| POX-MVA-008-2020-051 | VISIT 3 | 1 | 1 | VACCINIA NAIVE SUBJECTS WITH ACTIVE ATOPIC DERMATITIS | N | 2 |
| POX-MVA-008-2020-051 | VISIT 4 | 456 | 15 | VACCINIA NAIVE SUBJECTS WITH ACTIVE ATOPIC DERMATITIS | N | 2 |
| POX-MVA-008-2020-051 | VISIT 5 | 318 | 1 | VACCINIA NAIVE SUBJECTS WITH ACTIVE ATOPIC DERMATITIS | N | 2 |
| POX-MVA-008-2021-010 | VISIT 1 | 1 | 1 | VACCINIA NAIVE SUBJECTS WITH HISTORY OF ATOPIC DERMATITIS | N | 2 |
| POX-MVA-008-2021-010 | VISIT 2 | 1 | 1 | VACCINIA NAIVE SUBJECTS WITH HISTORY OF ATOPIC DERMATITIS | N | 2 |
| POX-MVA-008-2021-010 | VISIT 3 | 100 | 15 | VACCINIA NAIVE SUBJECTS WITH HISTORY OF ATOPIC DERMATITIS | N | 2 |
| POX-MVA-008-2021-014 | VISIT 1 | 1 | 1 | VACCINIA NAIVE SUBJECTS WITH ACTIVE ATOPIC DERMATITIS | N | 2 |
| POX-MVA-008-2021-014 | VISIT 2 | 50 | 1 | VACCINIA NAIVE SUBJECTS WITH ACTIVE ATOPIC DERMATITIS | N | 2 |
| POX-MVA-008-2021-014 | VISIT 3 | 221 | 1 | VACCINIA NAIVE SUBJECTS WITH ACTIVE ATOPIC DERMATITIS | N | 2 |
| POX-MVA-008-2021-014 | VISIT 4 | 1175 | 159 | VACCINIA NAIVE SUBJECTS WITH ACTIVE ATOPIC DERMATITIS | N | 2 |
| POX-MVA-008-2021-014 | VISIT 5 | 617 | 15 | VACCINIA NAIVE SUBJECTS WITH ACTIVE ATOPIC DERMATITIS | N | 2 |
| POX-MVA-008-2021-017 | VISIT 1 | 1 | 1 | VACCINIA NAIVE SUBJECTS WITH ACTIVE ATOPIC DERMATITIS | N | 2 |
| POX-MVA-008-2021-017 | VISIT 2 | 1 | 1 | VACCINIA NAIVE SUBJECTS WITH ACTIVE ATOPIC DERMATITIS | N | 2 |
| POX-MVA-008-2021-017 | VISIT 3 | 100 | 1 | VACCINIA NAIVE SUBJECTS WITH ACTIVE ATOPIC DERMATITIS | N | 2 |
| POX-MVA-008-2021-017 | VISIT 4 | 265 | 15 | VACCINIA NAIVE SUBJECTS WITH ACTIVE ATOPIC DERMATITIS | N | 2 |
| POX-MVA-008-2021-017 | VISIT 5 | 294 | 15 | VACCINIA NAIVE SUBJECTS WITH ACTIVE ATOPIC DERMATITIS | N | 2 |
| POX-MVA-008-2023-030 | VISIT 1 | 1 | 1 | VACCINIA NAIVE SUBJECTS WITH HISTORY OF ATOPIC DERMATITIS | N | 2 |
| POX-MVA-008-2023-030 | VISIT 2 | 1 | 1 | VACCINIA NAIVE SUBJECTS WITH HISTORY OF ATOPIC DERMATITIS | N | 2 |
| POX-MVA-008-2023-030 | VISIT 3 | 1 | 1 | VACCINIA NAIVE SUBJECTS WITH HISTORY OF ATOPIC DERMATITIS | N | 2 |
| POX-MVA-008-2023-030 | VISIT 4 | 585 | 100 | VACCINIA NAIVE SUBJECTS WITH HISTORY OF ATOPIC DERMATITIS | N | 2 |
| POX-MVA-008-2023-032 | VISIT 1 | 1 | 1 | VACCINIA NAIVE SUBJECTS WITH ACTIVE ATOPIC DERMATITIS | N | 1 |
| POX-MVA-008-2023-032 | VISIT 2 | 1 | 1 | VACCINIA NAIVE SUBJECTS WITH ACTIVE ATOPIC DERMATITIS | N | 1 |
| POX-MVA-008-2023-032 | VISIT 5 | 1 | 1 | VACCINIA NAIVE SUBJECTS WITH ACTIVE ATOPIC DERMATITIS | N | 1 |
| POX-MVA-008-2024-001 | VISIT 1 | 1 | 1 | VACCINIA NAIVE SUBJECTS WITH ACTIVE ATOPIC DERMATITIS | N | 2 |
| POX-MVA-008-2024-001 | VISIT 2 | 1 | 1 | VACCINIA NAIVE SUBJECTS WITH ACTIVE ATOPIC DERMATITIS | N | 2 |
| POX-MVA-008-2024-001 | VISIT 3 | 50 | 1 | VACCINIA NAIVE SUBJECTS WITH ACTIVE ATOPIC DERMATITIS | N | 2 |
| POX-MVA-008-2024-001 | VISIT 4 | 1787 | 558 | VACCINIA NAIVE SUBJECTS WITH ACTIVE ATOPIC DERMATITIS | N | 2 |
| POX-MVA-008-2024-001 | VISIT 5 | 800 | 198 | VACCINIA NAIVE SUBJECTS WITH ACTIVE ATOPIC DERMATITIS | N | 2 |
| POX-MVA-008-2024-001 | FOLLOW-UP VISIT | 50 | 15 | VACCINIA NAIVE SUBJECTS WITH ACTIVE ATOPIC DERMATITIS | N | 2 |
| POX-MVA-008-2024-002 | VISIT 1 | 1 | 1 | HEALTHY, VACCINIA NAIVE SUBJECTS WITHOUT ATOPIC DISEASE | N | 2 |
| POX-MVA-008-2024-002 | VISIT 2 | 1 | 1 | HEALTHY, VACCINIA NAIVE SUBJECTS WITHOUT ATOPIC DISEASE | N | 2 |
| POX-MVA-008-2024-002 | VISIT 3 | 235 | 15 | HEALTHY, VACCINIA NAIVE SUBJECTS WITHOUT ATOPIC DISEASE | N | 2 |
| POX-MVA-008-2024-002 | VISIT 4 | 1927 | 109 | HEALTHY, VACCINIA NAIVE SUBJECTS WITHOUT ATOPIC DISEASE | N | 2 |
| POX-MVA-008-2024-002 | VISIT 5 | 855 | 122 | HEALTHY, VACCINIA NAIVE SUBJECTS WITHOUT ATOPIC DISEASE | N | 2 |
| POX-MVA-008-2024-002 | FOLLOW-UP VISIT | 100 | 15 | HEALTHY, VACCINIA NAIVE SUBJECTS WITHOUT ATOPIC DISEASE | N | 2 |
| POX-MVA-008-2024-003 | VISIT 1 | 1 | 1 | HEALTHY, VACCINIA NAIVE SUBJECTS WITHOUT ATOPIC DISEASE | N | 2 |
| POX-MVA-008-2024-003 | VISIT 2 | 50 | 1 | HEALTHY, VACCINIA NAIVE SUBJECTS WITHOUT ATOPIC DISEASE | N | 2 |
| POX-MVA-008-2024-003 | VISIT 3 | 200 | 1 | HEALTHY, VACCINIA NAIVE SUBJECTS WITHOUT ATOPIC DISEASE | N | 2 |
| POX-MVA-008-2024-003 | VISIT 4 | 278 | 15 | HEALTHY, VACCINIA NAIVE SUBJECTS WITHOUT ATOPIC DISEASE | N | 2 |
| POX-MVA-008-2024-003 | VISIT 5 | 200 | 15 | HEALTHY, VACCINIA NAIVE SUBJECTS WITHOUT ATOPIC DISEASE | N | 2 |
| POX-MVA-008-2024-003 | FOLLOW-UP VISIT | 50 | 1 | HEALTHY, VACCINIA NAIVE SUBJECTS WITHOUT ATOPIC DISEASE | N | 2 |
| POX-MVA-008-2024-011 | VISIT 1 | 1 | 1 | VACCINIA NAIVE SUBJECTS WITH ACTIVE ATOPIC DERMATITIS | N | 2 |
| POX-MVA-008-2024-011 | VISIT 2 | 1 | 1 | VACCINIA NAIVE SUBJECTS WITH ACTIVE ATOPIC DERMATITIS | N | 2 |
| POX-MVA-008-2024-011 | VISIT 3 | 100 | 1 | VACCINIA NAIVE SUBJECTS WITH ACTIVE ATOPIC DERMATITIS | N | 2 |
| POX-MVA-008-2024-011 | VISIT 4 | 1919 | 122 | VACCINIA NAIVE SUBJECTS WITH ACTIVE ATOPIC DERMATITIS | N | 2 |
| POX-MVA-008-2024-011 | VISIT 5 | 704 | 15 | VACCINIA NAIVE SUBJECTS WITH ACTIVE ATOPIC DERMATITIS | N | 2 |
| POX-MVA-008-2024-011 | FOLLOW-UP VISIT | 50 | 15 | VACCINIA NAIVE SUBJECTS WITH ACTIVE ATOPIC DERMATITIS | N | 2 |
| POX-MVA-008-2024-015 | VISIT 1 | 1 | 1 | HEALTHY, VACCINIA NAIVE SUBJECTS WITHOUT ATOPIC DISEASE | N | 2 |
| POX-MVA-008-2024-015 | VISIT 2 | 1 | 1 | HEALTHY, VACCINIA NAIVE SUBJECTS WITHOUT ATOPIC DISEASE | N | 2 |
| POX-MVA-008-2024-015 | VISIT 3 | 100 | 15 | HEALTHY, VACCINIA NAIVE SUBJECTS WITHOUT ATOPIC DISEASE | N | 2 |
| POX-MVA-008-2024-015 | VISIT 4 | 1642 | 126 | HEALTHY, VACCINIA NAIVE SUBJECTS WITHOUT ATOPIC DISEASE | N | 2 |
| POX-MVA-008-2024-015 | VISIT 5 | 224 | 15 | HEALTHY, VACCINIA NAIVE SUBJECTS WITHOUT ATOPIC DISEASE | N | 2 |
| POX-MVA-008-2024-025 | VISIT 1 | 1 | 1 | VACCINIA NAIVE SUBJECTS WITH ACTIVE ATOPIC DERMATITIS | N | 2 |
| POX-MVA-008-2024-025 | VISIT 2 | 1 | 1 | VACCINIA NAIVE SUBJECTS WITH ACTIVE ATOPIC DERMATITIS | N | 2 |
| POX-MVA-008-2024-025 | VISIT 3 | 50 | 1 | VACCINIA NAIVE SUBJECTS WITH ACTIVE ATOPIC DERMATITIS | N | 2 |
| POX-MVA-008-2024-025 | VISIT 4 | 241 | 81 | VACCINIA NAIVE SUBJECTS WITH ACTIVE ATOPIC DERMATITIS | N | 2 |
| POX-MVA-008-2024-025 | VISIT 5 | 1990 | 225 | VACCINIA NAIVE SUBJECTS WITH ACTIVE ATOPIC DERMATITIS | N | 2 |
| POX-MVA-008-2025-001 | VISIT 1 | 1 | 1 | HEALTHY, VACCINIA NAIVE SUBJECTS WITHOUT ATOPIC DISEASE | N | 2 |
| POX-MVA-008-2025-001 | VISIT 2 | 1 | 1 | HEALTHY, VACCINIA NAIVE SUBJECTS WITHOUT ATOPIC DISEASE | N | 2 |
| POX-MVA-008-2025-001 | VISIT 3 | 50 | 1 | HEALTHY, VACCINIA NAIVE SUBJECTS WITHOUT ATOPIC DISEASE | N | 2 |
| POX-MVA-008-2025-001 | VISIT 4 | 299 | 15 | HEALTHY, VACCINIA NAIVE SUBJECTS WITHOUT ATOPIC DISEASE | N | 2 |
| POX-MVA-008-2025-001 | VISIT 5 | 293 | 15 | HEALTHY, VACCINIA NAIVE SUBJECTS WITHOUT ATOPIC DISEASE | N | 2 |
| POX-MVA-008-2025-001 | FOLLOW-UP VISIT | 100 | 15 | HEALTHY, VACCINIA NAIVE SUBJECTS WITHOUT ATOPIC DISEASE | N | 2 |
| POX-MVA-008-2025-003 | VISIT 1 | 1 | 1 | HEALTHY, VACCINIA NAIVE SUBJECTS WITHOUT ATOPIC DISEASE | N | 2 |
| POX-MVA-008-2025-003 | VISIT 2 | 1 | 1 | HEALTHY, VACCINIA NAIVE SUBJECTS WITHOUT ATOPIC DISEASE | N | 2 |
| POX-MVA-008-2025-003 | VISIT 3 | 50 | 1 | HEALTHY, VACCINIA NAIVE SUBJECTS WITHOUT ATOPIC DISEASE | N | 2 |
| POX-MVA-008-2025-003 | VISIT 4 | 907 | 188 | HEALTHY, VACCINIA NAIVE SUBJECTS WITHOUT ATOPIC DISEASE | N | 2 |
| POX-MVA-008-2025-003 | VISIT 5 | 375 | 15 | HEALTHY, VACCINIA NAIVE SUBJECTS WITHOUT ATOPIC DISEASE | N | 2 |
| POX-MVA-008-2025-004 | VISIT 1 | 1 | 1 | VACCINIA NAIVE SUBJECTS WITH HISTORY OF ATOPIC DERMATITIS | N | 2 |
| POX-MVA-008-2025-004 | VISIT 2 | 1 | 1 | VACCINIA NAIVE SUBJECTS WITH HISTORY OF ATOPIC DERMATITIS | N | 2 |
| POX-MVA-008-2025-004 | VISIT 3 | 50 | 1 | VACCINIA NAIVE SUBJECTS WITH HISTORY OF ATOPIC DERMATITIS | N | 2 |
| POX-MVA-008-2025-004 | VISIT 4 | 275 | 99 | VACCINIA NAIVE SUBJECTS WITH HISTORY OF ATOPIC DERMATITIS | N | 2 |
| POX-MVA-008-2025-004 | VISIT 5 | 219 | 15 | VACCINIA NAIVE SUBJECTS WITH HISTORY OF ATOPIC DERMATITIS | N | 2 |
| POX-MVA-008-2025-004 | FOLLOW-UP VISIT | 50 | 1 | VACCINIA NAIVE SUBJECTS WITH HISTORY OF ATOPIC DERMATITIS | N | 2 |
| POX-MVA-008-2025-005 | VISIT 1 | 1 | 1 | HEALTHY, VACCINIA NAIVE SUBJECTS WITHOUT ATOPIC DISEASE | N | 1 |
| POX-MVA-008-2025-005 | VISIT 2 | 1 | 1 | HEALTHY, VACCINIA NAIVE SUBJECTS WITHOUT ATOPIC DISEASE | N | 1 |
| POX-MVA-008-2025-005 | VISIT 3 | 1 | 1 | HEALTHY, VACCINIA NAIVE SUBJECTS WITHOUT ATOPIC DISEASE | N | 1 |
| POX-MVA-008-2025-005 | VISIT 4 | 1 | 1 | HEALTHY, VACCINIA NAIVE SUBJECTS WITHOUT ATOPIC DISEASE | N | 1 |
| POX-MVA-008-2025-005 | VISIT 5 | 1 | 1 | HEALTHY, VACCINIA NAIVE SUBJECTS WITHOUT ATOPIC DISEASE | N | 1 |
| POX-MVA-008-2025-005 | FOLLOW-UP VISIT | 1 | 1 | HEALTHY, VACCINIA NAIVE SUBJECTS WITHOUT ATOPIC DISEASE | N | 1 |
| POX-MVA-008-2025-006 | VISIT 1 | 1 | 1 | HEALTHY, VACCINIA NAIVE SUBJECTS WITHOUT ATOPIC DISEASE | N | 2 |
| POX-MVA-008-2025-006 | VISIT 2 | 1 | 1 | HEALTHY, VACCINIA NAIVE SUBJECTS WITHOUT ATOPIC DISEASE | N | 2 |
| POX-MVA-008-2025-006 | VISIT 3 | 50 | 1 | HEALTHY, VACCINIA NAIVE SUBJECTS WITHOUT ATOPIC DISEASE | N | 2 |
| POX-MVA-008-2025-006 | VISIT 4 | 436 | 120 | HEALTHY, VACCINIA NAIVE SUBJECTS WITHOUT ATOPIC DISEASE | N | 2 |
| POX-MVA-008-2025-006 | VISIT 5 | 356 | 97 | HEALTHY, VACCINIA NAIVE SUBJECTS WITHOUT ATOPIC DISEASE | N | 2 |
| POX-MVA-008-2025-006 | FOLLOW-UP VISIT | 50 | 1 | HEALTHY, VACCINIA NAIVE SUBJECTS WITHOUT ATOPIC DISEASE | N | 2 |
| POX-MVA-008-2025-008 | VISIT 1 | 1 | 1 | HEALTHY, VACCINIA NAIVE SUBJECTS WITHOUT ATOPIC DISEASE | N | 2 |
| POX-MVA-008-2025-008 | VISIT 2 | 1 | 1 | HEALTHY, VACCINIA NAIVE SUBJECTS WITHOUT ATOPIC DISEASE | N | 2 |
| POX-MVA-008-2025-008 | VISIT 3 | 50 | 1 | HEALTHY, VACCINIA NAIVE SUBJECTS WITHOUT ATOPIC DISEASE | N | 2 |
| POX-MVA-008-2025-008 | VISIT 4 | 476 | 256 | HEALTHY, VACCINIA NAIVE SUBJECTS WITHOUT ATOPIC DISEASE | N | 2 |
| POX-MVA-008-2025-008 | VISIT 5 | 393 | 15 | HEALTHY, VACCINIA NAIVE SUBJECTS WITHOUT ATOPIC DISEASE | N | 2 |
| POX-MVA-008-2025-009 | VISIT 1 | 1 | 1 | HEALTHY, VACCINIA NAIVE SUBJECTS WITHOUT ATOPIC DISEASE | N | 2 |
| POX-MVA-008-2025-009 | VISIT 2 | 1 | 1 | HEALTHY, VACCINIA NAIVE SUBJECTS WITHOUT ATOPIC DISEASE | N | 2 |
| POX-MVA-008-2025-009 | VISIT 3 | 100 | 1 | HEALTHY, VACCINIA NAIVE SUBJECTS WITHOUT ATOPIC DISEASE | N | 2 |
| POX-MVA-008-2025-009 | VISIT 4 | 566 | 183 | HEALTHY, VACCINIA NAIVE SUBJECTS WITHOUT ATOPIC DISEASE | N | 2 |
| POX-MVA-008-2025-009 | VISIT 5 | 384 | 231 | HEALTHY, VACCINIA NAIVE SUBJECTS WITHOUT ATOPIC DISEASE | N | 2 |
| POX-MVA-008-2025-011 | VISIT 1 | 1 | 1 | HEALTHY, VACCINIA NAIVE SUBJECTS WITHOUT ATOPIC DISEASE | N | 2 |
| POX-MVA-008-2025-011 | VISIT 2 | 1 | 1 | HEALTHY, VACCINIA NAIVE SUBJECTS WITHOUT ATOPIC DISEASE | N | 2 |
| POX-MVA-008-2025-011 | VISIT 3 | 100 | 15 | HEALTHY, VACCINIA NAIVE SUBJECTS WITHOUT ATOPIC DISEASE | N | 2 |
| POX-MVA-008-2025-011 | VISIT 4 | 828 | 206 | HEALTHY, VACCINIA NAIVE SUBJECTS WITHOUT ATOPIC DISEASE | N | 2 |
| POX-MVA-008-2025-011 | VISIT 5 | 1702 | 239 | HEALTHY, VACCINIA NAIVE SUBJECTS WITHOUT ATOPIC DISEASE | N | 2 |
| POX-MVA-008-2025-011 | FOLLOW-UP VISIT | 100 | 15 | HEALTHY, VACCINIA NAIVE SUBJECTS WITHOUT ATOPIC DISEASE | N | 2 |
| POX-MVA-008-2025-012 | VISIT 1 | 1 | 1 | HEALTHY, VACCINIA NAIVE SUBJECTS WITHOUT ATOPIC DISEASE | N | 2 |
| POX-MVA-008-2025-012 | VISIT 2 | 1 | 1 | HEALTHY, VACCINIA NAIVE SUBJECTS WITHOUT ATOPIC DISEASE | N | 2 |
| POX-MVA-008-2025-012 | VISIT 3 | 100 | 1 | HEALTHY, VACCINIA NAIVE SUBJECTS WITHOUT ATOPIC DISEASE | N | 2 |
| POX-MVA-008-2025-012 | VISIT 4 | 308 | 15 | HEALTHY, VACCINIA NAIVE SUBJECTS WITHOUT ATOPIC DISEASE | N | 2 |
| POX-MVA-008-2025-012 | VISIT 5 | 349 | 15 | HEALTHY, VACCINIA NAIVE SUBJECTS WITHOUT ATOPIC DISEASE | N | 2 |
| POX-MVA-008-2025-026 | VISIT 1 | 1 | 1 | HEALTHY, VACCINIA NAIVE SUBJECTS WITHOUT ATOPIC DISEASE | N | 2 |
| POX-MVA-008-2025-026 | VISIT 2 | 1 | 1 | HEALTHY, VACCINIA NAIVE SUBJECTS WITHOUT ATOPIC DISEASE | N | 2 |
| POX-MVA-008-2025-026 | VISIT 3 | 50 | 1 | HEALTHY, VACCINIA NAIVE SUBJECTS WITHOUT ATOPIC DISEASE | N | 2 |
| POX-MVA-008-2025-026 | VISIT 4 | 652 | 15 | HEALTHY, VACCINIA NAIVE SUBJECTS WITHOUT ATOPIC DISEASE | N | 2 |
| POX-MVA-008-2025-026 | VISIT 5 | 287 | 15 | HEALTHY, VACCINIA NAIVE SUBJECTS WITHOUT ATOPIC DISEASE | N | 2 |
| POX-MVA-008-2025-028 | VISIT 1 | 1 | 1 | HEALTHY, VACCINIA NAIVE SUBJECTS WITHOUT ATOPIC DISEASE | N | 2 |
| POX-MVA-008-2025-028 | VISIT 2 | 1 | 1 | HEALTHY, VACCINIA NAIVE SUBJECTS WITHOUT ATOPIC DISEASE | N | 2 |
| POX-MVA-008-2025-028 | VISIT 3 | 200 | 15 | HEALTHY, VACCINIA NAIVE SUBJECTS WITHOUT ATOPIC DISEASE | N | 2 |
| POX-MVA-008-2025-028 | VISIT 4 | 582 | 138 | HEALTHY, VACCINIA NAIVE SUBJECTS WITHOUT ATOPIC DISEASE | N | 2 |
| POX-MVA-008-2025-028 | VISIT 5 | 547 | 15 | HEALTHY, VACCINIA NAIVE SUBJECTS WITHOUT ATOPIC DISEASE | N | 2 |
| POX-MVA-008-2025-029 | VISIT 1 | 1 | 1 | VACCINIA NAIVE SUBJECTS WITH ACTIVE ATOPIC DERMATITIS | N | 2 |
| POX-MVA-008-2025-029 | VISIT 2 | 1 | 1 | VACCINIA NAIVE SUBJECTS WITH ACTIVE ATOPIC DERMATITIS | N | 2 |
| POX-MVA-008-2025-029 | VISIT 3 | 100 | 1 | VACCINIA NAIVE SUBJECTS WITH ACTIVE ATOPIC DERMATITIS | N | 2 |
| POX-MVA-008-2025-029 | VISIT 4 | 379 | 124 | VACCINIA NAIVE SUBJECTS WITH ACTIVE ATOPIC DERMATITIS | N | 2 |
| POX-MVA-008-2025-029 | VISIT 5 | 300 | 15 | VACCINIA NAIVE SUBJECTS WITH ACTIVE ATOPIC DERMATITIS | N | 2 |
| POX-MVA-008-2025-033 | VISIT 1 | 1 | 1 | HEALTHY, VACCINIA NAIVE SUBJECTS WITHOUT ATOPIC DISEASE | N | 1 |
| POX-MVA-008-2025-033 | VISIT 2 | 1 | 1 | HEALTHY, VACCINIA NAIVE SUBJECTS WITHOUT ATOPIC DISEASE | N | 1 |
| POX-MVA-008-2025-033 | VISIT 3 | 293 | 1 | HEALTHY, VACCINIA NAIVE SUBJECTS WITHOUT ATOPIC DISEASE | N | 1 |
| POX-MVA-008-2025-033 | VISIT 4 | 224 | 15 | HEALTHY, VACCINIA NAIVE SUBJECTS WITHOUT ATOPIC DISEASE | N | 1 |
| POX-MVA-008-2025-033 | VISIT 5 | 100 | 1 | HEALTHY, VACCINIA NAIVE SUBJECTS WITHOUT ATOPIC DISEASE | N | 1 |
| POX-MVA-008-2025-045 | VISIT 2 | 1 | 1 | VACCINIA NAIVE SUBJECTS WITH HISTORY OF ATOPIC DERMATITIS | N | 2 |
| POX-MVA-008-2025-045 | VISIT 3 | 50 | 1 | VACCINIA NAIVE SUBJECTS WITH HISTORY OF ATOPIC DERMATITIS | N | 2 |
| POX-MVA-008-2025-045 | VISIT 4 | 548 | 102 | VACCINIA NAIVE SUBJECTS WITH HISTORY OF ATOPIC DERMATITIS | N | 2 |
| POX-MVA-008-2025-045 | VISIT 5 | 332 | 15 | VACCINIA NAIVE SUBJECTS WITH HISTORY OF ATOPIC DERMATITIS | N | 2 |
| POX-MVA-008-2025-048 | VISIT 1 | 50 | 1 | VACCINIA NAIVE SUBJECTS WITH ACTIVE ATOPIC DERMATITIS | N | 1 |
| POX-MVA-008-2025-048 | VISIT 2 | 556 | 227 | VACCINIA NAIVE SUBJECTS WITH ACTIVE ATOPIC DERMATITIS | N | 1 |
| POX-MVA-008-2025-048 | VISIT 3 | 1191 | 480 | VACCINIA NAIVE SUBJECTS WITH ACTIVE ATOPIC DERMATITIS | N | 1 |
| POX-MVA-008-2025-048 | VISIT 4 | 718 | 393 | VACCINIA NAIVE SUBJECTS WITH ACTIVE ATOPIC DERMATITIS | N | 1 |
| POX-MVA-008-2025-048 | VISIT 5 | 413 | 233 | VACCINIA NAIVE SUBJECTS WITH ACTIVE ATOPIC DERMATITIS | N | 1 |
| POX-MVA-008-2026-003 | VISIT 1 | 1 | 1 | VACCINIA NAIVE SUBJECTS WITH ACTIVE ATOPIC DERMATITIS | N | 2 |
| POX-MVA-008-2026-003 | VISIT 2 | 1 | 1 | VACCINIA NAIVE SUBJECTS WITH ACTIVE ATOPIC DERMATITIS | N | 2 |
| POX-MVA-008-2026-003 | VISIT 3 | 200 | 1 | VACCINIA NAIVE SUBJECTS WITH ACTIVE ATOPIC DERMATITIS | N | 2 |
| POX-MVA-008-2026-003 | VISIT 4 | 7248 | 147 | VACCINIA NAIVE SUBJECTS WITH ACTIVE ATOPIC DERMATITIS | N | 2 |
| POX-MVA-008-2026-003 | VISIT 5 | 2443 | 242 | VACCINIA NAIVE SUBJECTS WITH ACTIVE ATOPIC DERMATITIS | N | 2 |
| POX-MVA-008-2026-003 | FOLLOW-UP VISIT | 205 | 15 | VACCINIA NAIVE SUBJECTS WITH ACTIVE ATOPIC DERMATITIS | N | 2 |
| POX-MVA-008-2026-005 | VISIT 1 | 50 | 1 | VACCINIA NAIVE SUBJECTS WITH HISTORY OF ATOPIC DERMATITIS | N | 2 |
| POX-MVA-008-2026-005 | VISIT 2 | 50 | 1 | VACCINIA NAIVE SUBJECTS WITH HISTORY OF ATOPIC DERMATITIS | N | 2 |
| POX-MVA-008-2026-005 | VISIT 3 | 50 | 1 | VACCINIA NAIVE SUBJECTS WITH HISTORY OF ATOPIC DERMATITIS | N | 2 |
| POX-MVA-008-2026-005 | VISIT 4 | 697 | 15 | VACCINIA NAIVE SUBJECTS WITH HISTORY OF ATOPIC DERMATITIS | N | 2 |
| POX-MVA-008-2026-005 | VISIT 5 | 386 | 89 | VACCINIA NAIVE SUBJECTS WITH HISTORY OF ATOPIC DERMATITIS | N | 2 |
| POX-MVA-008-2026-005 | FOLLOW-UP VISIT | 50 | 1 | VACCINIA NAIVE SUBJECTS WITH HISTORY OF ATOPIC DERMATITIS | N | 2 |
| POX-MVA-008-2026-006 | VISIT 1 | 1 | 1 | HEALTHY, VACCINIA NAIVE SUBJECTS WITHOUT ATOPIC DISEASE | N | 2 |
| POX-MVA-008-2026-006 | VISIT 2 | 1 | 1 | HEALTHY, VACCINIA NAIVE SUBJECTS WITHOUT ATOPIC DISEASE | N | 2 |
| POX-MVA-008-2026-006 | VISIT 3 | 50 | 1 | HEALTHY, VACCINIA NAIVE SUBJECTS WITHOUT ATOPIC DISEASE | N | 2 |
| POX-MVA-008-2026-006 | VISIT 4 | 413 | 15 | HEALTHY, VACCINIA NAIVE SUBJECTS WITHOUT ATOPIC DISEASE | N | 2 |
| POX-MVA-008-2026-006 | VISIT 5 | 239 | 15 | HEALTHY, VACCINIA NAIVE SUBJECTS WITHOUT ATOPIC DISEASE | N | 2 |
| POX-MVA-008-2026-007 | VISIT 1 | 1 | 1 | HEALTHY, VACCINIA NAIVE SUBJECTS WITHOUT ATOPIC DISEASE | N | 2 |
| POX-MVA-008-2026-007 | VISIT 2 | 50 | 1 | HEALTHY, VACCINIA NAIVE SUBJECTS WITHOUT ATOPIC DISEASE | N | 2 |
| POX-MVA-008-2026-007 | VISIT 3 | 227 | 15 | HEALTHY, VACCINIA NAIVE SUBJECTS WITHOUT ATOPIC DISEASE | N | 2 |
| POX-MVA-008-2026-007 | VISIT 4 | 900 | 15 | HEALTHY, VACCINIA NAIVE SUBJECTS WITHOUT ATOPIC DISEASE | N | 2 |
| POX-MVA-008-2026-007 | VISIT 5 | 501 | 15 | HEALTHY, VACCINIA NAIVE SUBJECTS WITHOUT ATOPIC DISEASE | N | 2 |
| POX-MVA-008-2026-008 | VISIT 1 | 1 | 1 | VACCINIA NAIVE SUBJECTS WITH HISTORY OF ATOPIC DERMATITIS | N | 2 |
| POX-MVA-008-2026-008 | VISIT 2 | 1 | 1 | VACCINIA NAIVE SUBJECTS WITH HISTORY OF ATOPIC DERMATITIS | N | 2 |
| POX-MVA-008-2026-008 | VISIT 3 | 200 | 1 | VACCINIA NAIVE SUBJECTS WITH HISTORY OF ATOPIC DERMATITIS | N | 2 |
| POX-MVA-008-2026-008 | VISIT 4 | 929 | 117 | VACCINIA NAIVE SUBJECTS WITH HISTORY OF ATOPIC DERMATITIS | N | 2 |
| POX-MVA-008-2026-008 | VISIT 5 | 746 | 1 | VACCINIA NAIVE SUBJECTS WITH HISTORY OF ATOPIC DERMATITIS | N | 2 |
| POX-MVA-008-2026-008 | FOLLOW-UP VISIT | 50 | 15 | VACCINIA NAIVE SUBJECTS WITH HISTORY OF ATOPIC DERMATITIS | N | 2 |
| POX-MVA-008-2026-010 | VISIT 1 | 1 | 1 | HEALTHY, VACCINIA NAIVE SUBJECTS WITHOUT ATOPIC DISEASE | N | 2 |
| POX-MVA-008-2026-010 | VISIT 2 | 1 | 1 | HEALTHY, VACCINIA NAIVE SUBJECTS WITHOUT ATOPIC DISEASE | N | 2 |
| POX-MVA-008-2026-010 | VISIT 3 | 50 | 1 | HEALTHY, VACCINIA NAIVE SUBJECTS WITHOUT ATOPIC DISEASE | N | 2 |
| POX-MVA-008-2026-010 | VISIT 4 | 910 | 164 | HEALTHY, VACCINIA NAIVE SUBJECTS WITHOUT ATOPIC DISEASE | N | 2 |
| POX-MVA-008-2026-010 | VISIT 5 | 728 | 99 | HEALTHY, VACCINIA NAIVE SUBJECTS WITHOUT ATOPIC DISEASE | N | 2 |
| POX-MVA-008-2026-011 | VISIT 1 | 1 | 1 | HEALTHY, VACCINIA NAIVE SUBJECTS WITHOUT ATOPIC DISEASE | N | 2 |
| POX-MVA-008-2026-011 | VISIT 2 | 1 | 1 | HEALTHY, VACCINIA NAIVE SUBJECTS WITHOUT ATOPIC DISEASE | N | 2 |
| POX-MVA-008-2026-011 | VISIT 3 | 50 | 1 | HEALTHY, VACCINIA NAIVE SUBJECTS WITHOUT ATOPIC DISEASE | N | 2 |
| POX-MVA-008-2026-011 | VISIT 4 | 426 | 1 | HEALTHY, VACCINIA NAIVE SUBJECTS WITHOUT ATOPIC DISEASE | N | 2 |
| POX-MVA-008-2026-011 | VISIT 5 | 200 | 1 | HEALTHY, VACCINIA NAIVE SUBJECTS WITHOUT ATOPIC DISEASE | N | 2 |
| POX-MVA-008-2026-012 | VISIT 1 | 1 | 1 | HEALTHY, VACCINIA NAIVE SUBJECTS WITHOUT ATOPIC DISEASE | N | 2 |
| POX-MVA-008-2026-012 | VISIT 2 | 1 | 1 | HEALTHY, VACCINIA NAIVE SUBJECTS WITHOUT ATOPIC DISEASE | N | 2 |
| POX-MVA-008-2026-012 | VISIT 3 | 50 | 1 | HEALTHY, VACCINIA NAIVE SUBJECTS WITHOUT ATOPIC DISEASE | N | 2 |
| POX-MVA-008-2026-012 | VISIT 4 | 1025 | 96 | HEALTHY, VACCINIA NAIVE SUBJECTS WITHOUT ATOPIC DISEASE | N | 2 |
| POX-MVA-008-2026-012 | VISIT 5 | 435 | 15 | HEALTHY, VACCINIA NAIVE SUBJECTS WITHOUT ATOPIC DISEASE | N | 2 |
| POX-MVA-008-2026-013 | VISIT 1 | 1 | 1 | HEALTHY, VACCINIA NAIVE SUBJECTS WITHOUT ATOPIC DISEASE | N | 2 |
| POX-MVA-008-2026-013 | VISIT 2 | 1 | 1 | HEALTHY, VACCINIA NAIVE SUBJECTS WITHOUT ATOPIC DISEASE | N | 2 |
| POX-MVA-008-2026-013 | VISIT 3 | 100 | 1 | HEALTHY, VACCINIA NAIVE SUBJECTS WITHOUT ATOPIC DISEASE | N | 2 |
| POX-MVA-008-2026-013 | VISIT 4 | 2836 | 245 | HEALTHY, VACCINIA NAIVE SUBJECTS WITHOUT ATOPIC DISEASE | N | 2 |
| POX-MVA-008-2026-013 | VISIT 5 | 1312 | 15 | HEALTHY, VACCINIA NAIVE SUBJECTS WITHOUT ATOPIC DISEASE | N | 2 |
| POX-MVA-008-2026-014 | VISIT 1 | 1 | 1 | HEALTHY, VACCINIA NAIVE SUBJECTS WITHOUT ATOPIC DISEASE | N | 2 |
| POX-MVA-008-2026-014 | VISIT 2 | 1 | 1 | HEALTHY, VACCINIA NAIVE SUBJECTS WITHOUT ATOPIC DISEASE | N | 2 |
| POX-MVA-008-2026-014 | VISIT 3 | 1 | 1 | HEALTHY, VACCINIA NAIVE SUBJECTS WITHOUT ATOPIC DISEASE | N | 2 |
| POX-MVA-008-2026-014 | VISIT 4 | 50 | 1 | HEALTHY, VACCINIA NAIVE SUBJECTS WITHOUT ATOPIC DISEASE | N | 2 |
| POX-MVA-008-2026-014 | VISIT 5 | 1 | 1 | HEALTHY, VACCINIA NAIVE SUBJECTS WITHOUT ATOPIC DISEASE | N | 2 |
| POX-MVA-008-2026-016 | VISIT 1 | 1 | 1 | HEALTHY, VACCINIA NAIVE SUBJECTS WITHOUT ATOPIC DISEASE | N | 2 |
| POX-MVA-008-2026-016 | VISIT 2 | 1 | 1 | HEALTHY, VACCINIA NAIVE SUBJECTS WITHOUT ATOPIC DISEASE | N | 2 |
| POX-MVA-008-2026-016 | VISIT 3 | 100 | 1 | HEALTHY, VACCINIA NAIVE SUBJECTS WITHOUT ATOPIC DISEASE | N | 2 |
| POX-MVA-008-2026-016 | VISIT 4 | 1656 | 1 | HEALTHY, VACCINIA NAIVE SUBJECTS WITHOUT ATOPIC DISEASE | N | 2 |
| POX-MVA-008-2026-016 | VISIT 5 | 848 | 109 | HEALTHY, VACCINIA NAIVE SUBJECTS WITHOUT ATOPIC DISEASE | N | 2 |
| POX-MVA-008-2026-017 | VISIT 1 | 1 | 1 | VACCINIA NAIVE SUBJECTS WITH HISTORY OF ATOPIC DERMATITIS | N | 2 |
| POX-MVA-008-2026-017 | VISIT 2 | 1 | 1 | VACCINIA NAIVE SUBJECTS WITH HISTORY OF ATOPIC DERMATITIS | N | 2 |
| POX-MVA-008-2026-017 | VISIT 3 | 246 | 1 | VACCINIA NAIVE SUBJECTS WITH HISTORY OF ATOPIC DERMATITIS | N | 2 |
| POX-MVA-008-2026-017 | VISIT 4 | 957 | 15 | VACCINIA NAIVE SUBJECTS WITH HISTORY OF ATOPIC DERMATITIS | N | 2 |
| POX-MVA-008-2026-017 | VISIT 5 | 576 | 15 | VACCINIA NAIVE SUBJECTS WITH HISTORY OF ATOPIC DERMATITIS | N | 2 |
| POX-MVA-008-2026-017 | FOLLOW-UP VISIT | 50 | 1 | VACCINIA NAIVE SUBJECTS WITH HISTORY OF ATOPIC DERMATITIS | N | 2 |
| POX-MVA-008-2026-018 | VISIT 1 | 1 | 1 | HEALTHY, VACCINIA NAIVE SUBJECTS WITHOUT ATOPIC DISEASE | N | 2 |
| POX-MVA-008-2026-018 | VISIT 2 | 1 | 1 | HEALTHY, VACCINIA NAIVE SUBJECTS WITHOUT ATOPIC DISEASE | N | 2 |
| POX-MVA-008-2026-018 | VISIT 3 | 100 | 1 | HEALTHY, VACCINIA NAIVE SUBJECTS WITHOUT ATOPIC DISEASE | N | 2 |
| POX-MVA-008-2026-018 | VISIT 4 | 935 | 309 | HEALTHY, VACCINIA NAIVE SUBJECTS WITHOUT ATOPIC DISEASE | N | 2 |
| POX-MVA-008-2026-018 | VISIT 5 | 501 | 15 | HEALTHY, VACCINIA NAIVE SUBJECTS WITHOUT ATOPIC DISEASE | N | 2 |
| POX-MVA-008-2026-020 | VISIT 1 | 1 | 1 | HEALTHY, VACCINIA NAIVE SUBJECTS WITHOUT ATOPIC DISEASE | N | 2 |
| POX-MVA-008-2026-020 | VISIT 2 | 100 | 1 | HEALTHY, VACCINIA NAIVE SUBJECTS WITHOUT ATOPIC DISEASE | N | 2 |
| POX-MVA-008-2026-020 | VISIT 3 | 100 | 1 | HEALTHY, VACCINIA NAIVE SUBJECTS WITHOUT ATOPIC DISEASE | N | 2 |
| POX-MVA-008-2026-020 | VISIT 4 | 1023 | 162 | HEALTHY, VACCINIA NAIVE SUBJECTS WITHOUT ATOPIC DISEASE | N | 2 |
| POX-MVA-008-2026-020 | VISIT 5 | 416 | 138 | HEALTHY, VACCINIA NAIVE SUBJECTS WITHOUT ATOPIC DISEASE | N | 2 |
| POX-MVA-008-2026-021 | VISIT 1 | 1 | 1 | HEALTHY, VACCINIA NAIVE SUBJECTS WITHOUT ATOPIC DISEASE | N | 2 |
| POX-MVA-008-2026-021 | VISIT 2 | 1 | 1 | HEALTHY, VACCINIA NAIVE SUBJECTS WITHOUT ATOPIC DISEASE | N | 2 |
| POX-MVA-008-2026-021 | VISIT 4 | 5502 | 611 | HEALTHY, VACCINIA NAIVE SUBJECTS WITHOUT ATOPIC DISEASE | N | 2 |
| POX-MVA-008-2026-021 | VISIT 5 | 1178 | 236 | HEALTHY, VACCINIA NAIVE SUBJECTS WITHOUT ATOPIC DISEASE | N | 2 |
| POX-MVA-008-2026-024 | VISIT 1 | 1 | 1 | HEALTHY, VACCINIA NAIVE SUBJECTS WITHOUT ATOPIC DISEASE | N | 2 |
| POX-MVA-008-2026-024 | VISIT 2 | 1 | 1 | HEALTHY, VACCINIA NAIVE SUBJECTS WITHOUT ATOPIC DISEASE | N | 2 |
| POX-MVA-008-2026-024 | VISIT 3 | 823 | 1 | HEALTHY, VACCINIA NAIVE SUBJECTS WITHOUT ATOPIC DISEASE | N | 2 |
| POX-MVA-008-2026-024 | VISIT 4 | 4375 | 250 | HEALTHY, VACCINIA NAIVE SUBJECTS WITHOUT ATOPIC DISEASE | N | 2 |
| POX-MVA-008-2026-024 | VISIT 5 | 991 | 15 | HEALTHY, VACCINIA NAIVE SUBJECTS WITHOUT ATOPIC DISEASE | N | 2 |
| POX-MVA-008-2026-026 | VISIT 1 | 1 | 1 | HEALTHY, VACCINIA NAIVE SUBJECTS WITHOUT ATOPIC DISEASE | N | 2 |
| POX-MVA-008-2026-026 | VISIT 2 | 1 | 1 | HEALTHY, VACCINIA NAIVE SUBJECTS WITHOUT ATOPIC DISEASE | N | 2 |
| POX-MVA-008-2026-026 | VISIT 3 | 50 | 1 | HEALTHY, VACCINIA NAIVE SUBJECTS WITHOUT ATOPIC DISEASE | N | 2 |
| POX-MVA-008-2026-026 | VISIT 4 | 396 | 205 | HEALTHY, VACCINIA NAIVE SUBJECTS WITHOUT ATOPIC DISEASE | N | 2 |
| POX-MVA-008-2026-026 | VISIT 5 | 294 | 15 | HEALTHY, VACCINIA NAIVE SUBJECTS WITHOUT ATOPIC DISEASE | N | 2 |
| POX-MVA-008-2026-028 | VISIT 1 | 1 | 1 | VACCINIA NAIVE SUBJECTS WITH HISTORY OF ATOPIC DERMATITIS | N | 1 |
| POX-MVA-008-2026-028 | VISIT 2 | 1 | 1 | VACCINIA NAIVE SUBJECTS WITH HISTORY OF ATOPIC DERMATITIS | N | 1 |
| POX-MVA-008-2026-029 | VISIT 1 | 1 | 1 | HEALTHY, VACCINIA NAIVE SUBJECTS WITHOUT ATOPIC DISEASE | N | 1 |
| POX-MVA-008-2026-029 | VISIT 2 | 1 | 1 | HEALTHY, VACCINIA NAIVE SUBJECTS WITHOUT ATOPIC DISEASE | N | 1 |
| POX-MVA-008-2026-029 | VISIT 3 | 50 | 1 | HEALTHY, VACCINIA NAIVE SUBJECTS WITHOUT ATOPIC DISEASE | N | 1 |
| POX-MVA-008-2026-029 | VISIT 4 | 50 | 1 | HEALTHY, VACCINIA NAIVE SUBJECTS WITHOUT ATOPIC DISEASE | N | 1 |
| POX-MVA-008-2026-029 | VISIT 5 | 50 | 1 | HEALTHY, VACCINIA NAIVE SUBJECTS WITHOUT ATOPIC DISEASE | N | 1 |
| POX-MVA-008-2026-033 | VISIT 1 | 1 | 1 | HEALTHY, VACCINIA NAIVE SUBJECTS WITHOUT ATOPIC DISEASE | N | 1 |
| POX-MVA-008-2026-033 | VISIT 2 | 1 | 1 | HEALTHY, VACCINIA NAIVE SUBJECTS WITHOUT ATOPIC DISEASE | N | 1 |
| POX-MVA-008-2026-033 | VISIT 3 | 50 | 1 | HEALTHY, VACCINIA NAIVE SUBJECTS WITHOUT ATOPIC DISEASE | N | 1 |
| POX-MVA-008-2026-033 | VISIT 4 | 1 | 1 | HEALTHY, VACCINIA NAIVE SUBJECTS WITHOUT ATOPIC DISEASE | N | 1 |
| POX-MVA-008-2026-033 | VISIT 5 | 1 | 1 | HEALTHY, VACCINIA NAIVE SUBJECTS WITHOUT ATOPIC DISEASE | N | 1 |
| POX-MVA-008-2026-047 | VISIT 1 | 1 | 1 | HEALTHY, VACCINIA NAIVE SUBJECTS WITHOUT ATOPIC DISEASE | N | 1 |
| POX-MVA-008-2026-047 | VISIT 2 | 1 | 1 | HEALTHY, VACCINIA NAIVE SUBJECTS WITHOUT ATOPIC DISEASE | N | 1 |
| POX-MVA-008-2026-047 | VISIT 5 | 100 | 1 | HEALTHY, VACCINIA NAIVE SUBJECTS WITHOUT ATOPIC DISEASE | N | 1 |
| POX-MVA-008-2026-054 | VISIT 1 | 1 | 1 | HEALTHY, VACCINIA NAIVE SUBJECTS WITHOUT ATOPIC DISEASE | N | 2 |
| POX-MVA-008-2026-054 | VISIT 2 | 1 | 1 | HEALTHY, VACCINIA NAIVE SUBJECTS WITHOUT ATOPIC DISEASE | N | 2 |
| POX-MVA-008-2026-054 | VISIT 3 | 237 | 15 | HEALTHY, VACCINIA NAIVE SUBJECTS WITHOUT ATOPIC DISEASE | N | 2 |
| POX-MVA-008-2026-059 | VISIT 1 | 1 | 1 | HEALTHY, VACCINIA NAIVE SUBJECTS WITHOUT ATOPIC DISEASE | N | 1 |
| POX-MVA-008-2026-059 | VISIT 2 | 1 | 1 | HEALTHY, VACCINIA NAIVE SUBJECTS WITHOUT ATOPIC DISEASE | N | 1 |
| POX-MVA-008-2026-059 | VISIT 3 | 50 | 1 | HEALTHY, VACCINIA NAIVE SUBJECTS WITHOUT ATOPIC DISEASE | N | 1 |
| POX-MVA-008-2026-059 | VISIT 4 | 50 | 1 | HEALTHY, VACCINIA NAIVE SUBJECTS WITHOUT ATOPIC DISEASE | N | 1 |
| POX-MVA-008-2026-059 | VISIT 5 | 50 | 1 | HEALTHY, VACCINIA NAIVE SUBJECTS WITHOUT ATOPIC DISEASE | N | 1 |
| POX-MVA-008-2026-060 | VISIT 1 | 1 | 1 | VACCINIA NAIVE SUBJECTS WITH ACTIVE ATOPIC DERMATITIS | N | 1 |
| POX-MVA-008-2026-060 | VISIT 2 | 50 | 1 | VACCINIA NAIVE SUBJECTS WITH ACTIVE ATOPIC DERMATITIS | N | 1 |
| POX-MVA-008-2026-060 | VISIT 3 | 222 | 1 | VACCINIA NAIVE SUBJECTS WITH ACTIVE ATOPIC DERMATITIS | N | 1 |
| POX-MVA-008-2026-060 | VISIT 4 | 211 | 1 | VACCINIA NAIVE SUBJECTS WITH ACTIVE ATOPIC DERMATITIS | N | 1 |
| POX-MVA-008-2026-060 | VISIT 5 | 50 | 1 | VACCINIA NAIVE SUBJECTS WITH ACTIVE ATOPIC DERMATITIS | N | 1 |
| POX-MVA-008-2026-066 | VISIT 1 | 1 | 1 | VACCINIA NAIVE SUBJECTS WITH ACTIVE ATOPIC DERMATITIS | N | 2 |
| POX-MVA-008-2026-066 | VISIT 2 | 1 | 1 | VACCINIA NAIVE SUBJECTS WITH ACTIVE ATOPIC DERMATITIS | N | 2 |
| POX-MVA-008-2026-066 | VISIT 3 | 50 | 1 | VACCINIA NAIVE SUBJECTS WITH ACTIVE ATOPIC DERMATITIS | N | 2 |
| POX-MVA-008-2026-066 | VISIT 4 | 806 | 15 | VACCINIA NAIVE SUBJECTS WITH ACTIVE ATOPIC DERMATITIS | N | 2 |
| POX-MVA-008-2026-066 | VISIT 5 | 604 | 15 | VACCINIA NAIVE SUBJECTS WITH ACTIVE ATOPIC DERMATITIS | N | 2 |
| POX-MVA-008-2026-071 | VISIT 1 | 1 | 1 | VACCINIA NAIVE SUBJECTS WITH HISTORY OF ATOPIC DERMATITIS | N | 2 |
| POX-MVA-008-2026-071 | VISIT 2 | 1 | 1 | VACCINIA NAIVE SUBJECTS WITH HISTORY OF ATOPIC DERMATITIS | N | 2 |
| POX-MVA-008-2026-071 | VISIT 3 | 100 | 1 | VACCINIA NAIVE SUBJECTS WITH HISTORY OF ATOPIC DERMATITIS | N | 2 |
| POX-MVA-008-2026-071 | VISIT 4 | 1031 | 149 | VACCINIA NAIVE SUBJECTS WITH HISTORY OF ATOPIC DERMATITIS | N | 2 |
| POX-MVA-008-2026-071 | VISIT 5 | 471 | 15 | VACCINIA NAIVE SUBJECTS WITH HISTORY OF ATOPIC DERMATITIS | N | 2 |
| POX-MVA-008-2026-072 | VISIT 1 | 1 | 1 | VACCINIA NAIVE SUBJECTS WITH ACTIVE ATOPIC DERMATITIS | N | 2 |
| POX-MVA-008-2026-072 | VISIT 2 | 50 | 15 | VACCINIA NAIVE SUBJECTS WITH ACTIVE ATOPIC DERMATITIS | N | 2 |
| POX-MVA-008-2026-072 | VISIT 3 | 200 | 15 | VACCINIA NAIVE SUBJECTS WITH ACTIVE ATOPIC DERMATITIS | N | 2 |
| POX-MVA-008-2026-072 | VISIT 4 | 264 | 150 | VACCINIA NAIVE SUBJECTS WITH ACTIVE ATOPIC DERMATITIS | N | 2 |
| POX-MVA-008-2026-072 | VISIT 5 | 255 | 105 | VACCINIA NAIVE SUBJECTS WITH ACTIVE ATOPIC DERMATITIS | N | 2 |
